# Differential expression of apoptotic genes *PDIA3 *and *MAP3K5 *distinguishes between low- and high-risk prostate cancer

**DOI:** 10.1186/1476-4598-8-130

**Published:** 2009-12-27

**Authors:** Nicole Chui Pressinotti, Helmut Klocker, Georg Schäfer, Van-Duc Luu, Markus Ruschhaupt, Ruprecht Kuner, Eberhard Steiner, Annemarie Poustka, Georg Bartsch, Holger Sültmann

**Affiliations:** 1 German Cancer Research Center, Division of Molecular Genome Analysis, Im Neuenheimer Feld 580, D-69120 Heidelberg, Germany; 2 Abbott GmbH & Co. KG, Max-Planck Ring 2, Wiesbaden, Germany; 3 University Clinics for Urology, Innsbruck Medical University, Anichstraße 35, A-6020 Innsbruck, Austria; 4 Institute for Pathology, Innsbruck Medical University, Müllerstraße 40, A-6020 Innsbruck, Austria; 5 Applied Biosystems, Grundstrasse 10, CH-6343 Rotkreuz, Switzerland

## Abstract

**Background:**

Despite recent progress in the identification of genetic and molecular alterations in prostate cancer, markers associated with tumor progression are scarce. Therefore precise diagnosis of patients and prognosis of the disease remain difficult. This study investigated novel molecular markers discriminating between low and highly aggressive types of prostate cancer.

**Results:**

Using 52 microdissected cell populations of low- and high-risk prostate tumors, we identified via global cDNA microarrays analysis almost 1200 genes being differentially expressed among these groups. These genes were analyzed by statistical, pathway and gene enrichment methods. Twenty selected candidate genes were verified by quantitative real time PCR and immunohistochemistry. In concordance with the mRNA levels, two genes *MAP3K5 *and *PDIA3 *exposed differential protein expression. Functional characterization of *PDIA3 *revealed a pro-apoptotic role of this gene in PC3 prostate cancer cells.

**Conclusions:**

Our analyses provide deeper insights into the molecular changes occurring during prostate cancer progression. The genes *MAP3K5 *and *PDIA3 *are associated with malignant stages of prostate cancer and therefore provide novel potential biomarkers.

## Background

Prostate cancer is the most frequent cancer diagnosed in men (20.3% of the total), followed by lung (17.2%) and colorectal cancer (12.8%) [1]. Measuring prostate specific antigen (PSA) has been a matter of routine to detect prostate cancer, but is insufficient to distinguish between different tumor grades. The Gleason Grading System is commonly used for histology-based grading of prostate cancer tissue [2]. Since prostate tumors are often multifocal, the Gleason Score (GS) is the sum of the two most prevalent tumor patterns, which are graded 1 (CA1) as the most differentiated and 5 (CA5) as the least differentiated pattern of cancerous glands. Other methods for sub-classification have been described in recent reports [3]. These indicate that translocations fusing the strong androgen-responsive gene *TMPRSS2 *with *ERG *or other oncogenic *ETS *factors may facilitate prostate cancer development. It has been proposed that the presence or absence of this genetic rearrangement may be used, much like the Gleason grading system, as a diagnostic tool to extract prognostically relevant sub-classifications of this cancer [4].

The discrimination between different tumor grades is important with respect to treatment decisions: Currently, many men who are diagnosed with GS 6 prostate cancer are often "over"-treated and risk suffering from urinary and sexual dysfunction [5]. Therefore, it is important to develop a sensitive and specific diagnostic tool to distinguish between different tumor grades. To address this problem, many groups have recently started to profile gene expression levels in prostate tumor tissues to identify deregulated genes during disease progression. However, although many of these have addressed the question of molecular differences between normal, tumor, benign prostatic hyperplasia (BPH), and the putative precursor lesion prostatic intraepithelial neoplasia (PIN), little is still known about molecular changes between low- and high-risk tumors [6-9].

In the present study, we performed microarray-based gene expression profile analysis of 65 microdissected tissues comprising 25 samples of GS 6, 27 of GS 8-10 and 13 non cancerous samples. We sought to identify biological markers of distinct functional groups for the discrimination between low- and high-risk tumors. Overall, we found 20 genes with a significant alteration in expression between high-risk compared to low-risk tumors. Two of these genes exhibited Gleason grade associated protein expression in tumor tissues, which could serve as a valuable diagnostic tool in the future.

## Results

### mRNA expression analysis revealed large expression differences between GS 6 and GS 8-10 tumors

To selectively isolate pure populations of prostate epithelial cancer cells with different Gleason Scores, we first applied laser-capture microdissection. We monitored the gene expression levels by hybridization of twice-amplified RNA to cDNA microarrays representing ~37500 mapped genes. In total, we hybridized 65 RNA samples derived from 13 benign and 52 prostate cancer tissue comprising 25 samples with Gleason Score (GS) 6 and 27 samples with GS 8-10 (Table 1). After quality assessment of microarray hybridizations, we subjected gene expression profiles to SAM [10]. Numbers of deregulated genes identified by SAM analyses are summarized in Table 2, and complete gene lists are provided (see additional file 1 and additional file 2).

**Table 1:** Characteristics of study population

Stage	N =	median age
Benign	13	63

GS 6	25/0*	60
	
GS 8	13/4*	66
	
GS 9	13/2*	
	
GS 10	1/0*	

* matched benign

**Table 2:** Number of differentially expressed genes (FDR < 5%)

Comparison (SAM test)	up	down
Normal ↔ Tumor	243	2390

Normal ↔ Tumor (GS6)	463	2016

Normal ↔ Tumor (GS8-10)	454	2001

Tumor (GS6) ↔ Tumor (GS8-10)	**1141**	**54**

For the identification of grade-discriminating genes, we compared the expression levels of GS 6 with GS 8-10 tumors. SAM analysis revealed 1141 up-regulated and 54 down-regulated non-redundant genes in advanced tumors (FDR 5%; see additional file 1). For validation, we compared our data with an independent study from True and coworkers, who reported 86 genes as deregulated during tumor progression from low to high GS [6]. Of these, we identified 24 genes (28%) which all displayed the same tendency as in the original report (see additional file 3). Another comparison to the study of Lapointe and coworkers [7], who described 41 genes to be associated to a higher Gleason score revealed an overlap of six genes (*BGN*, *COL1A2*, *COL3A1*, *PLA2G2A*, *SPARC*, *VCAN*).

To identify biological processes associated with tumor progression, we performed gene ontology analysis with genes differentially regulated between low- and high-risk tumors. In order to extract highly significant canonical pathways, each gene symbol was mapped to its corresponding gene object in the IPA Knowledge Base, and networks were generated. Significant canonical pathways were related to actin-mediated processes, e.g. regulation of actin-based motility mediated by Rho-family GTPases (11 of 92 annotated genes, p = 5.23E-03) and actin cytoskeleton signaling (19 of 221 annotated genes, p = 1.4E-02). In addition, various metabolic processes, including oxidative phosphorylation (22 of 158 annotated genes; p = 8.95E-06) and protein ubiquitination (19 of 205 annotated genes; p = 5.6E-03) were deregulated in high-risk tumors. In order to extract as many genes as possible involved in apoptotic processes we used two further GO analysis tools (FatiGO [11] and GOstat [12]) and identified a set of 46 genes associated with apoptosis (GOstat p = 0.00033; see additional file 4).

Additionally, we compared gene signatures between normal and tumor tissue, which lead to the identification of > 2500 deregulated genes (FDR 5%) of which 2390 genes were down and 243 up-regulated (see additional file 2). We performed separate SAM analyses between normal and GS 6 or GS 8-10 and revealed 2016 and 2001 down-regulated as well as 463 and 454 up-regulated non redundant genes (Table 2). Of these, 1197 genes were deregulated with the same tendency in both tumor groups. Interestingly, three genes (*VCAN*, *CLK1 *and *TMEM16G*) revealed opposite expression levels in these comparisons. *VCAN *and *CLK1 *were found to be significantly down-regulated in GS 6, but up-regulated in high-risk tumors in comparison to normal tissue. In agreement with these results, over-expression of *VCAN *and *CLK1 *between primary prostate cancer and metastatic cancer has recently been described [9]. In contrast *TMEM16G*, for which the microarray findings were supported by qRT-PCR data, showed the opposite trend. *TMEM16G *was found up-regulated between normal and GS 6 tissue, but down-regulated between normal and GS 8 as well as between GS 6 and GS 8 tissues. In a recent study, *TMEM16G *was described as a prostate-specific plasma membrane protein promoting cell-cell contact in the prostate cancer cell line LNCaP [13].

### Validation of selected genes by qRT-PCR

**Figure 1:**
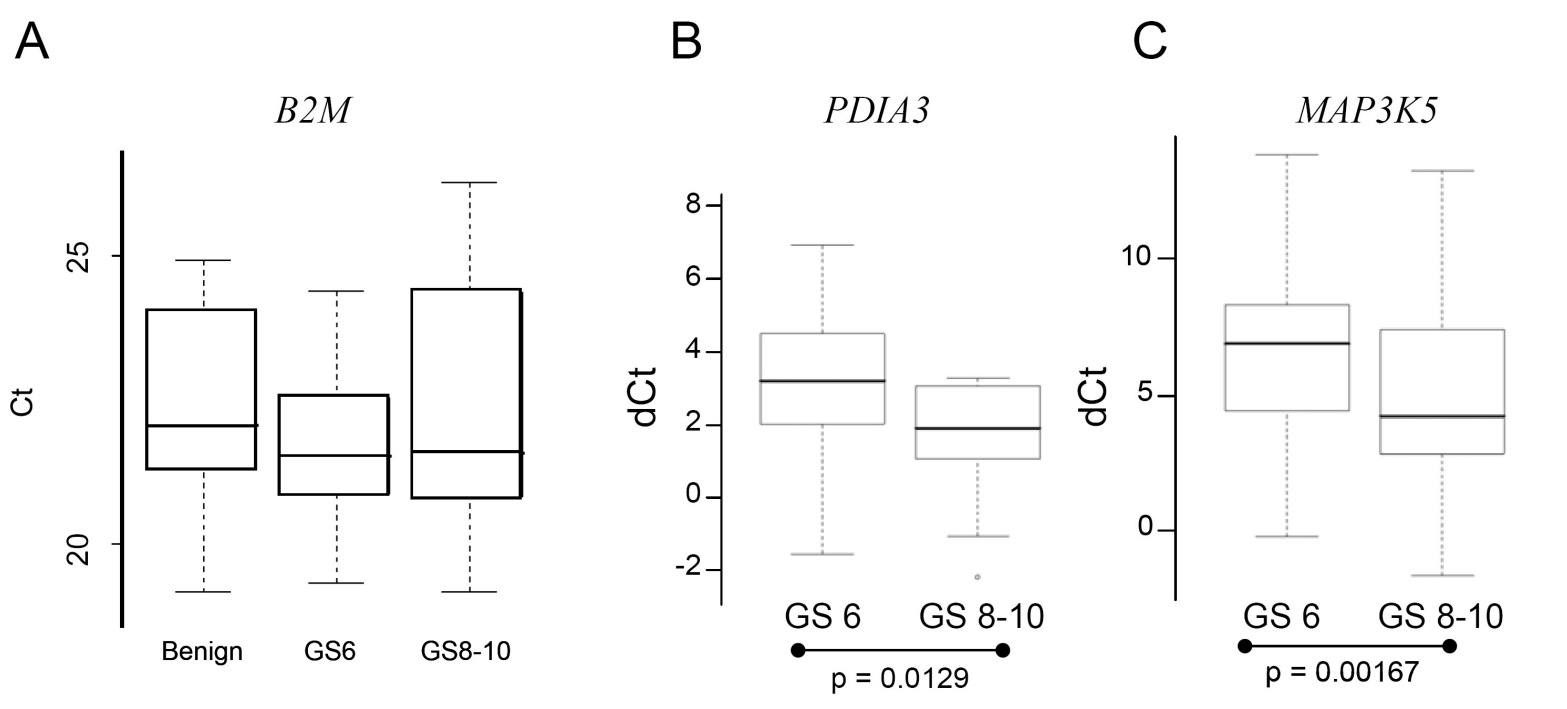
**Validation of *PDIA3 *and *MAP3K5 *mRNA expression via qRT PCR**. **(A) **The housekeeping gene β-2 microglobulin (*B2M*) was chosen due to its even expression (mean Ct value) in each analyzed group (benign, GS 6 and GS 8-10 tissue). **(B, C) **Mean normalized expression levels (dCt) of GS 6 and GS 8-10 was determined for *PDIA3 *(B) and *MAP3K5 *(C). Results showed a significant increase of transcript abundance levels in GS 8-10 tumors in comparison to GS 6 tumors.

Based on the microarray gene expression differences between GS 6 and GS 8-10 tumors (see additional file 1), 68 genes were validated in the same cohort via quantitative real time PCR (see additional file 5). *B2M *(β2-microglobulin) was used as a housekeeping gene due to its even expression in all analyzed patient groups (Figure 1A). We focused on a selection of genes that are linked to cancer-relevant gene ontology categories like apoptosis, cell morphology, metabolism and ubiquitylation. For example, 24/68 selected genes are functionally associated to apoptosis (see additional file 4).

In the qRT-PCR analysis of the 68 genes, 23 were significantly deregulated between low- and high-risk tumors (Wilcoxon p < 0.1; see additional file 5). Twenty of these (87%; Table 3) were in concordance with the microarray results. Thirty-three genes were determined as non-significant and 12 genes exhibited Ct values below detection level. Deregulation of the apoptotic process plays a major role in tumorigenesis and influences therapeutic outcome [14]. In total, 11 of 20 significantly verified genes are involved in apoptotic processes (*PDIA3*, *MAP3K5, ANXA5*, *VDAC1*, *NGFRAP1*, *TEGT*, *NPM1*, *VCP*, *TRAF4*, *HMGB1*, *and ROCK1*). The two genes *MAP3K5 *and *PDIA3 *were selected for *in-depth *analysis because of their association to apoptosis and previous findings in cancer studies. QRT-PCR expression patterns of *PDIA3 *and *MAP3K5 *are given in Figure 1B and 1C.

**Table 3:** qRT-PCR verified differentially expressed genes between GS 6 and GS 8-10

No.	Gene symbol	RZPD ID	IMAGE ID	GO ID	GO term	Microarray		qRT PCR	
						q-value (%)	fold change	p-value (%)	fold change
1	*ANXA5*	IMAGp998G1518	66470	GO:0006916	anti-apoptosis	0.00	1.47	0.10	4.28

2	*APLP2*	IMAGp998M03537	248618	GO:0016021	transmembrane	0.94	1.22	6.10	2.83

3	*COL1A2*	IMAGp998D09597	271448	GO:0001501	skeletal development	0.40	1.18	5.12	2.02

4	*CRIM1*	IMAGp998K13587	267780	GO:0016021	transmembrane	0.00	1.20	8.46	3.23

5	*ECHS1*	IMAGp998L12154	32898	GO:0006635	fatty acid beta-oxidation	2.43	1.18	5.48	1.89

6	*HMGB1*	RZPDp201F0834D	6067961	GO:0006915	apoptosis	0.00	1.40	3.25	4.68

7	*MAP3K5*	IMAGp998O22144	28450	GO:0006915	apoptosis	0.17	1.35	6.94	2.98

8	*NGFRAP1*	IMAGp998O16794	347367	GO:0007275	multicellular organismal development	0.00	1.32	0.59	3.95

9	*NPM1*	RZPDp202B129D	3996837	GO:0006950	response to stress	0.00	1.30	2.59	3.07

10	*NUB1*	RZPDp1096A0718D	6064678	GO:0006511	ubiquitin-dependent protein catabolic process	0.64	1.12	5.87	1.62

11	*PDIA3*	RZPDp1096G0216D	5561830	GO:0006915	apoptosis	1.29	1.43	3.05	2.40

12	*PLA2G2A*	IMAGp998N13665	297804	GO:0006644	phospholipid metabolic process	0.50	1.75	4.92	3.00

13	*ROCK1*	RZPDp201C1129D	5575521	GO:0006915	apoptosis	0.17	1.17	0.00	9.76

14	*TEGT*	RZPDp1096F101D	160553	GO:0006915	apoptosis	0.00	1.32	0.73	2.77

15	*TMEM16G*	IMAGp998D184645	1895393	GO:0016021	transmembrane	0.00	0.66	4.37	0.35

16	*TMEM69*	IMAGp998I22653	293085	GO:0016021	transmembrane	3.78	1.08	4.56	2.77

17	*TRAF4*	RZPDp201H0728D	5541746	GO:0006915	apoptosis	4.29	1.26	8.58	2.59

18	*VCAN*	RZPDp1096C062D	201932	GO:0007155	cell adhesion	0.00	1.31	7.06	2.92

19	*VCP*	IMAGp998O10119	123873	GO:0006915	apoptosis	1.29	1.18	7.88	2.75

20	*VDAC1*	IMAGp998D08136	129751	GO:0008632	apoptotic program	0.00	1.51	0.30	5.41

### Immunohistochemistry demonstrates Gleason-grade associated protein expression of MAP3K5 and PDIA3

**Figure 2:**
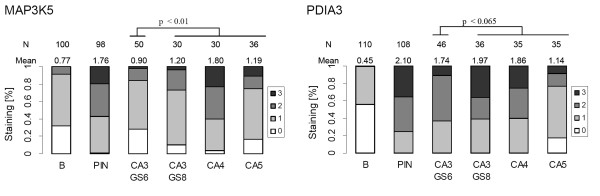
**IHC analysis of Gleason grade-associated protein expression of PDIA3 and MAP3K5**. Summary of PDIA3 and MAP3K5 protein expression quantification in tissue samples. Paraffin tissue sections were stained according to a standard IHC protocol using a staining automate and immunoreactivity of the different Gleason patterns identified in each specimen were scored by an uropathologist according to a 4 point scale (no - 0, weak - 1, intermediate - 2 and strong - 3 staining). For both antigens immunoreactivity was higher in tumors than in benign epithelium in accordance with the gene expression and real-time PCR data. Within the different tumor patterns staining intensity increased from CA3 to CA4 and decreased in the most dedifferentiated CA5 tumor regions. Interestingly, PDIA3 staining intensity in CA 3 regions within GS6 tumors (CA3 CS6) and within GS8 tumors (CA3 GS8) differed significantly, whereas this was not observed with MAP3K5. (B: Benign tissue; CA3: Gleason pattern 3, CA4: Gleason pattern 4, CA5: Gleason pattern 5).

To confirm our data at the protein level, we performed immunohistochemical analysis of the proteins MAP3K5 and PDIA3 representing the largest functional group (apoptosis) of validated genes. Specificity of antibodies was controlled using western blotting (data not shown). Protein expression levels in tumor tissue samples were scored according to a 4 point scoring system. Lowest expression levels of MAP3K5 and PDIA3 proteins were seen in benign epithelial cells (Figure 2). In agreement with the transcript analyses, MAP3K5 exhibited a significant Gleason grade-associated protein expression (p < 0.01, Wilcoxon signed rank test). Highest expression levels were observed in Gleason pattern 4 regions (mean 1.8) whereas Gleason pattern 3 (mean 1.2) and Gleason pattern 5 tumor regions (mean 1.19) displayed lower immunoreactivity. Of note, we also observed significant protein overexpression in prostate intraepithelial neoplasia (PIN) and in regions of inflammation (data not shown), which is in agreement with the described involvement of MAP3K5 with inflammation processes. Immunostaining was observed in the cytoplasm.

**Figure 3:**
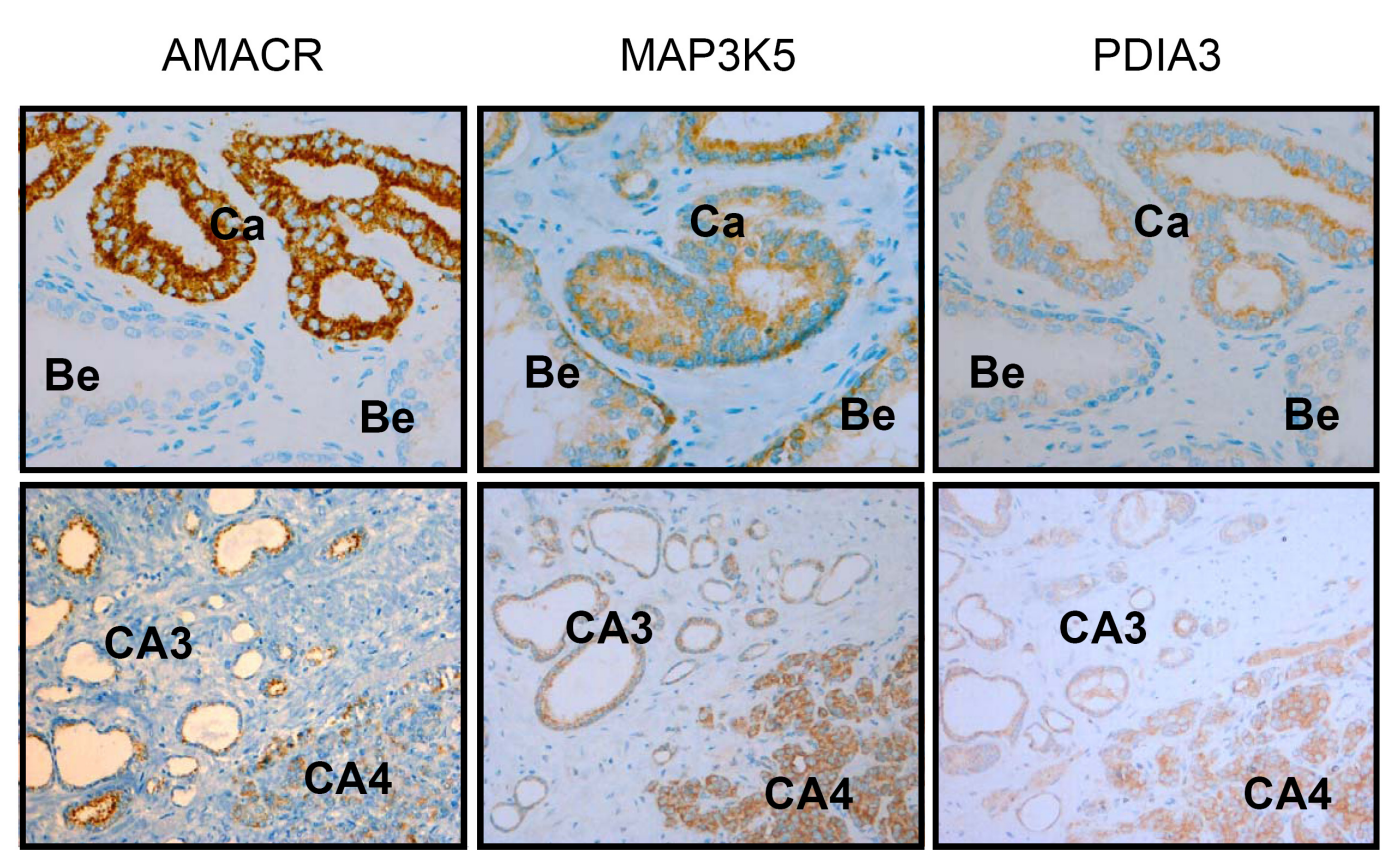
**IHC analysis for the proteins AMACR, MAP3K5 and PDIA3**. Representative IHC pictures are shown for AMACR, MAP3K5 and PDIA3. AMARC was used as a marker for confirmation of the tumor. MAP3K5 and PDIA3 IHC revealed cytoplasmatic localization of both antigens and higher expression in tumors as compared to benign epithelium. Gleason pattern CA4 displayed highest staining levels, compared with CA3 (lower midst and right pictures; magnification upper panel: 400×; lower panel: 200×).

PDIA3 also showed a grade-associated protein expression (p < 0.065, Wilcoxon signed rank test; Figure 2). Expression was significantly increased in tumor cells compared to benign epithelium. Among different Gleason patterns in the tumors, Gleason pattern 4 (GP4) showed the highest expression level. Interestingly, expression levels of PDIA3 in Gleason pattern 3 (CA3) seem to depend on the accompanying Gleason pattern in the tumor. PDIA3 expression was higher in presence of higher Gleason patterns (CA3 in GS 8 tumors, e.g. CA3 associated with CA5; mean score 1.97) than in tumors with uniform Gleason pattern 3 (CA3 in GS6 tumors; mean score 1.74). Immunostainings showed cytoplasmic and perinuclear localization of PDIA3. In advanced tumors, PDIA3 and MAP3K5 displayed a heterogeneous staining pattern and the variances of intensity distributions were higher. Representative pictures of PDIA3 and MAP3K5 in different tissue regions are shown in Figure 3, where AMACR serves as a positive control for tumor cells [15].

### Decreased apoptotic activity upon knockdown of *PDIA3 *in prostate cancer cell lines

**Figure 4:**
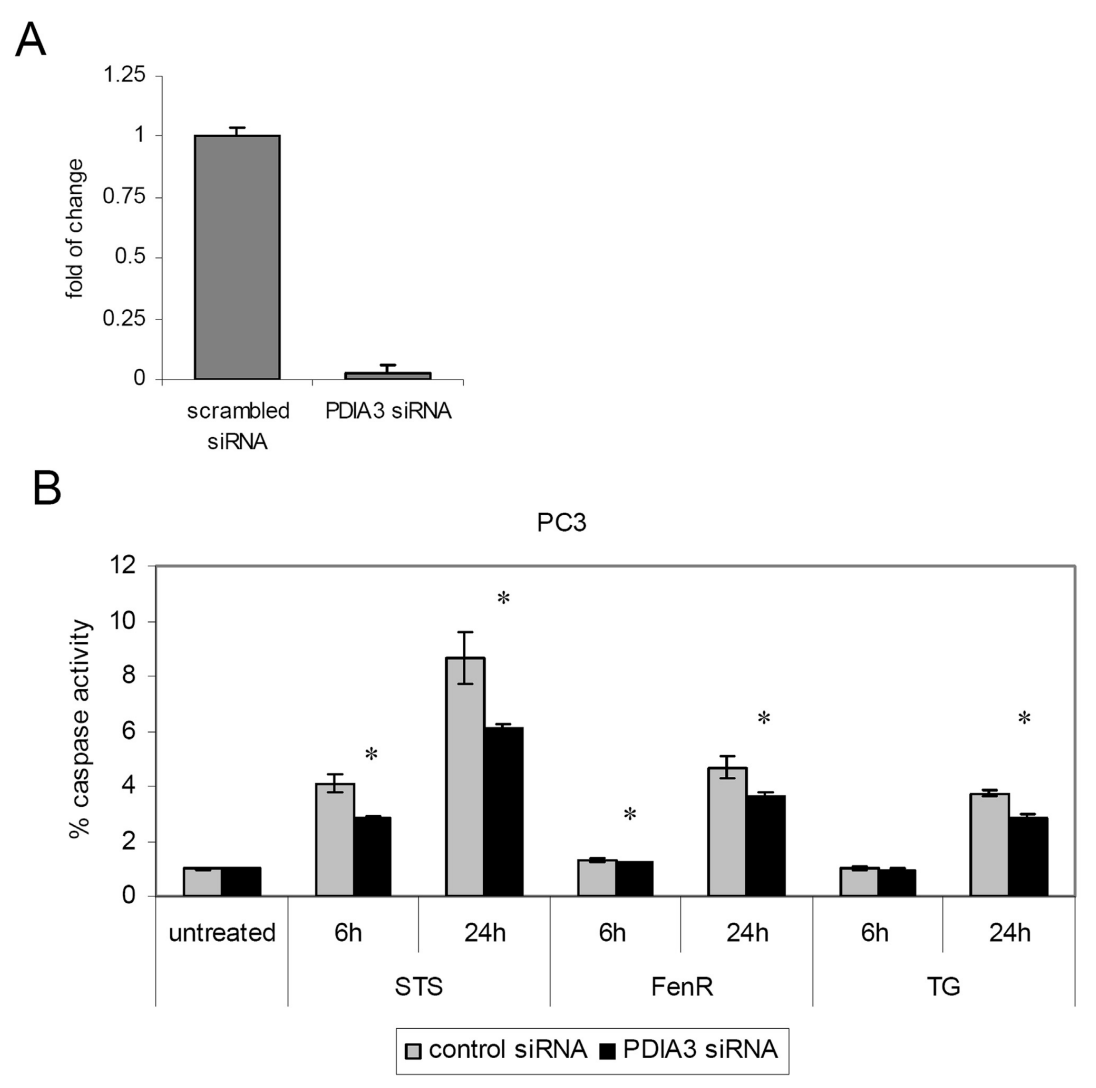
**siRNA mediated knockdown of *PDIA3 *decreased apoptosis in prostate cancer cell line**. **(A) **Knockdown efficiency measured via qRT PCR 48 h after transfection with 20 nM siRNA. **(B) **PC3 cells were treated with 20 nM scrambled siRNA control and *PDIA3 *siRNA. 48 h after transfection induction of apoptosis was performed with 1 μM Staurosporine (STS), 20 μM Fenretinide (FenR) or 1.5 μM Tapsigargin (TG) for 6 and 24 hours. Apoptosis was measured by determining caspase activation and compared to untreated control. Bar heights and error bars are means and upper range of triplicate samples relative to control treatment. * P < 0.05 (unpaired t-test).

MAP3K5 and PDIA3 were found to be proteins associated with apoptotic processes via pathway analysis. Unlike MAP3K5, whose pro-apoptotic and inflammatory role in context of tumorigenesis is well established [16,17], the function for PDIA3 in apoptosis has been largely unexplored. To investigate an apoptosis-related function of PDIA3 we performed siRNA-based knockdown in the human prostate cancer cell lines PC3 and LNCaP. 48 hours after siRNA treatment (20 nM or 40 nM) the knockdown efficiency was determined by qRT-PCR (Figure 4A). Induction of apoptosis was mediated by three different stimuli. Staurosporine (STS), Fenretinide (FenR) and Tapsigargin (TG) are known to activate apoptosis via distinct mechanisms [18-20]. Each stimulus activated the apoptotic pathway reflected by activation of caspase 3 and/or caspase 7 (Figure 4B). *PDIA3 *siRNA treatment revealed a significant decrease of caspase activation in PC3 cells with all stimuli in comparison to control siRNA treated cells. In LNCaP cells similar results were obtained, but were only significant after STS induction (data not shown). These results indicate a novel, pro-apoptotic role for *PDIA3 *in prostate cancer cells.

## Discussion

Recent studies showed that it is important to include different tumor stages of prostate cancer in gene expression analyses to be able to find new diagnostic and prognostic markers [6,8,9]. Here, we generated gene expression profiles of tissues from low-risk (GS 6) and high-risk prostate tumors (GS 8-10) tissues. In contrast to most other published studies, all tissue samples were carefully microdissected before RNA isolation. The comparison of these profiles revealed that both tumor subgroups differ by a large number of genes, most of which are up-regulated in high GS tumors. A comparison with another published data set [6] suggested that these results reflect a general trend in transcriptional activation in advanced prostate tumors. However, it cannot be fully ruled out that systematic changes introduced by different extents of stromal cells [8] in the microdissected material as well as two rounds of RNA amplification contribute to this bias.

Among the detected genes many are involved in pathways, which are known to be altered in tumor progression such as apoptosis, morphologic changes, metabolism, and ubiquitin-mediated protein degradation. Twenty representatives of these processes were verified via qRT-PCR, and a set of genes discriminating between less and more aggressive tumor forms was identified. One of the hallmarks of aggressive cancer is the imbalance between cell survival and apoptosis. Our gene ontology analysis revealed that a pronounced number of apoptosis-related genes exhibited expression changes between low- and high-risk prostate tumors. Thus, for validation, we focused our analysis on up-regulated anti-apoptotic genes and key players of apoptotic signaling and verified the expression level of 11 selected genes. These data were supported by the analysis of protein expression levels for MAP3K5 and PDIA3 by IHC. *MAP3K5 *and *PDIA3 *were also identified in other prostate cancer profiling studies [6,7,9,21]. MAP3K5 (also known as apoptosis signal-regulating kinase 1; ASK1) has been widely accepted as one of the key components regulating reactive oxygen species (ROS) - induced JNK and p38 activation leading to differentially regulated apoptosis [22]. ROS - dependent activation of MAP3K5 also plays a critical role in innate immune responses through production of proinflammatory cytokines [23]. There is considerable evidence suggesting that oxidative stress contributes to the pathogenesis of prostate cancer [24,25]. Given that mitochondria are a major source of reactive oxygen species (ROS), altered mitochondrial bioenergetics might induce *MAP3K5 *over-expression and contribute to the malignant progression of prostate tumors. In concordance with this hypothesis, we also found a significant number of deregulated genes involved in oxidative phosphorylation and mitochondrial dysfunction.

Like MAP3K5, PDIA3 (protein disulfide isomerase A3) is a member of the endoplasmatic reticulum stress signaling pathway also known as unfolded protein response (UPR), and its expression level increases in response to cellular stress due to its function as a chaperone [26]. Recently published data connected PDIA3 to the apoptotic process and demonstrated an anti-apoptotic effect of PDIA3 in the melanoma cell line A375 after induction of ER stress [27]. In contrast, our study suggested a decrease of caspase activity due to down regulation of PDIA3 in prostate cancer cell lines. This result suggests that the observed increase of *PDIA3 *in this study is most likely due to elevated cellular stress. But besides the role as a chaperone, PDIA3 might function as a pro-apoptotic protein in the prostate. Taking our IHC data of PDIA3 into account, PDIA3 protein concentration decreases significantly in CA5 compared to CA4 tissues and expression data comparing localized with metastatic prostate cancer showed a down-regulation of *PDIA3 *[9]. These findings support the idea that down regulation of *PDIA3 *might play a role in late onset of prostate cancer progression. A lack of PDIA3 expression also correlates with increased tumor invasion and advanced stage of gastric cancer and has therefore been proposed to be a negative prognostic marker [28].

In addition to its role in the ER stress pathway, PDIA3 has recently gained attention due to its function as a component of the peptide-loading complex of the major histocompatibility complex (MHC) class I pathway [29,30]. In PDIA3 deficient mice this complex is impaired and negatively influences presentation of antigenic peptides. This may help tumors to escape from immune surveillance by cytotoxic T cells [31].

The results of our IHC analysis point to a potential use of PDIA3 as a diagnostic marker: PDIA3 expression of Gleason pattern 3 tumors is higher in the presence of a Gleason pattern 5 tumor than in presence of another Gleason pattern 3 tumor. Additionally, *PDIA3 *and *MAP3K5 *have been found to be significantly (FDR 5%) up-regulated in tumors harboring a TMPRSS2 - fusion protein (data not shown). This underlines the opportunity to use PDIA3 and MAP3K5 as discriminating biomarkers in respect to histological grading system and gene arrangement classification.

In summary, this study validated a set of 20 genes, which discriminated between low and high Gleason grade prostate tumors. These genes comprise important functional processes well known to be involved in tumor progression such as apoptosis, morphological changes, metabolism and others. In addition, we show a grade-associated protein expression of MAP3K5 and PDIA3. In particular, high PDIA3 protein levels in Gleason pattern 3 cancers may indicate the presence of more aggressive tumor foci in the same tissue and could be of diagnostic value, possibly as part of a larger molecular signature.

## Materials and methods

### Tissue specimens

Frozen and paraffin-embedded prostate tissue samples were obtained from previously untreated patients who had undergone radical prostatectomy after tumor diagnosis in a PSA based screening program performed in Tyrol by the Department of Urology, Medical University of Innsbruck [32]. The study was approved by the ethics committee at the Medical University of Innsbruck. Immediately after surgery, the prostate specimens were cooled in ice/water and brought to the pathologist who performed a rapid section and isolated a prostate slice that was embedded in Tissue-Tek OCT Compound (Sakura, Tokyo, Japan), snap frozen in liquid nitrogen and stored at -80°C until use. The rest of the prostate was fixed and paraffin-embedded according to standard procedures.

### Tissue microdissection

For isolation of total RNA, frozen sections were stained with hematoxylin and eosin for pathological analysis and exact localization of the tumors. Parallel unstained slides were used for microdissection. These were pre-treated for 1 min in each of the following pre-cooled solutions: 75% ethanol, RNase-free water, 100% ethanol (twice) and xylene (twice), and air dried. Laser-capture microdissection was performed on a Pix Cell II microdissection microscope (Arcturus, Sunnyvale, CA, USA) using 2,000-5,000 laser impulses corresponding to approximately 15 000 - 30 000 cells for each sample. Tumor samples were isolated from a cohort of Gleason score 6 tumors (Gleason pattern 3) and a group of Gleason score 8 - 10 tumors (Gleason patterns 4 and 5). Benign epithelial cell samples were microdissected apart from tumor foci from histopathologically normal regions of the same specimens. After microdissection, total RNA was isolated using the PicoPure isolation kit (MDC, Sunnyvale, USA) according to the protocol of the supplier. Quality control was done employing the Agilent Bioanalyzer 2100 system (Agilent Technologies, Waldbronn, Germany).

### Microarray analysis

20 ng of RNA isolated from laser-capture microdissected epithelial prostate cells of tissues from patients who had undergone radical prostatectomy were subjected to a two-round amplification using the MessageAmpTM II aRNA Amplification Kit (Applied Biosystems/Ambion, Austin, USA). The quality of amplified RNA (aRNA) fragments was assessed by microcapillary electrophoresis using the Agilent Bioanalyzer 2100 system. Two micrograms of aRNA were subjected to microarray hybridization as described in [33]. Briefly, aRNA was reverse transcribed using SuperScript II reverse transcriptase (Invitrogen, San Diego, CA, USA) and labeled with Cy5-dUTP. Each sample was compared to a common reference (Universal Human Reference RNA; Stratagene, La Jolla, CA, USA) labeled with Cy3-dUTP. Hybridizations were done using the platform Human Unigene3.1 cDNA Array 37.5K v1.0 (NCBI, GEO, GPL3050) representing estimated 22,000 transcripts. Data were analyzed using the GenePix 4.0 software (Axon Instruments, Foster City, USA). Low quality measurements were excluded from further analysis. Raw expression values were pre-processed using ArrayMagic [34] and thereby normalized using the VSN method [35].

### Data analysis and data mining

Significance Analysis of Microarrays (SAM) was applied to identify genes differentially regulated between normal tissue, tumor tissue GS 6 and GS ≥ 8 [10]. A two class unpaired SAM test with 1000 permutations was used. The False Discovery Rate (FDR) was set below 5%. Results from the SAM analysis were imported into FatiGO [11] and Ingenuity Pathways Analysis (IPA) software (Ingenuity Systems, Redwood City, CA, USA) to identify gene ontologies that were significantly over- or under-represented. MatchMiner software [36] was used to match gene entries between different microarray studies.

### Quantitative real-time RT-PCR validation

Verification of expression of selected genes was performed via quantitative real-time RT-PCR using the ABI Prism 7900 HT Sequence Detection System (Applied Biosystems, Foster City, CA, USA) and the Universal Probe Library System (Roche, Basel, Switzerland). Ct values were extracted by using the SDS-software (Applied Biosystems). The expression level of the housekeeping gene β-2-microglobulin was used for normalization, calculated with the 2^-ΔΔCt ^method [37]. Gene expression differences between GS 6 and GS 8-10 tumors were analyzed using t-test. A list of examined genes including mean Ct values of each analyzed group and corresponding primers is given in additional file 5.

### Immunohistochemical analysis

For validation of expression at the protein level, we used immunohistochemistry (IHC) on corresponding paraffin-embedded tissue specimens from the same patient cohort. Immunohistochemistry was performed with 5 μm paraffin tissue sections employing the Ventana Discovery - XT staining automat (Roche). Standard CC1 pre-treatment and antigen retrieval was followed by incubation with antibody solution for 1 hr, choice of amplification kit, universal antibody solution for 60 min, staining with DAP map kit and counter stain for 4 min with haematoxylin II bluing reagent (all from Roche). For expression analysis of MAP3K5 (ASK1) protein the mouse monoclonal antibody EP553Y (Abcam Limited, Cambridge, MA, USA) was used at a dilution of 1:40 for the PDIA3 protein the mouse monoclonal antibody MaPERp571 (Abcam) at a dilution of 1:10. Specificity of staining was controlled by including a control antibody (DAKO Cytomation, Glostrup, Denmark). Immunoreactivity was then scored by a uropathologist and stratified according to the histology and the Gleason pattern of the specimens using a 4 point scaling system: 0, no staining, 1, weak staining, 2, intermediate staining, 3, strong staining.

### RNA interference and apoptosis assay

The human prostate cancer cell line PC3 and LNCaP were purchased from ATCC (Manassas, VA, USA) and cultured in RPMI 1640 or HAMs F12 medium, respectively. The medium was supplemented with 50 units/ml penicillin, 50 μg/ml streptomycin sulphate, 1% nonessential amino acids, and 10% FBS (all from GIBCO/BRL, Gaithersburg, MD, USA).

PDIA3 siRNA (target sequences, see additional file 6) was purchased from Dharmacon (Lafayette, CO, USA) and evaluated against a scrambled siRNA control (Qiagen, Hilden, Germany). The siRNA knockdown experiments were performed by plating 0.8 × 10^4 ^cells PC3 cells in a 96-well plate (NUNC, Roskilde, Denmark) overnight. For transfection, siRNA and Lipofectamine 2000 (Invitrogen, Carlsbad, CA, USA) were diluted separately and incubated for 5 min at room temperature. The two solutions were mixed and incubated for 20 min at room temperature. siRNA-Lipofectamine 2000 mixture was then added to the cells, and the plate was mixed by gentle rocking. Transfected cells were incubated at 37°C and 5% CO_2 _for 48 h. Knockdown efficiency was verified by qRT PCR.

Induction of apoptosis was performed by adding the indicated amounts of Staurosporine (Roche, Mannheim, Germany), Fenretinide (Sigma, Munich, Germany) or Tapsigargin (Sigma) for 6 and 24 hours, respectively. Control cells were left untreated. Activation of apoptosis was determined by measuring caspase 3 and 7 activities using Caspase-Glo 3/7^® ^Assay (Promega, Madison, WI, USA) following the manufacturer's protocol.

## Data deposition

The microarray data sets reported in this paper have been deposited MIAME compliant to NCBI Gene Expression Omnibus (GEO) database (accession no. GSE15484).

## Competing interests

The authors declare that they have no competing interests.

## Authors' contributions

NCP participated in the design and coordination of the study, contributed to assay designs and methods development, interpretation of data, and drafted the manuscript. HS and AP directed the design and coordination of the study and contributed to drafting the manuscript. HK, GS and ES identified the cancer cases, provided microdissected samples, performed immunohistochemistry and drafted the manuscript. GB participated in the design and coordination of the study. VDL carried out the microarray study, participated in the study design and methods development. RK participated in data analysis and manuscript drafting. MR performed statistical data analysis.

All authors read and approved the final manuscript.

## Supplementary Material

Additional file 1 Differentially expressed genes between GS 6 and GS 8-10 prostate cancer. A two class unpaired SAM test with 1000 permutations was performed to identify genes differentially regulated between GS 6 and GS ≥ 8 tumors. The False Discovery Rate (FDR) was set below 5%. Genes were assigned using RZPD ID, gene symbol and gene name. Click here for file

Additional file 2 Differentially expressed genes between prostate normal and cancer tissue. A two class unpaired SAM test with 1000 permutations was performed to identify genes differentially regulated between normal and cancer tissue. The False Discovery Rate (FDR) was set below 5%. Genes were assigned using RZPD ID, gene symbol and gene name. Click here for file

Additional file 3 Differentially expressed genes between tumors of high versus low Gleason score across independent datasets. Intersection of differentially expressed genes between high- and low risk prostate cancer with microarray data of True et al. 2006 [6] and Lapointe et al. 2004 [7]. Click here for file

Additional file 4 Selected genes linked to apoptosis and differentially expressed genes between tumors of high versus low Gleason score. Gene ontology analyses (FatiGO, Ingenuity, GOstat) were performed to link differentially expressed genes between high versus low Gleason score to the gene ontology apoptosis (GO:0006915, GO:0008219). Statistical data from the microarray experiment were included. Click here for file

Additional file 5 Genes analyzed by quantitative real-time PCR. In total, 68 genes were analyzed by qRT-PCR in order to validate expression differences between tumors of high (GS8-10) and low (GS6) Gleason scores. p-values from the statistical analysis are shown. In addition, fold change, mean Ct values of both tumor groups and the primer sequences of the gene-specific assays are indicated. Click here for file

Additional file 6 Sequences of siRNAs (Dharmacon smartPool On-Target plus) used for knockdown of PDIA3. Click here for file
