# Supplementary material for: Differential expression of apoptotic genes PDIA3 and MAP3K5 distinguishes between low- and high-risk prostate cancer
Source: Mol Cancer. 2009 Dec 27;8:130. doi: 10.1186/1476-4598-8-130 (PMC2807430; doi:10.1186/1476-4598-8-130)
Supplement: Additional file 1 — Differentially expressed genes between GS 6 and GS 8-10 prostate cancer. A two class unpaired SAM test with 1000 permutations was performed to identify genes differentially regulated between GS 6 and GS ≥ 8 tumors. The False Discovery Rate (FDR) was set below 5%. Genes were assigned using RZPD ID, gene symbol and gene name. [file 1476-4598-8-130-S1.PDF]

Additional File 1: Differentially expressed genes between prostate cancer GS 6 and GS 8-10

No.	RZPD ID	q-value(%)	Fold Change	Gene Symbol	Name
1	IMAGp998D08136	0.00	1.51	*VDAC1*	Voltage-dependent anion channel 1
2	RZPDp201H0327D	0.00	1.72	*HSD17B4*	Hydroxysteroid (17-beta) dehydrogenase 4
3	IMAGp998C21189	0.00	1.32	*STIM2*	Stromal interaction molecule 2
4	IMAGp998P24664	0.00	1.38	*UQCRH*	Ubiquinol-cytochrome c reductase hinge protein
5	IMAGp998H03152	0.00	1.31	*PTDSS1*	Phosphatidylserine synthase 1
6	IMAGp998M13132	0.00	1.49	*YWHAZ*	Tyrosine 3-monooxygenase/tryptophan 5-monooxygenase activation protein, zeta polypeptide
7	RZPDp202B129D	0.00	1.30	*NPM1*	Nucleophosmin (nucleolar phosphoprotein B23, numatrin)
8	IMAGp998B11692	0.00	1.39	*TBCA*	Tubulin folding cofactor A
9	IMAGp998G02590	0.00	1.34	*CTTNBP2NL*	CTTNBP2 N-terminal like
10	IMAGp998D12182	0.00	1.55	*ATP6V1A*	ATPase, H+ transporting, lysosomal 70kDa, V1 subunit A
11	IMAGp998G12153	0.00	1.46	*PFN2*	Profilin 2
12	RZPDp1096H1014D	0.00	1.31	*EIF4B*	Eukaryotic translation initiation factor 4B
13	RZPDp201B0334D	0.00	1.44	*VDAC1*	Voltage-dependent anion channel 1
14	IMAGp998A10145	0.00	1.39	*PAK4*	P21(CDKN1A)-activated kinase 4
15	RZPDp201C0720D	0.00	1.54	*ERRFI1*	ERBB receptor feedback inhibitor 1
16	IMAGp998P183582	0.00	1.33	*N/A*	Transcribed locus
17	IMAGp998P10840	0.00	1.67	*CCDC80*	Coiled-coil domain containing 80
18	RZPDp1096A0420D	0.00	1.30	*UBE2L6*	Ubiquitin-conjugating enzyme E2L 6
19	IMAGp998D02200	0.00	1.38	*ATP13A3*	ATPase type 13A3
20	IMAGp998E17174	0.00	1.18	*IQGAP1*	IQ motif containing GTPase activating protein 1
21	RZPDp1096D0213D	0.00	1.34	*HMGN2*	High-mobility group nucleosomal binding domain 2
22	IMAGp998A132000	0.00	1.28	*SNHG1*	Small nucleolar RNA host gene (non-protein coding) 1
23	IMAGp998H16268	0.00	1.32	*DNAJA1*	DnaJ (Hsp40) homolog, subfamily A, member 1
24	IMAGp998A06154	0.00	1.34	*NAP1L1*	Nucleosome assembly protein 1-like 1
25	IMAGp998B18613	0.00	1.29	*GNAI3*	Guanine nucleotide binding protein (G protein), alpha inhibiting activity polypeptide 3
26	IMAGp998G19656	0.00	1.37	*ADAR*	Adenosine deaminase, RNA-specific
27	IMAGp998M191167	0.00	1.34	*TRIAP1*	TP53 regulated inhibitor of apoptosis 1
28	IMAGp998D20286	0.00	1.46	*HADHB*	Hydroxyacyl-Coenzyme A dehydrogenase/3-ketoacyl-Coenzyme A thiolase/enoyl-Coenzyme A hydratase (trifunctional protein), beta subunit
29	IMAGp998J165190	0.00	1.28	*N/A*	Transcribed locus
30	IMAGp998B22375	0.00	1.27	*CDC42*	Cell division cycle 42 (GTP binding protein, 25kDa)
31	IMAGp998B01363	0.00	1.42	*BUB3*	BUB3 budding uninhibited by benzimidazoles 3 homolog (yeast)
32	RZPDp202F063D	0.00	1.21	*SNN*	Stannin
33	RZPDp201A0330D	0.00	1.32	*C1orf43*	Chromosome 1 open reading frame 43
34	RZPDp202E076D	0.00	1.15	*ODF2*	Outer dense fiber of sperm tails 2
35	IMAGp998H031824	0.00	2.79	*N/A*	Transcribed locus
36	RZPDp201F0519D	0.00	1.49	*SCP2*	Sterol carrier protein 2
37	IMAGp998C21738	0.00	1.45	*ACTG1*	Actin, gamma 1
38	IMAGp998C23825	0.00	1.18	*LMNA*	Lamin A/C
39	IMAGp998M15655	0.00	1.44	*HNRPC*	Heterogeneous nuclear ribonucleoprotein C (C1/C2)
40	RZPDp201E0129D	0.00	1.57	*LOC151579*	Similar to basic leucine zipper and W2 domains 1
41	IMAGp998D165687	0.00	1.28	*N/A*	CDNA FLJ42664 fis, clone BRAMY2019985
42	IMAGp998A06525	0.00	1.52	*ERRFI1*	ERBB receptor feedback inhibitor 1
43	RZPDp201C0234D	0.00	1.40	*RRM2B*	Ribonucleotide reductase M2 B (TP53 inducible)
44	IMAGp998G22650	0.00	1.37	*ACTR3*	ARP3 actin-related protein 3 homolog (yeast)
45	IMAGp998K21387	0.00	1.42	*CSDE1*	Cold shock domain containing E1, RNA-binding
46	RZPDp202C104D	0.00	1.31	*LBH*	Limb bud and heart development homolog (mouse)
47	IMAGp998C06214	0.00	1.31	*KIDINS220*	Kinase D-interacting substance of 220 kDa
48	IMAGp998C01311	0.00	1.36	*CCNG2*	Cyclin G2
49	IMAGp998L10144	0.00	1.26	*SUMO4*	SMT3 suppressor of mif two 3 homolog 4 (S. cerevisiae)
50	IMAGp998C01528	0.00	1.22	*N/A*	Transcribed locus
51	IMAGp998N20159	0.00	1.34	*XRN1*	5'-3' exoribonuclease 1
52	IMAGp998E011822	0.00	1.28	*LRFN1*	Leucine rich repeat and fibronectin type III domain containing 1
53	IMAGp998A061862	0.00	1.44	*EIF4A2*	Eukaryotic translation initiation factor 4A, isoform 2
54	IMAGp998N11530	0.00	1.26	*FBXO10*	F-box protein 10
55	RZPDp1096C073D	0.00	1.38	*LRBA*	LPS-responsive vesicle trafficking, beach and anchor containing
56	IMAGp998O041170	0.00	1.39	*PNRC2*	Proline-rich nuclear receptor coactivator 2
57	IMAGp998G1518	0.00	1.47	*ANXA5*	Annexin A5
58	IMAGp998C15159	0.00	1.28	*RRAGA*	Ras-related GTP binding A
59	IMAGp998L18825	0.00	1.23	*NDUFB3*	NADH dehydrogenase (ubiquinone) 1 beta subcomplex, 3, 12kDa
60	RZPDp201H0119D	0.00	1.32	*CSNK1A1*	Casein kinase 1, alpha 1
61	RZPDp1096E1216D	0.00	1.33	*HIST1H2BK*	Histone cluster 1, H2bk
62	RZPDp201H1132D	0.00	1.25	*CAB39*	Calcium binding protein 39
63	RZPDp201F0516D	0.00	1.34	*ZC3H3*	Zinc finger CCCH-type containing 3
64	IMAGp998N19122	0.00	1.25	*NDUFS2*	NADH dehydrogenase (ubiquinone) Fe-S protein 2, 49kDa (NADH-coenzyme Q reductase)
65	IMAGp998E23140	0.00	1.30	*MARCKS*	Myristoylated alanine-rich protein kinase C substrate
66	RZPDp1096A0216D	0.00	1.29	*FYTTD1*	Forty-two-three domain containing 1
67	IMAGp998A081943	0.00	1.23	*TOB1*	Transducer of ERBB2, 1
68	IMAGp998A15692	0.00	1.22	*TOP2B*	Topoisomerase (DNA) II beta 180kDa
69	IMAGp998N09597	0.00	1.43	*EIF4G2*	Eukaryotic translation initiation factor 4 gamma, 2
70	IMAGp998C16178	0.00	1.48	*SNX3*	Sorting nexin 3
71	IMAGp998H07420	0.00	1.35	*PSMC2*	Proteasome (prosome, macropain) 26S subunit, ATPase, 2
72	IMAGp998I04122	0.00	1.25	*RAC1*	Ras-related C3 botulinum toxin substrate 1 (rho family, small GTP binding protein Rac1)
73	IMAGp998P05174	0.00	1.32	*NCOA4*	Nuclear receptor coactivator 4
74	IMAGp998O11158	0.00	1.30	*ATP5F1*	ATP synthase, H+ transporting, mitochondrial F0 complex, subunit B1
75	RZPDp1096D076D	0.00	1.39	*ARL6IP1*	ADP-ribosylation factor-like 6 interacting protein 1
76	IMAGp998D051998	0.00	1.20	*SEC11A*	SEC11 homolog A (S. cerevisiae)
77	IMAGp998N16280	0.00	1.20	*SPIN1*	Spindlin 1
78	IMAGp998O17578	0.00	1.36	*H3F3B*	H3 histone, family 3B (H3.3B)
79	RZPDp202D017D	0.00	1.18	*GPD1L*	Glycerol-3-phosphate dehydrogenase 1-like
80	IMAGp998K05583	0.00	1.30	*SFRS10*	Splicing factor, arginine/serine-rich 10 (transformer 2 homolog, Drosophila)
81	IMAGp998I201865	0.00	1.15	*OSBPL8*	Oxysterol binding protein-like 8
82	IMAGp998G04114	0.00	1.21	*HSPC111*	Hypothetical protein HSPC111
83	IMAGp998F10422	0.00	1.22	*MRPL3*	Mitochondrial ribosomal protein L3
84	IMAGp998J071118	0.00	1.27	*EIF3S3*	Eukaryotic translation initiation factor 3, subunit 3 gamma, 40kDa
85	IMAGp998L061167	0.00	1.29	*SNX4*	Sorting nexin 4
86	IMAGp998H21591	0.00	1.14	*SNRPB2*	Small nuclear ribonucleoprotein polypeptide B''
87	RZPDp202F106D	0.00	1.23	*SHFM1*	Split hand/foot malformation (ectrodactyly) type 1
88	IMAGp998N041725	0.00	1.33	*YWHAQ*	Tyrosine 3-monooxygenase/tryptophan 5-monooxygenase activation protein, theta polypeptide
89	IMAGp998K21143	0.00	1.27	*LASP1*	LIM and SH3 protein 1
90	IMAGp998E05223	0.00	1.15	*USP9X*	Ubiquitin specific peptidase 9, X-linked
91	IMAGp998B1472	0.00	1.42	*GNAS*	GNAS complex locus
92	IMAGp998K081157	0.00	1.35	*RNF19*	Ring finger protein 19
93	IMAGp998L06144	0.00	1.26	*N/A*	In multiple clusters
94	IMAGp998F01215	0.00	1.45	*BZW1*	Basic leucine zipper and W2 domains 1
95	IMAGp998P02206	0.00	1.21	*LGR4*	Leucine-rich repeat-containing G protein-coupled receptor 4
96	RZPDp1096D096D	0.00	1.41	*GNAS*	GNAS complex locus
97	IMAGp998B20319	0.00	1.23	*C1orf128*	Chromosome 1 open reading frame 128
98	RZPDp201E1211D	0.00	1.25	*PPIA*	Peptidylprolyl isomerase A (cyclophilin A)
99	IMAGp998P231160	0.00	1.26	*N/A*	Shwachman-Bodian-Diamond syndrome pseudogene, mRNA (cDNA clone IMAGE:4329436)
100	IMAGp998E10541	0.00	1.26	*NR3C1*	Nuclear receptor subfamily 3, group C, member 1 (glucocorticoid receptor)
101	IMAGp998G021851	0.00	1.21	*GBAS*	Glioblastoma amplified sequence
102	IMAGp998M021787	0.00	1.29	*LOC387826*	Hypothetical LOC387826
103	IMAGp998O16794	0.00	1.32	*NGFRAP1*	Nerve growth factor receptor (TNFRSF16) associated protein 1
104	RZPDp201A0533D	0.00	1.25	*MON2*	MON2 homolog (S. cerevisiae)
105	IMAGp998H01617	0.00	1.15	*PPP1CC*	Protein phosphatase 1, catalytic subunit, gamma isoform
106	IMAGp998B011853	0.00	1.31	*NDUFC2*	NADH dehydrogenase (ubiquinone) 1, subcomplex unknown, 2, 14.5kDa
107	RZPDp1096B061D	0.00	1.15	*MAPKAPK5*	Mitogen-activated protein kinase-activated protein kinase 5
108	IMAGp998A15281	0.00	1.25	*ALDH7A1*	Aldehyde dehydrogenase 7 family, member A1
109	IMAGp998P111198	0.00	1.30	*WWP1*	WW domain containing E3 ubiquitin protein ligase 1
110	IMAGp998K22655	0.00	1.16	*UBFD1*	Ubiquitin family domain containing 1
111	RZPDp202B088D	0.00	1.68	*MFAP1*	Microfibrillar-associated protein 1
112	IMAGp998A04844	0.00	1.23	*UBFD1*	Ubiquitin family domain containing 1
113	IMAGp998F1684	0.00	1.24	*DLD*	Dihydrolipoamide dehydrogenase
114	RZPDp201D0129D	0.00	1.20	*KIAA0738*	KIAA0738 gene product
115	IMAGp998F024495	0.00	1.80	*N/A*	Transcribed locus
116	RZPDp1096D091D	0.00	1.19	*ZFR*	Zinc finger RNA binding protein
117	IMAGp998C23979	0.00	1.25	*U2AF1*	U2 small nuclear RNA auxiliary factor 1
118	IMAGp998G17273	0.00	1.42	*N/A*	Transcribed locus
119	IMAGp998A08613	0.00	2.48	*BCCIP*	BRCA2 and CDKN1A interacting protein
120	IMAGp998M15110	0.00	1.18	*GPD1L*	Glycerol-3-phosphate dehydrogenase 1-like
121	IMAGp998E12266	0.00	1.12	*UBE2D2*	Ubiquitin-conjugating enzyme E2D 2 (UBC4/5 homolog, yeast)
122	IMAGp998P09174	0.00	1.27	*TARDBP*	TAR DNA binding protein
123	IMAGp998P06328	0.00	1.17	*SRXN1*	Sulfiredoxin 1 homolog (S. cerevisiae)
124	IMAGp998B112035	0.00	1.19	*C18orf55*	Chromosome 18 open reading frame 55
125	IMAGp998J06157	0.00	1.18	*ITGB1BP1*	Integrin beta 1 binding protein 1
126	RZPDp201F0834D	0.00	1.40	*HMGB1*	High-mobility group box 1
127	IMAGp998O17195	0.00	1.18	*ZFR*	Zinc finger RNA binding protein
128	IMAGp998B16580	0.00	1.17	*FAM96A*	Family with sequence similarity 96, member A
129	RZPDp1096F067D	0.00	1.37	*EIF3S6*	Eukaryotic translation initiation factor 3, subunit 6 48kDa
130	RZPDp1096A088D	0.00	1.35	*ALDH7A1*	Aldehyde dehydrogenase 7 family, member A1
131	RZPDp1096H031D	0.00	1.18	*MKI67IP*	MKI67 (FHA domain) interacting nucleolar phosphoprotein
132	RZPDp1096C062D	0.00	1.31	*VCAN*	Versican
133	RZPDp202E0710D	0.00	1.16	*UQCRH*	Ubiquinol-cytochrome c reductase hinge protein
134	IMAGp998A06282	0.00	1.85	*CTDSP2*	CTD (carboxy-terminal domain, RNA polymerase II, polypeptide A) small phosphatase 2
135	IMAGp998D19605	0.00	1.36	*LRBA*	LPS-responsive vesicle trafficking, beach and anchor containing

136	IMAGp998C21389	0.00	1.27	*VCAN*	Versican
137	RZPDp202B058D	0.00	1.19	*DDB1*	Damage-specific DNA binding protein 1, 127kDa
138	RZPDp202D085D	0.00	1.23	*STK11*	Serine/threonine kinase 11
139	IMAGp998J13540	0.00	1.36	*SORT1*	Sortilin 1
140	IMAGp998B011161	0.00	1.51	*Gcom1*	GRINL1A combined protein
141	RZPDp201H1231D	0.00	1.19	*C20orf30*	Chromosome 20 open reading frame 30
142	IMAGp998P011821	0.00	1.27	*EAF1*	ELL associated factor 1
143	IMAGp998N16175	0.00	1.22	*GRIP2*	Glutamate receptor interacting protein 2
144	IMAGp998O19979	0.00	1.21	*N/A*	Transcribed locus
145	IMAGp998C151790	0.00	1.20	*SEPT2*	Septin 2
146	IMAGp998L22661	0.00	1.33	*N/A*	CDNA: FLJ22522 fis, clone HRC12491
147	IMAGp998K03526	0.00	1.22	*APH1A*	Anterior pharynx defective 1 homolog A (C. elegans)
148	IMAGp998G181785	0.00	1.21	*THRAP4*	Thyroid hormone receptor associated protein 4
149	IMAGp998F24110	0.00	1.21	*EIF5*	Eukaryotic translation initiation factor 5
150	IMAGp998M10592	0.00	1.18	*DCBLD2*	Discoidin, CUB and LCCL domain containing 2
151	IMAGp998I011118	0.00	1.58	*ZFAND6*	Zinc finger, AN1-type domain 6
152	IMAGp998O112010	0.00	1.41	*TPD52*	Tumor protein D52
153	IMAGp998K16440	0.00	1.20	*RNF12*	Ring finger protein 12
154	RZPDp1096H115D	0.00	1.21	*PSMB2*	Proteasome (prosome, macropain) subunit, beta type, 2
155	IMAGp998D173955	0.00	1.41	*N/A*	Transcribed locus
156	IMAGp998O131116	0.00	1.25	*PPIC*	Peptidylprolyl isomerase C (cyclophilin C)
157	IMAGp998A02177	0.00	1.17	*ADNP*	Activity-dependent neuroprotector
158	RZPDp202A012D	0.00	1.23	*ARHGAP22*	Rho GTPase activating protein 22
159	IMAGp998B11244	0.00	1.15	*CCDC90B*	Coiled-coil domain containing 90B
160	IMAGp998I221937	0.00	1.24	*NSUN2*	NOL1/NOP2/Sun domain family, member 2
161	IMAGp998L14278	0.00	1.24	*FKBP3*	FK506 binding protein 3, 25kDa
162	IMAGp998P24142	0.00	1.26	*TSPAN13*	Tetraspanin 13
163	IMAGp998F121203	0.00	1.16	*HMGN2*	High-mobility group nucleosomal binding domain 2
164	IMAGp998E09596	0.00	1.15	*ZFP91*	Zinc finger protein 91 homolog (mouse)
165	IMAGp998K13587	0.00	1.20	*CRIM1*	Cysteine rich transmembrane BMP regulator 1 (chordin-like)
166	IMAGp998M22367	0.00	1.30	*RHOA*	Ras homolog gene family, member A
167	IMAGp998I031832	0.00	2.16	*N/A*	Transcribed locus
168	IMAGp998N14139	0.00	1.23	*N/A*	CDNA clone IMAGE:5262496
169	RZPDp201A062D	0.00	1.24	*TUBA4B*	Tubulin, alpha 4b
170	IMAGp998H19228	0.00	1.20	*SMAD5*	SMAD family member 5
171	RZPDp201C0134D	0.00	1.60	*C1orf21*	Chromosome 1 open reading frame 21
172	IMAGp998H031906	0.00	1.22	*DEK*	DEK oncogene (DNA binding)
173	RZPDp1096A111D	0.00	1.19	*SHOC2*	Soc-2 suppressor of clear homolog (C. elegans)
174	IMAGp998H16144	0.00	1.26	*FKBP4*	FK506 binding protein 4, 59kDa
175	IMAGp998G06140	0.00	1.13	*HUWE1*	HECT, UBA and WWE domain containing 1
176	IMAGp998D04631	0.00	1.40	*ATP11A*	ATPase, Class VI, type 11A
177	IMAGp998F041944	0.00	1.15	*MED10*	Mediator of RNA polymerase II transcription, subunit 10 homolog (NUT2, S. cerevisiae)
178	RZPDp202F052D	0.00	1.16	*UTP14A*	UTP14, U3 small nucleolar ribonucleoprotein, homolog A (yeast)
179	IMAGp998A24202	0.00	1.27	*ENOPH1*	Enolase-phosphatase 1
180	IMAGp998F014642	0.00	1.19	*N/A*	CDNA FLJ12909 fis, clone NT2RP2004400
181	IMAGp998O0186	0.00	1.35	*COX6C*	Cytochrome c oxidase subunit VIc
182	RZPDp201D1230D	0.00	1.29	*CCT8*	Chaperonin containing TCP1, subunit 8 (theta)
183	IMAGp998L06462	0.00	1.34	*RSRC2*	Arginine/serine-rich coiled-coil 2
184	IMAGp998F10663	0.00	1.37	*MRCL3*	Myosin regulatory light chain MRCL3
185	IMAGp998G101792	0.00	1.14	*CHMP5*	Chromatin modifying protein 5
186	IMAGp998L154205	0.00	1.14	*N/A*	Data not found
187	IMAGp998N16142	0.00	1.24	*PCBP1*	Poly(rC) binding protein 1
188	IMAGp998O13794	0.00	1.20	*N/A*	Transcribed locus, moderately similar to XP_513998.2 dynamin 3 [Pan troglodytes]
189	RZPDp1096F101D	0.00	1.32	*TEGT*	Testis enhanced gene transcript (BAX inhibitor 1)
190	IMAGp998M22199	0.00	1.35	*TCF21*	Transcription factor 21
191	IMAGp998A22148	0.00	1.17	*ABCG1*	ATP-binding cassette, sub-family G (WHITE), member 1
192	IMAGp998O18545	0.00	1.20	*UBLCP1*	Ubiquitin-like domain containing CTD phosphatase 1
193	IMAGp998H12275	0.00	1.14	*UBE2N*	Ubiquitin-conjugating enzyme E2N (UBC13 homolog, yeast)
194	RZPDp201B0234D	0.00	1.45	*NEDD4L*	Neural precursor cell expressed, developmentally down-regulated 4-like
195	IMAGp998N08175	0.00	1.18	*PTMA*	Prothymosin, alpha (gene sequence 28)
196	IMAGp998C11138	0.00	1.19	*RETSAT*	Retinol saturase (all-trans-retinol 13,14-reductase)
197	IMAGp998G09524	0.00	1.17	*TUBA1B*	Tubulin, alpha 1b
198	IMAGp998D19269	0.00	1.19	*RBBP7*	Retinoblastoma binding protein 7
199	IMAGp998H12204	0.00	1.48	*UGDH*	UDP-glucose dehydrogenase
200	IMAGp998O2092	0.00	1.22	*SAE2*	SUMO1 activating enzyme subunit 2
201	IMAGp998H12142	0.00	1.16	*MAL2*	Mal, T-cell differentiation protein 2
202	IMAGp998K01373	0.00	1.31	*MYCBP2*	MYC binding protein 2
203	IMAGp998E091200	0.00	1.13	*NCBP2*	Nuclear cap binding protein subunit 2, 20kDa
204	IMAGp998N13464	0.00	1.19	*E2F3*	E2F transcription factor 3
205	IMAGp998A061931	0.00	1.13	*GNL2*	Guanine nucleotide binding protein-like 2 (nucleolar)
206	IMAGp998J03871	0.00	1.24	*N/A*	Homo sapiens, clone IMAGE:5533883, mRNA
207	IMAGp998D13403	0.00	1.28	*C2orf25*	Chromosome 2 open reading frame 25
208	IMAGp998A03183	0.00	1.32	*N/A*	Transcribed locus
209	IMAGp998D04657	0.00	1.24	*EIF4E2*	Eukaryotic translation initiation factor 4E family member 2
210	IMAGp998B24976	0.00	1.23	*SFRS2B*	Splicing factor, arginine/serine-rich 2B
211	RZPDp202G086D	0.00	1.14	*LAD1*	Ladinin 1
212	RZPDp201B0635D	0.00	1.16	*ACAD9*	Acyl-Coenzyme A dehydrogenase family, member 9
213	IMAGp998K20778	0.00	1.59	*LOC728554*	Similar to THO complex 3
214	IMAGp998B12233	0.00	1.15	*KIAA1219*	KIAA1219
215	RZPDp201F0528D	0.00	1.22	*SHC1*	SHC (Src homology 2 domain containing) transforming protein 1
216	IMAGp998B133627	0.00	1.16	*N/A*	Transcribed locus
217	RZPDp1096A1220D	0.00	1.30	*CSDE1*	Cold shock domain containing E1, RNA-binding
218	IMAGp998B231870	0.00	1.17	*BLOC1S2*	Biogenesis of lysosome-related organelles complex-1, subunit 2
219	IMAGp998I211855	0.00	1.20	*C11orf58*	Chromosome 11 open reading frame 58
220	IMAGp998I13537	0.00	1.34	*APEX1*	APEX nuclease (multifunctional DNA repair enzyme) 1
221	IMAGp998B121791	0.00	1.13	*EIF4A3*	Eukaryotic translation initiation factor 4A, isoform 3
222	IMAGp998B17283	0.00	1.20	*DKFZP564J0863*	DKFZP564J0863 protein
223	IMAGp998B19603	0.00	1.16	*EIF4B*	Eukaryotic translation initiation factor 4B
224	IMAGp998D13282	0.00	1.13	*PTP4A2*	Protein tyrosine phosphatase type IVA, member 2
225	IMAGp998K04139	0.00	1.42	*SPARC*	Secreted protein, acidic, cysteine-rich (osteonectin)
226	RZPDp202F028D	0.00	1.14	*DDX1*	DEAD (Asp-Glu-Ala-Asp) box polypeptide 1
227	IMAGp998C01149	0.00	1.36	*SPRED2*	Sprouty-related, EVH1 domain containing 2
228	IMAGp998K23612	0.00	1.13	*ELP4*	Elongation protein 4 homolog (S. cerevisiae)
229	IMAGp998M191779	0.00	1.23	*C20orf72*	Chromosome 20 open reading frame 72
230	IMAGp998D08280	0.00	1.53	*SPTLC2*	Serine palmitoyltransferase, long chain base subunit 2
231	RZPDp201D0135D	0.00	1.27	*TRIAP1*	TP53 regulated inhibitor of apoptosis 1
232	RZPDp201B1229D	0.00	1.18	*CAPZA2*	Capping protein (actin filament) muscle Z-line, alpha 2
233	IMAGp998I10536	0.00	1.28	*PARP9*	Poly (ADP-ribose) polymerase family, member 9
234	RZPDp202D102D	0.00	1.17	*LARP1*	La ribonucleoprotein domain family, member 1
235	IMAGp998J19172	0.00	1.19	*LFNG*	LFNG O-fucosylpeptide 3-beta-N-acetylglucosaminyltransferase
236	RZPDp1096B0120D	0.00	1.18	*EI24*	Etoposide induced 2.4 mRNA
237	IMAGp998J13733	0.00	1.35	*N/A*	Full length insert cDNA clone ZC30C07
238	IMAGp998G021792	0.00	1.29	*FTMT*	Ferritin mitochondrial
239	IMAGp998M121887	0.00	1.22	*PITPNB*	Phosphatidylinositol transfer protein, beta
240	RZPDp201B1036D	0.00	1.32	*ACTR2*	ARP2 actin-related protein 2 homolog (yeast)
241	IMAGp998P01118	0.00	1.50	*ACTR2*	ARP2 actin-related protein 2 homolog (yeast)
242	IMAGp998O1572	0.00	1.18	*DKFZP686E2158*	Hypothetical protein LOC643155
243	IMAGp998L031206	0.00	1.19	*AK3*	Adenylate kinase 3
244	RZPDp201D1232D	0.00	1.29	*DNM3*	Dynamin 3
245	IMAGp998N213511	0.00	1.23	*MGC20983*	Hypothetical protein MGC20983
246	IMAGp998J2273	0.00	1.13	*ARL6IP1*	ADP-ribosylation factor-like 6 interacting protein 1
247	RZPDp1096G0514D	0.00	1.41	*SH3BP2*	SH3-domain binding protein 2
248	RZPDp202D0710D	0.00	1.12	*MRPS33*	Mitochondrial ribosomal protein S33
249	IMAGp998J021165	0.00	1.29	*PDE7B*	Phosphodiesterase 7B
250	RZPDp201D042D	0.00	1.18	*MORF4L1*	Mortality factor 4 like 1
251	RZPDp1096D0617D	0.00	1.09	*HOMER1*	Homer homolog 1 (Drosophila)
252	RZPDp201C0715D	0.00	1.19	*PZP*	Pregnancy-zone protein
253	IMAGp998K12781	0.00	1.20	*RAB23*	RAB23, member RAS oncogene family
254	IMAGp998D16269	0.00	1.38	*SURF5*	Surfeit 5
255	IMAGp998A01140	0.00	1.23	*TCEA1*	Transcription elongation factor A (SII), 1
256	IMAGp998I185323	0.00	1.26	*SRP54*	Signal recognition particle 54kDa
257	IMAGp998L081748	0.00	1.34	*IFITM3*	Interferon induced transmembrane protein 3 (1-8U)
258	RZPDp201A0830D	0.17	1.14	*SEPT7*	Septin 7
259	IMAGp998O14371	0.17	1.18	*ITSN1*	Intersectin 1 (SH3 domain protein)
260	IMAGp998B242982	0.17	1.12	*PTPRA*	Protein tyrosine phosphatase, receptor type, A
261	IMAGp998F04258	0.17	1.19	*N/A*	CDNA FLJ43454 fis, clone OCBBF2034906
262	RZPDp1096G107D	0.17	1.22	*REEP5*	Receptor accessory protein 5
263	IMAGp998F20169	0.17	1.39	*RPL15*	Ribosomal protein L15
264	IMAGp998F10191	0.17	1.22	*PERP*	PERP, TP53 apoptosis effector
265	IMAGp998L11138	0.17	1.30	*EIF3S6*	Eukaryotic translation initiation factor 3, subunit 6 48kDa
266	IMAGp998A09411	0.17	1.20	*HNRPDL*	Heterogeneous nuclear ribonucleoprotein D-like
267	IMAGp998H08377	0.17	1.22	*LOC401152*	HCV F-transactivated protein 1
268	IMAGp998N07174	0.17	1.19	*MORF4L2*	Mortality factor 4 like 2
269	IMAGp998N142001	0.17	1.21	*PRSS23*	Protease, serine, 23
270	IMAGp998P10268	0.17	1.15	*SCYE1*	Small inducible cytokine subfamily E, member 1 (endothelial monocyte-activating)

271	IMAGp998O16872	0.17	1.18	*DCUN1D5*	DCN1, defective in cullin neddylation 1, domain containing 5 (S. cerevisiae)
272	RZPDp202G045D	0.17	1.27	*RGS10*	Regulator of G-protein signalling 10
273	IMAGp998B23224	0.17	1.20	*SOS2*	Son of sevenless homolog 2 (Drosophila)
274	IMAGp998B081898	0.17	1.22	*C1orf149*	Chromosome 1 open reading frame 149
275	IMAGp998J24200	0.17	1.21	*N/A*	CDNA clone IMAGE:3897094
276	IMAGp998H05155	0.17	1.13	*MRPS35*	Mitochondrial ribosomal protein S35
277	IMAGp998G09378	0.17	1.31	*KIAA0251*	KIAA0251 protein
278	RZPDp201H1020D	0.17	1.22	*LOC283398*	Similar to Succinyl-CoA ligase [GDP-forming] beta-chain, mitochondrial precursor
279	IMAGp998H1687	0.17	1.19	*ZCCHC9*	Zinc finger, CCHC domain containing 9
280	IMAGp998I18591	0.17	1.14	*N/A*	CDNA FLJ33024 fis, clone THYMU1000532
281	IMAGp998K151159	0.17	2.00	*LTV1*	LTV1 homolog (S. cerevisiae)
282	IMAGp998C15391	0.17	1.26	*DDX5*	DEAD (Asp-Glu-Ala-Asp) box polypeptide 5
283	IMAGp998H09170	0.17	1.22	*CYCS*	Cytochrome c, somatic
284	IMAGp998N09625	0.17	1.25	*ARF4*	ADP-ribosylation factor 4
285	IMAGp998E12372	0.17	1.22	*HDGF*	Hepatoma-derived growth factor (high-mobility group protein 1-like)
286	RZPDp201D0230D	0.17	1.15	*LBR*	Lamin B receptor
287	IMAGp998D11276	0.17	1.13	*GPBP1*	GC-rich promoter binding protein 1
288	RZPDp202D113D	0.17	1.14	*URG4*	Up-regulated gene 4
289	RZPDp201D021D	0.17	1.24	*GPR21*	G protein-coupled receptor 21
290	IMAGp998C06286	0.17	2.07	*KIAA1840*	KIAA1840
291	IMAGp998M13143	0.17	1.18	*GEM*	GTP binding protein overexpressed in skeletal muscle
292	IMAGp998K121778	0.17	1.18	*SLC39A9*	Solute carrier family 39 (zinc transporter), member 9
293	RZPDp202C018D	0.17	1.18	*IDH2*	Isocitrate dehydrogenase 2 (NADP+), mitochondrial
294	IMAGp998O22144	0.17	1.35	*MAP3K5*	Mitogen-activated protein kinase kinase kinase 5
295	RZPDp1096G0616D	0.17	1.18	*MAN2A2*	Mannosidase, alpha, class 2A, member 2
296	IMAGp998N23220	0.17	1.18	*MAP3K1*	Mitogen-activated protein kinase kinase kinase 1
297	IMAGp998F23844	0.17	1.21	*LOC285548*	Hypothetical protein LOC285548
298	IMAGp998H12976	0.17	1.17	*ATAD5*	ATPase family, AAA domain containing 5
299	RZPDp201A011D	0.17	1.24	*SPAG17*	Sperm associated antigen 17
300	RZPDp201E0634D	0.17	1.24	*PGM2*	Phosphoglucomutase 2
301	IMAGp998O21408	0.17	1.25	*H1F0*	H1 histone family, member 0
302	RZPDp1096G067D	0.17	1.22	*PTMA*	Prothymosin, alpha (gene sequence 28)
303	IMAGp998I13225	0.17	1.16	*SYPL1*	Synaptophysin-like 1
304	IMAGp998E201114	0.17	1.19	*RCC2*	Regulator of chromosome condensation 2
305	RZPDp202F027D	0.17	1.11	*COX10*	COX10 homolog, cytochrome c oxidase assembly protein, heme A: farnesyltransferase (yeast)
306	IMAGp998E1697	0.17	1.16	*TSPAN3*	Tetraspanin 3
307	IMAGp998A11675	0.17	1.19	*SNX2*	Sorting nexin 2
308	RZPDp1096G0513D	0.17	1.39	*ATP5A1*	ATP synthase, H+ transporting, mitochondrial F1 complex, alpha subunit 1, cardiac muscle
309	RZPDp1096C1119D	0.17	1.20	*N/A*	Data not found
310	RZPDp201C1129D	0.17	1.17	*ROCK1*	Rho-associated, coiled-coil containing protein kinase 1
311	IMAGp998I22159	0.17	1.19	*SIAHBP1*	Fuse-binding protein-interacting repressor
312	IMAGp998J2374	0.17	1.18	*C5orf15*	Chromosome 5 open reading frame 15
313	RZPDp201D039D	0.17	1.17	*COX7B*	Cytochrome c oxidase subunit VIIb
314	IMAGp998J14274	0.17	1.23	*KIAA1191*	KIAA1191
315	RZPDp201C0217D	0.17	1.14	*TRIM44*	Tripartite motif-containing 44
316	IMAGp998P16659	0.17	1.31	*POLR2B*	Polymerase (RNA) II (DNA directed) polypeptide B, 140kDa
317	IMAGp998I22404	0.17	1.28	*HSPA8*	Heat shock 70kDa protein 8
318	IMAGp998O22440	0.17	1.09	*TAF12*	TAF12 RNA polymerase II, TATA box binding protein (TBP)-associated factor, 20kDa
319	IMAGp998P04272	0.17	1.14	*COX7A2L*	Cytochrome c oxidase subunit VIIa polypeptide 2 like
320	RZPDp1096D017D	0.17	1.50	*PCDHAC2*	Protocadherin alpha subfamily C, 2
321	IMAGp998P222312	0.17	1.33	*N/A*	Transcribed locus
322	IMAGp998O22824	0.17	1.51	*CXCL14*	Chemokine (C-X-C motif) ligand 14
323	RZPDp202A019D	0.17	1.27	*SNF1LK2*	SNF1-like kinase 2
324	IMAGp998K06152	0.17	1.24	*YBX1*	Y box binding protein 1
325	IMAGp998I071725	0.17	1.20	*TMEM2*	Transmembrane protein 2
326	IMAGp998N08976	0.17	1.29	*N/A*	Data not found
327	RZPDp201E1136D	0.17	1.17	*SND1*	Staphylococcal nuclease and tudor domain containing 1
328	IMAGp998N15154	0.17	1.15	*N/A*	Transcribed locus, weakly similar to NP_009956.2 homologue; Rhb1p [Saccharomyces cerevisiae]
329	IMAGp998O011116	0.17	1.16	*SLAIN2*	SLAIN motif family, member 2
330	RZPDp201A0232D	0.17	1.31	*HMGB1*	High-mobility group box 1
331	RZPDp202E026D	0.17	1.13	*SRCAP*	Snf2-related CBP activator protein
332	IMAGp998B20284	0.17	1.21	*H2AFY2*	H2A histone family, member Y2
333	IMAGp998D08131	0.17	1.25	*IRF2BP2*	Interferon regulatory factor 2 binding protein 2
334	IMAGp998E021999	0.17	1.11	*SUPT4H1*	Suppressor of Ty 4 homolog 1 (S. cerevisiae)
335	IMAGp998B13278	0.17	1.19	*MKRN1*	Makorin, ring finger protein, 1
336	IMAGp998I06123	0.17	1.37	*EIF3S10*	Eukaryotic translation initiation factor 3, subunit 10 theta, 150/170kDa
337	RZPDp201F0328D	0.17	1.30	*IFIT1*	Interferon-induced protein with tetratricopeptide repeats 1
338	IMAGp998A19414	0.17	1.21	*UQCRC2*	Ubiquinol-cytochrome c reductase core protein II
339	IMAGp998D07314	0.17	1.16	*SNRPG*	Small nuclear ribonucleoprotein polypeptide G
340	RZPDp201D1231D	0.17	1.21	*RGS12*	Regulator of G-protein signalling 12
341	IMAGp998P20173	0.17	1.19	*EIF4H*	Eukaryotic translation initiation factor 4H
342	IMAGp998I08401	0.17	1.18	*FARSB*	Phenylalanyl-tRNA synthetase, beta subunit
343	IMAGp998K11275	0.17	1.17	*H2AFY*	H2A histone family, member Y
344	IMAGp998E05186	0.17	1.11	*COIL*	Coilin
345	IMAGp998L13786	0.17	1.22	*ZNF364*	Zinc finger protein 364
346	IMAGp998H11332	0.17	1.21	*TUBB3*	Tubulin, beta 3
347	RZPDp201D0236D	0.17	1.22	*CNOT2*	CCR4-NOT transcription complex, subunit 2
348	IMAGp998C151162	0.17	1.17	*TRIOBP*	TRIO and F-actin binding protein
349	IMAGp998H045568	0.17	1.11	*CASK*	Calcium/calmodulin-dependent serine protein kinase (MAGUK family)
350	IMAGp998D20786	0.17	1.14	*RBM39*	RNA binding motif protein 39
351	RZPDp202E035D	0.17	1.32	*FZD7*	Frizzled homolog 7 (Drosophila)
352	IMAGp998A07148	0.17	1.23	*FGF1*	Fibroblast growth factor 1 (acidic)
353	RZPDp202E021D	0.17	1.12	*LHX2*	LIM homeobox 2
354	RZPDp201G0515D	0.17	1.32	*N/A*	Transcribed locus
355	IMAGp998C1773	0.17	1.17	*N/A*	Transcribed locus
356	IMAGp998K22833	0.17	1.12	*SRP14*	Signal recognition particle 14kDa (homologous Alu RNA binding protein)
357	RZPDp202F073D	0.17	1.21	*C7orf28B*	Chromosome 7 open reading frame 28B
358	IMAGp998A17282	0.30	1.54	*CACNA1B*	Calcium channel, voltage-dependent, N type, alpha 1B subunit
359	IMAGp998H02230	0.30	1.29	*GATA2*	GATA binding protein 2
360	IMAGp998O16285	0.30	1.13	*SEP15*	15 kDa selenoprotein
361	IMAGp998L21565	0.30	1.17	*XPO1*	Exportin 1 (CRM1 homolog, yeast)
362	RZPDp202E086D	0.30	1.15	*HMBS*	Hydroxymethylbilane synthase
363	RZPDp201E0635D	0.30	1.14	*GNAS*	GNAS complex locus
364	IMAGp998A08198	0.30	1.16	*STT3B*	STT3, subunit of the oligosaccharyltransferase complex, homolog B (S. cerevisiae)
365	IMAGp998E181777	0.30	1.15	*SLC39A11*	Solute carrier family 39 (metal ion transporter), member 11
366	RZPDp202H054D	0.30	1.13	*BIRC7*	Baculoviral IAP repeat-containing 7 (livin)
367	RZPDp201E0234D	0.30	1.22	*DLAT*	Dihydrolipoamide S-acetyltransferase (E2 component of pyruvate dehydrogenase complex)
368	RZPDp202D072D	0.30	1.13	*RGS16*	Regulator of G-protein signalling 16
369	IMAGp998J19276	0.30	1.19	*HCFC1*	Host cell factor C1 (VP16-accessory protein)
370	IMAGp998G081786	0.30	1.16	*EFCBP1*	EF-hand calcium binding protein 1
371	IMAGp998D06543	0.30	1.18	*DHRS1*	Dehydrogenase/reductase (SDR family) member 1
372	IMAGp998E23314	0.30	1.20	*KIAA1718*	KIAA1718 protein
373	IMAGp998D12135	0.30	1.22	*KRTCAP2*	Keratinocyte associated protein 2
374	IMAGp998J24664	0.30	1.20	*MFHAS1*	Malignant fibrous histiocytoma amplified sequence 1
375	RZPDp201F0632D	0.30	1.27	*FAM120A*	Family with sequence similarity 120A
376	RZPDp201H0328D	0.30	1.21	*SDAD1*	SDA1 domain containing 1
377	IMAGp998J221748	0.30	1.12	*ARIH1*	Ariadne homolog, ubiquitin-conjugating enzyme E2 binding protein, 1 (Drosophila)
378	IMAGp998K11247	0.30	1.10	*POLR2E*	Polymerase (RNA) II (DNA directed) polypeptide E, 25kDa
379	IMAGp998M171898	0.30	1.18	*PSMB2*	Proteasome (prosome, macropain) subunit, beta type, 2
380	IMAGp998I151787	0.30	1.13	*TUBA3E*	Tubulin, alpha 3e
381	IMAGp998I181855	0.30	1.17	*AHSA1*	AHA1, activator of heat shock 90kDa protein ATPase homolog 1 (yeast)
382	IMAGp998M24393	0.30	1.17	*TRAPPC5*	Trafficking protein particle complex 5
383	IMAGp998C1476	0.30	1.27	*RPL22*	Ribosomal protein L22
384	IMAGp998L131007	0.30	1.11	*STK36*	Serine/threonine kinase 36, fused homolog (Drosophila)
385	IMAGp998O13616	0.30	1.21	*SSU72*	SSU72 RNA polymerase II CTD phosphatase homolog (S. cerevisiae)
386	IMAGp998B04178	0.30	1.23	*OCIAD1*	OCIA domain containing 1
387	IMAGp998H07312	0.30	1.12	*PRDX5*	Peroxiredoxin 5
388	IMAGp998M15230	0.30	1.12	*COPS2*	COP9 constitutive photomorphogenic homolog subunit 2 (Arabidopsis)
389	RZPDp1096B044D	0.30	1.21	*CLK1*	CDC-like kinase 1
390	IMAGp998D03170	0.30	1.38	*TMEM123*	Transmembrane protein 123
391	RZPDp1096F125D	0.30	1.28	*EIF2C2*	Eukaryotic translation initiation factor 2C, 2
392	IMAGp998F09658	0.30	1.14	*C17orf32*	Chromosome 17 open reading frame 32
393	RZPDp202F113D	0.30	1.12	*FARSA*	Phenylalanyl-tRNA synthetase, alpha subunit
394	RZPDp201B0633D	0.30	1.20	*PCCB*	Propionyl Coenzyme A carboxylase, beta polypeptide
395	IMAGp998C11819	0.30	1.13	*C19orf50*	Chromosome 19 open reading frame 50
396	RZPDp1096C1018D	0.30	1.14	*SKP1A*	S-phase kinase-associated protein 1A (p19A)
397	IMAGp998G07165	0.30	1.10	*UFC1*	Ubiquitin-fold modifier conjugating enzyme 1
398	IMAGp998D094458	0.30	1.16	*N/A*	Transcribed locus
399	IMAGp998O21375	0.30	1.42	*KIAA0372*	KIAA0372
400	IMAGp998F18170	0.40	1.25	*CRYL1*	Crystallin, lambda 1
401	IMAGp998E21185	0.40	1.17	*ARMC1*	Armadillo repeat containing 1
402	IMAGp998N11270	0.40	1.25	*TSPYL5*	TSPY-like 5
403	RZPDp1096D106D	0.40	1.08	*ZNF366*	Zinc finger protein 366
404	IMAGp998B0974	0.40	1.14	*AGPAT7*	1-acylglycerol-3-phosphate O-acyltransferase 7 (lysophosphatidic acid acyltransferase, eta)
405	IMAGp998N21598	0.40	1.13	*ARCN1*	Archain 1

406	IMAGp998I102226	0.40	1.21	N/A	Transcribed locus
407	RZPDp201E0615D	0.40	1.15	SIX2	Sine oculis homeobox homolog 2 (Drosophila)
408	IMAGp998K231858	0.40	1.11	C5orf26	Chromosome 5 open reading frame 26
409	IMAGp998L03214	0.40	1.20	N/A	Transcribed locus
410	RZPDp1096B016D	0.40	1.24	N/A	Data not found
411	IMAGp998M02340	0.40	1.24	HLA-DMA	Major histocompatibility complex, class II, DM alpha
412	IMAGp998G11583	0.40	1.19	CYP24A1	Cytochrome P450, family 24, subfamily A, polypeptide 1
413	IMAGp998B06472	0.40	1.16	FVT1	Follicular lymphoma variant translocation 1
414	IMAGp998K034506	0.40	1.30	HIST1H4B	Histone cluster 1, H4b
415	RZPDp1096A051D	0.40	1.10	ATP6V1F	ATPase, H+ transporting, lysosomal 14kDa, V1 subunit F
416	RZPDp1096G073D	0.40	1.13	N/A	CDNA FLJ33024 fis, clone THYMU1000532
417	IMAGp998D09597	0.40	1.18	COL1A2	Collagen, type I, alpha 2
418	IMAGp998D071855	0.40	1.13	NUP50	Nucleoporin 50kDa
419	IMAGp998O13473	0.40	1.25	N/A	Data not found
420	IMAGp998P16166	0.40	1.14	N/A	Transcribed locus, strongly similar to XP_527520.2 neuromedin B receptor [Pan troglodytes]
421	RZPDp1096A011D	0.40	1.51	RNF8	Ring finger protein 8
422	IMAGp998G2370	0.40	1.21	IARS	Isoleucyl-tRNA synthetase
423	RZPDp201B0933D	0.40	1.19	SFRS1	Splicing factor, arginine/serine-rich 1 (splicing factor 2, alternate splicing factor)
424	IMAGp998O092001	0.40	1.22	UBE2I	Ubiquitin-conjugating enzyme E2I (UBC9 homolog, yeast)
425	RZPDp202D062D	0.40	1.14	KLHL21	Kelch-like 21 (Drosophila)
426	RZPDp201E1135D	0.40	1.15	TTF2	Transcription termination factor, RNA polymerase II
427	RZPDp201D0628D	0.40	1.24	LARP1	La ribonucleoprotein domain family, member 1
428	IMAGp998J1879	0.40	1.11	N/A	Data not found
429	IMAGp998P091965	0.40	1.17	BMP4	Bone morphogenetic protein 4
430	IMAGp998K17543	0.40	1.15	CNN3	Calponin 3, acidic
431	IMAGp998F06678	0.40	1.12	JDP2	Jun dimerization protein 2
432	RZPDp202H093D	0.40	1.17	SH3BGRL3	SH3 domain binding glutamic acid-rich protein like 3
433	RZPDp202F051D	0.40	1.21	GPATCH8	G patch domain containing 8
434	IMAGp998B15185	0.40	1.18	CLTA	Clathrin, light chain (Lca)
435	RZPDp1096A082D	0.40	1.19	KIAA0329	KIAA0329
436	RZPDp202G067D	0.40	1.14	BCL3	B-cell CLL/lymphoma 3
437	IMAGp998D171169	0.40	1.27	SACM1L	SAC1 suppressor of actin mutations 1-like (yeast)
438	IMAGp998A0372	0.40	1.14	HNRPU	Heterogeneous nuclear ribonucleoprotein U (scaffold attachment factor A)
439	RZPDp201A0117D	0.40	1.12	EIF4ENIF1	Eukaryotic translation initiation factor 4E nuclear import factor 1
440	IMAGp998A09338	0.40	1.19	DDR1	Discoidin domain receptor family, member 1
441	IMAGp998O05787	0.40	1.17	F8A1	Coagulation factor VIII-associated (intronic transcript) 1
442	IMAGp998J21787	0.40	1.08	GNPAT	Glyceronephosphate O-acyltransferase
443	IMAGp998B17175	0.40	1.17	N/A	Data not found
444	RZPDp201C0229D	0.40	1.10	KIF5B	Kinesin family member 5B
445	RZPDp201B0420D	0.50	1.09	CRTC3	CREB regulated transcription coactivator 3
446	RZPDp202E078D	0.50	1.11	ARF3	ADP-ribosylation factor 3
447	IMAGp998F16171	0.50	1.09	ATRN	Attractin
448	RZPDp201H0528D	0.50	1.12	RKHD2	Ring finger and KH domain containing 2
449	IMAGp998C06469	0.50	1.31	PB1	Polybromo 1
450	IMAGp998O23147	0.50	1.13	SPTAN1	Spectrin, alpha, non-erythrocytic 1 (alpha-fodrin)
451	IMAGp998L01121	0.50	1.22	C15orf15	Chromosome 15 open reading frame 15
452	IMAGp998P211052	0.50	1.11	N/A	Transcribed locus
453	RZPDp1096D0613D	0.50	1.13	SEPT2	Septin 2
454	IMAGp998K18671	0.50	1.16	NME1	Non-metastatic cells 1, protein (NM23A) expressed in
455	RZPDp202E096D	0.50	1.12	MAGOH	Mago-nashi homolog, proliferation-associated (Drosophila)
456	RZPDp201D0627D	0.50	1.17	USP21	Ubiquitin specific peptidase 21
457	IMAGp998H02691	0.50	1.18	ZCCHC17	Zinc finger, CCHC domain containing 17
458	IMAGp998P13154	0.50	1.11	PPP4R1	Protein phosphatase 4, regulatory subunit 1
459	IMAGp998M16210	0.50	1.15	MATR3	Matrin 3
460	RZPDp202E116D	0.50	1.23	TRIM25	Tripartite motif-containing 25
461	RZPDp1096C022D	0.50	2.40	N/A	Px19 protein pseudogene mRNA, partial sequence
462	IMAGp998H202012	0.50	1.21	N/A	Transcribed locus
463	IMAGp998G2018	0.50	1.18	N/A	CDNA FLJ32348 fis, clone PROST2007200
464	IMAGp998F02111	0.50	1.09	ARHGAP21	Rho GTPase activating protein 21
465	IMAGp998P09274	0.50	1.12	PMVK	Phosphomevalonate kinase
466	RZPDp1096G056D	0.50	1.13	SGPP2	Sphingosine-1-phosphate phosphotase 2
467	IMAGp998K183150	0.50	1.14	N/A	Transcribed locus, strongly similar to XP_001071840.1 similar to autism susceptibility candidate 2 [Rattus norvegicus]
468	IMAGp998C101864	0.50	1.10	TEX10	Testis expressed sequence 10
469	IMAGp998C07283	0.50	1.19	ADCK2	AarF domain containing kinase 2
470	RZPDp201C0933D	0.50	1.22	FKBP4	FK506 binding protein 4, 59kDa
471	IMAGp998G24794	0.50	1.18	N/A	CDNA FLJ38388 fis, clone FEBRA2004485
472	IMAGp998G18157	0.50	1.16	PTPRN	Protein tyrosine phosphatase, receptor type, N
473	IMAGp998N13665	0.50	1.75	PLA2G2A	Phospholipase A2, group IIA (platelets, synovial fluid)
474	RZPDp1096G1215D	0.50	1.18	RBBP4	Retinoblastoma binding protein 4
475	IMAGp998F08139	0.50	1.16	SIN3A	SIN3 homolog A, transcription regulator (yeast)
476	RZPDp202F127D	0.50	1.12	OXA1L	Oxidase (cytochrome c) assembly 1-like
477	IMAGp998O17461	0.50	1.75	COL2A1	Collagen, type II, alpha 1 (primary osteoarthritis, spondyloepiphyseal dysplasia, congenital)
478	IMAGp998F11736	0.50	1.13	PARN	Poly(A)-specific ribonuclease (deadenylation nuclease)
479	IMAGp998G23650	0.50	1.15	RIC8A	Resistance to inhibitors of cholinesterase 8 homolog A (C. elegans)
480	IMAGp998C08143	0.50	1.17	NGFR	Nerve growth factor receptor (TNFR superfamily, member 16)
481	IMAGp998D07421	0.50	1.11	ATP6V1H	ATPase, H+ transporting, lysosomal 50/57kDa, V1 subunit H
482	RZPDp201D0420D	0.50	1.17	TMEM45B	Transmembrane protein 45B
483	RZPDp202A035D	0.50	1.12	TRIM47	Tripartite motif-containing 47
484	RZPDp1096A0717D	0.50	1.08	MARCH6	Membrane-associated ring finger (C3HC4) 6
485	RZPDp201D0536D	0.50	1.33	C6orf51	Chromosome 6 open reading frame 51
486	IMAGp998A01519	0.56	1.11	FLJ35348	FLJ35348
487	RZPDp1096E0514D	0.56	1.19	ZNF146	Zinc finger protein 146
488	IMAGp998B134113	0.56	1.15	N/A	Full length insert cDNA clone ZD75H06
489	IMAGp998I09822	0.56	1.13	P15RS	Hypothetical protein FLJ10656
490	IMAGp998P14135	0.56	1.27	ATP6V1B2	ATPase, H+ transporting, lysosomal 56/58kDa, V1 subunit B2
491	IMAGp998P18665	0.56	1.13	PAPD4	PAP associated domain containing 4
492	IMAGp998I03176	0.56	1.28	CRYM	Crystallin, mu
493	IMAGp998M12660	0.56	1.28	SERINC3	Serine incorporator 3
494	RZPDp202C107D	0.56	1.10	LOC283951	Hypothetical protein LOC283951
495	IMAGp998M15411	0.56	1.23	RPL30	Ribosomal protein L30
496	RZPDp201A0329D	0.56	1.29	IDH1	Isocitrate dehydrogenase 1 (NADP+), soluble
497	RZPDp202A089D	0.56	1.09	KIAA0841	KIAA0841
498	IMAGp998F16827	0.56	1.18	N/A	Transcribed locus
499	IMAGp998I18158	0.56	1.17	RBM4	RNA binding motif protein 4
500	RZPDp202F036D	0.56	1.18	YWHAZ	Tyrosine 3-monooxygenase/tryptophan 5-monooxygenase activation protein, zeta polypeptide
501	RZPDp202D108D	0.56	1.21	IK	IK cytokine, down-regulator of HLA II
502	IMAGp998A17666	0.56	1.39	C17orf63	Chromosome 17 open reading frame 63
503	IMAGp998N20699	0.56	1.45	N/A	Clone FBD8 Cri-du-chat critical region mRNA
504	IMAGp998M184415	0.56	1.16	NAIP	NLR family, apoptosis inhibitory protein
505	IMAGp998H19786	0.56	1.38	TTC14	Tetratricopeptide repeat domain 14
506	RZPDp201C0533D	0.56	1.13	DYNLT3	Dynein, light chain, Tctex-type 3
507	IMAGp998D08281	0.56	1.18	WBSCR17	Williams-Beuren syndrome chromosome region 17
508	IMAGp998F09163	0.56	1.15	TUBB4	Tubulin, beta 4
509	IMAGp998M081724	0.56	1.14	ZNF403	Zinc finger protein 403
510	IMAGp998N04280	0.56	1.15	SYF2	SYF2 homolog, RNA splicing factor (S. cerevisiae)
511	IMAGp998O03131	0.56	1.10	MKI67IP	MKI67 (FHA domain) interacting nucleolar phosphoprotein
512	IMAGp998L07117	0.56	1.12	N/A	CDNA clone IMAGE:4842353
513	IMAGp998L22373	0.56	1.23	AQP3	Aquaporin 3 (Gill blood group)
514	IMAGp998F031854	0.56	1.22	IKBKAP	Inhibitor of kappa light polypeptide gene enhancer in B-cells, kinase complex-associated protein
515	RZPDp202H128D	0.56	1.20	SORD	Sorbitol dehydrogenase
516	IMAGp998H071204	0.56	1.17	RBM25	RNA binding motif protein 25
517	RZPDp201C1127D	0.56	1.14	PCDHGC3	Protocadherin gamma subfamily C, 3
518	RZPDp202H058D	0.56	1.17	C14orf166	Chromosome 14 open reading frame 166
519	IMAGp998D024682	0.56	1.15	MTX3	Metaxin 3
520	IMAGp998A06781	0.56	1.24	NSF	N-ethylmaleimide-sensitive factor
521	RZPDp1096C1120D	0.56	1.16	RAB1B	RAB1B, member RAS oncogene family
522	IMAGp998C011157	0.64	1.35	CLK1	CDC-like kinase 1
523	IMAGp998A16170	0.64	1.19	PABPC1	Poly(A) binding protein, cytoplasmic 1
524	IMAGp998L19593	0.64	1.19	PAFAH1B2	Platelet-activating factor acetylhydrolase, isoform Ib, beta subunit 30kDa
525	IMAGp998P114129	0.64	1.22	N/A	Data not found
526	RZPDp201D1219D	0.64	1.20	LOC728643	Heterogeneous nuclear ribonucleoprotein A1 pseudogene
527	RZPDp202D066D	0.64	1.15	FHOD1	Formin homology 2 domain containing 1
528	IMAGp998A10618	0.64	1.16	RP11-217H1.1	Implantation-associated protein
529	IMAGp998H21524	0.64	1.15	N/A	CDNA FLJ23712 fis, clone HEP12427
530	IMAGp998G09159	0.64	1.26	ADAMTS1	ADAM metallopeptidase with thrombospondin type 1 motif, 1
531	IMAGp998C14840	0.64	1.20	N/A	Data not found
532	RZPDp202G058D	0.64	1.14	DDB1	Damage-specific DNA binding protein 1, 127kDa
533	IMAGp998H185671	0.64	1.18	N/A	Transcribed locus
534	IMAGp998N171724	0.64	1.30	EEF2	Eukaryotic translation elongation factor 2
535	IMAGp998N071854	0.64	1.29	LRP11	Low density lipoprotein receptor-related protein 11
536	RZPDp1096D1214D	0.64	1.25	TMEM14B	Transmembrane protein 14B
537	RZPDp1096A0718D	0.64	1.12	NUB1	Negative regulator of ubiquitin-like proteins 1
538	RZPDp201D1234D	0.64	1.21	POLR3K	Polymerase (RNA) III (DNA directed) polypeptide K, 12.3 kDa
539	IMAGp998B22272	0.64	1.19	RAD23A	RAD23 homolog A (S. cerevisiae)
540	RZPDp201E0616D	0.64	1.14	HHATL	Hedgehog acyltransferase-like

541	IMAGp998N141824	0.64	1.11	*USP12*	Ubiquitin specific peptidase 12
542	IMAGp998O21276	0.64	1.13	*SNRPD1*	Small nuclear ribonucleoprotein D1 polypeptide 16kDa
543	IMAGp998J10400	0.64	1.10	*SCAMP3*	Secretory carrier membrane protein 3
544	IMAGp998D09384	0.64	1.18	*N/A*	Transcribed locus
545	IMAGp998O05287	0.64	1.69	*GRIA1*	Glutamate receptor, ionotropic, AMPA 1
546	IMAGp998E161860	0.64	1.13	*IGSF9*	Immunoglobulin superfamily, member 9
547	RZPDp1096A0117D	0.64	1.14	*ARPC3*	Actin related protein 2/3 complex, subunit 3, 21kDa
548	IMAGp998A022234	0.64	1.24	*CAPN13*	Calpain 13
549	IMAGp998J23420	0.64	1.13	*N/A*	Transcribed locus, strongly similar to NP_066407.1 histone family, member B [Homo sapiens]
550	IMAGp998E08142	0.64	1.20	*NCDN*	Neurochondrin
551	IMAGp998H04417	0.64	1.16	*GLI2*	GLI-Kruppel family member GLI2
552	IMAGp998I231864	0.64	1.13	*N/A*	Transcribed locus
553	IMAGp998A12536	0.80	1.23	*KIAA0143*	KIAA0143 protein
554	IMAGp998L094652	0.80	1.17	*N/A*	Transcribed locus
555	RZPDp1096A0313D	0.80	1.42	*HSP90AA2*	Heat shock protein 90kDa alpha (cytosolic), class A member 2
556	RZPDp1096A0219D	0.80	1.15	*NFYC*	Nuclear transcription factor Y, gamma
557	IMAGp998M15530	0.80	1.12	*SRPK1*	SFRS protein kinase 1
558	IMAGp998J161933	0.80	1.25	*ZNF192*	Zinc finger protein 192
559	IMAGp998O11274	0.80	1.17	*CAMTA1*	Calmodulin binding transcription activator 1
560	RZPDp202H053D	0.80	1.19	*KCNN2*	Potassium intermediate/small conductance calcium-activated channel, subfamily N, member 2
561	RZPDp1096D122D	0.80	1.11	*ARMCX6*	Armadillo repeat containing, X-linked 6
562	IMAGp998E05281	0.80	1.38	*GLS*	Glutaminase
563	IMAGp998G18258	0.80	1.19	*TEGT*	Testis enhanced gene transcript (BAX inhibitor 1)
564	IMAGp998A12286	0.80	1.25	*MFNG*	MFNG O-fucosylpeptide 3-beta-N-acetylglucosaminyltransferase
565	IMAGp998M131113	0.80	1.14	*BOLA3*	BolA homolog 3 (E. coli)
566	IMAGp998L05198	0.80	1.10	*RSBN1*	Round spermatid basic protein 1
567	IMAGp998I15475	0.80	1.12	*TMEM41B*	Transmembrane protein 41B
568	IMAGp998M17467	0.80	1.12	*RBM27*	RNA binding motif protein 27
569	IMAGp998F025242	0.80	1.22	*N/A*	Data not found
570	RZPDp202E087D	0.80	1.12	*LOC219854*	Hypothetical protein LOC219854
571	IMAGp998I01473	0.80	1.14	*GOLGA4*	Golgi autoantigen, golgin subfamily a, 4
572	IMAGp998O03648	0.80	1.12	*SSX4*	Synovial sarcoma, X breakpoint 4
573	RZPDp202A027D	0.80	1.16	*EIF2S2*	Eukaryotic translation initiation factor 2, subunit 2 beta, 38kDa
574	IMAGp998H04729	0.80	1.47	*C3orf63*	Chromosome 3 open reading frame 63
575	IMAGp998O17176	0.80	1.13	*N/A*	Full-length cDNA clone CS0DM001YA04 of Fetal liver of Homo sapiens (human)
576	IMAGp998P05869	0.80	1.14	*FPGS*	Folylpolyglutamate synthase
577	RZPDp201F0929D	0.80	1.16	*HNRPD*	Heterogeneous nuclear ribonucleoprotein D (AU-rich element RNA binding protein 1, 37kDa)
578	IMAGp998E11272	0.80	1.20	*SBF1*	SET binding factor 1
579	RZPDp202E048D	0.80	1.09	*SLC23A2*	Solute carrier family 23 (nucleobase transporters), member 2
580	IMAGp998E21690	0.80	1.40	*COL3A1*	Collagen, type III, alpha 1 (Ehlers-Danlos syndrome type IV, autosomal dominant)
581	IMAGp998B026136	0.80	1.12	*TMEM28*	Transmembrane protein 28
582	RZPDp202A021D	0.80	1.23	*NBPF3*	Neuroblastoma breakpoint family, member 3
583	IMAGp998H06241	0.80	1.12	*SRM*	Spermidine synthase
584	IMAGp998F04172	0.80	1.26	*SLC25A5*	Solute carrier family 25 (mitochondrial carrier; adenine nucleotide translocator), member 5
585	RZPDp202B104D	0.80	1.14	*RPA2*	Replication protein A2, 32kDa
586	IMAGp998J13784	0.80	1.09	*N/A*	Clone 24841 mRNA sequence
587	IMAGp998I20270	0.80	1.50	*C21orf130*	Chromosome 21 open reading frame 130
588	IMAGp998P171858	0.80	1.09	*SUV39H1*	Suppressor of variegation 3-9 homolog 1 (Drosophila)
589	IMAGp998G23793	0.80	1.12	*ALDH3B2*	Aldehyde dehydrogenase 3 family, member B2
590	IMAGp998D154408	0.80	1.17	*CD19*	CD19 molecule
591	IMAGp998K17160	0.94	1.20	*SYT1*	Synaptotagmin I
592	IMAGp998N081962	0.94	1.13	*COPS5*	COP9 constitutive photomorphogenic homolog subunit 5 (Arabidopsis)
593	IMAGp998L145682	0.94	1.19	*N/A*	Transcribed locus
594	IMAGp998H025601	0.94	1.27	*NOLA1*	Nucleolar protein family A, member 1 (H/ACA small nucleolar RNPs)
595	IMAGp998N08441	0.94	1.18	*CSDA*	Cold shock domain protein A
596	RZPDp1096F097D	0.94	1.20	*SYT1*	Synaptotagmin I
597	IMAGp998K18247	0.94	1.19	*CRY2*	Cryptochrome 2 (photolyase-like)
598	RZPDp202C054D	0.94	1.22	*TLX2*	T-cell leukemia homeobox 2
599	IMAGp998D11152	0.94	1.13	*PUM2*	Pumilio homolog 2 (Drosophila)
600	RZPDp201G0217D	0.94	1.11	*PMS2L2*	Postmeiotic segregation increased 2-like 2
601	IMAGp998C12887	0.94	1.19	*TRH*	Thyrotropin-releasing hormone
602	RZPDp1096F0816D	0.94	1.16	*TNFRSF21*	Tumor necrosis factor receptor superfamily, member 21
603	IMAGp998K07535	0.94	1.21	*PPAP2B*	Phosphatidic acid phosphatase type 2B
604	RZPDp1096F032D	0.94	1.14	*NR3C1*	Nuclear receptor subfamily 3, group C, member 1 (glucocorticoid receptor)
605	RZPDp1096C1117D	0.94	1.13	*MAGEA12*	Melanoma antigen family A, 12
606	IMAGp998K13519	0.94	1.17	*PGRMC2*	Progesterone receptor membrane component 2
607	IMAGp998I10385	0.94	1.26	*ALDH9A1*	Aldehyde dehydrogenase 9 family, member A1
608	RZPDp202B015D	0.94	1.11	*CXorf26*	Chromosome X open reading frame 26
609	IMAGp998B2173	0.94	1.15	*ZMYND11*	Zinc finger, MYND domain containing 11
610	IMAGp998C06652	0.94	1.18	*ACAT2*	Acetyl-Coenzyme A acetyltransferase 2 (acetoacetyl Coenzyme A thiolase)
611	IMAGp998D10578	0.94	1.11	*CPSF6*	Cleavage and polyadenylation specific factor 6, 68kDa
612	IMAGp998O05724	0.94	1.66	*FLJ21687*	PDZ domain containing, X chromosome
613	IMAGp998A16295	0.94	1.09	*CLPTM1*	Cleft lip and palate associated transmembrane protein 1
614	IMAGp998M0484	0.94	1.26	*DHRS7*	Dehydrogenase/reductase (SDR family) member 7
615	IMAGp998L04477	0.94	1.08	*TBC1D10B*	TBC1 domain family, member 10B
616	IMAGp998I1487	0.94	1.09	*AAAS*	Achalasia, adrenocortical insufficiency, alacrimia (Allgrove, triple-A)
617	IMAGp998L12830	0.94	1.28	*N/A*	Data not found
618	IMAGp998H03792	0.94	1.20	*NPAL2*	NIPA-like domain containing 2
619	IMAGp998B13269	0.94	1.10	*POLG2*	Polymerase (DNA directed), gamma 2, accessory subunit
620	RZPDp1096G044D	0.94	1.20	*PNRC2*	Proline-rich nuclear receptor coactivator 2
621	IMAGp998I17287	0.94	1.13	*DIAPH1*	Diaphanous homolog 1 (Drosophila)
622	IMAGp998A06784	0.94	1.23	*FUS*	Fusion (involved in t(12;16) in malignant liposarcoma)
623	IMAGp998F041817	0.94	1.28	*HIP1*	Huntingtin interacting protein 1
624	RZPDp1096C0815D	0.94	1.13	*OPN4*	Opsin 4 (melanopsin)
625	IMAGp998E18235	0.94	1.11	*ZFYVE21*	Zinc finger, FYVE domain containing 21
626	RZPDp201H1135D	0.94	1.14	*ZNF281*	Zinc finger protein 281
627	IMAGp998E12654	0.94	1.28	*SORL1*	Sortilin-related receptor, L(DLR class) A repeats-containing
628	IMAGp998M03537	0.94	1.22	*APLP2*	Amyloid beta (A4) precursor-like protein 2
629	IMAGp998B044416	0.94	1.15	*LOC780529*	Hypothetical LOC780529
630	IMAGp998L241199	0.94	1.10	*PCBP2*	Poly(rC) binding protein 2
631	IMAGp998F19540	0.94	1.13	*CLPTM1L*	CLPTM1-like
632	IMAGp998A03366	0.94	1.21	*KPNB1*	Karyopherin (importin) beta 1
633	IMAGp998A196055	0.94	1.23	*N/A*	Transcribed locus
634	IMAGp998C081165	0.94	1.13	*NRP1*	Neuropilin 1
635	RZPDp202C057D	0.94	1.09	*DDB1*	Damage-specific DNA binding protein 1, 127kDa
636	IMAGp998B042038	0.94	1.11	*BTF3L4*	Basic transcription factor 3-like 4
637	RZPDp201E1133D	0.94	1.11	*RINT1*	RAD50 interactor 1
638	IMAGp998I10257	0.94	1.11	*YTHDF1*	YTH domain family, member 1
639	IMAGp998H07208	0.94	1.13	*N/A*	Transcribed locus
640	RZPDp201F1234D	0.94	1.14	*SND1*	Staphylococcal nuclease and tudor domain containing 1
641	RZPDp201D0527D	0.94	1.14	*CDC42EP3*	CDC42 effector protein (Rho GTPase binding) 3
642	RZPDp201A0835D	0.94	1.23	*NOLA1*	Nucleolar protein family A, member 1 (H/ACA small nucleolar RNPs)
643	IMAGp998H03794	1.13	1.24	*SDC4*	Syndecan 4
644	IMAGp998N122003	1.13	1.14	*FLOT1*	Flotillin 1
645	RZPDp202G021D	1.13	1.10	*AMMECR1L*	AMME chromosomal region gene 1-like
646	IMAGp998O061892	1.13	1.12	*N/A*	CDNA FLJ12874 fis, clone NT2RP2003769
647	IMAGp998G19572	1.13	1.09	*N/A*	Transcribed locus
648	IMAGp998I075985	1.13	1.18	*N/A*	Transcribed locus
649	Ara-XCP2-6	1.13	1.60	*N/A*	Data not found
650	RZPDp202F107D	1.13	1.09	*GCN1L1*	GCN1 general control of amino-acid synthesis 1-like 1 (yeast)
651	IMAGp998G12370	1.13	1.12	*CCDC23*	Coiled-coil domain containing 23
652	RZPDp201A082D	1.13	1.21	*EIF1AY*	Eukaryotic translation initiation factor 1A, Y-linked
653	IMAGp998N061862	1.13	1.08	*TSR2*	TSR2, 20S rRNA accumulation, homolog (S. cerevisiae)
654	IMAGp998O214514	1.13	1.15	*N/A*	Transcribed locus
655	IMAGp998F01153	1.13	1.18	*UCHL1*	Ubiquitin carboxyl-terminal esterase L1 (ubiquitin thiolesterase)
656	IMAGp998N044258	1.13	1.13	*N/A*	Transcribed locus
657	IMAGp998D014158	1.13	1.14	*N/A*	Data not found
658	IMAGp998N13143	1.13	1.14	*CASC3*	Cancer susceptibility candidate 3
659	RZPDp202E036D	1.13	1.12	*EPRS*	Glutamyl-prolyl-tRNA synthetase
660	RZPDp1096D1217D	1.13	1.10	*PHF5A*	PHD finger protein 5A
661	IMAGp998P161786	1.13	1.13	*TYMS*	Thymidylate synthetase
662	IMAGp998K10153	1.13	1.18	*SRP9*	Signal recognition particle 9kDa
663	RZPDp202A105D	1.13	1.09	*FHOD1*	Formin homology 2 domain containing 1
664	IMAGp998E19276	1.13	1.14	*CYFIP2*	Cytoplasmic FMR1 interacting protein 2
665	IMAGp998C01779	1.13	1.23	*FAT*	FAT tumor suppressor homolog 1 (Drosophila)
666	RZPDp202B076D	1.13	1.62	*EYA4*	Eyes absent homolog 4 (Drosophila)
667	IMAGp998E10655	1.13	1.13	*ARHGEF3*	Rho guanine nucleotide exchange factor (GEF) 3
668	IMAGp998N235376	1.13	1.15	*ZBTB11*	Zinc finger and BTB domain containing 11
669	IMAGp998C07369	1.13	1.13	*N/A*	Transcribed locus
670	IMAGp998D06137	1.13	1.15	*CUGBP1*	CUG triplet repeat, RNA binding protein 1
671	IMAGp998M05615	1.13	1.15	*FAM84B*	Family with sequence similarity 84, member B
672	IMAGp998O08596	1.13	1.13	*N/A*	Data not found
673	IMAGp998L071204	1.13	1.09	*SMC3*	Structural maintenance of chromosomes 3
674	IMAGp998A24620	1.13	1.16	*NECAP1*	NECAP endocytosis associated 1
675	IMAGp998D11664	1.13	1.16	*E2F8*	E2F transcription factor 8

676	RZPDp201A0534D	1.13	2.15	*BLCAP*	Bladder cancer associated protein
677	RZPDp202E046D	1.13	1.18	*CCT3*	Chaperonin containing TCP1, subunit 3 (gamma)
678	IMAGp998N111854	1.13	1.12	*PTGES*	Prostaglandin E synthase
679	IMAGp998H12268	1.13	1.36	*TTC3*	Tetratricopeptide repeat domain 3
680	IMAGp998B105685	1.13	1.24	*ZNF267*	Zinc finger protein 267
681	IMAGp998M03235	1.13	1.14	*SGPP1*	Sphingosine-1-phosphate phosphatase 1
682	IMAGp998H12131	1.13	1.11	*N/A*	Data not found
683	IMAGp998L07469	1.13	1.10	*ZFX*	Zinc finger protein, X-linked
684	IMAGp998F24793	1.29	1.12	*MRFAP1L1*	Morf4 family associated protein 1-like 1
685	IMAGp998G15733	1.29	1.09	*N/A*	CDNA: FLJ23131 fis, clone LNG08502
686	IMAGp998A22167	1.29	1.11	*OSBPL9*	Oxysterol binding protein-like 9
687	IMAGp998I14376	1.29	1.13	*GOLPH3*	Golgi phosphoprotein 3 (coat-protein)
688	RZPDp202C073D	1.29	1.11	*VLDLR*	Very low density lipoprotein receptor
689	IMAGp998P24225	1.29	1.14	*PITPNA*	Phosphatidylinositol transfer protein, alpha
690	IMAGp998A142000	1.29	1.13	*TRIM25*	Tripartite motif-containing 25
691	IMAGp998L235918	1.29	1.16	*N/A*	Transcribed locus
692	RZPDp1096G011D	1.29	1.15	*ITGA11*	Integrin, alpha 11
693	IMAGp998L021905	1.29	1.06	*MRPS22*	Mitochondrial ribosomal protein S22
694	IMAGp998E24218	1.29	1.10	*CHD1L*	Chromodomain helicase DNA binding protein 1-like
695	IMAGp998G091791	1.29	1.15	*KCNJ10*	Potassium inwardly-rectifying channel, subfamily J, member 10
696	IMAGp998E05168	1.29	1.31	*RTN4*	Reticulon 4
697	IMAGp998H18272	1.29	1.11	*KIAA0753*	KIAA0753
698	IMAGp998H04194	1.29	1.13	*HIST1H2BK*	Histone cluster 1, H2bk
699	IMAGp998J10648	1.29	1.12	*OTUD4*	OTU domain containing 4
700	IMAGp998P17726	1.29	1.14	*N/A*	Full-length cDNA clone CS0DI067YK20 of Placenta Cot 25-normalized of Homo sapiens (human)
701	IMAGp998H22631	1.29	1.11	*GARNL1*	GTPase activating Rap/RanGAP domain-like 1
702	RZPDp201E0230D	1.29	1.31	*LRIG1*	Leucine-rich repeats and immunoglobulin-like domains 1
703	RZPDp202H113D	1.29	1.22	*CTTN*	Cortactin
704	IMAGp998L01146	1.29	1.18	*N/A*	CDNA FLJ30378 fis, clone BRACE2007953
705	RZPDp202D016D	1.29	1.10	*COPS6*	COP9 constitutive photomorphogenic homolog subunit 6 (Arabidopsis)
706	RZPDp201E1129D	1.29	1.09	*C12orf52*	Chromosome 12 open reading frame 52
707	RZPDp201B114D	1.29	1.26	*TRIB1*	Tribbles homolog 1 (Drosophila)
708	RZPDp202A056D	1.29	1.38	*PLAT*	Plasminogen activator, tissue
709	IMAGp998F232409	1.29	1.16	*VSIG9*	V-set and immunoglobulin domain containing 9
710	RZPDp202F075D	1.29	1.09	*KCNA1*	Potassium voltage-gated channel, shaker-related subfamily, member 1 (episodic ataxia with myokymia)
711	IMAGp998M12200	1.29	1.13	*C1GALT1*	Core 1 synthase, glycoprotein-N-acetylgalactosamine 3-beta-galactosyltransferase, 1
712	IMAGp998K011928	1.29	1.14	*MAP3K2*	Mitogen-activated protein kinase kinase kinase 2
713	RZPDp1096G0216D	1.29	1.43	*PDIA3*	Protein disulfide isomerase family A, member 3
714	IMAGp998J2389	1.29	1.12	*LSM3*	LSM3 homolog, U6 small nuclear RNA associated (S. cerevisiae)
715	RZPDp201F124D	1.29	1.10	*WWC3*	WWC family member 3
716	RZPDp201B0226D	1.29	1.11	*SNX9*	Sorting nexin 9
717	IMAGp998J15137	1.29	1.16	*N/A*	Transcribed locus
718	RZPDp202C027D	1.29	1.11	*C21orf59*	Chromosome 21 open reading frame 59
719	IMAGp998J23159	1.29	1.12	*SKP1A*	S-phase kinase-associated protein 1A (p19A)
720	IMAGp998M21677	1.29	1.12	*GFM1*	G elongation factor, mitochondrial 1
721	RZPDp1096C1219D	1.29	1.08	*NECAP2*	NECAP endocytosis associated 2
722	IMAGp998N13411	1.29	1.17	*N/A*	Transcribed locus
723	RZPDp201G0728D	1.29	1.21	*AGPAT6*	1-acylglycerol-3-phosphate O-acyltransferase 6 (lysophosphatidic acid acyltransferase, zeta)
724	IMAGp998O10119	1.29	1.18	*VCP*	Valosin-containing protein
725	RZPDp202H104D	1.29	1.24	*LOC388588*	Hypothetical gene supported by BC035379; BC042129
726	IMAGp998L235809	1.29	1.15	*N/A*	Transcribed locus
727	RZPDp201H0515D	1.29	1.22	*CYP2W1*	Cytochrome P450, family 2, subfamily W, polypeptide 1
728	IMAGp998I05318	1.29	1.10	*CAPNS1*	Calpain, small subunit 1
729	IMAGp998P24313	1.29	1.17	*COX8A*	Cytochrome c oxidase subunit 8A (ubiquitous)
730	IMAGp998O191853	1.29	1.08	*SMURF2*	SMAD specific E3 ubiquitin protein ligase 2
731	IMAGp998H0917	1.52	1.07	*ASNS*	Asparagine synthetase
732	RZPDp1096F0115D	1.52	1.18	*SOX5*	SRY (sex determining region Y)-box 5
733	IMAGp998M11663	1.52	1.20	*RPS23*	Ribosomal protein S23
734	RZPDp201A0820D	1.52	1.20	*TM9SF2*	Transmembrane 9 superfamily member 2
735	IMAGp998G0777	1.52	1.19	*NHP2L1*	NHP2 non-histone chromosome protein 2-like 1 (S. cerevisiae)
736	IMAGp998P02158	1.52	1.18	*N/A*	Full-length cDNA clone CS0DC012YG05 of Neuroblastoma Cot 25-normalized of Homo sapiens (human)
737	RZPDp202B053D	1.52	1.10	*C14orf131*	Chromosome 14 open reading frame 131
738	RZPDp202C127D	1.52	1.15	*CYBASC3*	Cytochrome b, ascorbate dependent 3
739	IMAGp998L13639	1.52	1.12	*VPS54*	Vacuolar protein sorting 54 homolog (S. cerevisiae)
740	IMAGp998A05327	1.52	1.12	*RUNX1T1*	Runt-related transcription factor 1; translocated to, 1 (cyclin D-related)
741	IMAGp998G10171	1.52	1.18	*LOC389203*	Hypothetical gene supported by BC032431
742	IMAGp998P19160	1.52	1.11	*EIF4G1*	Eukaryotic translation initiation factor 4 gamma, 1
743	IMAGp998K23891	1.52	1.09	*N/A*	Transcribed locus
744	IMAGp998D1383	1.52	1.11	*SLC12A7*	Solute carrier family 12 (potassium/chloride transporters), member 7
745	IMAGp998P03362	1.52	1.09	*N/A*	MRNA; cDNA DKFZp686D22106 (from clone DKFZp686D22106)
746	IMAGp998H081935	1.52	1.11	*RC3H2*	Ring finger and CCCH-type zinc finger domains 2
747	IMAGp998C24653	1.52	1.12	*SEPT6*	Septin 6
748	IMAGp998M191743	1.52	1.12	*N/A*	Data not found
749	IMAGp998M051198	1.52	1.20	*N/A*	Transcribed locus
750	RZPDp202C016D	1.52	1.08	*TUBG2*	Tubulin, gamma 2
751	IMAGp998J12268	1.52	1.15	*PEX7*	Peroxisomal biogenesis factor 7
752	IMAGp998E241118	1.52	1.17	*SNHG6*	Small nucleolar RNA host gene (non-protein coding) 6
753	IMAGp998F19151	1.52	1.37	*N/A*	Transcribed locus
754	IMAGp998N2273	1.52	1.10	*TERF1*	Telomeric repeat binding factor (NIMA-interacting) 1
755	IMAGp998I16620	1.52	1.37	*P15RS*	Hypothetical protein FLJ10656
756	RZPDp202E022D	1.52	1.13	*ZNF508*	Zinc finger protein 508
757	IMAGp998M15388	1.52	1.12	*SPCS2*	Signal peptidase complex subunit 2 homolog (S. cerevisiae)
758	RZPDp202F056D	1.52	1.14	*HLA-F*	Major histocompatibility complex, class I, F
759	IMAGp998M214158	1.52	1.06	*CCDC67*	Coiled-coil domain containing 67
760	RZPDp201B0136D	1.52	1.17	*VDP*	Vesicle docking protein p115
761	IMAGp998B1671	1.52	1.10	*PSMC6*	Proteasome (prosome, macropain) 26S subunit, ATPase, 6
762	IMAGp998O244248	1.52	1.10	*N/A*	Data not found
763	RZPDp202D018D	1.52	1.11	*EFTUD2*	Elongation factor Tu GTP binding domain containing 2
764	RZPDp202D029D	1.52	1.18	*RPS29*	Ribosomal protein S29
765	IMAGp998M062035	1.52	1.09	*N/A*	Transcribed locus
766	IMAGp998I13280	1.52	1.12	*PCCB*	Propionyl Coenzyme A carboxylase, beta polypeptide
767	RZPDp1096H106D	1.52	1.12	*HMCN2*	Hemicentin 2
768	IMAGp998B14686	1.52	1.55	*DTWD1*	DTW domain containing 1
769	IMAGp998F056110	1.52	1.11	*HEATR5B*	HEAT repeat containing 5B
770	IMAGp998D09578	1.52	1.11	*TOLLIP*	Toll interacting protein
771	RZPDp201C0120D	1.52	1.30	*LOC441383*	Hypothetical gene supported by AF086559; BC065734
772	RZPDp201G0517D	1.52	1.09	*PORCN*	Porcupine homolog (Drosophila)
773	IMAGp998C12213	1.52	1.14	*MBNL1*	Muscleblind-like (Drosophila)
774	RZPDp1096A0119D	1.52	1.18	*FOSL1*	FOS-like antigen 1
775	RZPDp202F076D	1.52	1.08	*LOXL2*	Lysyl oxidase-like 2
776	IMAGp998J033941	1.52	1.19	*BHMT2*	Betaine-homocysteine methyltransferase 2
777	IMAGp998O13658	1.52	1.08	*PRPF38B*	PRP38 pre-mRNA processing factor 38 (yeast) domain containing B
778	RZPDp201E123D	1.52	1.08	*DAD1*	Defender against cell death 1
779	IMAGp998C16692	1.52	1.10	*C15orf41*	Chromosome 15 open reading frame 41
780	RZPDp1096A102D	1.52	1.13	*PPME1*	Protein phosphatase methylesterase 1
781	IMAGp998F22161	1.86	1.13	*ENO2*	Enolase 2 (gamma, neuronal)
782	RZPDp1096F071D	1.86	1.11	*DSTN*	Destrin (actin depolymerizing factor)
783	RZPDp201H0828D	1.86	1.31	*HLA-DRB1*	Major histocompatibility complex, class II, DR beta 1
784	IMAGp998K06295	1.86	1.14	*FXYD6*	FXYD domain containing ion transport regulator 6
785	IMAGp998F025462	1.86	1.19	*TP53INP1*	Tumor protein p53 inducible nuclear protein 1
786	RZPDp202G064D	1.86	1.12	*TCEAL3*	Transcription elongation factor A (SII)-like 3
787	RZPDp1096E0614D	1.86	1.11	*NFIA*	Nuclear factor I/A
788	IMAGp998N19565	1.86	1.12	*PKNOX1*	PBX/knotted 1 homeobox 1
789	RZPDp1096C106D	1.86	1.13	*NMNAT2*	Nicotinamide nucleotide adenylyltransferase 2
790	RZPDp201D1128D	1.86	1.11	*TBCD*	Tubulin folding cofactor D
791	RZPDp201C1035D	1.86	1.08	*ZFX*	Zinc finger protein, X-linked
792	IMAGp998O22613	1.86	1.12	*IK*	IK cytokine, down-regulator of HLA II
793	RZPDp201D0228D	1.86	1.17	*ITGBL1*	Integrin, beta-like 1 (with EGF-like repeat domains)
794	IMAGp998O091789	1.86	1.16	*ZNF91*	Zinc finger protein 91
795	IMAGp998P13696	1.86	1.17	*TSTA3*	Tissue specific transplantation antigen P35B
796	RZPDp1096C033D	1.86	1.12	*GARNL1*	GTPase activating Rap/RanGAP domain-like 1
797	IMAGp998O13422	1.86	1.19	*SEC61B*	Sec61 beta subunit
798	IMAGp998N12248	1.86	1.13	*AFG3L2*	AFG3 ATPase family gene 3-like 2 (yeast)
799	RZPDp201F1233D	1.86	1.14	*YTHDF2*	YTH domain family, member 2
800	RZPDp1096A038D	1.86	1.09	*PTP4A2*	Protein tyrosine phosphatase type IVA, member 2
801	IMAGp998A20599	1.86	1.10	*SRGAP2*	SLIT-ROBO Rho GTPase activating protein 2
802	IMAGp998B121898	1.86	1.17	*IFITM1*	Interferon induced transmembrane protein 1 (9-27)
803	IMAGp998J091779	1.86	1.19	*RPL6*	Ribosomal protein L6
804	RZPDp201A0618D	1.86	1.14	*ITGA6*	Integrin, alpha 6
805	IMAGp998A051793	1.86	1.20	*SRP19*	Signal recognition particle 19kDa
806	RZPDp202B124D	1.86	1.16	*FASLG*	Fas ligand (TNF superfamily, member 6)
807	RZPDp202C065D	1.86	1.10	*CHAF1A*	Chromatin assembly factor 1, subunit A (p150)
808	RZPDp1096G127D	1.86	1.11	*EIF4G1*	Eukaryotic translation initiation factor 4 gamma, 1
809	IMAGp998J09171	1.86	1.30	*DAG1*	Dystroglycan 1 (dystrophin-associated glycoprotein 1)
810	RZPDp201A122D	1.86	1.13	*SNRPE*	Small nuclear ribonucleoprotein polypeptide E

811	IMAGp998P07417	1.86	1.17	*N/A*	Transcribed locus
812	IMAGp998N061016	1.86	1.16	*CG012*	Hypothetical gene CG012
813	IMAGp998G171864	1.86	1.14	*ARHGAP12*	Rho GTPase activating protein 12
814	IMAGp998I0897	1.86	1.13	*COX17*	COX17 cytochrome c oxidase assembly homolog (S. cerevisiae)
815	IMAGp998G086101	1.86	1.19	*STXBP6*	Syntaxin binding protein 6 (amisyn)
816	IMAGp998K16230	1.86	1.12	*CD47*	CD47 molecule
817	RZPDp201F1029D	1.86	1.11	*UBAP2*	Ubiquitin associated protein 2
818	RZPDp1096A0716D	1.86	1.09	*MAGEA3*	Melanoma antigen family A, 3
819	IMAGp998C03614	1.86	1.15	*USP9Y*	Ubiquitin specific peptidase 9, Y-linked (fat facets-like, Drosophila)
820	IMAGp998B031942	1.86	1.10	*ZMYM2*	Zinc finger, MYM-type 2
821	IMAGp998M1678	1.86	1.32	*CD74*	CD74 molecule, major histocompatibility complex, class II invariant chain
822	IMAGp998A042227	1.86	1.44	*AIFM3*	Apoptosis-inducing factor, mitochondrion-associated, 3
823	IMAGp998E041784	1.86	1.32	*N/A*	CDNA clone IMAGE:4831354
824	IMAGp998D07222	1.86	1.17	*ATP6V1G1*	ATPase, H+ transporting, lysosomal 13kDa, V1 subunit G1
825	IMAGp998O23564	1.86	1.22	*ARFGAP3*	ADP-ribosylation factor GTPase activating protein 3
826	IMAGp998E041200	1.86	1.20	*HOXA10*	Homeobox A10
827	IMAGp998E11285	1.86	1.10	*SLC39A1*	Solute carrier family 39 (zinc transporter), member 1
828	RZPDp1096E125D	1.86	1.13	*ARHGAP12*	Rho GTPase activating protein 12
829	RZPDp201F0217D	1.86	1.22	*NBPF15*	Neuroblastoma breakpoint family, member 15
830	IMAGp998F051165	1.86	1.06	*PHLPPL*	PH domain and leucine rich repeat protein phosphatase-like
831	IMAGp998M21367	2.10	1.17	*N/A*	Data not found
832	IMAGp998G164063	2.10	1.69	*N/A*	Data not found
833	IMAGp998D07674	2.10	1.11	*C19orf22*	Chromosome 19 open reading frame 22
834	IMAGp998N14571	2.10	1.08	*KIAA1143*	KIAA1143
835	IMAGp998J245775	2.10	1.07	*N/A*	Transcribed locus
836	IMAGp998I033161	2.10	1.33	*N/A*	Data not found
837	IMAGp998I181007	2.10	1.13	*PIGS*	Phosphatidylinositol glycan anchor biosynthesis, class S
838	IMAGp998M06255	2.10	1.13	*ITGA11*	Integrin, alpha 11
839	IMAGp998F02324	2.10	1.14	*XPO6*	Exportin 6
840	IMAGp998A18791	2.10	1.17	*PUM1*	Pumilio homolog 1 (Drosophila)
841	RZPDp201E028D	2.10	1.13	*SCN4A*	Sodium channel, voltage-gated, type IV, alpha subunit
842	IMAGp998E20154	2.10	1.14	*ATP6V1E1*	ATPase, H+ transporting, lysosomal 31kDa, V1 subunit E1
843	IMAGp998L02227	2.10	1.14	*EEF1B2*	Eukaryotic translation elongation factor 1 beta 2
844	IMAGp998P06139	2.10	1.18	*ZDHHC21*	Zinc finger, DHHC-type containing 21
845	IMAGp998O171776	2.10	1.19	*LGALS3BP*	Lectin, galactoside-binding, soluble, 3 binding protein
846	RZPDp202B113D	2.10	1.11	*FIBCD1*	Fibrinogen C domain containing 1
847	RZPDp1096C0620D	2.10	1.15	*GJA5*	Gap junction protein, alpha 5, 40kDa
848	IMAGp998B201196	2.10	1.11	*FKBP10*	FK506 binding protein 10, 65 kDa
849	IMAGp998D08129	2.10	1.15	*THRB*	Thyroid hormone receptor, beta (erythroblastic leukemia viral (v-erb-a) oncogene homolog 2, avian)
850	IMAGp998H211852	2.10	1.17	*EIF2C2*	Eukaryotic translation initiation factor 2C, 2
851	IMAGp998L20376	2.10	1.08	*WNK1*	WNK lysine deficient protein kinase 1
852	IMAGp998B03399	2.10	1.06	*PARP4*	Poly (ADP-ribose) polymerase family, member 4
853	IMAGp998N102000	2.10	1.10	*FER1L3*	Fer-1-like 3, myoferlin (C. elegans)
854	IMAGp998P06602	2.10	1.09	*TANK*	TRAF family member-associated NFKB activator
855	RZPDp202F079D	2.10	1.07	*RLN1*	Relaxin 1
856	IMAGp998H164472	2.10	1.17	*AASDH*	2-aminoadipic 6-semialdehyde dehydrogenase
857	IMAGp998P025201	2.10	1.07	*TM4SF19*	Transmembrane 4 L six family member 19
858	IMAGp998N07281	2.10	1.09	*SF3B2*	Splicing factor 3b, subunit 2, 145kDa
859	IMAGp998N02785	2.10	1.19	*PARK7*	Parkinson disease (autosomal recessive, early onset) 7
860	IMAGp998G18368	2.10	1.08	*ABHD12*	Abhydrolase domain containing 12
861	IMAGp998I194413	2.10	1.13	*N/A*	Transcribed locus
862	IMAGp998K12649	2.10	1.12	*N/A*	Full length insert cDNA clone ZA04F06
863	IMAGp998M07142	2.10	1.08	*TARDBP*	TAR DNA binding protein
864	IMAGp998A17525	2.10	1.08	*SMAP1L*	Stromal membrane-associated protein 1-like
865	IMAGp998O16420	2.10	1.09	*UBN1*	Ubinuclein 1
866	IMAGp998M23234	2.10	1.15	*EDEM3*	ER degradation enhancer, mannosidase alpha-like 3
867	IMAGp998P16171	2.10	1.08	*LRRN1*	Leucine rich repeat neuronal 1
868	IMAGp998F09192	2.10	1.11	*NCKAP1*	NCK-associated protein 1
869	IMAGp998A096083	2.10	1.14	*RNF121*	Ring finger protein 121
870	IMAGp998B245029	2.43	1.16	*N/A*	Transcribed locus
871	RZPDp1096E0613D	2.43	1.08	*KRTCAP3*	Keratinocyte associated protein 3
872	IMAGp998P04273	2.43	1.10	*GNAI2*	Guanine nucleotide binding protein (G protein), alpha inhibiting activity polypeptide 2
873	IMAGp998F18342	2.43	1.26	*NEFH*	Neurofilament, heavy polypeptide 200kDa
874	IMAGp998P224409	2.43	1.09	*N/A*	Transcribed locus
875	IMAGp998J125933	2.43	1.31	*N/A*	Transcribed locus, weakly similar to XP_512578.2 similar to LSM14 homolog A isoform 2 [Pan troglodytes]
876	IMAGp998L211112	2.43	1.25	*FH*	Fumarate hydratase
877	RZPDp201B112D	2.43	1.17	*VDAC3*	Voltage-dependent anion channel 3
878	IMAGp998G20195	2.43	1.30	*ZNF131*	Zinc finger protein 131
879	IMAGp998O20169	2.43	1.10	*RHOG*	Ras homolog gene family, member G (rho G)
880	RZPDp202A083D	2.43	1.13	*ALS2CR7*	Amyotrophic lateral sclerosis 2 (juvenile) chromosome region, candidate 7
881	RZPDp201F1035D	2.43	1.15	*SAR1A*	SAR1 gene homolog A (S. cerevisiae)
882	IMAGp998N10167	2.43	1.10	*N/A*	MRNA; cDNA DKFZp686L15210 (from clone DKFZp686L15210)
883	RZPDp1096B116D	2.43	1.54	*C1orf183*	Chromosome 1 open reading frame 183
884	RZPDp201B0632D	2.43	1.12	*PTPN12*	Protein tyrosine phosphatase, non-receptor type 12
885	IMAGp998E082758	2.43	1.18	*N/A*	MRNA; cDNA DKFZp686E22185 (from clone DKFZp686E22185)
886	IMAGp998B18399	2.43	1.18	*C22orf28*	Chromosome 22 open reading frame 28
887	IMAGp998B04155	2.43	1.10	*GLIS2*	GLIS family zinc finger 2
888	RZPDp201G0815D	2.43	1.13	*ATP8B1*	ATPase, Class I, type 8B, member 1
889	IMAGp998B0577	2.43	1.12	*WARS*	Tryptophanyl-tRNA synthetase
890	IMAGp998D23161	2.43	1.12	*N/A*	Transcribed locus
891	IMAGp998C21178	2.43	1.11	*RAB2A*	RAB2A, member RAS oncogene family
892	IMAGp998P0376	2.43	1.10	*MARCH3*	Membrane-associated ring finger (C3HC4) 3
893	IMAGp998N20188	2.43	1.22	*SFRS5*	Splicing factor, arginine/serine-rich 5
894	IMAGp998B22668	2.43	1.17	*VIM*	Vimentin
895	IMAGp998A19870	2.43	1.11	*PML*	Promyelocytic leukemia
896	IMAGp998D16285	2.43	1.12	*FBXO7*	F-box protein 7
897	RZPDp202F114D	2.43	1.09	*SLC6A8*	Solute carrier family 6 (neurotransmitter transporter, creatine), member 8
898	IMAGp998O021822	2.43	1.09	*PSCD2*	Pleckstrin homology, Sec7 and coiled-coil domains 2 (cytohesin-2)
899	IMAGp998L12154	2.43	1.18	*ECHS1*	Enoyl Coenzyme A hydratase, short chain, 1, mitochondrial
900	IMAGp998B22154	2.82	1.49	*LZTS1*	Leucine zipper, putative tumor suppressor 1
901	RZPDp201B1136D	2.82	1.21	*RERE*	Arginine-glutamic acid dipeptide (RE) repeats
902	RZPDp201C051D	2.82	1.14	*DHFR*	Dihydrofolate reductase
903	IMAGp998C23585	2.82	1.16	*PCBD1*	Pterin-4 alpha-carbinolamine dehydratase/dimerization cofactor of hepatocyte nuclear factor 1 alpha (TCF1)
904	IMAGp998I04668	2.82	1.17	*ATP6V1D*	ATPase, H+ transporting, lysosomal 34kDa, V1 subunit D
905	RZPDp201H066D	2.82	1.15	*MMP25*	Matrix metallopeptidase 25
906	IMAGp998J105404	2.82	1.16	*N/A*	Transcribed locus
907	RZPDp201E015D	2.82	1.06	*IL1RN*	Interleukin 1 receptor antagonist
908	RZPDp202F074D	2.82	1.14	*AMOT*	Angiomotin
909	RZPDp1096A0118D	2.82	1.11	*YLPM1*	YLP motif containing 1
910	RZPDp201E0228D	2.82	1.14	*CCDC9*	Coiled-coil domain containing 9
911	IMAGp998C17370	2.82	1.15	*N-PAC*	Cytokine-like nuclear factor n-pac
912	RZPDp201B1032D	2.82	1.09	*TPM3*	Tropomyosin 3
913	IMAGp998O03227	2.82	1.09	*FAM126B*	Family with sequence similarity 126, member B
914	IMAGp998N21461	2.82	1.13	*BGN*	Biglycan
915	IMAGp998H241113	2.82	1.10	*REXO2*	REX2, RNA exonuclease 2 homolog (S. cerevisiae)
916	IMAGp998P15590	2.82	1.12	*GTF2I*	General transcription factor II, i
917	IMAGp998A22186	2.82	1.15	*ARFIP1*	ADP-ribosylation factor interacting protein 1 (arfaptin 1)
918	IMAGp998K1579	2.82	1.19	*SH3YL1*	SH3 domain containing, Ysc84-like 1 (S. cerevisiae)
919	IMAGp998M20402	2.82	1.20	*HSD17B10*	Hydroxysteroid (17-beta) dehydrogenase 10
920	RZPDp201C0535D	2.82	1.15	*DC2*	DC2 protein
921	RZPDp202A118D	2.82	1.08	*LMNB2*	Lamin B2
922	IMAGp998K10590	2.82	1.10	*ZXDC*	ZXD family zinc finger C
923	IMAGp998F171793	2.82	1.08	*ACVR1B*	Activin A receptor, type IB
924	IMAGp998C181781	2.82	1.09	*TMEM109*	Transmembrane protein 109
925	IMAGp998P19276	2.82	1.13	*N/A*	Transcribed locus
926	RZPDp201G1129D	2.82	1.17	*FLNB*	Filamin B, beta (actin binding protein 278)
927	IMAGp998O03269	2.82	1.20	*JARID1A*	Jumonji, AT rich interactive domain 1A
928	IMAGp998D074417	2.82	1.18	*N/A*	Transcribed locus
929	IMAGp998E05373	2.82	1.11	*N/A*	CDNA: FLJ21199 fis, clone COL00235
930	IMAGp998H033811	2.82	1.11	*N/A*	Transcribed locus
931	IMAGp998P2389	2.82	1.08	*C21orf25*	Chromosome 21 open reading frame 25
932	IMAGp998B07284	2.82	1.17	*FADS2*	Fatty acid desaturase 2
933	IMAGp998O17665	2.82	1.20	*SESN1*	Sestrin 1
934	IMAGp998J23587	2.82	1.09	*SMEK1*	SMEK homolog 1, suppressor of mek1 (Dictyostelium)
935	RZPDp1096E116D	2.82	1.13	*CYP24A1*	Cytochrome P450, family 24, subfamily A, polypeptide 1
936	IMAGp998C06237	2.82	1.11	*AEBP1*	AE binding protein 1
937	IMAGp998C13144	2.82	1.13	*COX11*	COX11 homolog, cytochrome c oxidase assembly protein (yeast)
938	RZPDp201D0928D	2.82	1.23	*RGS1*	Regulator of G-protein signalling 1
939	RZPDp201F1228D	2.82	1.12	*RPL34*	Ribosomal protein L34
940	IMAGp998D20518	2.82	1.20	*SAT1*	Spermidine/spermine N1-acetyltransferase 1
941	IMAGp998P19155	2.82	1.18	*NRG3*	Neuregulin 3
942	RZPDp201A0336D	2.82	1.15	*PPP1R3F*	Protein phosphatase 1, regulatory (inhibitor) subunit 3F
943	IMAGp998K18564	2.82	1.06	*TOP2A*	Topoisomerase (DNA) II alpha 170kDa
944	RZPDp202H027D	2.82	1.16	*SHC1*	SHC (Src homology 2 domain containing) transforming protein 1
945	RZPDp1096C011D	2.82	1.09	*ARHGAP21*	Rho GTPase activating protein 21

946	RZPDp201D097D	2.82	1.19	*FAM38B*	Family with sequence similarity 38, member B
947	RZPDp201G1235D	2.82	1.12	*PSMC6*	Proteasome (prosome, macropain) 26S subunit, ATPase, 6
948	RZPDp1096D116D	2.82	1.08	*GLI1*	Glioma-associated oncogene homolog 1 (zinc finger protein)
949	RZPDp202D081D	3.25	1.10	*SLC6A8*	Solute carrier family 6 (neurotransmitter transporter, creatine), member 8
950	RZPDp202F087D	3.25	1.09	*FAM50B*	Family with sequence similarity 50, member B
951	RZPDp1096B0316D	3.25	1.18	*SYNCRIP*	Synaptotagmin binding, cytoplasmic RNA interacting protein
952	IMAGp998F13667	3.25	1.12	*N/A*	Transcribed locus
953	IMAGp998D175735	3.25	1.12	*N/A*	Transcribed locus
954	IMAGp998E205760	3.25	1.12	*N/A*	Transcribed locus
955	RZPDp202H084D	3.25	1.08	*CAMTA2*	Calmodulin binding transcription activator 2
956	IMAGp998A11271	3.25	1.11	*SNUPN*	Snurportin 1
957	IMAGp998C23160	3.25	1.13	*LSM2*	LSM2 homolog, U6 small nuclear RNA associated (S. cerevisiae)
958	IMAGp998P241904	3.25	1.15	*BTBD6*	BTB (POZ) domain containing 6
959	IMAGp998M141825	3.25	1.24	*SYBL1*	Synaptobrevin-like 1
960	RZPDp201B1220D	3.25	1.07	*SNX1*	Sorting nexin 1
961	IMAGp998B092011	3.25	1.07	*N/A*	Full length insert cDNA clone ZD86A03
962	IMAGp998D112036	3.25	1.09	*CBWD1*	COBW domain containing 1
963	IMAGp998M10277	3.25	1.14	*N/A*	CDNA clone IMAGE:4821984
964	IMAGp998G181857	3.25	1.07	*C22orf16*	Chromosome 22 open reading frame 16
965	RZPDp202B115D	3.25	1.31	*ZNF219*	Zinc finger protein 219
966	IMAGp998P135097	3.25	1.49	*N/A*	Full-length cDNA clone CL0BB004ZB12 of Neuroblastoma of Homo sapiens (human)
967	RZPDp201D0836D	3.25	1.10	*SPCS1*	Signal peptidase complex subunit 1 homolog (S. cerevisiae)
968	IMAGp998L162227	3.25	1.14	*LDHA*	Lactate dehydrogenase A
969	IMAGp998B16179	3.25	1.07	*SRI*	Sorcin
970	RZPDp201E064D	3.25	1.12	*CD109*	CD109 molecule
971	IMAGp998P24615	3.25	1.09	*KIAA0232*	KIAA0232 gene product
972	IMAGp998C081775	3.25	1.15	*SNRPB*	Small nuclear ribonucleoprotein polypeptides B and B1
973	IMAGp998C24650	3.25	1.08	*MRPS23*	Mitochondrial ribosomal protein S23
974	IMAGp998C242036	3.25	1.22	*PPP1R12A*	Protein phosphatase 1, regulatory (inhibitor) subunit 12A
975	IMAGp998N231934	3.25	1.08	*ZNF655*	Zinc finger protein 655
976	IMAGp998A171905	3.25	1.08	*CHMP2B*	Chromatin modifying protein 2B
977	IMAGp998H161864	3.25	1.08	*OSTalpha*	Organic solute transporter alpha
978	RZPDp202E056D	3.25	1.16	*RPL7*	Ribosomal protein L7
979	RZPDp201C0226D	3.25	1.20	*LOC400987*	Similar to ankyrin repeat domain 36
980	RZPDp201F0434D	3.25	1.16	*IDH2*	Isocitrate dehydrogenase 2 (NADP+), mitochondrial
981	RZPDp202D036D	3.25	1.12	*MARCH4*	Membrane-associated ring finger (C3HC4) 4
982	IMAGp998M16131	3.25	1.13	*N/A*	Data not found
983	IMAGp998I24285	3.25	1.09	*EIF3S7*	Eukaryotic translation initiation factor 3, subunit 7 zeta, 66/67kDa
984	RZPDp201D0535D	3.25	1.10	*MORF4L2*	Mortality factor 4 like 2
985	RZPDp202F0210D	3.25	1.07	*DAD1*	Defender against cell death 1
986	IMAGp998M051158	3.25	1.75	*N/A*	Data not found
987	RZPDp1096E067D	3.25	1.13	*SRP9*	Signal recognition particle 9kDa
988	RZPDp1096D0520D	3.25	1.33	*SERBP1*	SERPINE1 mRNA binding protein 1
989	IMAGp998A23531	3.25	1.12	*MDH1*	Malate dehydrogenase 1, NAD (soluble)
990	IMAGp998L14177	3.25	1.07	*DDX3X*	DEAD (Asp-Glu-Ala-Asp) box polypeptide 3, X-linked
991	IMAGp998J2417	3.25	1.08	*N/A*	Transcribed locus
992	IMAGp998J22323	3.25	1.16	*ABI2*	Abl interactor 2
993	IMAGp998G17783	3.78	1.08	*SERF1A*	Small EDRK-rich factor 1A (telomeric)
994	IMAGp998A081791	3.78	1.10	*ANKRD12*	Ankyrin repeat domain 12
995	IMAGp998C20738	3.78	1.09	*SERPINE1*	Serpin peptidase inhibitor, clade E (nexin, plasminogen activator inhibitor type 1), member 1
996	IMAGp998I22653	3.78	1.08	*TMEM69*	Transmembrane protein 69
997	IMAGp998O221933	3.78	1.16	*N/A*	Transcribed locus
998	RZPDp202D048D	3.78	1.27	*SAFB*	Scaffold attachment factor B
999	IMAGp998C16174	3.78	1.14	*NDRG4*	NDRG family member 4
1000	RZPDp202A031D	3.78	1.12	*ATP2B3*	ATPase, Ca++ transporting, plasma membrane 3
1001	IMAGp998E21158	3.78	1.13	*IPO8*	Importin 8
1002	RZPDp1096A017D	3.78	1.15	*CUGBP1*	CUG triplet repeat, RNA binding protein 1
1003	RZPDp201H0619D	3.78	1.06	*DDX46*	DEAD (Asp-Glu-Ala-Asp) box polypeptide 46
1004	RZPDp202F037D	3.78	1.06	*PLXNA3*	Plexin A3
1005	IMAGp998E06525	3.78	1.12	*MAP2K1IP1*	Mitogen-activated protein kinase kinase 1 interacting protein 1
1006	RZPDp1096F0315D	3.78	1.12	*C20orf119*	Chromosome 20 open reading frame 119
1007	IMAGp998F135401	3.78	1.11	*N/A*	Transcribed locus
1008	IMAGp998O19735	3.78	1.08	*ADIPOR2*	Adiponectin receptor 2
1009	IMAGp998O13601	3.78	1.14	*N/A*	Data not found
1010	IMAGp998M23148	3.78	1.06	*OSTM1*	Osteopetrosis associated transmembrane protein 1
1011	IMAGp998K164731	3.78	1.08	*PROC*	Protein C (inactivator of coagulation factors Va and VIIIa)
1012	IMAGp998G06268	3.78	1.16	*GNL3*	Guanine nucleotide binding protein-like 3 (nucleolar)
1013	IMAGp998I11171	3.78	1.09	*CHD8*	Chromodomain helicase DNA binding protein 8
1014	RZPDp1096A072D	3.78	1.20	*DNAJA4*	DnaJ (Hsp40) homolog, subfamily A, member 4
1015	IMAGp998H03169	3.78	1.07	*LSM14A*	LSM14A, SCD6 homolog A (S. cerevisiae)
1016	IMAGp998C211112	3.78	1.12	*ST8SIA4*	ST8 alpha-N-acetyl-neuraminide alpha-2,8-sialyltransferase 4
1017	IMAGp998K031092	3.78	1.12	*N/A*	Transcribed locus
1018	RZPDp1096G046D	3.78	1.08	*SCN2A*	Sodium channel, voltage-gated, type II, alpha subunit
1019	RZPDp202D117D	3.78	1.07	*CST7*	Cystatin F (leukocystatin)
1020	IMAGp998L16153	3.78	1.12	*DNAJB6*	DnaJ (Hsp40) homolog, subfamily B, member 6
1021	RZPDp201B1028D	3.78	1.11	*TUBG1*	Tubulin, gamma 1
1022	RZPDp202C0710D	3.78	1.06	*KIAA0423*	KIAA0423
1023	IMAGp998C11653	3.78	1.13	*SNX5*	Sorting nexin 5
1024	IMAGp998K0492	3.78	1.17	*HOOK1*	Hook homolog 1 (Drosophila)
1025	IMAGp998I154380	3.78	1.15	*COBLL1*	COBL-like 1
1026	RZPDp201G0615D	3.78	1.17	*PLEKHN1*	Pleckstrin homology domain containing, family N member 1
1027	RZPDp201B115D	3.78	1.08	*C17orf85*	Chromosome 17 open reading frame 85
1028	IMAGp998L03142	3.78	1.08	*ADSL*	Adenylosuccinate lyase
1029	RZPDp201A0713D	3.78	1.08	*NINJ2*	Ninjurin 2
1030	IMAGp998I18168	3.78	1.16	*ARG2*	Arginase, type II
1031	IMAGp998A141779	3.78	1.18	*SFRP4*	Secreted frizzled-related protein 4
1032	RZPDp202D074D	3.78	1.08	*FLJ20186*	Hypothetical protein FLJ20186
1033	IMAGp998N24149	3.78	1.07	*N/A*	CDNA clone IMAGE:3030163
1034	RZPDp1096D0713D	3.78	1.11	*DKFZp547K054*	Hypothetical protein DKFZp547K054
1035	IMAGp998E11121	3.78	1.15	*PAIP2*	Poly(A) binding protein interacting protein 2
1036	IMAGp998A0418	3.78	1.11	*VDAC2*	Voltage-dependent anion channel 2
1037	RZPDp1096B042D	3.78	1.11	*RXRA*	Retinoid X receptor, alpha
1038	IMAGp998J10689	3.78	1.60	*EEF1E1*	Eukaryotic translation elongation factor 1 epsilon 1
1039	IMAGp998O24113	3.78	1.06	*EIF4EBP2*	Eukaryotic translation initiation factor 4E binding protein 2
1040	IMAGp998J174274	3.78	1.07	*INVS*	Inversin
1041	IMAGp998D01157	3.78	1.08	*ZNF289*	Zinc finger protein 289, ID1 regulated
1042	IMAGp998N12138	3.78	1.11	*MXI1*	MAX interactor 1
1043	RZPDp202C068D	3.78	1.07	*SERGEF*	Secretion regulating guanine nucleotide exchange factor
1044	RZPDp202D1010D	3.78	1.07	*SUB1*	SUB1 homolog (S. cerevisiae)
1045	IMAGp998H23406	3.78	1.15	*ATP11B*	ATPase, Class VI, type 11B
1046	RZPDp1096F095D	3.78	1.10	*IRX6*	Iroquois homeobox protein 6
1047	IMAGp998B20616	3.78	1.17	*TMEM87A*	Transmembrane protein 87A
1048	RZPDp202H098D	3.78	1.10	*CLSTN3*	Calsyntenin 3
1049	IMAGp998F065797	4.29	1.12	*ZNF616*	Zinc finger protein 616
1050	RZPDp1096G053D	4.29	1.09	*IL32*	Interleukin 32
1051	RZPDp202C093D	4.29	1.11	*FAM108A1*	Family with sequence similarity 108, member A1
1052	IMAGp998I0317	4.29	1.15	*CHST1*	Carbohydrate (keratan sulfate Gal-6) sulfotransferase 1
1053	IMAGp998F055668	4.29	1.08	*ANP32E*	Acidic (leucine-rich) nuclear phosphoprotein 32 family, member E
1054	RZPDp201G0530D	4.29	1.10	*HMGB3*	High-mobility group box 3
1055	IMAGp998A154909	4.29	1.29	*N/A*	Data not found
1056	IMAGp998J05329	4.29	1.14	*NDUFB9*	NADH dehydrogenase (ubiquinone) 1 beta subcomplex, 9, 22kDa
1057	IMAGp998H233208	4.29	1.69	*N/A*	Transcribed locus
1058	IMAGp998J055808	4.29	1.80	*N/A*	Transcribed locus
1059	IMAGp998C14273	4.29	1.09	*LOC339400*	Hypothetical protein LOC339400
1060	IMAGp998P231815	4.29	1.15	*MX1*	Myxovirus (influenza virus) resistance 1, interferon-inducible protein p78 (mouse)
1061	IMAGp998F241824	4.29	1.05	*TMEM16A*	Transmembrane protein 16A
1062	RZPDp201A1030D	4.29	1.14	*NAT5*	N-acetyltransferase 5
1063	IMAGp998I14117	4.29	1.12	*GRN*	Granulin
1064	IMAGp998B081784	4.29	1.31	*N/A*	Transcribed locus, strongly similar to XP_001167650.1 hypothetical protein [Pan troglodytes]
1065	IMAGp998D11523	4.29	1.25	*ASS1*	Argininosuccinate synthetase 1
1066	IMAGp998M224416	4.29	1.09	*N/A*	Transcribed locus
1067	IMAGp998C03131	4.29	1.05	*RAI14*	Retinoic acid induced 14
1068	IMAGp998D192344	4.29	1.13	*PPP1R9A*	Protein phosphatase 1, regulatory (inhibitor) subunit 9A
1069	IMAGp998D06697	4.29	1.35	*BCL2L2*	BCL2-like 2
1070	IMAGp998J204057	4.29	1.20	*PRM2*	Protamine 2
1071	IMAGp998C08158	4.29	1.26	*PDLIM3*	PDZ and LIM domain 3
1072	RZPDp202A016D	4.29	1.08	*ANXA8*	Annexin A8
1073	RZPDp1096G055D	4.29	1.19	*OSMR*	Oncostatin M receptor
1074	IMAGp998C2272	4.29	1.14	*N/A*	Transcribed locus
1075	IMAGp998N105213	4.29	1.14	*ZNF404*	Zinc finger protein 404
1076	IMAGp998J08128	4.29	1.09	*CCDC72*	Coiled-coil domain containing 72
1077	IMAGp998K07261	4.29	1.09	*C1orf9*	Chromosome 1 open reading frame 9
1078	IMAGp998C18172	4.29	1.21	*CHRAC1*	Chromatin accessibility complex 1
1079	IMAGp998N061965	4.29	1.23	*TBL1XR1*	Transducin (beta)-like 1X-linked receptor 1
1080	RZPDp201D0919D	4.29	1.09	*USP47*	Ubiquitin specific peptidase 47

1081	IMAGp998K232973	4.29	1.12	*UBE2J1*	Ubiquitin-conjugating enzyme E2, J1 (UBC6 homolog, yeast)
1082	IMAGp998L12593	4.29	1.11	*RNF41*	Ring finger protein 41
1083	RZPDp201B0336D	4.29	1.14	*TRIM28*	Tripartite motif-containing 28
1084	IMAGp998L10617	4.29	1.13	*SETD3*	SET domain containing 3
1085	IMAGp998I205925	4.29	1.12	*N/A*	Transcribed locus
1086	RZPDp201B1117D	4.29	1.07	*ZNF187*	Zinc finger protein 187
1087	IMAGp998B0474	4.29	1.09	*CDK4*	Cyclin-dependent kinase 4
1088	IMAGp998G15694	4.29	1.10	*SOD1*	Superoxide dismutase 1, soluble (amyotrophic lateral sclerosis 1 (adult))
1089	RZPDp201H0728D	4.29	1.26	*TRAF4*	TNF receptor-associated factor 4
1090	IMAGp998C101202	4.29	1.19	*NFIC*	Nuclear factor I/C (CCAAT-binding transcription factor)
1091	IMAGp998C021833	4.29	1.08	*HNRPUL1*	Heterogeneous nuclear ribonucleoprotein U-like 1
1092	IMAGp998C14658	4.29	1.10	*UGCG*	UDP-glucose ceramide glucosyltransferase
1093	IMAGp998G04277	4.71	1.10	*WDR47*	WD repeat domain 47
1094	RZPDp202F123D	4.71	1.08	*C14orf173*	Chromosome 14 open reading frame 173
1095	RZPDp1096B022D	4.71	1.18	*KPNB1*	Karyopherin (importin) beta 1
1096	IMAGp998J19734	4.71	1.08	*OPRS1*	Opioid receptor, sigma 1
1097	IMAGp998P095740	4.71	1.14	*N/A*	Transcribed locus
1098	RZPDp1096D0218D	4.71	1.26	*ZNF140*	Zinc finger protein 140
1099	IMAGp998D221205	4.71	1.07	*PDPK1*	3-phosphoinositide dependent protein kinase-1
1100	IMAGp998J01156	4.71	1.09	*NR2E1*	Nuclear receptor subfamily 2, group E, member 1
1101	RZPDp202C097D	4.71	1.09	*PTP4A2*	Protein tyrosine phosphatase type IVA, member 2
1102	IMAGp998G135688	4.71	1.15	*N/A*	Transcribed locus
1103	RZPDp202E047D	4.71	1.25	*STAT1*	Signal transducer and activator of transcription 1, 91kDa
1104	IMAGp998C144452	4.71	1.06	*N/A*	Transcribed locus
1105	IMAGp998B171780	4.71	1.15	*TFAP2C*	Transcription factor AP-2 gamma (activating enhancer binding protein 2 gamma)
1106	IMAGp998K14213	4.71	1.06	*DKFZp761E198*	DKFZp761E198 protein
1107	IMAGp998A24372	4.71	1.15	*SEC22C*	SEC22 vesicle trafficking protein homolog C (S. cerevisiae)
1108	RZPDp202E095D	4.71	1.07	*WDR16*	WD repeat domain 16
1109	RZPDp202C118D	4.71	1.10	*FLJ20186*	Hypothetical protein FLJ20186
1110	IMAGp998D02437	4.71	1.14	*N/A*	Data not found
1111	IMAGp998C22385	4.71	1.13	*N/A*	Data not found
1112	IMAGp998F143027	4.71	1.21	*N/A*	Transcribed locus, moderately similar to XP_517599.2 bone morphogenetic protein receptor, type IB [Pan troglodytes]
1113	IMAGp998F151749	4.71	1.06	*USP39*	Ubiquitin specific peptidase 39
1114	IMAGp998E132010	4.71	1.10	*RASA2*	RAS p21 protein activator 2
1115	IMAGp998F101781	4.71	1.11	*XRCC6*	X-ray repair complementing defective repair in Chinese hamster cells 6 (Ku autoantigen, 70kDa)
1116	Ara-RCP1-2	4.71	1.53	*N/A*	Data not found
1117	IMAGp998B05373	4.71	1.11	*NRD1*	Nardilysin (N-arginine dibasic convertase)
1118	RZPDp202A074D	4.71	1.08	*KIAA1509*	KIAA1509
1119	IMAGp998I20639	4.71	1.11	*YPEL2*	Yippee-like 2 (Drosophila)
1120	IMAGp998H16151	4.71	1.18	*GRB10*	Growth factor receptor-bound protein 10
1121	IMAGp998K24349	4.71	1.27	*HLA-DRB1*	Major histocompatibility complex, class II, DR beta 1
1122	IMAGp998B04172	4.71	1.08	*KIAA1411*	KIAA1411
1123	IMAGp998H05270	4.71	1.07	*EYA2*	Eyes absent homolog 2 (Drosophila)
1124	IMAGp998I19178	4.71	1.17	*MS4A8B*	Membrane-spanning 4-domains, subfamily A, member 8B
1125	IMAGp998K07242	5.36	1.11	*IGFBP4*	Insulin-like growth factor binding protein 4
1126	IMAGp998L0873	5.36	1.15	*SKIV2L2*	Superkiller viralicidic activity 2-like 2 (S. cerevisiae)
1127	IMAGp998P02236	5.36	1.10	*IL6ST*	Interleukin 6 signal transducer (gp130, oncostatin M receptor)
1128	RZPDp201F0228D	5.36	1.10	*YARS*	Tyrosyl-tRNA synthetase
1129	IMAGp998I20590	5.36	1.14	*HIST1H3D*	Histone cluster 1, H3d
1130	RZPDp1096H0314D	5.36	1.05	*PPP1R8*	Protein phosphatase 1, regulatory (inhibitor) subunit 8
1131	IMAGp998F134494	5.36	1.07	*N/A*	Transcribed locus
1132	RZPDp201E0617D	5.36	1.10	*ZNF720*	Zinc finger protein 720
1133	IMAGp998I06597	5.36	1.20	*SELS*	Selenoprotein S
1134	IMAGp998D12140	5.36	1.22	*FGFR2*	Fibroblast growth factor receptor 2
1135	IMAGp998M015211	5.36	1.06	*LOC646951*	Similar to CG14142-PA
1136	RZPDp201B033D	5.36	1.12	*BNC2*	Basonuclin 2
1137	RZPDp202D101D	5.36	1.07	*SYMPK*	Symplekin
1138	IMAGp998D14240	5.36	1.09	*LRRC59*	Leucine rich repeat containing 59
1139	IMAGp998I20178	5.36	1.19	*SPP1*	Secreted phosphoprotein 1 (osteopontin, bone sialoprotein I, early T-lymphocyte activation 1)
1140	IMAGp998C03793	5.36	1.05	*MCF2L*	MCF.2 cell line derived transforming sequence-like
1141	IMAGp998C012580	5.36	1.07	*N/A*	Data not found
1142	IMAGp998D184645	0.00	0.66	*TMEM16G*	Transmembrane protein 16G
1143	IMAGp998P011858	0.17	0.81	*PLXNB1*	Plexin B1
1144	IMAGp998C18473	0.17	0.60	*REV3L*	REV3-like, catalytic subunit of DNA polymerase zeta (yeast)
1145	IMAGp998J185570	0.17	0.85	*XKR6*	XK, Kell blood group complex subunit-related family, member 6
1146	RZPDp201B0233D	0.56	0.85	*C10orf116*	Chromosome 10 open reading frame 116
1147	IMAGp998H171901	1.52	0.82	*C10orf116*	Growth arrest and DNA-damage-inducible, beta
1148	IMAGp998N141165	2.43	0.80	*HSF4*	Heat shock transcription factor 4
1149	IMAGp998L105077	2.43	0.82	*GGT3*	Gamma-glutamyltransferase 3
1150	IMAGp998G02615	2.82	0.79	*MYBPC1*	Myosin binding protein C, slow type
1151	RZPDp201A106D	3.78	0.69	*GGTL4*	Gamma-glutamyltransferase-like 4,Gamma-glutamyltransferase-like 4
1152	IMAGp998N01368	3.78	0.75	*SLC43A1*	Solute carrier family 43, member 1
1153	IMAGp998H17257	3.78	0.88	*N/A*	CDNA FLJ36638 fis, clone TRACH2018950
1154	IMAGp998E094498	3.78	0.89	*CCDC33*	Coiled-coil domain containing 33
1155	IMAGp998D13314	4.29	0.88	*SYNGR3*	Synaptogyrin 3
1156	IMAGp998B06415	4.29	0.89	*FAM122A*	Family with sequence similarity 122A
1157	IMAGp998E074019	4.29	0.88	*RP5-860F19.3*	KIAA1442 protein
1158	IMAGp998C131907	4.29	0.67	*TPM2*	Tropomyosin 2 (beta)
1159	IMAGp998J064155	4.29	0.83	*TPM1*	Tropomyosin 1 (alpha)
1160	IMAGp998G171945	4.29	0.90	*HKR1*	GLI-Kruppel family member HKR1
1161	IMAGp998O24111	4.29	0.87	*MICAL2*	Microtubule associated monoxygenase, calponin and LIM domain containing 2
1162	IMAGp998N01977	4.29	0.90	*KIF13A*	Kinesin family member 13A
1163	IMAGp998O061827	4.71	0.90	*DOCK1*	Dedicator of cytokinesis 1
1164	RZPDp201G075D	4.71	0.79	*P53AIP1*	P53-regulated apoptosis-inducing protein 1
1165	IMAGp998B15184	4.71	0.66	*DSC2*	Desmocollin 2
1166	IMAGp998D22127	4.71	0.90	*MGMT*	O-6-methylguanine-DNA methyltransferase
1167	IMAGp998E174411	4.71	0.92	*N/A*	CDNA clone IMAGE:4837645
1168	IMAGp998D241165	4.71	0.85	*ANKZF1*	Ankyrin repeat and zinc finger domain containing 1
1169	RZPDp202C081D	4.71	0.86	*FLJ30092*	AF-1 specific protein phosphatase
1170	IMAGp998F031851	4.71	0.76	*PARD3*	Par-3 partitioning defective 3 homolog (C. elegans)
1171	IMAGp998K22676	4.71	0.84	*PLEKHO1*	Pleckstrin homology domain containing, family O member 1
1172	IMAGp998J212983	4.71	0.59	*MSMB*	Microseminoprotein, beta-
1173	RZPDp1096C098D	4.71	0.89	*KIF1B*	Kinesin family member 1B
1174	IMAGp998E054894	4.71	0.85	*N/A*	CDNA FLJ43589 fis, clone SKNSH2010015
1175	IMAGp998A11421	4.71	0.78	*MT1H*	Metallothionein 1H
1176	IMAGp998E21691	4.71	0.90	*MGC45800*	Hypothetical protein LOC90768
1177	IMAGp998J195714	4.71	0.89	*N/A*	CDNA FLJ42545 fis, clone BRACE3004783
1178	IMAGp998M20388	4.71	0.75	*SLC22A3*	Solute carrier family 22 (extraneuronal monoamine transporter), member 3
1179	IMAGp998M242484	4.71	0.78	*N/A*	Transcribed locus
1180	IMAGp998O161161	4.71	0.84	*SERF1A*	Small EDRK-rich factor 1A (telomeric)
1181	IMAGp998C073966	4.71	0.92	*N/A*	Transcribed locus,Transcribed locus
1182	IMAGp998M131825	4.71	0.90	*JMJD4*	Jumonji domain containing 4
1183	IMAGp998F204494	4.71	0.90	*TRUB1*	TruB pseudouridine (psi) synthase homolog 1 (E. coli)
1184	IMAGp998M10728	4.71	0.87	*SERHL2*	Serine hydrolase-like 2
1185	IMAGp998N18174	4.71	0.92	*ELOVL2*	Transcribed locus,Elongation of very long chain fatty acids (FEN1/Elo2, SUR4/Elo3, yeast)-like 2
1186	RZPDp201F1017D	4.71	0.89	*FLCN*	Folliculin
1187	IMAGp998L04133	4.71	0.82	*DLC1*	Deleted in liver cancer 1
1188	IMAGp998D22407	4.71	0.77	*AZGP1*	Alpha-2-glycoprotein 1, zinc-binding
1189	IMAGp998E19235	4.71	0.91	*ITGB4BP*	Integrin beta 4 binding protein
1190	RZPDp201B043D	4.71	0.82	*LOC348262*	Hypothetical protein LOC348262
1191	IMAGp998G05276	4.71	0.86	*CLEC2B*	C-type lectin domain family 2, member B
1192	IMAGp998J125970	4.71	0.91	*PRSS23*	Protease, serine, 23,Transcribed locus
1193	IMAGp998G214170	4.71	0.93	*TMEM132D*	Transmembrane protein 132D
1194	IMAGp998G135933	4.71	0.79	*RALGPS2*	Ral GEF with PH domain and SH3 binding motif 2
1195	IMAGp998E171006	4.71	0.86	*PHF19*	PHD finger protein 19
