# Supplementary material for: Differential expression of apoptotic genes PDIA3 and MAP3K5 distinguishes between low- and high-risk prostate cancer
Source: Mol Cancer. 2009 Dec 27;8:130. doi: 10.1186/1476-4598-8-130 (PMC2807430; doi:10.1186/1476-4598-8-130)
Supplement: Additional file 2 — Differentially expressed genes between prostate normal and cancer tissue. A two class unpaired SAM test with 1000 permutations was performed to identify genes differentially regulated between normal and cancer tissue. The False Discovery Rate (FDR) was set below 5%. Genes were assigned using RZPD ID, gene symbol and gene name. [file 1476-4598-8-130-S2.PDF]

Additional File 2: Differentially expressed genes between prostate normal and cancer tissue

No.	RZPD ID	q-value(%)	Fold Change	Gene Symbol	Name
1	IMAGp998P17266	0.00	1.684	*REPS2*	Transcribed locus,RALBP1 associated Eps domain containing 2
2	IMAGp998D07222	0.00	1.563	*ATP6V1G1*	Transcribed locus,ATPase, H+ transporting, lysosomal 13kDa, V1 subunit G1
3	IMAGp998I23369	0.00	1.495	*TXN*	Thioredoxin
4	IMAGp998C21147	0.00	1.730	*C4orf18*	Chromosome 4 open reading frame 18
5	IMAGp998I01789	0.00	1.526	*C1orf85*	Chromosome 1 open reading frame 85
6	IMAGp998G06268	0.00	1.450	*GNL3*	Guanine nucleotide binding protein-like 3 (nucleolar)
7	IMAGp998I05236	0.00	1.620	*SEL1L*	Sel-1 suppressor of lin-12-like (C. elegans)
8	RZPDp202D029D	0.00	1.523	*RPS29*	Ribosomal protein S29
9	IMAGp998J12119	0.00	2.517	*ERG*	V-ets erythroblastosis virus E26 oncogene homolog (avian)
10	IMAGp998G09679	0.00	1.437	*RPL39*	Ribosomal protein L39,Transcribed locus, strongly similar to NP_037007.1 protein L39 [Rattus norvegicus]
11	IMAGp998M13642	0.00	1.500	*N/A*	MRNA; cDNA DKFZp547K189 (from clone DKFZp547K189)
12	IMAGp998H03792	0.00	1.454	*NPAL2*	NIPA-like domain containing 2
13	IMAGp998H09170	0.00	1.491	*CYCS*	Cytochrome c, somatic,Transcribed locus, weakly similar to XP_370188.1 protein MG06685.4 [Magnaporthe grisea 70-15]
14	IMAGp998M05615	0.00	1.318	*FAM84B*	Family with sequence similarity 84, member B
15	IMAGp998L23224	0.00	1.521	*SPG21*	Spastic paraplegia 21, maspardin (autosomal recessive, Mast syndrome)
16	IMAGp998C232012	0.00	1.635	*ALDH1A3*	Aldehyde dehydrogenase 1 family, member A3
17	IMAGp998G20275	0.00	1.305	*RNF24*	Ring finger protein 24,Full-length cDNA clone CS0DJ002YF02 of T cells (Jurkat cell line) Cot 10-normalized of Homo sapiens (human)
18	IMAGp998P24142	0.00	1.370	*TSPAN13*	Tetraspanin 13
19	IMAGp998H231115	0.00	1.318	*WT1*	Wilms tumor 1
20	IMAGp998F04157	0.00	1.376	*FGF13*	Fibroblast growth factor 13
21	IMAGp998D07314	0.00	1.327	*GIPC1,SNRPG*	GIPC PDZ domain containing family, member 1,Small nuclear ribonucleoprotein polypeptide G
22	IMAGp998A04469	0.00	1.226	*MEMO1*	Chromosome 2 open reading frame 4
23	IMAGp998E06781	0.00	1.498	*MBOAT2*	Membrane bound O-acyltransferase domain containing 2
24	IMAGp998E011937	0.00	1.256	*TMTC4*	Transmembrane and tetratricopeptide repeat containing 4,Transcribed locus
25	IMAGp998L03828	0.00	1.701	*TMEM178*	Transmembrane protein 178
26	IMAGp998M22780	0.00	1.539	*N/A*	Transcribed locus, strongly similar to XP_001172939.1 hypothetical protein [Pan troglodytes]
27	IMAGp998M23234	0.14	1.356	*EDEM3*	ER degradation enhancer, mannosidase alpha-like 3
28	IMAGp998J01615	0.14	1.185	*TRIM33*	Tripartite motif-containing 33
29	IMAGp998N061965	0.14	1.496	*TBL1XR1*	Transducin (beta)-like 1X-linked receptor 1
30	IMAGp998D04631	0.14	1.511	*ATP11A*	ATPase, Class VI, type 11A
31	RZPDp202D0210D	0.14	1.198	*AK3*	Adenylate kinase 3
32	RZPDp1096G095D	0.14	1.497	*SFTPA2*	Surfactant, pulmonary-associated protein A2,Surfactant, pulmonary-associated protein A2
33	IMAGp998O16872	0.14	1.295	*DCUN1D5*	Transcribed locus,DCN1, defective in cullin neddylation 1, domain containing 5 (S. cerevisiae)
34	IMAGp998M03537	0.14	1.421	*APLP2*	Amyloid beta (A4) precursor-like protein 2
35	IMAGp998H01660	0.14	1.271	*MAT1A*	Methionine adenosyltransferase I, alpha
36	IMAGp998C10644	0.14	1.530	*ABCC4*	ATP-binding cassette, sub-family C (CFTR/MRP), member 4
37	IMAGp998P17726	0.14	1.306	*CBX3*	Chromobox homolog 3 (HP1 gamma homolog, Drosophila)
38	IMAGp998G07729	0.14	1.214	*SNX8*	Sorting nexin 8
39	IMAGp998H05155	0.14	1.202	*MRPS35*	Mitochondrial ribosomal protein S35
40	IMAGp998L061167	0.14	1.361	*SNX4*	Sorting nexin 4
41	IMAGp998C09679	0.14	1.262	*hCG_1815491*	HCG1815491
42	IMAGp998F10112	0.14	1.585	*PLA1A*	Phospholipase A1 member A
43	IMAGp998J14179	0.14	1.213	*RAB11A*	RAB11A, member RAS oncogene family
44	IMAGp998P04272	0.14	1.222	*COX7A2L*	Cytochrome c oxidase subunit VIIa polypeptide 2 like
45	IMAGp998O15696	0.14	1.174	*CAPZA1*	Capping protein (actin filament) muscle Z-line, alpha 1
46	IMAGp998H19228	0.14	1.274	*SMAD5*	SMAD family member 5
47	RZPDp201G0228D	0.14	1.256	*PPP1CB*	Protein phosphatase 1, catalytic subunit, beta isoform
48	IMAGp998G24794	0.14	1.321	*N/A*	CDNA FLJ38388 fis, clone FEBRA2004485
49	RZPDp201E064D	0.14	1.250	*CD109*	CD109 molecule
50	IMAGp998O2092	0.14	1.320	*SAE2*	SUMO1 activating enzyme subunit 2
51	IMAGp998E03727	0.14	1.153	*IQGAP2*	IQ motif containing GTPase activating protein 2
52	IMAGp998B11437	0.24	1.193	*THOC2*	THO complex 2
53	IMAGp998C11339	0.24	1.232	*PCF11*	PCF11, cleavage and polyadenylation factor subunit, homolog (S. cerevisiae)
54	IMAGp998H22631	0.24	1.206	*GARNL1*	GTPase activating Rap/RanGAP domain-like 1
55	IMAGp998F11377	0.24	1.354	*SNORA24*	Small nucleolar RNA, H/ACA box 24
56	IMAGp998P05178	0.24	1.230	*SATB2,NACA*	SATB family member 2,Nascent-polypeptide-associated complex alpha polypeptide
57	IMAGp998L162576	0.24	1.226	*PRKAA1*	Protein kinase, AMP-activated, alpha 1 catalytic subunit
58	IMAGp998B121117	0.24	1.205	*SOX4*	SRY (sex determining region Y)-box 4
59	IMAGp998M171825	0.24	1.138	*LOC643836*	Similar to Zinc finger protein 62 homolog (Zfp-62) (ZT3)
60	IMAGp998F172577	0.32	1.319	*N/A*	Transcribed locus
61	IMAGp998A132000	0.32	1.285	*STX5*	Syntaxin 5
62	RZPDp202C098D	0.32	1.155	*PYCR1*	Pyrroline-5-carboxylate reductase 1
63	IMAGp998K074642	0.32	1.612	*NPY*	Neuropeptide Y
64	IMAGp998H12204	0.32	1.559	*UGDH*	UDP-glucose dehydrogenase
65	IMAGp998K15639	0.32	1.766	*CACNA1D*	Transcribed locus, strongly similar to XP_530610.1
66	IMAGp998L2374	0.32	1.172	*PABPC4*	Poly(A) binding protein, cytoplasmic 4 (inducible form)
67	IMAGp998M16210	0.32	1.263	*MATR3*	Matrin 3
68	IMAGp998G101114	0.32	1.176	*MAN2A1*	Mannosidase, alpha, class 2A, member 1
69	IMAGp998A0372	0.32	1.240	*HNRPU*	Heterogeneous nuclear ribonucleoprotein U (scaffold attachment factor A)
70	IMAGp998P192977	0.32	1.174	*TLR10*	Toll-like receptor 10
71	IMAGp998I01473	0.32	1.251	*GOLGA4*	Golgi autoantigen, golgin subfamily a, 4
72	IMAGp998O0186	0.32	1.496	*COX6C*	Cytochrome c oxidase subunit VIc
73	IMAGp998C20532	0.49	1.244	*OGT*	O-linked N-acetylglucosamine (GlcNAc) transferase (UDP-N-acetylglucosamine:polypeptide-N-acetylglucosaminyl transferase)
74	IMAGp998A05197	0.49	1.139	*PSCD3*	Pleckstrin homology, Sec7 and coiled-coil domains 3
75	IMAGp998H04194	0.49	1.224	*HIST1H2BK*	Histone cluster 1, H2bk
76	IMAGp998K18671	0.49	1.267	*NME1*	Non-metastatic cells 1, protein (NM23A) expressed in
77	IMAGp998L06170	0.49	1.206	*ATP5L*	ATP synthase, H+ transporting, mitochondrial F0 complex, subunit G
78	IMAGp998C14789	0.49	1.217	*COX7C*	Cytochrome c oxidase subunit VIIc
79	RZPDp1096D017D	0.49	1.588	*PCDHAC2*	Protocadherin alpha subfamily C, 2
80	RZPDp202B1110D	0.49	1.153	*UCRC*	Ubiquinol-cytochrome c reductase complex (7.2 kD)
81	IMAGp998C224304	0.49	1.127	*MBNL2*	Muscleblind-like 2 (Drosophila)
82	IMAGp998A08156	0.49	1.153	*RWDD1*	RWD domain containing 1
83	IMAGp998A22642	0.49	1.173	*N/A*	Transcribed locus,Clone CDABP0105 mRNA sequence,Transcribed locus
84	IMAGp998H06241	0.49	1.207	*SRM*	Spermidine synthase
85	IMAGp998H22222	0.49	1.129	*CD2AP*	CD2-associated protein
86	IMAGp998B02536	0.49	1.143	*MRPL22*	Mitochondrial ribosomal protein L22
87	IMAGp998L10144	0.49	1.274	*MAP3K7IP2*	Mitogen-activated protein kinase kinase kinase 7 interacting protein 2
88	IMAGp998H194500	0.49	1.144	*N/A*	CDNA FLJ35874 fis, clone TESTI2008331
89	IMAGp998N211942	0.49	1.570	*AMACR*	Alpha-methylacyl-CoA racemase
90	IMAGp998I21692	0.49	1.190	*VBP1*	Von Hippel-Lindau binding protein 1
91	IMAGp998G01193	0.49	1.508	*RPLP1*	Ribosomal protein, large, P1
92	RZPDp1096G1215D	0.66	1.250	*RBBP4*	Retinoblastoma binding protein 4
93	RZPDp201H0225D	0.66	1.231	*PRSS1*	TCR beta chain Vbeta13S1/S3-SLQGVNSPLHF-Jbeta1.6,Protease, serine, 1 (trypsin 1)
94	IMAGp998L11214	0.66	1.255	*PLEKHA5*	Pleckstrin homology domain containing, family A member 5
95	IMAGp998I20639	0.66	1.198	*YPEL2*	Yippee-like 2 (Drosophila)
96	RZPDp201H022D	0.66	1.300	*SEC61G*	Sec61 gamma subunit
97	IMAGp998O011197	0.66	1.139	*DKC1*	Dyskeratosis congenita 1, dyskerin
98	IMAGp998B12621	0.66	1.357	*LBR*	Lamin B receptor
99	RZPDp201C0535D	0.66	1.282	*DC2*	DC2 protein
100	IMAGp998F02226	0.66	1.295	*GALNT3*	UDP-N-acetyl-alpha-D-galactosamine:polypeptide N-acetylgalactosaminyltransferase 3 (GalNAc-T3)
101	RZPDp202A038D	0.66	1.293	*FH*	Fumarate hydratase
102	IMAGp998B064272	0.66	1.141	*TMEM81*	Transmembrane protein 81
103	IMAGp998P07649	0.66	1.421	*HNRPH1*	Heterogeneous nuclear ribonucleoprotein H1 (H)
104	IMAGp998F03979	0.66	1.261	*SRBD1*	S1 RNA binding domain 1
105	IMAGp998N021903	0.66	1.196	*RHOF*	Ras homolog gene family, member F (in filopodia)
106	IMAGp998D14240	0.66	1.214	*LRRC59,EME1*	Leucine rich repeat containing 59,Essential meiotic endonuclease 1 homolog 1 (S. pombe)
107	IMAGp998N07174	0.66	1.248	*MORF4L2*	Mortality factor 4 like 2
108	IMAGp998P081159	0.66	1.213	*HK2*	Hexokinase 2
109	RZPDp202F049D	0.66	1.201	*NEB*	Nebulin
110	IMAGp998P11319	0.84	1.305	*GPR162*	G protein-coupled receptor 162
111	IMAGp998F10422	0.84	1.225	*MRPL3*	Mitochondrial ribosomal protein L3
112	IMAGp998H221936	0.84	1.265	*CCDC14*	Coiled-coil domain containing 14,Transcribed locus
113	RZPDp202A033D	0.84	1.257	*AIFM2*	Apoptosis-inducing factor, mitochondrion-associated, 2
114	IMAGp998N05829	0.84	1.176	*PSMD11*	Proteasome (prosome, macropain) 26S subunit, non-ATPase, 11
115	IMAGp998N041725	0.84	1.331	*IKZF5,YWHAQ*	IKAROS family zinc finger 5 (Pegasus),Tyrosine 3-monooxygenase/tryptophan 5-monooxygenase activation protein, theta polypeptide
116	IMAGp998O0669	0.84	1.542	*SPOCK1*	Sparc/osteonectin, cwcv and kazal-like domains proteoglycan (testican) 1
117	IMAGp998J01976	0.84	1.135	*PPM1H*	Protein phosphatase 1H (PP2C domain containing)
118	IMAGp998H23880	0.84	1.233	*TAF9*	TAF9 RNA polymerase II, TATA box binding protein (TBP)-associated factor, 32kDa
119	IMAGp998D05738	0.84	1.130	*RCN2*	Reticulocalbin 2, EF-hand calcium binding domain
120	IMAGp998J2273	0.84	1.163	*ARL6IP1*	ADP-ribosylation factor-like 6 interacting protein 1
121	IMAGp998G05239	0.84	1.235	*RPL38,TEK*	Ribosomal protein L38,TEK tyrosine kinase, endothelial (venous malformations, multiple cutaneous and mucosal)
122	IMAGp998P03693	0.84	1.135	*OXR1*	Oxidation resistance 1
123	IMAGp998H02386	0.84	1.530	*KIAA1450*	KIAA1450 protein
124	IMAGp998K04160	0.84	1.125	*ANXA7*	Annexin A7
125	IMAGp998F06177	0.84	1.179	*YWHAE,LOC649395*	Tyrosine 3-monooxygenase/tryptophan 5-monooxygenase activation protein, epsilon polypeptide
126	IMAGp998C01311	0.84	1.352	*CCNG2*	Cyclin G2
127	RZPDp201F0426D	0.84	1.176	*TMCC2*	Transmembrane and coiled-coil domain family 2
128	RZPDp201D0418D	0.84	1.167	*ATP5G1*	ATP synthase, H+ transporting, mitochondrial F0 complex, subunit C1 (subunit 9)
129	IMAGp998C18473	0.84	1.567	*REV3L*	REV3-like, catalytic subunit of DNA polymerase zeta (yeast)
130	IMAGp998M03235	0.84	1.221	*SGPP1*	Sphingosine-1-phosphate phosphatase 1
131	IMAGp998H16151	0.84	1.344	*GRB10*	Growth factor receptor-bound protein 10
132	IMAGp998B171010	0.84	1.186	*ODF2*	Outer dense fiber of sperm tails 2
133	RZPDp202B129D	1.20	1.236	*NPM1*	Nucleophosmin (nucleolar phosphoprotein B23, numatrin)
134	IMAGp998G032039	1.20	1.125	*ZNF785*	Zinc finger protein 785
135	RZPDp202A074D	1.20	1.165	*KIAA1509,CFL1*	KIAA1509,Cofilin 1 (non-muscle)

136	IMAGp998H07686	1.20	1.116	*ACBD3*	Acyl-Coenzyme A binding domain containing 3
137	IMAGp998I03524	1.20	1.177	*UQCRB,LOC727947*	Ubiquinol-cytochrome c reductase binding protein,Similar to Ubiquinol-cytochrome c reductase complex 14 kDa protein (Complex III subunit VI) (QP-C)
138	IMAGp998C21313	1.20	1.275	*COX6B1*	Cytochrome c oxidase subunit Vib polypeptide 1 (ubiquitous)
139	IMAGp998B23461	1.20	1.228	*ATP5E*	ATP synthase, H+ transporting, mitochondrial F1 complex, epsilon subunit
140	IMAGp998H09697	1.20	1.177	*ITGB1,EMR3*	Integrin, beta 1 (fibronectin receptor, beta polypeptide, antigen CD29 includes MDF2, MSK12)
141	IMAGp998K17340	1.20	1.351	*COPG*	Transcribed locus,Coatomer protein complex, subunit gamma
142	IMAGp998J092038	1.20	1.128	*C12orf29*	Chromosome 12 open reading frame 29
143	IMAGp998O112010	1.20	1.399	*TPD52*	Tumor protein D52
144	IMAGp998B01522	1.20	1.257	*RPL31*	Ribosomal protein L31
145	IMAGp998I0897	1.20	1.208	*COX17*	COX17 cytochrome c oxidase assembly homolog (S. cerevisiae)
146	IMAGp998H12275	1.89	1.162	*UBE2N*	Ubiquitin-conjugating enzyme E2N (UBC13 homolog, yeast)
147	IMAGp998G17783	1.89	1.141	*SERF1A*	Small EDRK-rich factor 1A (telomeric)
148	IMAGp998P08140	1.89	1.162	*RABGGTB*	Rab geranylgeranyltransferase, beta subunit
149	IMAGp998M13132	1.89	1.369	*YWHAZ*	Tyrosine 3-monooxygenase/tryptophan 5-monooxygenase activation protein, zeta polypeptide
150	IMAGp998O06698	1.89	1.218	*ACSL3*	Acyl-CoA synthetase long-chain family member 3
151	IMAGp998M011199	1.89	1.301	*RPL27*	Ribosomal protein L27
152	IMAGp998O054432	1.89	1.271	*SFTPA2*	Surfactant, pulmonary-associated protein A2,Surfactant, pulmonary-associated protein A2
153	IMAGp998L225406	1.89	1.364	*GALNT7*	UDP-N-acetyl-alpha-D-galactosamine:polypeptide N-acetylgalactosaminyltransferase 7 (GalNAc-T7)
154	RZPDp1096D0217D	1.89	1.136	*SLC38A2*	Solute carrier family 38, member 2
155	IMAGp998H171785	1.89	1.185	*PRKAA1*	Protein kinase, AMP-activated, alpha 1 catalytic subunit
156	IMAGp998A061009	1.89	1.124	*FXR1*	Fragile X mental retardation, autosomal homolog 1
157	RZPDp202B116D	1.89	1.171	*RZPDp202B116D*	RZPDp202B116D
158	IMAGp998B05191	1.89	1.132	*SLC25A32*	Solute carrier family 25, member 32
159	IMAGp998N09473	1.89	1.137	*CGA*	Glycoprotein hormones, alpha polypeptide
160	RZPDp201B0220D	1.89	1.148	*NKRF*	NF-kappaB repressing factor
161	RZPDp201A0820D	1.89	1.293	*TM9SF2*	Transmembrane 9 superfamily member 2
162	IMAGp998L23644	1.89	1.123	*GALNT1*	UDP-N-acetyl-alpha-D-galactosamine:polypeptide N-acetylgalactosaminyltransferase 1 (GalNAc-T1)
163	IMAGp998E18284	1.89	1.191	*HYOU1*	Hypoxia up-regulated 1
164	IMAGp998O091157	1.89	1.220	*PDLIM2*	PDZ and LIM domain 2 (mystique)
165	IMAGp998O03131	1.89	1.142	*MKI67IP*	MKI67 (FHA domain) interacting nucleolar phosphoprotein
166	IMAGp998F03380	1.89	1.278	*COX7A2*	Cytochrome c oxidase subunit VIIa polypeptide 2 (liver)
167	IMAGp998P10625	1.89	1.143	*C6orf65*	Chromosome 6 open reading frame 65
168	IMAGp998C102577	1.89	1.110	*AQP7P1*	Aquaporin 7 pseudogene 1
169	IMAGp998I05460	2.75	1.141	*EIF2S1*	Eukaryotic translation initiation factor 2, subunit 1 alpha, 35kDa
170	IMAGp998A12536	2.75	1.314	*KIAA0143*	KIAA0143 protein
171	IMAGp998J231864	2.75	1.188	*NDUFA4*	NADH dehydrogenase (ubiquinone) 1 alpha subcomplex, 4, 9kDa
172	IMAGp998A011726	2.75	1.135	*STIP1*	Stress-induced-phosphoprotein 1 (Hsp70/Hsp90-organizing protein)
173	IMAGp998J08179	2.75	1.622	*APOD*	Apolipoprotein D
174	IMAGp998J151864	2.75	1.116	*ZNF717*	Zinc finger protein 717
175	IMAGp998B16580	2.75	1.173	*FAM96A*	Family with sequence similarity 96, member A
176	IMAGp998O131204	2.75	1.103	*USP33*	Ubiquitin specific peptidase 33
177	RZPDp1096D0315D	2.75	1.144	*GLT25D1*	Glycosyltransferase 25 domain containing 1
178	IMAGp998L162227	2.75	1.245	*LDHA*	Lactate dehydrogenase A
179	RZPDp1096A111D	2.75	1.201	*SHOC2*	Soc-2 suppressor of clear homolog (C. elegans)
180	IMAGp998E09327	2.75	1.250	*RPL7*	Ribosomal protein L7
181	RZPDp1096C0218D	2.75	1.116	*ZNF614*	Zinc finger protein 614
182	IMAGp998J2389	2.75	1.182	*LSM3*	LSM3 homolog, U6 small nuclear RNA associated (S. cerevisiae)
183	IMAGp998J08128	2.75	1.162	*CCDC72*	PNAS-103 mRNA, partial sequence,Coiled-coil domain containing 72
184	RZPDp201H0327D	2.75	1.465	*HSD17B4*	Hydroxysteroid (17-beta) dehydrogenase 4
185	RZPDp1096C033D	2.75	1.184	*GARNL1*	GTPase activating Rap/RanGAP domain-like 1
186	IMAGp998I195918	2.75	1.110	*TP53AP1*	TP53 activated protein 1,Transcribed locus
187	IMAGp998D03170	2.75	1.414	*TMEM123*	Transmembrane protein 123
188	RZPDp201D0917D	3.76	1.165	*EIF2S2*	Eukaryotic translation initiation factor 2, subunit 2 beta, 38kDa
189	IMAGp998O12206	3.76	1.337	*ZFX*	Zinc finger protein, X-linked
190	RZPDp201E0419D	3.76	1.138	*ACLY*	ATP citrate lyase
191	IMAGp998D13403	3.76	1.278	*C2orf25*	Chromosome 2 open reading frame 25
192	IMAGp998L02206	3.76	1.092	*GGCX*	Gamma-glutamyl carboxylase
193	IMAGp998J23159	3.76	1.175	*SKP1A*	S-phase kinase-associated protein 1A (p19A)
194	RZPDp202D085D	3.76	1.244	*STK11*	Serine/threonine kinase 11
195	IMAGp998G1518	3.76	1.364	*ANXA5*	Annexin A5
196	IMAGp998F181782	3.76	1.135	*SEH1L*	SEH1-like (S. cerevisiae)
197	IMAGp998L03142	3.76	1.149	*ADSL*	Adenylosuccinate lyase
198	IMAGp998N071789	3.76	1.423	*SH3PXD2A*	SH3 and PX domains 2A
199	RZPDp201D0326D	3.76	1.152	*RANBP1*	RAN binding protein 1
200	IMAGp998M104303	3.76	1.125	*N/A*	Transcribed locus
201	RZPDp202H025D	3.76	1.122	*AURKA*	Aurora kinase A
202	RZPDp1096H114D	3.76	1.118	*CCNU*	Uracil-DNA glycosylase 2
203	IMAGp998M15411	3.76	1.321	*RPL30*	Ribosomal protein L30
204	IMAGp998K06226	3.76	1.115	*FNDC3A*	Fibronectin type III domain containing 3A
205	IMAGp998E081961	3.76	1.283	*RPL24*	Transcribed locus, weakly similar to XP_366635.1 protein MG02711.4 [Magnaporthe grisea 70-15],Ribosomal protein L24
206	IMAGp998N14139	3.76	1.208	*N/A*	CDNA clone IMAGE:5262496
207	IMAGp998K18127	3.76	1.170	*N/A*	Transcribed locus
208	IMAGp998E055449	3.76	1.145	*ITGB7*	Integrin, beta 7
209	IMAGp998M1770	3.76	1.172	*SFPQ*	Splicing factor proline/glutamine-rich (polypyrimidine tract binding protein associated)
210	IMAGp998G15694	3.76	1.217	*SOD1*	Superoxide dismutase 1, soluble (amyotrophic lateral sclerosis 1 (adult))
211	IMAGp998C11367	3.76	1.247	*RPL23A,hCG_16001*	Ribosomal protein L23a,HCG16001
212	IMAGp998F23436	3.76	1.132	*HIPK1*	Homeodomain interacting protein kinase 1
213	IMAGp998I1579	3.76	1.260	*THSD1P*	Thrombospondin, type I, domain containing 1 pseudogene
214	IMAGp998L24621	3.76	1.129	*ZDHHC9*	Zinc finger, DHHC-type containing 9
215	IMAGp998N07530	3.76	1.115	*AMMECR1*	Alport syndrome, mental retardation, midface hypoplasia and elliptocytosis chromosomal region, gene 1
216	IMAGp998L06173	3.76	1.210	*TCBA1*	T-cell lymphoma breakpoint associated target 1
217	RZPDp202B064D	3.76	1.153	*NCR3*	Natural cytotoxicity triggering receptor 3
218	RZPDp1096E014D	3.76	1.104	*KCNS3*	Potassium voltage-gated channel, delayed-rectifier, subfamily S, member 3
219	IMAGp998N16280	3.76	1.183	*SPIN1*	Spindlin 1
220	IMAGp998L073856	4.84	1.115	*ATF2*	Activating transcription factor 2
221	IMAGp998E164175	4.84	1.145	*RPL10L*	Ribosomal protein L10-like
222	IMAGp998J241199	4.84	1.116	*YWHAG*	Tyrosine 3-monooxygenase/tryptophan 5-monooxygenase activation protein, gamma polypeptide
223	RZPDp1096C0420D	4.84	1.112	*RAD21*	RAD21 homolog (S. pombe)
224	IMAGp998B12612	4.84	1.096	*MRPL42*	Mitochondrial ribosomal protein L42
225	IMAGp998L14194	4.84	1.116	*TXNDC1*	Thioredoxin domain containing 1
226	IMAGp998P183582	4.84	1.259	*HS3ST4*	Heparan sulfate (glucosamine) 3-O-sulfotransferase 4
227	IMAGp998D01688	4.84	1.108	*LOC285419*	Hypothetical protein LOC285419
228	RZPDp1096D0915D	4.84	1.149	*LYSMD4*	LysM, putative peptidoglycan-binding, domain containing 4
229	IMAGp998H16437	4.84	1.121	*USMG5*	Upregulated during skeletal muscle growth 5 homolog (mouse)
230	IMAGp998E201824	4.84	1.172	*NDUFS5*	NADH dehydrogenase (ubiquinone) Fe-S protein 5, 15kDa (NADH-coenzyme Q reductase)
231	RZPDp201E0532D	4.84	1.118	*GDI2*	GDP dissociation inhibitor 2
232	IMAGp998F24469	4.84	1.119	*C11orf10*	Chromosome 11 open reading frame 10
233	IMAGp998C15132	4.84	1.563	*CAMKK2,CRYL1*	Calcium/calmodulin-dependent protein kinase kinase 2, beta,Crystallin, lambda 1
234	IMAGp998C041785	4.84	1.090	*N/A*	Transcribed locus
235	IMAGp998F081722	4.84	1.220	*KIAA1344*	KIAA1344
236	IMAGp998G23667	4.84	1.226	*C6orf108*	Chromosome 6 open reading frame 108
237	RZPDp1096A0717D	4.84	1.095	*MARCH6*	Membrane-associated ring finger (C3HC4) 6
238	IMAGp998N09625	4.84	1.261	*ARF4*	ADP-ribosylation factor 4
239	IMAGp998D17244	4.84	1.125	*NUP107*	Nucleoporin 107kDa
240	IMAGp998D041904	4.84	1.111	*BXDC1*	Brix domain containing 1
241	RZPDp202B097D	4.84	1.218	*TTLL12*	Tubulin tyrosine ligase-like family, member 12
242	RZPDp201D1026D	4.84	1.180	*ARF5*	ADP-ribosylation factor 5
243	IMAGp998N141199	4.84	1.240	*IFI30*	Interferon, gamma-inducible protein 30
244	IMAGp998B096097	0.00	0.480	*LAMB3*	Laminin, beta 3
245	IMAGp998B225484	0.00	0.380	*COL9A1*	Collagen, type IX, alpha 1
246	IMAGp998F18342	0.00	0.279	*NEFH*	Neurofilament, heavy polypeptide 200kDa
247	IMAGp998P07172	0.00	0.590	*GPC1*	Glypican 1
248	RZPDp201H0819D	0.00	0.526	*NDRG2*	NDRG family member 2
249	IMAGp998H13246	0.00	0.650	*KRT6A*	Keratin 6A
250	IMAGp998B221777	0.00	0.709	*WFDC2*	WAP four-disulfide core domain 2
251	IMAGp998P09780	0.00	0.620	*KRT13*	Keratin 13
252	IMAGp998L02239	0.00	0.366	*FCGBP*	Fc fragment of IgG binding protein
253	IMAGp998C12230	0.00	0.627	*C21orf63*	Chromosome 21 open reading frame 63
254	IMAGp998A132675	0.00	0.513	*LOC152485*	Hypothetical protein LOC152485
255	IMAGp998D031999	0.00	0.610	*TRIM29*	Tripartite motif-containing 29
256	IMAGp998K151776	0.00	0.469	*KRT19*	Keratin 19
257	IMAGp998G08626	0.00	0.567	*N/A*	Transcribed locus
258	IMAGp998J02547	0.00	0.598	*SYT7*	Synaptotagmin VII
259	IMAGp998L074548	0.00	0.479	*N/A*	Data not found
260	IMAGp998A065260	0.00	0.711	*N/A*	Transcribed locus
261	IMAGp998J02348	0.00	0.759	*THSD4*	Thrombospondin, type I, domain containing 4
262	IMAGp998E074019	0.00	0.723	*RP5-860F19.3*	KIAA1442 protein
263	IMAGp998G155575	0.00	0.694	*N/A*	CDNA FLJ25345 fis, clone TST01118
264	IMAGp998M15110	0.00	0.629	*GPD1L*	Glycerol-3-phosphate dehydrogenase 1-like
265	IMAGp998G185401	0.00	0.473	*VSIG2*	V-set and immunoglobulin domain containing 2
266	IMAGp998L22373	0.00	0.560	*AQP3*	Aquaporin 3 (Gill blood group)
267	IMAGp998H06192	0.00	0.634	*S100P*	S100 calcium binding protein P
268	RZPDp202D017D	0.00	0.666	*GPD1L*	Glycerol-3-phosphate dehydrogenase 1-like
269	RZPDp1096B124D	0.00	0.710	*LMNA*	Lamin A/C
270	IMAGp998B07284	0.00	0.611	*FADS2*	Fatty acid desaturase 2

271	IMAGp998J17789	0.00	0.661	ZDHHC14	Zinc finger, DHHC-type containing 14
272	IMAGp998K04133	0.00	0.432	ZDHHC3	Zinc finger, DHHC-type containing 3
273	RZPDp201C036D	0.00	0.616	ATP2C2	ATPase, Ca++ transporting, type 2C, member 2
274	IMAGp998J125767	0.00	0.607	THSD4	Thrombospondin, type I, domain containing 4
275	IMAGp998G19782	0.00	0.752	COL7A1	Collagen, type VII, alpha 1 (epidermolysis bullosa, dystrophic, dominant and recessive)
276	RZPDp201B0233D	0.00	0.554	C10orf116	Chromosome 10 open reading frame 116
277	IMAGp998P2389	0.00	0.758	C21orf25	Chromosome 21 open reading frame 25
278	IMAGp998H05270	0.00	0.784	EYA2	Eyes absent homolog 2 (Drosophila)
279	IMAGp998C19245	0.00	0.713	NR1H2	Nuclear receptor subfamily 1, group H, member 2
280	IMAGp998P19276	0.00	0.661	N/A	Transcribed locus
281	IMAGp998F09155	0.00	0.740	CTSH	Cathepsin H
282	RZPDp201B0235D	0.00	0.626	SERPINB8	Serpin peptidase inhibitor, clade B (ovalbumin), member 8
283	RZPDp1096C0517D	0.00	0.624	FADS2	Fatty acid desaturase 2
284	IMAGp998C21165	0.00	0.767	PER1	Period homolog 1 (Drosophila)
285	IMAGp998M04380	0.00	0.756	CXCL5	Chemokine (C-X-C motif) ligand 5
286	IMAGp998F181775	0.00	0.766	KRT16	Keratin 16 (focal non-epidermolytic palmoplantar keratoderma)
287	IMAGp998F10191	0.00	0.635	PERP	PERP, TP53 apoptosis effector
288	IMAGp998E20251	0.00	0.760	LAMB2	Laminin, beta 2 (laminin S)
289	IMAGp998A19110	0.00	0.717	DHX38	DEAH (Asp-Glu-Ala-His) box polypeptide 38
290	IMAGp998N102000	0.00	0.730	FER1L3	Fer-1-like 3, myoferlin (C. elegans)
291	IMAGp998L18113	0.00	0.826	C9orf3	Chromosome 9 open reading frame 3
292	IMAGp998E13793	0.00	0.615	NFKBIA	Nuclear factor of kappa light polypeptide gene enhancer in B-cells inhibitor, alpha
293	IMAGp998O171776	0.00	0.664	LGALS3BP	Lectin, galactoside-binding, soluble, 3 binding protein
294	RZPDp202A078D	0.00	0.737	MYH14	Myosin, heavy chain 14
295	IMAGp998J152985	0.00	0.799	ZNF395	Zinc finger protein 395
296	IMAGp998C12116	0.00	0.718	hCG_2003663	HCG2003663
297	IMAGp998D10131	0.00	0.766	STX4	Syntaxin 4
298	IMAGp998L1678	0.00	0.671	DGCR8	DiGeorge syndrome critical region gene 8
299	IMAGp998P17239	0.00	0.667	ZNFX1	Zinc finger, NFX1-type containing 1
300	IMAGp998N09165	0.00	0.802	RUTBC3	RUN and TBC1 domain containing 3
301	RZPDp201F054D	0.00	0.693	GNMT	Glycine N-methyltransferase
302	IMAGp998J2392	0.00	0.584	ANPEP	Alanyl (membrane) aminopeptidase (aminopeptidase N, aminopeptidase M, microsomal aminopeptidase, CD13, p150)
303	IMAGp998N111004	0.00	0.759	N/A	Transcribed locus
304	IMAGp998H18566	0.00	0.614	IER3	Immediate early response 3
305	IMAGp998C08442	0.00	0.702	N/A	Data not found
306	IMAGp998J12380	0.00	0.781	MRPS18A	Mitochondrial ribosomal protein S18A
307	IMAGp998G05288	0.00	0.693	GABARAPL1	GABA(A) receptor-associated protein like 1
308	IMAGp998N09781	0.00	0.689	AUTS2	Autism susceptibility candidate 2
309	IMAGp998K12697	0.00	0.545	MYLK	Myosin, light chain kinase
310	IMAGp998B075198	0.00	0.656	SERPINB11	Serpin peptidase inhibitor, clade B (ovalbumin), member 11
311	IMAGp998F02140	0.00	0.788	ITPKC	Inositol 1,4,5-trisphosphate 3-kinase C
312	IMAGp998A02189	0.00	0.794	N/A	Transcribed locus
313	IMAGp998P111206	0.00	0.733	METTL7A	Methyltransferase like 7A
314	RZPDp1096C0615D	0.00	0.814	NXF1	Nuclear RNA export factor 1
315	IMAGp998M03113	0.00	0.743	ORMDL3	ORM1-like 3 (S. cerevisiae)
316	RZPDp202G064D	0.00	0.745	TCEAL3	Transcription elongation factor A (SII)-like 3
317	RZPDp202H036D	0.00	0.798	DHRS4	Dehydrogenase/reductase (SDR family) member 4
318	IMAGp998P09274	0.00	0.765	PMVK	Phosphomevalonate kinase
319	IMAGp998I18204	0.00	0.830	RBM10	RNA binding motif protein 10
320	IMAGp998P16781	0.00	0.817	EMILIN3	Elastin microfibril interfacer 3
321	IMAGp998P013196	0.00	0.825	N/A	Transcribed locus
322	IMAGp998M172272	0.00	0.582	ZBTB20	Zinc finger and BTB domain containing 20
323	IMAGp998P11266	0.00	0.743	ACAA1	Acetyl-Coenzyme A acyltransferase 1 (peroxisomal 3-oxoacyl-Coenzyme A thiolase)
324	IMAGp998I115626	0.00	0.542	N/A	Transcribed locus
325	IMAGp998F11159	0.00	0.807	CHD4	Chromodomain helicase DNA binding protein 4
326	IMAGp998H09672	0.00	0.785	PGM5	Phosphoglucomutase 5
327	RZPDp201G1129D	0.00	0.688	FLNB	Filamin B, beta (actin binding protein 278)
328	IMAGp998O115997	0.00	0.712	LOC152485	Hypothetical protein LOC152485
329	IMAGp998G22149	0.00	0.773	EXOC3	Exocyst complex component 3
330	IMAGp998A05373	0.00	0.809	CTDP1	CTD (carboxy-terminal domain, RNA polymerase II, polypeptide A) phosphatase, subunit 1
331	IMAGp998H244408	0.00	0.829	RNF212	Ring finger protein 212
332	IMAGp998J092001	0.00	0.842	DMKN	Dermokine
333	IMAGp998P09159	0.00	0.679	PDE9A	Phosphodiesterase 9A
334	IMAGp998F13667	0.00	0.770	N/A	Transcribed locus
335	IMAGp998N06617	0.00	0.788	PPP1R7	Protein phosphatase 1, regulatory (inhibitor) subunit 7
336	IMAGp998B07725	0.00	0.638	PACSIN2	Protein kinase C and casein kinase substrate in neurons 2
337	IMAGp998D10977	0.00	0.664	N/A	Data not found
338	RZPDp201D066D	0.00	0.693	CYP3A4	Cytochrome P450, family 3, subfamily A, polypeptide 4
339	IMAGp998P13679	0.00	0.716	LRRFIP2	Leucine rich repeat (in FLII) interacting protein 2
340	IMAGp998F11419	0.00	0.789	FCGRT	Fc fragment of IgG, receptor, transporter, alpha
341	IMAGp998F073056	0.00	0.620	N/A	Transcribed locus
342	RZPDp1096D032D	0.00	0.730	N/A	CDNA clone IMAGE:4902949
343	RZPDp202B091D	0.00	0.690	TRPM8	Transient receptor potential cation channel, subfamily M, member 8
344	IMAGp998J14656	0.00	0.588	C6orf111	Chromosome 6 open reading frame 111
345	IMAGp998A126075	0.00	0.861	GPR161	G protein-coupled receptor 161
346	IMAGp998K235618	0.00	0.641	RHOV	Ras homolog gene family, member V
347	IMAGp998E08598	0.00	0.832	GNPTAB	N-acetylglucosamine-1-phosphate transferase, alpha and beta subunits
348	IMAGp998N08792	0.00	0.797	ING1	Inhibitor of growth family, member 1
349	IMAGp998O20401	0.00	0.687	RBM9	RNA binding motif protein 9
350	IMAGp998D09273	0.00	0.810	C16orf53	Chromosome 16 open reading frame 53
351	IMAGp998N19535	0.00	0.805	N/A	CDNA FLJ38546 fis, clone HCHON2001646
352	IMAGp998M091852	0.00	0.616	N/A	Data not found
353	RZPDp201C016D	0.00	0.795	ACOXL	Acyl-Coenzyme A oxidase-like
354	IMAGp998A211017	0.00	0.712	N/A	Transcribed locus
355	IMAGp998F07200	0.00	0.866	GPR56	G protein-coupled receptor 56
356	IMAGp998E24156	0.00	0.690	TBC1D25	TBC1 domain family, member 25
357	IMAGp998H03794	0.00	0.684	SDC4	Syndecan 4
358	IMAGp998M14689	0.00	0.816	MAST4	Microtubule associated serine/threonine kinase family member 4
359	RZPDp202D103D	0.00	0.792	LONP1	Lon peptidase 1, mitochondrial
360	IMAGp998K03165	0.00	0.630	N/A	Transcribed locus
361	RZPDp201D013D	0.00	0.703	AKAP1	A kinase (PRKA) anchor protein 1
362	IMAGp998C19145	0.00	0.836	MEIS2	Meis homeobox 2
363	RZPDp201A014D	0.00	0.775	AMT	Aminomethyltransferase
364	IMAGp998F13205	0.00	0.735	N/A	Data not found
365	IMAGp998K166119	0.00	0.651	N/A	Transcribed locus
366	IMAGp998E17110	0.00	0.799	STAT6	Signal transducer and activator of transcription 6, interleukin-4 induced
367	IMAGp998G241784	0.00	0.794	PKD1	Polycystic kidney disease 1 (autosomal dominant)
368	RZPDp202H037D	0.00	0.713	RAB13	RAB13, member RAS oncogene family
369	IMAGp998L17533	0.00	0.820	NEU1	Sialidase 1 (lysosomal sialidase)
370	IMAGp998F18976	0.00	0.701	N/A	Data not found
371	IMAGp998L19534	0.00	0.772	TOM1L2	Target of myb1-like 2 (chicken)
372	IMAGp998N22296	0.00	0.830	DHRS4	Dehydrogenase/reductase (SDR family) member 4
373	IMAGp998D02462	0.00	0.671	N/A	Data not found
374	IMAGp998P241904	0.00	0.742	BTBD6	BTB (POZ) domain containing 6
375	IMAGp998D06161	0.00	0.753	HBEGF	Heparin-binding EGF-like growth factor
376	IMAGp998F19380	0.00	0.828	RING1	Ring finger protein 1
377	IMAGp998M14786	0.00	0.745	SELM	Selenoprotein M
378	IMAGp998J02135	0.00	0.790	SPAG7	Sperm associated antigen 7
379	RZPDp1096G1013D	0.00	0.688	LOC124220	Similar to common salivary protein 1
380	IMAGp998D13589	0.00	0.765	LOC283378	Hypothetical protein LOC283378
381	IMAGp998J235624	0.00	0.717	SLC14A1	Solute carrier family 14 (urea transporter), member 1 (Kidd blood group)
382	IMAGp998E05373	0.00	0.769	N/A	CDNA: FLJ21199 fis, clone COL00235
383	RZPDp1096C1220D	0.00	0.682	ERC1	ELKS/RAB6-interacting/CAST family member 1
384	IMAGp998C16473	0.00	0.735	SLC25A37	Solute carrier family 25, member 37
385	RZPDp1096B0516D	0.00	0.839	PAF1	Paf1, RNA polymerase II associated factor, homolog (S. cerevisiae)
386	RZPDp1096C018D	0.00	0.763	GABARAPL1	GABA(A) receptor-associated protein like 1
387	IMAGp998A09338	0.00	0.747	DDR1	Discoidin domain receptor family, member 1
388	IMAGp998I14117	0.00	0.735	GRN	Granulin
389	RZPDp201C024D	0.00	0.801	LOC728477	Similar to Golgi autoantigen, golgin subfamily a, 2
390	RZPDp1096E033D	0.00	0.859	SLC14A1	Solute carrier family 14 (urea transporter), member 1 (Kidd blood group)
391	RZPDp202H113D	0.00	0.694	CTTN	Cortactin
392	IMAGp998C21172	0.00	0.858	LSAMP	Limbic system-associated membrane protein
393	IMAGp998J225199	0.00	0.620	TMPRSS2	Transmembrane protease, serine 2
394	RZPDp201A1115D	0.00	0.739	VPS13D	Vacuolar protein sorting 13 homolog D (S. cerevisiae)
395	IMAGp998E14244	0.00	0.745	UNC84B	Unc-84 homolog B (C. elegans)
396	IMAGp998O18246	0.00	0.873	FLJ21865	Endo-beta-N-acetylglucosaminidase
397	IMAGp998F03388	0.00	0.746	N/A	CDNA clone IMAGE:4902949
398	IMAGp998J195510	0.00	0.760	N/A	Transcribed locus
399	IMAGp998F061815	0.00	0.822	CTSF	Cathepsin F
400	IMAGp998K24349	0.00	0.655	HLA-DRB1	Major histocompatibility complex, class II, DR beta 1
401	RZPDp1096B042D	0.00	0.793	RXRA	Retinoid X receptor, alpha
402	IMAGp998M14789	0.00	0.836	UBXD1	UBX domain containing 1
403	IMAGp998I23123	0.00	0.735	CLDN4	Claudin 4
404	IMAGp998E16348	0.00	0.824	N/A	CDNA: FLJ22566 fis, clone HSI01980
405	IMAGp998L13735	0.00	0.764	FHL2	Four and a half LIM domains 2

406	RZPDp202H104D	0.00	0.685	*LOC388588*	Hypothetical gene supported by BC035379; BC042129
407	IMAGp998N075574	0.00	0.792	*N/A*	CDNA FLJ14139 fis, clone MAMMA1002830
408	IMAGp998I184251	0.00	0.785	*KIAA1546*	KIAA1546
409	IMAGp998I11171	0.00	0.824	*CHD8*	Chromodomain helicase DNA binding protein 8
410	IMAGp998I055632	0.00	0.893	*CELSR1*	Cadherin, EGF LAG seven-pass G-type receptor 1 (flamingo homolog, Drosophila)
411	IMAGp998F23844	0.00	0.777	*LOC285548*	Hypothetical protein LOC285548
412	IMAGp998C201962	0.00	0.850	*SLC2A5*	Solute carrier family 2 (facilitated glucose/fructose transporter), member 5
413	IMAGp998C17245	0.00	0.852	*SERINC2*	Serine incorporator 2
414	IMAGp998I1581	0.00	0.676	*FOXO3A*	Forkhead box O3A
415	IMAGp998P041864	0.00	0.666	*LOC440905*	Hypothetical protein LOC440905
416	IMAGp998B14123	0.00	0.864	*ACP5*	Acid phosphatase 5, tartrate resistant
417	IMAGp998K114539	0.00	0.883	*N/A*	Transcribed locus
418	IMAGp998D1075	0.00	0.728	*FLJ10159*	Hypothetical protein FLJ10159
419	IMAGp998C24738	0.00	0.866	*ZBTB4*	Zinc finger and BTB domain containing 4
420	RZPDp201B0625D	0.00	0.789	*ATF3*	Activating transcription factor 3
421	IMAGp998D20594	0.00	0.882	*MAPK8IP3*	Mitogen-activated protein kinase 8 interacting protein 3
422	IMAGp998I19118	0.00	0.696	*N/A*	Data not found
423	IMAGp998F025457	0.00	0.729	*N/A*	Transcribed locus
424	IMAGp998A21229	0.00	0.784	*ABP1*	Amiloride binding protein 1 (amine oxidase (copper-containing))
425	IMAGp998K192010	0.00	0.721	*N/A*	Data not found
426	RZPDp201C1032D	0.00	0.752	*BLVRB*	Biliverdin reductase B (flavin reductase (NADPH))
427	RZPDp1096F1014D	0.00	0.846	*KCNH7*	Potassium voltage-gated channel, subfamily H (eag-related), member 7
428	IMAGp998J22828	0.00	0.666	*ZNF540*	Zinc finger protein 540
429	IMAGp998B20113	0.00	0.740	*ALDH4A1*	Aldehyde dehydrogenase 4 family, member A1
430	IMAGp998A201906	0.00	0.827	*EFNA1*	Ephrin-A1
431	IMAGp998C03160	0.00	0.830	*NXF1*	Nuclear RNA export factor 1
432	IMAGp998G11239	0.00	0.875	*LYNX1*	Ly6/neurotoxin 1
433	IMAGp998H13524	0.00	0.612	*N/A*	In multiple clusters
434	IMAGp998N181852	0.00	0.871	*D2HGDH*	D-2-hydroxyglutarate dehydrogenase
435	IMAGp998F09407	0.00	0.750	*N/A*	Data not found
436	IMAGp998C04659	0.00	0.761	*HSPB8*	Heat shock 22kDa protein 8
437	IMAGp998P16654	0.00	0.733	*DZIP3*	Zinc finger DAZ interacting protein 3
438	IMAGp998P055468	0.00	0.719	*FLJ22374*	Hypothetical protein FLJ22374
439	IMAGp998L175489	0.00	0.728	*N/A*	Transcribed locus
440	IMAGp998O125577	0.00	0.749	*HOXB13*	Homeobox B13
441	IMAGp998A072456	0.00	0.680	*TGM4*	Transglutaminase 4 (prostate)
442	IMAGp998D10143	0.00	0.771	*DPYSL3*	Dihydropyrimidinase-like 3
443	IMAGp998H18139	0.00	0.622	*FOS*	V-fos FBJ murine osteosarcoma viral oncogene homolog
444	IMAGp998G1918	0.00	0.735	*N/A*	Data not found
445	IMAGp998B23166	0.00	0.835	*TMBIM1*	Transmembrane BAX inhibitor motif containing 1
446	IMAGp998M164500	0.00	0.855	*RTDR1*	Rhabdoid tumor deletion region gene 1
447	IMAGp998L23165	0.00	0.857	*TRIP10*	Thyroid hormone receptor interactor 10
448	IMAGp998P011858	0.00	0.812	*PLXNB1*	Plexin B1
449	IMAGp998D104416	0.00	0.801	*N/A*	Transcribed locus
450	IMAGp998D22407	0.00	0.628	*AZGP1*	Alpha-2-glycoprotein 1, zinc-binding
451	IMAGp998I10526	0.00	0.830	*N/A*	Data not found
452	IMAGp998O13168	0.00	0.808	*VGLL4*	Vestigial like 4 (Drosophila)
453	IMAGp998J054158	0.00	0.779	*N/A*	Transcribed locus
454	IMAGp998H13516	0.00	0.854	*RXRA*	Retinoid X receptor, alpha
455	IMAGp998H23652	0.00	0.824	*KIAA0355*	KIAA0355
456	IMAGp998L07469	0.00	0.817	*ZFX*	Zinc finger protein, X-linked
457	IMAGp998H20868	0.00	0.776	*TGM7*	Transglutaminase 7
458	IMAGp998I06231	0.00	0.814	*RAB1A*	RAB1A, member RAS oncogene family
459	RZPDp201G0633D	0.00	0.836	*MGC7036*	Hypothetical protein MGC7036
460	IMAGp998L081858	0.00	0.870	*CAMK2G*	Calcium/calmodulin-dependent protein kinase (CaM kinase) II gamma
461	IMAGp998L03661	0.00	0.574	*N/A*	Transcribed locus
462	IMAGp998M0977	0.00	0.786	*TUBGCP2*	Tubulin, gamma complex associated protein 2
463	IMAGp998F201004	0.00	0.828	*N/A*	Transcribed locus
464	IMAGp998O01287	0.00	0.704	*SYT17*	Synaptotagmin XVII
465	RZPDp202D023D	0.00	0.820	*ACTN4*	Actinin, alpha 4
466	IMAGp998F22656	0.00	0.820	*NUDC*	Nuclear distribution gene C homolog (A. nidulans)
467	IMAGp998H142974	0.00	0.844	*N/A*	CDNA FLJ13825 fis, clone THYRO1000558
468	IMAGp998D175290	0.00	0.756	*GBP2*	Guanylate binding protein 2, interferon-inducible
469	IMAGp998N085601	0.00	0.752	*ID2*	Inhibitor of DNA binding 2, dominant negative helix-loop-helix protein
470	RZPDp202C055D	0.00	0.786	*LRPAP1*	Low density lipoprotein receptor-related protein associated protein 1
471	IMAGp998N09242	0.00	0.806	*NUMA1*	Nuclear mitotic apparatus protein 1
472	IMAGp998N115997	0.00	0.848	*N/A*	Transcribed locus
473	RZPDp1096A0916D	0.00	0.799	*LONP1*	Lon peptidase 1, mitochondrial
474	IMAGp998E14241	0.00	0.859	*STARD3*	START domain containing 3
475	IMAGp998A06784	0.00	0.745	*FUS*	Fusion (involved in t(12;16) in malignant liposarcoma)
476	RZPDp201G1016D	0.00	0.766	*IMPAD1*	Inositol monophosphatase domain containing 1
477	IMAGp998D14885	0.00	0.808	*N/A*	Data not found
478	RZPDp201C0934D	0.00	0.831	*VAC14*	Vac14 homolog (S. cerevisiae)
479	IMAGp998K18780	0.00	0.768	*LMAN2*	Lectin, mannose-binding 2
480	IMAGp998K191161	0.00	0.767	*ACOT4*	Acyl-CoA thioesterase 4
481	RZPDp201E0118D	0.00	0.770	*DDX27*	DEAD (Asp-Glu-Ala-Asp) box polypeptide 27
482	RZPDp202F044D	0.00	0.873	*TPCN1*	Two pore segment channel 1
483	IMAGp998I04193	0.00	0.714	*FLJ43390*	Hypothetical LOC646113
484	RZPDp1096G097D	0.00	0.786	*PTN*	Pleiotrophin (heparin binding growth factor 8, neurite growth-promoting factor 1)
485	IMAGp998N24160	0.00	0.817	*WDR16*	WD repeat domain 16
486	IMAGp998A195854	0.00	0.717	*N/A*	Transcribed locus
487	RZPDp201A0617D	0.00	0.824	*COG8*	Component of oligomeric golgi complex 8
488	IMAGp998J13153	0.00	0.844	*MEF2D*	MADS box transcription enhancer factor 2, polypeptide D (myocyte enhancer factor 2D)
489	RZPDp202A026D	0.00	0.719	*LOC162427*	Hypothetical protein LOC162427
490	RZPDp1096A084D	0.00	0.840	*LLGL2*	Lethal giant larvae homolog 2 (Drosophila)
491	RZPDp1096D023D	0.00	0.814	*PABPN1*	Poly(A) binding protein, nuclear 1
492	IMAGp998I141859	0.00	0.851	*LLGL2*	Lethal giant larvae homolog 2 (Drosophila)
493	IMAGp998P22201	0.00	0.780	*SASH1*	SAM and SH3 domain containing 1
494	RZPDp201B061D	0.00	0.745	*SLC18A2*	Solute carrier family 18 (vesicular monoamine), member 2
495	IMAGp998N05673	0.00	0.816	*PABPN1*	Poly(A) binding protein, nuclear 1
496	RZPDp201H115D	0.00	0.860	*N/A*	CDNA FLJ90129 fis, clone HEMBB1000309
497	IMAGp998D155387	0.00	0.774	*PMS2L5*	Postmeiotic segregation increased 2-like 5
498	IMAGp998M241999	0.00	0.881	*ASCC2*	Activating signal cointegrator 1 complex subunit 2
499	IMAGp998B045571	0.00	0.659	*GDEP*	Gene differentially expressed in prostate
500	RZPDp201D0927D	0.00	0.814	*DYNC1LI2*	Dynein, cytoplasmic 1, light intermediate chain 2
501	IMAGp998P231815	0.00	0.794	*MX1*	Myxovirus (influenza virus) resistance 1, interferon-inducible protein p78 (mouse)
502	IMAGp998B09121	0.00	0.763	*DNAPTP6*	DNA polymerase-transactivated protein 6
503	IMAGp998L095323	0.00	0.797	*N/A*	Transcribed locus, moderately similar to NP_663570.1 reductase 4 [Mus musculus]
504	IMAGp998I03176	0.00	0.698	*CRYM*	Crystallin, mu
505	IMAGp998O12221	0.00	0.743	*N/A*	Transcribed locus
506	IMAGp998G084652	0.00	0.743	*N/A*	CDNA FLJ39316 fis, clone OCBBF2014052
507	IMAGp998B095833	0.00	0.892	*TCEAL6*	Transcription elongation factor A (SII)-like 6
508	RZPDp1096D1118D	0.00	0.713	*MT1X*	Metallothionein 1X
509	IMAGp998E065587	0.00	0.803	*MAP4K5*	Mitogen-activated protein kinase kinase kinase kinase 5
510	IMAGp998G081935	0.00	0.818	*DDIT3*	DNA-damage-inducible transcript 3
511	IMAGp998O041942	0.00	0.873	*CTNNBIP1*	Catenin, beta interacting protein 1
512	IMAGp998H24247	0.00	0.839	*KCNE1*	Potassium voltage-gated channel, Isk-related family, member 1
513	IMAGp998C23825	0.00	0.822	*LMNA*	Lamin A/C
514	IMAGp998F12338	0.00	0.707	*EGR1*	Early growth response 1
515	IMAGp998M151005	0.00	0.774	*N/A*	Data not found
516	IMAGp998E13235	0.00	0.873	*DNMT3A*	DNA (cytosine-5-)-methyltransferase 3 alpha
517	IMAGp998M13166	0.00	0.853	*ADCY2*	Adenylate cyclase 2 (brain)
518	IMAGp998D02115	0.00	0.760	*N/A*	Data not found
519	IMAGp998C11138	0.00	0.800	*RETSAT*	Retinol saturase (all-trans-retinol 13,14-reductase)
520	IMAGp998M20284	0.00	0.808	*HIVEP2*	Human immunodeficiency virus type I enhancer binding protein 2
521	IMAGp998F026074	0.00	0.816	*WIF1*	WNT inhibitory factor 1
522	IMAGp998H09274	0.00	0.833	*SHC2*	SHC (Src homology 2 domain containing) transforming protein 2
523	IMAGp998E242228	0.00	0.707	*N/A*	Transcribed locus
524	IMAGp998L191010	0.00	0.758	*N/A*	Data not found
525	IMAGp998G09159	0.00	0.744	*ADAMTS1*	ADAM metallopeptidase with thrombospondin type 1 motif, 1
526	RZPDp201A0916D	0.00	0.782	*CACNA1G*	Calcium channel, voltage-dependent, T type, alpha 1G subunit
527	IMAGp998I15342	0.00	0.839	*APBB1*	Amyloid beta (A4) precursor protein-binding, family B, member 1 (Fe65)
528	IMAGp998C14840	0.00	0.777	*N/A*	Data not found
529	IMAGp998M211898	0.00	0.897	*KIAA0913*	KIAA0913
530	IMAGp998I051156	0.00	0.799	*N/A*	Transcribed locus
531	IMAGp998K09651	0.00	0.834	*NFE2L1*	Nuclear factor (erythroid-derived 2)-like 1
532	IMAGp998A19821	0.00	0.406	*N/A*	Full length insert cDNA clone ZE01A04
533	IMAGp998C144648	0.00	0.799	*N/A*	Transcribed locus
534	IMAGp998H22532	0.00	0.740	*FAM109B*	Family with sequence similarity 109, member B
535	IMAGp998K20677	0.00	0.847	*SIDT2*	SID1 transmembrane family, member 2
536	IMAGp998P05978	0.00	0.857	*N/A*	Data not found
537	IMAGp998J141008	0.00	0.805	*CHD7*	Chromodomain helicase DNA binding protein 7
538	RZPDp201B0915D	0.00	0.571	*CENPN*	Centromere protein N
539	IMAGp998C234462	0.00	0.861	*N/A*	Transcribed locus
540	IMAGp998B21183	0.00	0.782	*KCNK7*	Potassium channel, subfamily K, member 7

541	IMAGp998I171817	0.00	0.456	*SCAMP4*	Secretory carrier membrane protein 4
542	IMAGp998H22667	0.00	0.806	*SNRPN*	Small nuclear ribonucleoprotein polypeptide N
543	IMAGp998K12649	0.00	0.819	*N/A*	Full length insert cDNA clone ZA04F06
544	RZPDp202E101D	0.00	0.826	*FAAH*	Fatty acid amide hydrolase
545	RZPDp201G0528D	0.00	0.780	*N/A*	Data not found
546	IMAGp998K23540	0.00	0.799	*LOC124446*	Hypothetical protein BC017488
547	IMAGp998K17601	0.00	0.812	*LOC339123*	Hypothetical LOC339123
548	IMAGp998B22237	0.16	0.802	*N/A*	Full-length cDNA clone CS0DH005YI18 of T cells (Jurkat cell line) of Homo sapiens (human)
549	IMAGp998E05168	0.16	0.727	*RTN4*	Reticulon 4
550	IMAGp998L12830	0.16	0.739	*N/A*	Data not found
551	RZPDp201A0633D	0.16	0.812	*IRF3*	Interferon regulatory factor 3
552	RZPDp1096H1113D	0.16	0.894	*GABRP*	Gamma-aminobutyric acid (GABA) A receptor, pi
553	IMAGp998J174997	0.16	0.833	*KLF12*	Kruppel-like factor 12
554	RZPDp201E0826D	0.16	0.817	*PDCD11*	Programmed cell death 11
555	IMAGp998M16131	0.16	0.795	*N/A*	Data not found
556	RZPDp202D112D	0.16	0.817	*TMC4*	Transmembrane channel-like 4
557	IMAGp998M08783	0.16	0.871	*N/A*	Transcribed locus
558	IMAGp998A08269	0.16	0.757	*GAP43*	Growth associated protein 43
559	IMAGp998C18461	0.16	0.821	*COVA1*	Cytosolic ovarian carcinoma antigen 1
560	IMAGp998B17172	0.16	0.822	*FHOD3*	Formin homology 2 domain containing 3
561	RZPDp201E0327D	0.16	0.800	*HERC2P4*	Hect domain and RLD 2 pseudogene 4
562	RZPDp201F064D	0.16	0.862	*PALMD*	Palmdelphin
563	IMAGp998D03146	0.16	0.824	*NAP1L4*	Nucleosome assembly protein 1-like 4
564	IMAGp998N071747	0.16	0.758	*N/A*	Transcribed locus
565	RZPDp202D053D	0.16	0.812	*FLJ31568*	FLJ31568 protein
566	IMAGp998L063066	0.16	0.879	*RHOV*	Ras homolog gene family, member V
567	IMAGp998A166099	0.16	0.788	*N/A*	Transcribed locus
568	IMAGp998L041825	0.16	0.769	*NUDT10*	Nudix (nucleoside diphosphate linked moiety X)-type motif 10
569	IMAGp998F16827	0.16	0.807	*N/A*	Transcribed locus
570	IMAGp998C131725	0.16	0.790	*N/A*	Transcribed locus
571	IMAGp998H16312	0.16	0.891	*N/A*	Transcribed locus
572	RZPDp201G1115D	0.16	0.833	*TMCC2*	Transmembrane and coiled-coil domain family 2
573	IMAGp998K24264	0.16	0.800	*SERPING1*	Serpin peptidase inhibitor, clade G (C1 inhibitor), member 1, (angioedema, hereditary)
574	IMAGp998N23281	0.16	0.864	*PRMT2*	Protein arginine methyltransferase 2
575	IMAGp998I091789	0.16	0.904	*FBXW4*	F-box and WD repeat domain containing 4
576	IMAGp998C08143	0.16	0.820	*NGFR*	Nerve growth factor receptor (TNFR superfamily, member 16)
577	IMAGp998A196055	0.16	0.768	*N/A*	Transcribed locus
578	IMAGp998L21392	0.16	0.745	*N/A*	Data not found
579	IMAGp998J03121	0.16	0.798	*C15orf29*	Chromosome 15 open reading frame 29
580	IMAGp998O155601	0.16	0.812	*N/A*	Transcribed locus
581	RZPDp1096F052D	0.16	0.711	*EGR1*	Early growth response 1
582	RZPDp202H125D	0.16	0.867	*QPRT*	Quinolinate phosphoribosyltransferase (nicotinate-nucleotide pyrophosphorylase (carboxylating))
583	IMAGp998O181787	0.16	0.731	*TAGLN*	Transgelin
584	IMAGp998M021794	0.16	0.853	*N/A*	Data not found
585	IMAGp998P18280	0.16	0.750	*N/A*	Clone 23948 mRNA sequence
586	IMAGp998A24474	0.16	0.743	*N/A*	PRO0132 protein, mRNA (cDNA clone MGC:22358 IMAGE:4722371)
587	IMAGp998E09362	0.16	0.815	*JMJD2A*	Jumonji domain containing 2A
588	IMAGp998D09368	0.16	0.797	*CAPN3*	Calpain 3, (p94)
589	IMAGp998M20572	0.16	0.766	*CCDC92*	Coiled-coil domain containing 92
590	IMAGp998M09113	0.16	0.812	*N/A*	Data not found
591	IMAGp998I01168	0.16	0.885	*HTATIP*	HIV-1 Tat interacting protein, 60kDa
592	IMAGp998N08240	0.16	0.862	*EXOC7*	Exocyst complex component 7
593	IMAGp998G245416	0.16	0.775	*N/A*	Transcribed locus
594	IMAGp998K01373	0.16	0.772	*MYCBP2*	MYC binding protein 2
595	RZPDp1096A058D	0.16	0.802	*GSTK1*	Glutathione S-transferase kappa 1
596	IMAGp998H18371	0.16	0.794	*N/A*	CDNA FLJ40669 fis, clone THYMU2020883
597	IMAGp998P23436	0.16	0.809	*ABLIM1*	Actin binding LIM protein 1
598	IMAGp998H144736	0.16	0.855	*N/A*	Transcribed locus
599	IMAGp998K19670	0.16	0.797	*ACTA1*	Actin, alpha 1, skeletal muscle
600	IMAGp998P101998	0.16	0.735	*BACE2*	Beta-site APP-cleaving enzyme 2
601	IMAGp998H08437	0.16	0.885	*N/A*	CDNA clone IMAGE:5294560
602	IMAGp998E083818	0.16	0.792	*NOPE*	Likely ortholog of mouse neighbor of Punc E11
603	IMAGp998D13520	0.16	0.879	*VAMP3*	Vesicle-associated membrane protein 3 (cellubrevin)
604	IMAGp998D10979	0.16	0.817	*SLC1A5*	Solute carrier family 1 (neutral amino acid transporter), member 5
605	IMAGp998E06370	0.16	0.759	*N/A*	Data not found
606	IMAGp998B031200	0.16	0.900	*XYLT2*	Xylosyltransferase II
607	IMAGp998O225109	0.16	0.838	*N/A*	Prohibitin pseudogene, mRNA (cDNA clone MGC:20874 IMAGE:4547239)
608	IMAGp998E15653	0.16	0.745	*N/A*	Transcribed locus
609	RZPDp201E0720D	0.16	0.646	*ZBTB16*	Zinc finger and BTB domain containing 16
610	IMAGp998G165198	0.16	0.830	*N/A*	Transcribed locus
611	IMAGp998B014337	0.16	0.870	*N/A*	Transcribed locus
612	IMAGp998J20280	0.16	0.835	*SLC25A29*	Solute carrier family 25, member 29
613	IMAGp998C101202	0.16	0.788	*NFIC*	Nuclear factor I/C (CCAAT-binding transcription factor)
614	IMAGp998H04781	0.16	0.836	*MAGED2*	Melanoma antigen family D, 2
615	IMAGp998O15653	0.16	0.619	*N/A*	Data not found
616	IMAGp998A24580	0.16	0.822	*WIPI2*	WD repeat domain, phosphoinositide interacting 2
617	IMAGp998B13329	0.16	0.790	*N/A*	Data not found
618	IMAGp998C23979	0.16	0.813	*N/A*	In multiple clusters
619	IMAGp998D11368	0.16	0.784	*INADL*	InaD-like (Drosophila)
620	IMAGp998D075210	0.16	0.785	*C14orf2*	Chromosome 14 open reading frame 2
621	IMAGp998O24976	0.16	0.896	*N/A*	Data not found
622	IMAGp998H235164	0.16	0.695	*N/A*	Transcribed locus
623	IMAGp998L221782	0.16	0.781	*N/A*	Transcribed locus
624	IMAGp998B18399	0.16	0.757	*C22orf28*	Chromosome 22 open reading frame 28
625	IMAGp998G125382	0.24	0.771	*FOXP4*	Forkhead box P4
626	IMAGp998K075807	0.24	0.861	*TUBA8*	Tubulin, alpha 8
627	RZPDp1096F1213D	0.24	0.757	*HLA-DRB4*	Major histocompatibility complex, class II, DR beta 4
628	IMAGp998G07609	0.24	0.799	*N/A*	Data not found
629	RZPDp201A0427D	0.24	0.822	*CRLF3*	Cytokine receptor-like factor 3
630	IMAGp998H024725	0.24	0.834	*NAP1L4*	Nucleosome assembly protein 1-like 4
631	IMAGp998M191785	0.24	0.893	*DKFZP566E164*	DKFZP566E164 protein
632	RZPDp202D108D	0.24	0.801	*IK*	IK cytokine, down-regulator of HLA II
633	IMAGp998H232376	0.24	0.682	*N/A*	Transcribed locus
634	IMAGp998I085413	0.24	0.782	*N/A*	CDNA FLJ39413 fis, clone PLACE6015729
635	IMAGp998L125455	0.24	0.847	*N/A*	Full-length cDNA clone CS0DI022YE21 of Placenta Cot 25-normalized of Homo sapiens (human)
636	IMAGp998E07663	0.24	0.763	*N/A*	Transcribed locus
637	IMAGp998K19612	0.24	0.854	*NLRP1*	NLR family, pyrin domain containing 1
638	RZPDp201B0634D	0.24	0.758	*ACTN1*	Actinin, alpha 1
639	RZPDp202C041D	0.24	0.823	*FBXO17*	F-box protein 17
640	RZPDp201C1120D	0.24	0.827	*EBI3*	Epstein-Barr virus induced gene 3
641	IMAGp998P05214	0.24	0.784	*CALU*	Calumenin
642	IMAGp998M08183	0.24	0.834	*C1orf115*	Chromosome 1 open reading frame 115
643	IMAGp998M085380	0.24	0.842	*PPP3CB*	Protein phosphatase 3 (formerly 2B), catalytic subunit, beta isoform
644	RZPDp201B0833D	0.24	0.869	*HSP90AB1*	Heat shock protein 90kDa alpha (cytosolic), class B member 1
645	RZPDp201D0433D	0.24	0.842	*SETD5*	SET domain containing 5
646	IMAGp998G24281	0.24	0.853	*ARIH2*	Ariadne homolog 2 (Drosophila)
647	IMAGp998B204113	0.24	0.865	*LOC440518*	Similar to Golgin subfamily A member 8A/B (Golgi autoantigen golgin-67) (88 kDa Golgi protein) (Gm88 autoantigen)
648	IMAGp998K13520	0.24	0.839	*FAM44B*	Family with sequence similarity 44, member B
649	IMAGp998A02423	0.24	0.833	*N/A*	Transcribed locus, weakly similar to XP_512323.2 insulin receptor, partial [Pan troglodytes]
650	IMAGp998G23650	0.24	0.824	*RIC8A*	Resistance to inhibitors of cholinesterase 8 homolog A (C. elegans)
651	RZPDp202C056D	0.24	0.818	*TP53I13*	Tumor protein p53 inducible protein 13
652	IMAGp998B125496	0.24	0.809	*CARD6*	Caspase recruitment domain family, member 6
653	IMAGp998P04149	0.24	0.831	*POR*	P450 (cytochrome) oxidoreductase
654	IMAGp998E11743	0.24	0.887	*N/A*	Transcribed locus
655	RZPDp202B117D	0.24	0.847	*PGPEP1*	Pyroglutamyl-peptidase I
656	IMAGp998F101114	0.24	0.815	*N/A*	Transcribed locus
657	RZPDp201F1232D	0.24	0.831	*SEC24D*	SEC24 related gene family, member D (S. cerevisiae)
658	IMAGp998O221817	0.24	0.759	*NRSN2*	Neurensin 2
659	IMAGp998K181157	0.24	0.848	*USP4*	Ubiquitin specific peptidase 4 (proto-oncogene)
660	IMAGp998D12110	0.24	0.818	*N/A*	Data not found
661	IMAGp998P081781	0.24	0.896	*SNCG*	Synuclein, gamma (breast cancer-specific protein 1)
662	IMAGp998E22462	0.24	0.812	*N/A*	Data not found
663	RZPDp202F086D	0.24	0.882	*VPS37B*	Vacuolar protein sorting 37 homolog B (S. cerevisiae)
664	IMAGp998E20624	0.24	0.825	*N/A*	Transcribed locus
665	RZPDp201H1120D	0.24	0.847	*RUFY3*	RUN and FYVE domain containing 3
666	IMAGp998E073323	0.24	0.776	*N/A*	Transcribed locus
667	RZPDp1096H1216D	0.24	0.863	*TOP1MT*	Topoisomerase (DNA) I, mitochondrial
668	RZPDp1096C0617D	0.24	0.822	*MKNK2*	MAP kinase interacting serine/threonine kinase 2
669	IMAGp998B131005	0.24	0.773	*GINS1*	GINS complex subunit 1 (Psf1 homolog)
670	IMAGp998P09779	0.24	0.802	*N/A*	In multiple clusters
671	IMAGp998I245983	0.24	0.821	*ALOX15B*	Arachidonate 15-lipoxygenase, type B
672	IMAGp998K081008	0.24	0.865	*N/A*	Transcribed locus
673	IMAGp998I18252	0.24	0.838	*SYTL1*	Synaptotagmin-like 1
674	IMAGp998B034147	0.32	0.813	*MAST4*	Microtubule associated serine/threonine kinase family member 4
675	IMAGp998M23538	0.32	0.629	*BRD3*	Bromodomain containing 3

676	IMAGp998L051781	0.32	0.903	*CRYAB*	Crystallin, alpha B
677	IMAGp998B04836	0.32	0.871	*KLF6*	Kruppel-like factor 6
678	IMAGp998E014326	0.32	0.876	*LAMP1*	Lysosomal-associated membrane protein 1
679	IMAGp998L121157	0.32	0.840	*CD59*	CD59 molecule, complement regulatory protein
680	RZPDp1096C1216D	0.32	0.857	*COTL1*	Coactosin-like 1 (Dictyostelium)
681	RZPDp201A0235D	0.32	0.850	*SAPS1*	SAPS domain family, member 1
682	IMAGp998C12135	0.32	0.767	*N/A*	Transcribed locus, moderately similar to XP_508432.1 similar to solute carrier family 43, member 1
683	IMAGp998A01469	0.32	0.884	*PIP5K1C*	Phosphatidylinositol-4-phosphate 5-kinase, type I, gamma
684	RZPDp201C031D	0.32	0.824	*ZNF514*	Zinc finger protein 514
685	IMAGp998K145595	0.32	0.891	*N/A*	Transcribed locus
686	IMAGp998G15611	0.32	0.813	*COLEC12*	Collectin sub-family member 12
687	IMAGp998L081860	0.32	0.881	*ELF3*	E74-like factor 3 (ets domain transcription factor, epithelial-specific)
688	IMAGp998E221905	0.32	0.817	*N/A*	Transcribed locus
689	IMAGp998N074518	0.32	0.820	*N/A*	Data not found
690	RZPDp201G041D	0.32	0.843	*SLITRK3*	SLIT and NTRK-like family, member 3
691	RZPDp201B0236D	0.32	0.781	*PSMB8*	Proteasome (prosome, macropain) subunit, beta type, 8 (large multifunctional peptidase 7)
692	IMAGp998N025289	0.32	0.841	*SH2D3C*	SH2 domain containing 3C
693	IMAGp998B06650	0.32	0.849	*PEX26*	Peroxisome biogenesis factor 26
694	IMAGp998M04362	0.32	0.797	*N/A*	CDNA clone IMAGE:5294683
695	IMAGp998H094140	0.32	0.805	*ZNF552*	Zinc finger protein 552
696	IMAGp998F175814	0.32	0.806	*RPL31*	Ribosomal protein L31
697	IMAGp998J171858	0.32	0.872	*TNS1*	Tensin 1
698	IMAGp998F21232	0.32	0.879	*MAF*	V-maf musculoaponeurotic fibrosarcoma oncogene homolog (avian)
699	IMAGp998F07163	0.32	0.830	*CAND2*	Cullin-associated and neddylation-dissociated 2 (putative)
700	IMAGp998F19780	0.32	0.867	*N/A*	In multiple clusters
701	IMAGp998H21823	0.32	0.854	*FLJ39378*	Hypothetical protein FLJ39378
702	IMAGp998I22178	0.32	0.822	*UBC*	Ubiquitin C
703	IMAGp998M20261	0.32	0.820	*PILRA*	Paired immunoglobin-like type 2 receptor alpha
704	IMAGp998B01872	0.32	0.834	*N/A*	Transcribed locus, weakly similar to XP_519878.1
705	IMAGp998J20594	0.32	0.854	*IGSF3*	Immunoglobulin superfamily, member 3
706	IMAGp998D06543	0.32	0.810	*DHRS1*	Dehydrogenase/reductase (SDR family) member 1
707	IMAGp998M032305	0.32	0.811	*STARD4*	START domain containing 4, sterol regulated
708	IMAGp998I025671	0.32	0.897	*GABRP*	Gamma-aminobutyric acid (GABA) A receptor, pi
709	IMAGp998L01146	0.32	0.802	*N/A*	CDNA FLJ30378 fis, clone BRACE2007953
710	IMAGp998K115786	0.32	0.802	*HOXA11S*	Homeo box A11, antisense
711	IMAGp998I141857	0.32	0.834	*IFNAR1*	Interferon (alpha, beta and omega) receptor 1
712	IMAGp998M011897	0.32	0.883	*C1orf160*	Chromosome 1 open reading frame 160
713	IMAGp998G13163	0.32	0.818	*SRF*	Serum response factor (c-fos serum response element-binding transcription factor)
714	IMAGp998O10119	0.32	0.804	*VCP*	Valosin-containing protein
715	IMAGp998I111008	0.49	0.788	*CIDEC*	Cell death-inducing DFFA-like effector c
716	IMAGp998F09120	0.49	0.842	*MBTPS1*	Membrane-bound transcription factor peptidase, site 1
717	IMAGp998I042004	0.49	0.873	*PACS1*	Phosphofurin acidic cluster sorting protein 1
718	IMAGp998F175381	0.49	0.821	*LMBR1L*	Limb region 1 homolog (mouse)-like
719	IMAGp998M161859	0.49	0.879	*ENTPD2*	Ectonucleoside triphosphate diphosphohydrolase 2
720	IMAGp998J11518	0.49	0.912	*N/A*	Transcribed locus
721	IMAGp998B21274	0.49	0.897	*PVRL1*	Poliovirus receptor-related 1 (herpesvirus entry mediator C; nectin)
722	RZPDp1096E0914D	0.49	0.810	*TRPC4*	Transient receptor potential cation channel, subfamily C, member 4
723	RZPDp201E0915D	0.49	0.874	*ATPBD3*	ATP binding domain 3
724	IMAGp998N186087	0.49	0.674	*N/A*	Transcribed locus
725	IMAGp998F14581	0.49	0.862	*TRPM1*	Transient receptor potential cation channel, subfamily M, member 1
726	IMAGp998H144077	0.49	0.774	*N/A*	Homo sapiens, clone IMAGE:5241654, mRNA
727	IMAGp998P164889	0.49	0.858	*RBCK1*	RanBP-type and C3HC4-type zinc finger containing 1
728	IMAGp998O16237	0.49	0.911	*TYROBP*	TYRO protein tyrosine kinase binding protein
729	IMAGp998G17218	0.49	0.911	*FLJ43663*	Hypothetical protein FLJ43663
730	IMAGp998I195403	0.49	0.847	*C18orf37*	Chromosome 18 open reading frame 37
731	IMAGp998F232409	0.49	0.825	*VSIG9*	V-set and immunoglobulin domain containing 9
732	IMAGp998G145464	0.49	0.825	*TDRKH*	Tudor and KH domain containing
733	IMAGp998G04535	0.49	0.798	*DHX57*	DEAH (Asp-Glu-Ala-Asp/His) box polypeptide 57
734	RZPDp1096G057D	0.49	0.819	*C9orf140*	Chromosome 9 open reading frame 140
735	IMAGp998A07375	0.49	0.792	*PTCD3*	Pentatricopeptide repeat domain 3
736	IMAGp998J18593	0.49	0.811	*N/A*	Data not found
737	RZPDp202A016D	0.49	0.883	*ANXA8*	Annexin A8
738	IMAGp998N202227	0.49	0.832	*CXYorf3*	Chromosome X and Y open reading frame 3
739	IMAGp998D084030	0.49	0.885	*SCNM1*	Sodium channel modifier 1
740	RZPDp202C065D	0.49	0.820	*CHAF1A*	Chromatin assembly factor 1, subunit A (p150)
741	IMAGp998I21315	0.49	0.794	*BRUNOL5*	Bruno-like 5, RNA binding protein (Drosophila)
742	IMAGp998J105404	0.49	0.813	*N/A*	Transcribed locus
743	IMAGp998A09131	0.49	0.749	*GALNT10*	UDP-N-acetyl-alpha-D-galactosamine:polypeptide N-acetylgalactosaminyltransferase 10 (GalNAc-T10)
744	IMAGp998A16667	0.49	0.816	*TBX19*	T-box 19
745	IMAGp998O04273	0.49	0.781	*N/A*	Data not found
746	IMAGp998P015793	0.49	0.852	*N/A*	Data not found
747	IMAGp998E18235	0.49	0.856	*ZFYVE21*	Zinc finger, FYVE domain containing 21
748	IMAGp998A2488	0.49	0.801	*EFEMP1*	EGF-containing fibulin-like extracellular matrix protein 1
749	IMAGp998D10128	0.49	0.827	*SETD5*	SET domain containing 5
750	IMAGp998E152228	0.49	0.829	*N/A*	Transcribed locus
751	RZPDp202F056D	0.49	0.847	*HLA-F*	Major histocompatibility complex, class I, F
752	IMAGp998H085468	0.49	0.845	*FBXW7*	F-box and WD repeat domain containing 7
753	RZPDp1096H085D	0.49	0.886	*TMEM63C*	Transmembrane protein 63C
754	IMAGp998M18201	0.49	0.815	*NEIL1*	Nei endonuclease VIII-like 1 (E. coli)
755	IMAGp998I076119	0.49	0.919	*UPK3A*	Uroplakin 3A
756	IMAGp998E034543	0.49	0.881	*N/A*	Transcribed locus
757	IMAGp998A124199	0.49	0.825	*N/A*	Data not found
758	IMAGp998B06132	0.49	0.817	*N/A*	Data not found
759	RZPDp1096G108D	0.49	0.785	*RBMY1F*	RNA binding motif protein, Y-linked, family 1, member F
760	IMAGp998I162639	0.49	0.681	*N/A*	Data not found
761	IMAGp998L08169	0.49	0.826	*N/A*	CDNA FLJ44201 fis, clone THYMU3000841
762	RZPDp202D124D	0.49	0.794	*IL27RA*	Interleukin 27 receptor, alpha
763	IMAGp998B202402	0.49	0.805	*N/A*	Transcribed locus
764	RZPDp201H0516D	0.49	0.834	*HYPE*	Huntingtin interacting protein E
765	RZPDp1096F0314D	0.49	0.842	*SPC25*	SPC25, NDC80 kinetochore complex component, homolog (S. cerevisiae)
766	IMAGp998N214504	0.49	0.815	*N/A*	Transcribed locus
767	IMAGp998J094349	0.49	0.834	*N/A*	Transcribed locus
768	IMAGp998C05249	0.49	0.911	*PRR5*	Proline rich 5 (renal)
769	RZPDp201G064D	0.49	0.885	*RGS12*	Regulator of G-protein signalling 12
770	IMAGp998K031945	0.66	0.836	*CD1D*	CD1d molecule
771	IMAGp998P14978	0.66	0.849	*LOC731656*	Hypothetical protein LOC731656
772	IMAGp998D05522	0.66	0.806	*N/A*	Data not found
773	RZPDp202A071D	0.66	0.808	*GRIK4*	Glutamate receptor, ionotropic, kainate 4
774	IMAGp998E161860	0.66	0.858	*IGSF9*	Immunoglobulin superfamily, member 9
775	RZPDp1096A074D	0.66	0.918	*LYPD3*	LY6/PLAUR domain containing 3
776	IMAGp998N16624	0.66	0.824	*ATXN3*	Ataxin 3
777	RZPDp202F074D	0.66	0.815	*AMOT*	Angiomotin
778	IMAGp998G214548	0.66	0.780	*N/A*	Transcribed locus
779	IMAGp998H01976	0.66	0.901	*N/A*	Transcribed locus
780	IMAGp998P15788	0.66	0.852	*WDR5*	WD repeat domain 5
781	IMAGp998L041165	0.66	0.848	*IL10RB*	Interleukin 10 receptor, beta
782	RZPDp201B0916D	0.66	0.800	*LRRC8E*	Leucine rich repeat containing 8 family, member E
783	IMAGp998O241781	0.66	0.810	*NR4A1*	Nuclear receptor subfamily 4, group A, member 1
784	IMAGp998H222308	0.66	0.731	*N/A*	Transcribed locus
785	IMAGp998G205585	0.66	0.795	*LOC150166*	Hypothetical protein LOC150166
786	IMAGp998N21237	0.66	0.917	*MCF2L*	MCF.2 cell line derived transforming sequence-like
787	IMAGp998H15466	0.66	0.774	*N/A*	Transcribed locus
788	IMAGp998O244595	0.66	0.857	*SPTBN5*	Spectrin, beta, non-erythrocytic 5
789	IMAGp998I18168	0.66	0.814	*ARG2*	Arginase, type II
790	IMAGp998J03330	0.66	0.885	*FAM5B*	Family with sequence similarity 5, member B
791	IMAGp998I0373	0.66	0.836	*MAPK10*	Mitogen-activated protein kinase 10
792	IMAGp998D16205	0.66	0.896	*SF3B5*	Splicing factor 3b, subunit 5, 10kDa
793	IMAGp998N24277	0.66	0.908	*ARMC7*	Armadillo repeat containing 7
794	RZPDp201A0515D	0.66	0.799	*LOC729390*	Similar to DKFZP434B061 protein
795	IMAGp998B225164	0.66	0.809	*N/A*	Data not found
796	IMAGp998O123986	0.66	0.822	*CTSC*	Cathepsin C
797	RZPDp202D051D	0.66	0.834	*IMPG2*	Interphotoreceptor matrix proteoglycan 2
798	RZPDp201G0127D	0.66	0.873	*JARID2*	Jumonji, AT rich interactive domain 2
799	IMAGp998H091065	0.66	0.823	*C5*	Complement component 5
800	RZPDp1096F101D	0.66	0.783	*TEGT*	Testis enhanced gene transcript (BAX inhibitor 1)
801	IMAGp998M13462	0.66	0.857	*FAM118A*	Family with sequence similarity 118, member A
802	RZPDp201C091D	0.66	0.877	*HCCA2*	HCCA2 protein
803	IMAGp998I054862	0.66	0.829	*EXOSC6*	Exosome component 6
804	IMAGp998G015569	0.66	0.904	*CCDC102A*	Coiled-coil domain containing 102A
805	IMAGp998M01319	0.66	0.826	*N/A*	Full length insert cDNA clone YN86A01
806	IMAGp998B11793	0.66	0.852	*TBC1D16*	TBC1 domain family, member 16
807	IMAGp998P204991	0.66	0.820	*N/A*	Transcribed locus
808	IMAGp998K08371	0.66	0.813	*N/A*	Data not found
809	IMAGp998G205380	0.66	0.814	*N/A*	Clone pp7583 unknown mRNA
810	IMAGp998A09358	0.66	0.802	*C21orf13*	Chromosome 21 open reading frame 13

811	IMAGp998L14154	0.66	0.845	*PCDH10*	Protocadherin 10
812	IMAGp998M231786	0.66	0.909	*C14orf166B*	Chromosome 14 open reading frame 166B
813	IMAGp998F12559	0.66	0.852	*GSTA3*	Glutathione S-transferase A3
814	RZPDp201G1131D	0.66	0.881	*TPBG*	Trophoblast glycoprotein
815	IMAGp998G181785	0.66	0.835	*THRAP4*	Thyroid hormone receptor associated protein 4
816	IMAGp998K13692	0.66	0.846	*TTF1*	Transcription termination factor, RNA polymerase I
817	IMAGp998P231167	0.66	0.790	*NEO1*	Neogenin homolog 1 (chicken)
818	RZPDp201E0218D	0.66	0.821	*LRRC8E*	Leucine rich repeat containing 8 family, member E
819	RZPDp201E0333D	0.66	0.814	*ARPC1B*	Actin related protein 2/3 complex, subunit 1B, 41kDa
820	RZPDp201E0432D	0.66	0.845	*USP28*	Ubiquitin specific peptidase 28
821	IMAGp998M191743	0.66	0.844	*N/A*	Data not found
822	IMAGp998H142002	0.66	0.820	*ESCO2*	Establishment of cohesion 1 homolog 2 (S. cerevisiae)
823	IMAGp998N07974	0.66	0.863	*N/A*	Data not found
824	IMAGp998N07602	0.66	0.776	*N/A*	Transcribed locus
825	IMAGp998G111781	0.66	0.871	*NLRC5*	NLR family, CARD domain containing 5
826	IMAGp998M194890	0.66	0.885	*N/A*	Transcribed locus
827	RZPDp201F0828D	0.66	0.823	*HLA-DQB1*	Major histocompatibility complex, class II, DQ beta 1
828	IMAGp998A21515	0.66	0.900	*SLC20A2*	Solute carrier family 20 (phosphate transporter), member 2
829	IMAGp998P20366	0.66	0.341	*ORM2*	Orosomucoid 2
830	IMAGp998N075268	0.66	0.832	*NANS*	N-acetylneuraminic acid synthase (sialic acid synthase)
831	IMAGp998D114004	0.66	0.839	*ATXN3*	Ataxin 3
832	IMAGp998M045715	0.66	0.780	*DOCK10*	Dedicator of cytokinesis 10
833	IMAGp998H18334	0.66	0.805	*DLGAP1*	Discs, large (Drosophila) homolog-associated protein 1
834	RZPDp201G0615D	0.66	0.815	*PLEKHN1*	Pleckstrin homology domain containing, family N member 1
835	IMAGp998D231008	0.66	0.900	*TRAPPC3*	Trafficking protein particle complex 3
836	IMAGp998B09675	0.66	0.792	*CST6*	Cystatin E/M
837	IMAGp998J18726	0.66	0.862	*LITAF*	Lipopolysaccharide-induced TNF factor
838	IMAGp998E164900	0.66	0.899	*CARD9*	Caspase recruitment domain family, member 9
839	IMAGp998K144357	0.66	0.828	*N/A*	Transcribed locus
840	IMAGp998L1879	0.66	0.862	*SMOC1*	SPARC related modular calcium binding 1
841	IMAGp998P154336	0.66	0.817	*N/A*	Data not found
842	IMAGp998M225476	0.66	0.836	*SMAD1*	SMAD family member 1
843	IMAGp998N173853	0.66	0.805	*FBXO10*	F-box protein 10
844	IMAGp998K11273	0.66	0.822	*CNTN5*	Contactin 5
845	IMAGp998C104645	0.66	0.794	*N/A*	CDNA FLJ37989 fis, clone CTONG2011676
846	IMAGp998C11594	0.66	0.839	*N/A*	Transcribed locus
847	IMAGp998I241859	0.66	0.911	*SLC25A25*	Solute carrier family 25 (mitochondrial carrier; phosphate carrier), member 25
848	IMAGp998K21835	0.66	0.869	*N/A*	In multiple clusters
849	IMAGp998A131856	0.66	0.848	*SNRP70*	Small nuclear ribonucleoprotein 70kDa polypeptide (RNP antigen)
850	IMAGp998K045631	0.66	0.826	*N/A*	CDNA FLJ13722 fis, clone PLACE2000455
851	IMAGp998C151203	0.66	0.909	*FBXO18*	F-box protein, helicase, 18
852	IMAGp998L15195	0.66	0.878	*IFNGR1*	Interferon gamma receptor 1
853	IMAGp998L011783	0.66	0.876	*N/A*	KIAA1657 protein
854	IMAGp998P12387	0.66	0.822	*PLCG2*	Phospholipase C, gamma 2 (phosphatidylinositol-specific)
855	IMAGp998O155598	0.66	0.809	*DFNB31*	Deafness, autosomal recessive 31
856	IMAGp998J04209	0.66	0.785	*N/A*	Transcribed locus
857	IMAGp998D17422	0.66	0.799	*N/A*	Data not found
858	RZPDp1096F032D	0.66	0.853	*NR3C1*	Nuclear receptor subfamily 3, group C, member 1 (glucocorticoid receptor)
859	IMAGp998I101781	0.66	0.776	*C1orf116*	Chromosome 1 open reading frame 116
860	RZPDp202C114D	0.66	0.856	*TMEM86B*	Transmembrane protein 86B
861	IMAGp998M04829	0.66	0.851	*COG2*	Component of oligomeric golgi complex 2
862	IMAGp998I194608	0.66	0.920	*N/A*	Transcribed locus
863	IMAGp998M06124	0.66	0.848	*N/A*	Data not found
864	IMAGp998F03978	0.66	0.916	*KIAA1967*	KIAA1967
865	IMAGp998A104766	0.66	0.808	*N/A*	Transcribed locus
866	IMAGp998M221785	0.66	0.918	*LOC259308*	Hypothetical LOC259308
867	IMAGp998I011833	0.66	0.855	*DPP8,*	Dipeptidyl-peptidase 8,Transcribed locus
868	RZPDp202G053D	0.66	0.822	*CABP4*	Calcium binding protein 4
869	IMAGp998J131023	0.66	0.814	*N/A*	Data not found
870	IMAGp998A096083	0.66	0.846	*RNF121*	Ring finger protein 121
871	RZPDp201G0829D	0.66	0.801	*MYO1C*	Myosin IC
872	IMAGp998D105583	0.66	0.838	*SMOC2*	SPARC related modular calcium binding 2
873	RZPDp202C049D	0.84	0.902	*ABCC6*	ATP-binding cassette, sub-family C (CFTR/MRP), member 6
874	IMAGp998J155654	0.84	0.814	*CHRNB4*	Cholinergic receptor, nicotinic, beta 4
875	IMAGp998N151781	0.84	0.909	*NPEPL1*	Aminopeptidase-like 1
876	IMAGp998C2272	0.84	0.840	*N/A*	Transcribed locus
877	IMAGp998M14220	0.84	0.804	*POMT2*	Protein-O-mannosyltransferase 2
878	RZPDp202B017D	0.84	0.795	*DUSP8*	Dual specificity phosphatase 8
879	IMAGp998F2483	0.84	0.881	*N/A*	Data not found
880	IMAGp998E231725	0.84	0.813	*N/A*	Transcribed locus
881	IMAGp998M234596	0.84	0.835	*N/A*	Transcribed locus
882	IMAGp998G16444	0.84	0.882	*ABCA4*	ATP-binding cassette, sub-family A (ABC1), member 4
883	IMAGp998F215947	0.84	0.882	*N/A*	MRNA; cDNA DKFZp781M0898 (from clone DKFZp781M0898)
884	IMAGp998O11269	0.84	0.854	*C11orf41*	Chromosome 11 open reading frame 41
885	IMAGp998C181781	0.84	0.859	*TMEM109*	Transmembrane protein 109
886	IMAGp998M16208	0.84	0.927	*SULT2B1*	Sulfotransferase family, cytosolic, 2B, member 1
887	IMAGp998N24404	0.84	0.783	*N/A*	Transcribed locus
888	IMAGp998H21654	0.84	0.832	*N/A*	Data not found
889	IMAGp998D056082	0.84	0.839	*ZNF608*	Zinc finger protein 608
890	IMAGp998L13268	0.84	0.831	*MCAM*	Melanoma cell adhesion molecule
891	IMAGp998A11288	0.84	0.827	*SLA*	Src-like-adaptor
892	IMAGp998E135510	0.84	0.832	*ZNF83*	Zinc finger protein 83
893	IMAGp998N243806	0.84	0.877	*N/A*	Transcribed locus
894	RZPDp202B122D	0.84	0.899	*KRT33B*	Keratin 33B
895	IMAGp998I136119	0.84	0.802	*N/A*	Data not found
896	IMAGp998D04232	0.84	0.849	*LOC653319*	Hypothetical protein LOC653319
897	IMAGp998P192408	0.84	0.798	*PHF17*	PHD finger protein 17
898	IMAGp998B19566	0.84	0.918	*RAB24*	RAB24, member RAS oncogene family
899	IMAGp998O05871	0.84	0.902	*N/A*	Transcribed locus
900	IMAGp998D194496	0.84	0.917	*LOC645355*	Hypothetical LOC645355
901	IMAGp998O16524	0.84	0.828	*GOT2*	Glutamic-oxaloacetic transaminase 2, mitochondrial (aspartate aminotransferase 2)
902	IMAGp998F12366	0.84	0.797	*RPL23*	Ribosomal protein L23
903	IMAGp998L191853	0.84	0.833	*N/A*	Data not found
904	IMAGp998F246087	0.84	0.840	*N/A*	Transcribed locus
905	IMAGp998E075583	0.84	0.831	*LOC643837*	Hypothetical protein LOC643837
906	IMAGp998G06113	0.84	0.854	*N/A*	Data not found
907	IMAGp998E17202	0.84	0.839	*N/A*	Transcribed locus
908	IMAGp998F03372	0.84	0.845	*UBE2D3*	Ubiquitin-conjugating enzyme E2D 3 (UBC4/5 homolog, yeast)
909	IMAGp998F17286	0.84	0.855	*TUFM*	Tu translation elongation factor, mitochondrial
910	IMAGp998K12128	0.84	0.821	*SLC29A3*	Solute carrier family 29 (nucleoside transporters), member 3
911	IMAGp998N10470	0.84	0.810	*ATF7IP2*	Activating transcription factor 7 interacting protein 2
912	IMAGp998P24615	0.84	0.882	*KIAA0232*	KIAA0232 gene product
913	RZPDp202C019D	0.84	0.832	*RP11-45J16.2*	Flavin-containing monooxygenase pseudogene
914	IMAGp998J15358	0.84	0.799	*N/A*	Transcribed locus
915	IMAGp998K135507	0.84	0.828	*ANXA10*	Annexin A10
916	IMAGp998O054455	0.84	0.864	*LRRC28*	Leucine rich repeat containing 28
917	IMAGp998D23525	0.84	0.834	*TRIM10*	Tripartite motif-containing 10
918	IMAGp998O115617	0.84	0.831	*N/A*	Transcribed locus
919	IMAGp998G06652	0.84	0.837	*TF,*	Transferrin,Transcribed locus
920	IMAGp998M092039	0.84	0.808	*N/A*	Data not found
921	IMAGp998D19286	0.84	0.836	*EFNB3*	Ephrin-B3
922	IMAGp998B101927	0.84	0.924	*N/A*	Transcribed locus
923	IMAGp998L125587	0.84	0.825	*N/A*	Transcribed locus
924	IMAGp998G213851	0.84	0.792	*N/A*	Data not found
925	IMAGp998H071906	0.84	0.827	*KIAA0828*	Adenosylhomocysteinase 3
926	IMAGp998J01156	0.84	0.859	*NR2E1*	Nuclear receptor subfamily 2, group E, member 1
927	IMAGp998O04611	0.84	0.817	*N/A*	Transcribed locus
928	IMAGp998A0197	0.84	0.921	*SPECC1L*	SPECC1-like
929	IMAGp998J025670	0.84	0.859	*N/A*	Transcribed locus
930	IMAGp998F014642	0.84	0.853	*N/A*	CDNA FLJ12909 fis, clone NT2RP2004400
931	IMAGp998N03133	0.84	0.845	*ADD1*	Adducin 1 (alpha)
932	IMAGp998B101785	0.84	0.910	*ARRDC1*	Arrestin domain containing 1
933	IMAGp998K18403	0.84	0.830	*N/A*	Transcribed locus
934	IMAGp998B10725	0.84	0.928	*PPFIBP2*	PTPRF interacting protein, binding protein 2 (liprin beta 2)
935	IMAGp998B243850	0.84	0.828	*OBSCN*	Obscurin, cytoskeletal calmodulin and titin-interacting RhoGEF
936	IMAGp998O08442	0.84	0.827	*N/A*	Transcribed locus, moderately similar to XP_529501.1 hypothetical protein XP_529501 [Pan troglodytes]
937	RZPDp1096F025D	0.84	0.829	*HIST1H3E*	Histone cluster 1, H3e
938	IMAGp998A02370	0.84	0.803	*MT1G*	Metallothionein 1G
939	IMAGp998F022406	0.84	0.840	*N/A*	Transcribed locus
940	RZPDp1096F062D	0.84	0.853	*SULT1A1*	Sulfotransferase family, cytosolic, 1A, phenol-preferring, member 1
941	IMAGp998I23604	0.84	0.861	*N/A*	Data not found
942	IMAGp998L161863	0.84	0.911	*MSRA*	Methionine sulfoxide reductase A
943	IMAGp998E155510	0.84	0.820	*SMARCAL1*	SWI/SNF related, matrix associated, actin dependent regulator of chromatin, subfamily a-like 1
944	RZPDp201H0828D	0.84	0.747	*HLA-DRB1*	Major histocompatibility complex, class II, DR beta 1
945	IMAGp998P05830	0.84	0.842	*COL9A3*	Collagen, type IX, alpha 3

946	RZPDp201D0515D	0.84	0.841	*EPN3*	Epsin 3
947	IMAGp998F16559	0.84	0.907	*CREM*	CAMP responsive element modulator
948	IMAGp998G15289	0.84	0.842	*N/A*	Data not found
949	IMAGp998J16331	0.84	0.865	*MPDU1*	Mannose-P-dolichol utilization defect 1
950	RZPDp202A087D	0.84	0.833	*MIER2*	Mesoderm induction early response 1, family member 2
951	IMAGp998I02228	0.84	0.801	*N/A*	Transcribed locus
952	IMAGp998M071775	0.84	0.835	*N/A*	Transcribed locus
953	RZPDp201A1036D	0.84	0.851	*ZSCAN22*	Zinc finger and SCAN domain containing 22
954	IMAGp998C22385	0.84	0.842	*N/A*	Data not found
955	IMAGp998I075985	0.84	0.855	*N/A*	Transcribed locus
956	IMAGp998E19609	0.84	0.848	*RBBP6*	Retinoblastoma binding protein 6
957	IMAGp998D21154	0.84	0.858	*CTBP2*	C-terminal binding protein 2
958	IMAGp998G01441	0.84	0.803	*N/A*	Data not found
959	IMAGp998C095452	0.84	0.903	*N/A*	CDNA FLJ12030 fis, clone HEMBB1001868
960	IMAGp998H02230	0.84	0.796	*GATA2*	GATA binding protein 2
961	IMAGp998K06661	0.84	0.877	*SEC14L1*	SEC14-like 1 (S. cerevisiae)
962	RZPDp202F108D	0.84	0.814	*DNASE2*	Deoxyribonuclease II, lysosomal
963	IMAGp998K13360	0.84	0.805	*N/A*	Data not found
964	IMAGp998I18652	0.84	0.820	*CSPP1*	Centrosome and spindle pole associated protein 1
965	RZPDp201E052D	0.84	0.877	*N/A*	Transcribed locus
966	IMAGp998N02166	0.84	0.802	*NR4A2*	Nuclear receptor subfamily 4, group A, member 2
967	RZPDp201E0926D	0.84	0.904	*ZFYVE20*	Zinc finger, FYVE domain containing 20
968	IMAGp998P18194	0.84	0.817	*N/A*	Data not found
969	IMAGp998J21131	0.84	0.839	*N/A*	Data not found
970	IMAGp998N031795	0.84	0.814	*N/A*	CDNA FLJ26188 fis, clone ADG04821
971	IMAGp998K231822	0.84	0.883	*FLYWCH1*	FLYWCH-type zinc finger 1
972	IMAGp998F01668	0.84	0.801	*SMYD1*	SET and MYND domain containing 1
973	IMAGp998F185288	0.84	0.844	*LRGUK*	Leucine-rich repeats and guanylate kinase domain containing
974	IMAGp998D13659	0.84	0.828	*N/A*	Data not found
975	IMAGp998G24161	0.84	0.831	*N/A*	Transcribed locus
976	IMAGp998B115469	0.84	0.826	*POLD1*	Polymerase (DNA directed), delta 1, catalytic subunit 125kDa
977	IMAGp998G095790	0.84	0.906	*GUCA2B*	Guanylate cyclase activator 2B (uroguanylin)
978	IMAGp998C242036	0.84	0.798	*PPP1R12A*	Protein phosphatase 1, regulatory (inhibitor) subunit 12A
979	IMAGp998G01398	0.84	0.809	*C11orf58*	Chromosome 11 open reading frame 58
980	IMAGp998F205470	0.84	0.816	*KLHL8*	Kelch-like 8 (Drosophila)
981	IMAGp998N20787	0.84	0.795	*MCCC1*	Methylcrotonoyl-Coenzyme A carboxylase 1 (alpha)
982	IMAGp998B07930	0.84	0.830	*ACVRL1*	Activin A receptor type II-like 1
983	IMAGp998I094494	0.84	0.904	*N/A*	Transcribed locus
984	IMAGp998G01235	0.84	0.821	*N/A*	Data not found
985	RZPDp202F042D	0.84	0.830	*GP6*	Glycoprotein VI (platelet)
986	IMAGp998I12313	0.84	0.846	*N/A*	Transcribed locus, strongly similar to XP_531240.1 hypothetical protein XP_531240 [Pan troglodytes]
987	IMAGp998N073956	0.84	0.826	*N/A*	Transcribed locus
988	IMAGp998A18363	0.84	0.831	*PKHD1L1*	Polycystic kidney and hepatic disease 1 (autosomal recessive)-like 1
989	IMAGp998N102035	0.84	0.837	*N/A*	Data not found
990	IMAGp998N09367	0.84	0.823	*BRCA2*	Breast cancer 2, early onset
991	IMAGp998M184862	0.84	0.866	*ZNF607*	Zinc finger protein 607
992	IMAGp998E126096	0.84	0.837	*C12orf46*	Chromosome 12 open reading frame 46
993	RZPDp202B058D	0.84	0.861	*DDB1*	Damage-specific DNA binding protein 1, 127kDa
994	IMAGp998N226070	0.84	0.877	*ALDH1A2*	Aldehyde dehydrogenase 1 family, member A2
995	IMAGp998M09413	0.84	0.720	*N/A*	Data not found
996	RZPDp202A092D	0.84	0.869	*SLC35D2*	Solute carrier family 35, member D2
997	IMAGp998K072985	0.84	0.819	*N/A*	Transcribed locus
998	IMAGp998G22115	0.84	0.838	*N/A*	Data not found
999	IMAGp998J11124	0.84	0.909	*ZNF76*	Zinc finger protein 76 (expressed in testis)
1000	IMAGp998L24128	0.84	0.795	*ACOX1*	Acyl-Coenzyme A oxidase 1, palmitoyl
1001	IMAGp998C06840	0.84	0.829	*XRCC6BP1*	XRCC6 binding protein 1
1002	IMAGp998I215998	0.84	0.843	*N/A*	Transcribed locus
1003	IMAGp998G176096	0.84	0.881	*PHYHD1*	Phytanoyl-CoA dioxygenase domain containing 1
1004	IMAGp998K205287	0.84	0.844	*FLJ39660*	Hypothetical protein FLJ39660
1005	IMAGp998E18689	0.84	0.850	*N/A*	Transcribed locus
1006	IMAGp998L125664	0.84	0.850	*BCLAF1*	BCL2-associated transcription factor 1
1007	IMAGp998J233113	0.84	0.882	*N/A*	Transcribed locus
1008	IMAGp998N1172	0.84	0.912	*SUPT6H*	Suppressor of Ty 6 homolog (S. cerevisiae)
1009	IMAGp998H163902	0.84	0.845	*N/A*	Transcribed locus
1010	IMAGp998H125554	0.84	0.916	*MAN2C1*	Mannosidase, alpha, class 2C, member 1
1011	IMAGp998E104069	0.84	0.842	*N/A*	Data not found
1012	IMAGp998I085283	0.84	0.826	*N/A*	Transcribed locus
1013	IMAGp998F065797	0.84	0.849	*ZNF616*	Zinc finger protein 616
1014	RZPDp201F1119D	1.20	0.805	*CPT1A*	Carnitine palmitoyltransferase 1A (liver)
1015	IMAGp998A1188	1.20	0.849	*N/A*	Transcribed locus
1016	IMAGp998G112005	1.20	0.835	*N/A*	Transcribed locus
1017	IMAGp998K18247	1.20	0.839	*CRY2*	Cryptochrome 2 (photolyase-like)
1018	IMAGp998E10541	1.20	0.841	*N/A*	In multiple clusters
1019	IMAGp998K23119	1.20	0.816	*WIPF2*	WAS/WASL interacting protein family, member 2
1020	RZPDp202B046D	1.20	0.883	*PARD3*	Par-3 partitioning defective 3 homolog (C. elegans)
1021	IMAGp998P22654	1.20	0.810	*hCG_1813624*	HCG1813624
1022	IMAGp998G21530	1.20	0.796	*SPRY3*	Sprouty homolog 3 (Drosophila)
1023	IMAGp998J08238	1.20	0.851	*N/A*	Transcribed locus
1024	RZPDp201B0732D	1.20	0.884	*GMPR*	Guanosine monophosphate reductase
1025	IMAGp998K024893	1.20	0.870	*DHRS2*	Dehydrogenase/reductase (SDR family) member 2
1026	IMAGp998F21130	1.20	0.886	*DUSP16*	Dual specificity phosphatase 16
1027	IMAGp998F245671	1.20	0.841	*N/A*	Transcribed locus
1028	RZPDp1096A1215D	1.20	0.788	*LRP5L*	Low density lipoprotein receptor-related protein 5-like
1029	IMAGp998F08463	1.20	0.910	*GATA5*	GATA binding protein 5
1030	IMAGp998I235628	1.20	0.797	*N/A*	Transcribed locus
1031	IMAGp998H10972	1.20	0.822	*C14orf156*	Chromosome 14 open reading frame 156
1032	RZPDp201B036D	1.20	0.867	*TRPM6*	Transient receptor potential cation channel, subfamily M, member 6
1033	IMAGp998F164116	1.20	0.809	*N/A*	Transcribed locus
1034	IMAGp998I21413	1.20	0.824	*LOC400960*	Hypothetical gene supported by BC040598
1035	RZPDp1096A1217D	1.20	0.884	*RBM6*	RNA binding motif protein 6
1036	IMAGp998N061904	1.20	0.906	*N/A*	CDNA clone IMAGE:4800096
1037	IMAGp998M14248	1.20	0.823	*PSAP*	Prosaposin (variant Gaucher disease and variant metachromatic leukodystrophy)
1038	IMAGp998M14473	1.20	0.868	*STAP2*	Signal-transducing adaptor protein-2
1039	IMAGp998M225394	1.20	0.877	*MUC6*	Mucin 6, oligomeric mucus/gel-forming
1040	IMAGp998J131888	1.20	0.805	*N/A*	Data not found
1041	IMAGp998J061936	1.20	0.889	*N/A*	In multiple clusters
1042	IMAGp998D035499	1.20	0.842	*TMEM67*	Transmembrane protein 67
1043	IMAGp998P08662	1.20	0.864	*N/A*	MRNA; cDNA DKFZp667D2123 (from clone DKFZp667D2123)
1044	IMAGp998C05469	1.20	0.915	*N/A*	Homo sapiens, clone IMAGE:5586445, mRNA
1045	IMAGp998D236116	1.20	0.825	*N/A*	Data not found
1046	RZPDp1096C0316D	1.20	0.905	*ITGA3*	Integrin, alpha 3 (antigen CD49C, alpha 3 subunit of VLA-3 receptor)
1047	IMAGp998B242823	1.20	0.817	*LOC91431*	Prematurely terminated mRNA decay factor-like
1048	IMAGp998E03280	1.20	0.885	*PRPF6*	PRP6 pre-mRNA processing factor 6 homolog (S. cerevisiae)
1049	RZPDp1096F0815D	1.20	0.805	*N/A*	Data not found
1050	IMAGp998C123937	1.20	0.823	*N/A*	Transcribed locus
1051	IMAGp998I21155	1.20	0.894	*N/A*	Transcribed locus, weakly similar to XP_342577.3
1052	IMAGp998M235470	1.20	0.832	*RANBP2*	RAN binding protein 2
1053	IMAGp998E136080	1.20	0.845	*ADAMTSL5*	ADAMTS-like 5
1054	IMAGp998O103712	1.20	0.854	*N/A*	Transcribed locus
1055	IMAGp998I174137	1.20	0.847	*N/A*	Transcribed locus
1056	IMAGp998C235328	1.20	0.883	*N/A*	Transcribed locus
1057	IMAGp998D16285	1.20	0.845	*FBXO7*	F-box protein 7
1058	RZPDp202F064D	1.20	0.856	*PRUNE2*	Prune homolog 2 (Drosophila)
1059	RZPDp201C045D	1.20	0.851	*N/A*	Data not found
1060	IMAGp998I245106	1.20	0.829	*ANKFY1*	Ankyrin repeat and FYVE domain containing 1
1061	IMAGp998I085767	1.20	0.919	*N/A*	Transcribed locus
1062	IMAGp998N215403	1.20	0.855	*LOC642366*	Hypothetical LOC642366
1063	IMAGp998N185734	1.20	0.818	*N/A*	CDNA FLJ43044 fis, clone BRTHA3003474
1064	IMAGp998A095498	1.20	0.852	*C20orf74*	Chromosome 20 open reading frame 74
1065	IMAGp998L14113	1.20	0.816	*LOC285972*	Hypothetical protein LOC285972
1066	IMAGp998O13651	1.20	0.826	*GLUD1*	Glutamate dehydrogenase 1
1067	IMAGp998I12118	1.20	0.846	*ABHD5*	Abhydrolase domain containing 5
1068	IMAGp998O043797	1.20	0.837	*TTF2*	Transcription termination factor, RNA polymerase II
1069	IMAGp998L111749	1.20	0.915	*PRKD2*	Protein kinase D2
1070	IMAGp998B11397	1.20	0.873	*N/A*	Transcribed locus
1071	IMAGp998B104896	1.20	0.837	*N/A*	Transcribed locus
1072	IMAGp998P20177	1.20	0.850	*HMOX2*	Heme oxygenase (decycling) 2
1073	IMAGp998K232973	1.20	0.850	*UBE2J1*	Ubiquitin-conjugating enzyme E2, J1 (UBC6 homolog, yeast)
1074	IMAGp998D143932	1.20	0.841	*N/A*	Transcribed locus
1075	IMAGp998A19237	1.20	0.882	*LOC400566*	Hypothetical gene supported by AK128660
1076	IMAGp998I085399	1.20	0.834	*N/A*	Transcribed locus
1077	IMAGp998I044198	1.20	0.819	*N/A*	Data not found
1078	IMAGp998M20140	1.20	0.925	*EEFSEC*	Eukaryotic elongation factor, selenocysteine-tRNA-specific
1079	IMAGp998G205316	1.20	0.877	*OPLAH*	5-oxoprolinase (ATP-hydrolysing)
1080	RZPDp1096B114D	1.20	0.873	*ASTN2*	Astrotactin 2

1081	IMAGp998E20241	1.20	0.893	*PLVAP*	Plasmalemma vesicle associated protein
1082	RZPDp201A1229D	1.20	0.804	*FXR2*	Fragile X mental retardation, autosomal homolog 2
1083	RZPDp202E123D	1.20	0.815	*RCP9*	Calcitonin gene-related peptide-receptor component protein
1084	IMAGp998A24381	1.20	0.872	*TGOLN2*	Trans-golgi network protein 2
1085	IMAGp998J185995	1.20	0.839	*MCPH1*	Microcephaly, primary autosomal recessive 1
1086	IMAGp998C10274	1.20	0.848	*NAG6*	Hypothetical protein DKFZp434G156
1087	IMAGp998A12676	1.20	0.895	*VAMP8*	Vesicle-associated membrane protein 8 (endobrevin)
1088	RZPDp202D032D	1.20	0.830	*N/A*	Transcribed locus, strongly similar to XP_001079181.1 similar to uroplakin 1A [Rattus norvegicus]
1089	IMAGp998B13411	1.20	0.885	*N/A*	Data not found
1090	RZPDp202H013D	1.20	0.812	*OPA3*	Optic atrophy 3 (autosomal recessive, with chorea and spastic paraplegia)
1091	IMAGp998I15594	1.20	0.888	*NT5C3L*	5'-nucleotidase, cytosolic III-like
1092	IMAGp998I24133	1.20	0.835	*N/A*	Transcribed locus
1093	IMAGp998M16197	1.20	0.835	*N/A*	Data not found
1094	IMAGp998J03791	1.20	0.914	*FEM1A*	Fem-1 homolog a (C. elegans)
1095	IMAGp998F196087	1.20	0.835	*N/A*	Transcribed locus
1096	IMAGp998I154001	1.20	0.837	*N/A*	Transcribed locus
1097	IMAGp998B10138	1.20	0.893	*PLD3*	Phospholipase D family, member 3
1098	RZPDp1096D1017D	1.20	0.859	*URP2*	UNC-112 related protein 2
1099	IMAGp998B134247	1.20	0.817	*KTN1*	Kinectin 1 (kinesin receptor)
1100	IMAGp998M244883	1.20	0.838	*SHB*	Src homology 2 domain containing adaptor protein B
1101	IMAGp998K06295	1.20	0.858	*FXYD6*	FXYD domain containing ion transport regulator 6
1102	IMAGp998E13688	1.20	0.890	*CLDN23*	Claudin 23
1103	IMAGp998O01152	1.20	0.899	*NARFL*	Nuclear prelamin A recognition factor-like
1104	IMAGp998N11154	1.20	0.836	*N/A*	Transcribed locus
1105	IMAGp998C10620	1.20	0.814	*N/A*	Transcribed locus
1106	IMAGp998I0878	1.20	0.848	*N/A*	Transcribed locus
1107	IMAGp998O14473	1.20	0.816	*C1orf168*	Chromosome 1 open reading frame 168
1108	RZPDp202C096D	1.20	0.885	*RBM19*	RNA binding motif protein 19
1109	IMAGp998M05196	1.20	0.812	*CSNK1G1*	Casein kinase 1, gamma 1
1110	IMAGp998J086075	1.20	0.885	*N/A*	CDNA clone IMAGE:4791597
1111	IMAGp998A07742	1.20	0.873	*N/A*	Full length insert cDNA clone ZC64A04
1112	IMAGp998K22624	1.20	0.880	*LOC644941*	Similar to fatty acid amide hydrolase
1113	RZPDp202H117D	1.20	0.905	*N/A*	Transcribed locus, moderately similar to XP_001073289.1 hypothetical protein [Rattus norvegicus]
1114	IMAGp998L07377	1.20	0.823	*N/A*	Data not found
1115	IMAGp998E214497	1.20	0.878	*N/A*	Transcribed locus, strongly similar to XP_530080.1 hypothetical protein XP_530080 [Pan troglodytes]
1116	IMAGp998J231152	1.20	0.893	*C7orf49*	Chromosome 7 open reading frame 49
1117	IMAGp998G20692	1.20	0.844	*ZNF238*	Zinc finger protein 238
1118	IMAGp998H235269	1.20	0.868	*TDH*	L-threonine dehydrogenase
1119	RZPDp202D027D	1.20	0.836	*KLHL20*	Kelch-like 20 (Drosophila)
1120	IMAGp998B2486	1.20	0.816	*N/A*	Data not found
1121	IMAGp998F145990	1.20	0.868	*N/A*	Transcribed locus
1122	IMAGp998O075729	1.20	0.846	*N/A*	Data not found
1123	IMAGp998E0291	1.20	0.847	*N/A*	Data not found
1124	IMAGp998P10840	1.20	0.781	*CCDC80*	Coiled-coil domain containing 80
1125	IMAGp998D134570	1.20	0.879	*LOC728723*	Hypothetical protein LOC728723
1126	RZPDp202H044D	1.20	0.902	*EFHD2*	EF-hand domain family, member D2
1127	IMAGp998B22789	1.20	0.837	*N/A*	Transcribed locus
1128	IMAGp998L24422	1.20	0.807	*LOC400084*	Hypothetical gene supported by AK057632; AL137270; BC057846
1129	IMAGp998P11285	1.20	0.899	*MLLT4*	Myeloid/lymphoid or mixed-lineage leukemia (trithorax homolog, Drosophila); translocated to, 4
1130	IMAGp998J09563	1.20	0.843	*N/A*	Transcribed locus
1131	IMAGp998N22533	1.20	0.860	*N/A*	Transcribed locus
1132	IMAGp998M244455	1.20	0.852	*N/A*	Transcribed locus
1133	RZPDp201B1018D	1.20	0.896	*GRIPAP1*	GRIP1 associated protein 1
1134	IMAGp998D245648	1.20	0.853	*N/A*	Transcribed locus
1135	IMAGp998C035052	1.20	0.880	*N/A*	Data not found
1136	IMAGp998O09238	1.20	0.844	*KATNAL1*	Katanin p60 subunit A-like 1
1137	IMAGp998E075323	1.20	0.846	*KIF7*	Kinesin family member 7
1138	IMAGp998L01736	1.20	0.802	*HLA-A*	Major histocompatibility complex, class I, A
1139	IMAGp998J245491	1.20	0.845	*ZFPM1*	Zinc finger protein, multitype 1
1140	IMAGp998D064018	1.20	0.817	*N/A*	Data not found
1141	IMAGp998H152037	1.20	0.887	*FAM96B*	Family with sequence similarity 96, member B
1142	IMAGp998J075462	1.20	0.827	*KLHL6*	Kelch-like 6 (Drosophila)
1143	IMAGp998H191822	1.20	0.905	*SLC25A23*	Solute carrier family 25 (mitochondrial carrier; phosphate carrier), member 23
1144	IMAGp998G07167	1.20	0.846	*N/A*	Transcribed locus, moderately similar to XP_531054.1 hypothetical protein XP_531054 [Pan troglodytes]
1145	IMAGp998P05975	1.20	0.855	*N/A*	Data not found
1146	IMAGp998E11240	1.20	0.872	*LSDP5*	Lipid storage droplet protein 5
1147	IMAGp998G16387	1.20	0.818	*MSL-1*	Male-specific lethal-1 homolog
1148	RZPDp202C043D	1.20	0.821	*SIGLEC10*	Sialic acid binding Ig-like lectin 10
1149	RZPDp201G061D	1.20	0.880	*ITIH5*	Inter-alpha (globulin) inhibitor H5
1150	IMAGp998H124353	1.20	0.862	*N/A*	Transcribed locus
1151	IMAGp998G08405	1.20	0.861	*N/A*	Transcribed locus
1152	IMAGp998N231934	1.20	0.900	*ZNF655*	Zinc finger protein 655
1153	IMAGp998G174653	1.20	0.851	*EDEM3*	ER degradation enhancer, mannosidase alpha-like 3
1154	IMAGp998B0884	1.20	0.836	*MPP7*	Membrane protein, palmitoylated 7 (MAGUK p55 subfamily member 7)
1155	IMAGp998H12841	1.20	0.824	*HOXC13*	Homeobox C13
1156	RZPDp201D1029D	1.20	0.881	*NSUN5*	NOL1/NOP2/Sun domain family, member 5
1157	RZPDp202A095D	1.20	0.842	*RALGDS*	Ral guanine nucleotide dissociation stimulator
1158	IMAGp998P082652	1.20	0.862	*N/A*	Transcribed locus
1159	IMAGp998G1317	1.20	0.837	*N/A*	Transcribed locus
1160	IMAGp998D16219	1.20	0.814	*CDKN2D*	Cyclin-dependent kinase inhibitor 2D (p19, inhibits CDK4)
1161	IMAGp998G02472	1.20	0.808	*N/A*	Transcribed locus
1162	IMAGp998B195288	1.20	0.828	*N/A*	Transcribed locus
1163	IMAGp998F15120	1.20	0.843	*ZNF346*	Zinc finger protein 346
1164	IMAGp998I134601	1.20	0.832	*OLFML2A*	Olfactomedin-like 2A
1165	IMAGp998O09736	1.20	0.903	*MMP24*	Matrix metallopeptidase 24 (membrane-inserted)
1166	IMAGp998O14113	1.20	0.813	*N/A*	Data not found
1167	IMAGp998D07657	1.20	0.830	*N/A*	Transcribed locus
1168	IMAGp998A17121	1.20	0.833	*C10orf58*	Chromosome 10 open reading frame 58
1169	IMAGp998J035383	1.20	0.865	*N/A*	Transcribed locus
1170	IMAGp998F035317	1.20	0.839	*N/A*	CDNA FLJ42256 fis, clone TKIDN2010232
1171	IMAGp998I225648	1.20	0.851	*MAP4K1*	Mitogen-activated protein kinase kinase kinase kinase 1
1172	IMAGp998F041864	1.20	0.837	*N/A*	Data not found
1173	IMAGp998P16370	1.20	0.827	*FNDC3B*	Fibronectin type III domain containing 3B
1174	IMAGp998F205286	1.20	0.837	*N/A*	Transcribed locus
1175	IMAGp998F104932	1.20	0.870	*CABIN1*	Calcineurin binding protein 1
1176	IMAGp998D225298	1.20	0.881	*N/A*	Data not found
1177	IMAGp998P171999	1.20	0.814	*CA13*	Carbonic anhydrase XIII
1178	IMAGp998G06593	1.20	0.831	*N/A*	Transcribed locus
1179	IMAGp998D134117	1.20	0.921	*N/A*	Transcribed locus
1180	IMAGp998H2369	1.20	0.839	*PIGO*	Phosphatidylinositol glycan anchor biosynthesis, class O
1181	RZPDp202A014D	1.20	0.859	*LRRC47*	Leucine rich repeat containing 47
1182	IMAGp998C14131	1.20	0.835	*CBX3*	Chromobox homolog 3 (HP1 gamma homolog, Drosophila)
1183	IMAGp998H12117	1.20	0.847	*RABL4*	RAB, member of RAS oncogene family-like 4
1184	IMAGp998F10417	1.20	0.823	*N/A*	Data not found
1185	IMAGp998F234968	1.20	0.911	*ZNF423*	Zinc finger protein 423
1186	IMAGp998P16166	1.20	0.889	*N/A*	Transcribed locus, strongly similar to XP_527520.2 neuromedin B receptor [Pan troglodytes]
1187	IMAGp998H16976	1.20	0.866	*CAPN5*	Calpain 5
1188	IMAGp998L13587	1.20	0.884	*ZNF395*	Zinc finger protein 395
1189	IMAGp998E22393	1.20	0.868	*N/A*	Data not found
1190	RZPDp201D0928D	1.20	0.739	*RGS1*	Regulator of G-protein signalling 1
1191	IMAGp998P09242	1.20	0.830	*LRRIQ2*	Leucine-rich repeats and IQ motif containing 2
1192	IMAGp998J024162	1.20	0.830	*LOC727982*	Hypothetical protein LOC727982
1193	IMAGp998E241165	1.20	0.891	*ASTN2*	Astrotactin 2
1194	RZPDp1096F028D	1.20	0.782	*VGCNL1*	Voltage gated channel like 1
1195	IMAGp998A0574	1.20	0.842	*N/A*	Clone 23548 mRNA sequence
1196	IMAGp998L131007	1.20	0.876	*STK36*	Serine/threonine kinase 36, fused homolog (Drosophila)
1197	IMAGp998N225600	1.20	0.888	*EFHA2*	EF-hand domain family, member A2
1198	IMAGp998F20823	1.20	0.782	*C11orf75*	Chromosome 11 open reading frame 75
1199	IMAGp998O04444	1.20	0.866	*TRAF7*	TNF receptor-associated factor 7
1200	IMAGp998B04172	1.20	0.894	*KIAA1411*	KIAA1411
1201	IMAGp998E1374	1.20	0.823	*C9orf91*	Chromosome 9 open reading frame 91
1202	IMAGp998H041791	1.20	0.871	*FAM114A1*	Family with sequence similarity 114, member A1
1203	IMAGp998J024183	1.20	0.842	*N/A*	Data not found
1204	IMAGp998I211775	1.20	0.832	*N/A*	Data not found
1205	RZPDp202E116D	1.20	0.844	*TRIM25*	Tripartite motif-containing 25
1206	RZPDp1096D0515D	1.20	0.842	*NEK8*	NIMA (never in mitosis gene a)- related kinase 8
1207	RZPDp1096C045D	1.20	0.887	*KIAA0828*	Adenosylhomocysteinase 3
1208	IMAGp998E102643	1.20	0.839	*N/A*	Data not found
1209	IMAGp998F112621	1.20	0.819	*N/A*	Transcribed locus
1210	IMAGp998F05469	1.20	0.865	*N/A*	In multiple clusters
1211	IMAGp998H12260	1.20	0.821	*N/A*	Data not found
1212	IMAGp998K191776	1.20	0.898	*CABIN1*	Calcineurin binding protein 1
1213	IMAGp998I084457	1.20	0.895	*N/A*	Transcribed locus
1214	IMAGp998K046084	1.20	0.864	*N/A*	Transcribed locus
1215	IMAGp998O23441	1.20	0.829	*EXOC1*	Exocyst complex component 1

1216	IMAGp998O18155	1.20	0.834	CPLX2	Complexin 2
1217	IMAGp998E205627	1.20	0.910	N/A	Transcribed locus
1218	IMAGp998E135507	1.20	0.852	N/A	MRNA; cDNA DKFZp686I18116 (from clone DKFZp686I18116)
1219	IMAGp998B18670	1.20	0.884	TMEM141	Transmembrane protein 141
1220	IMAGp998A21151	1.20	0.849	N/A	Data not found
1221	RZPDp202D019D	1.20	0.923	AKR1C2	Aldo-keto reductase family 1, member C2 (dihydrodiol dehydrogenase 2; bile acid binding protein; 3-alpha hydroxysteroid dehydrogenase, type III)
1222	IMAGp998P115926	1.20	0.840	N/A	Transcribed locus
1223	IMAGp998O22327	1.20	0.842	N/A	Data not found
1224	IMAGp998G13270	1.20	0.906	HRBL	HIV-1 Rev binding protein-like
1225	IMAGp998F134527	1.20	0.882	MGC15613	Hypothetical protein MGC15613
1226	IMAGp998B04421	1.20	0.844	N/A	Transcribed locus
1227	IMAGp998H12401	1.20	0.867	N/A	Data not found
1228	RZPDp1096D0519D	1.20	0.768	NUP50	Nucleoporin 50kDa
1229	RZPDp201E0228D	1.20	0.864	CCDC9	Coiled-coil domain containing 9
1230	RZPDp201G091D	1.20	0.836	GNG13	Guanine nucleotide binding protein (G protein), gamma 13
1231	IMAGp998L04415	1.20	0.823	PHLDA2	Pleckstrin homology-like domain, family A, member 2
1232	RZPDp1096C0414D	1.20	0.857	HSDL1	Hydroxysteroid dehydrogenase like 1
1233	RZPDp1096H026D	1.20	0.848	LOC201164	Similar to CG12314 gene product
1234	IMAGp998E23374	1.20	0.804	N/A	Data not found
1235	IMAGp998B17373	1.20	0.846	DNAJA4	DnaJ (Hsp40) homolog, subfamily A, member 4
1236	RZPDp202F053D	1.20	0.904	GBA	Glucosidase, beta; acid (includes glucosylceramidase)
1237	IMAGp998K12169	1.20	0.904	IGSF21	Immunoglobin superfamily, member 21
1238	IMAGp998A20264	1.20	0.870	MGC61598	Similar to ankyrin-repeat protein Nrarp
1239	IMAGp998C12142	1.20	0.860	MAGEH1	Melanoma antigen family H, 1
1240	RZPDp202D088D	1.20	0.825	RUNDC2B	RUN domain containing 2B
1241	IMAGp998B20159	1.20	0.864	N/A	Clone 24571 mRNA sequence
1242	IMAGp998L12833	1.20	0.863	N/A	Transcribed locus
1243	IMAGp998C06237	1.20	0.876	AEBP1	AE binding protein 1
1244	IMAGp998L025600	1.20	0.825	GYLTL1B	Glycosyltransferase-like 1B
1245	IMAGp998P23278	1.20	0.878	EN2	Engrailed homeobox 2
1246	IMAGp998H246110	1.20	0.842	C21orf34	Chromosome 21 open reading frame 34
1247	IMAGp998M096083	1.20	0.844	HSPC159	Galectin-related protein
1248	IMAGp998A20830	1.20	0.879	N/A	Pheromone receptor (PHRET) pseudogene, partial mRNA sequence
1249	IMAGp998A246097	1.20	0.849	SGIP1	SH3-domain GRB2-like (endophilin) interacting protein 1
1250	IMAGp998M1192	1.20	0.846	EHMT1	Euchromatic histone-lysine N-methyltransferase 1
1251	IMAGp998L15196	1.20	0.824	TCTE3	T-complex-associated-testis-expressed 3
1252	IMAGp998M244209	1.20	0.889	KLHDC6	Kelch domain containing 6
1253	IMAGp998L135472	1.20	0.842	N/A	Transcribed locus
1254	IMAGp998E065655	1.20	0.871	CXXC4	CXXC finger 4
1255	IMAGp998G145500	1.20	0.831	DKFZP564O0823	DKFZP564O0823 protein
1256	RZPDp1096E104D	1.20	0.798	C11orf75	Chromosome 11 open reading frame 75
1257	IMAGp998N0580	1.20	0.834	C6orf72	Chromosome 6 open reading frame 72
1258	IMAGp998L115512	1.20	0.826	PELO	Pelota homolog (Drosophila)
1259	IMAGp998G18588	1.20	0.839	SNRK	SNF related kinase
1260	IMAGp998F045323	1.20	0.861	N/A	Transcribed locus
1261	IMAGp998B07911	1.20	0.913	N/A	Transcribed locus
1262	IMAGp998M215457	1.20	0.884	SDSL	Serine dehydratase-like
1263	IMAGp998H171901	1.20	0.837	GADD45B	Growth arrest and DNA-damage-inducible, beta
1264	RZPDp202B078D	1.20	0.810	DNAJC8	DnaJ (Hsp40) homolog, subfamily C, member 8
1265	IMAGp998B056099	1.20	0.814	N/A	Transcribed locus
1266	IMAGp998C144996	1.20	0.936	N/A	Transcribed locus
1267	RZPDp201G0717D	1.20	0.853	FGF10	Fibroblast growth factor 10
1268	RZPDp1096G1114D	1.20	0.928	ERICH1	Glutamate-rich 1
1269	IMAGp998K08276	1.20	0.773	HLA-B	Major histocompatibility complex, class I, B
1270	IMAGp998B20176	1.20	0.746	TKT	Transketolase (Wernicke-Korsakoff syndrome)
1271	IMAGp998J13121	1.20	0.828	HDDC3	HD domain containing 3
1272	IMAGp998D061795	1.20	0.832	N/A	Transcribed locus
1273	IMAGp998J101010	1.20	0.833	RHOBTB3	Rho-related BTB domain containing 3
1274	IMAGp998H24113	1.20	0.847	N/A	Transcribed locus
1275	IMAGp998D12737	1.20	0.927	SLC27A3	Solute carrier family 27 (fatty acid transporter), member 3
1276	RZPDp202G041D	1.20	0.858	N/A	Data not found
1277	IMAGp998I154521	1.20	0.906	SH3PX3	SH3 and PX domain containing 3
1278	IMAGp998G24369	1.20	0.858	TRIM34	Tripartite motif-containing 34
1279	IMAGp998O232676	1.20	0.878	KCNT2	Potassium channel, subfamily T, member 2
1280	RZPDp1096B0314D	1.20	0.841	RASL10B	RAS-like, family 10, member B
1281	RZPDp202C042D	1.20	0.895	N/A	Transcribed locus, weakly similar to XP_576276.2 hypothetical protein [Rattus norvegicus]
1282	IMAGp998J17781	1.20	0.889	IL23A	Interleukin 23, alpha subunit p19
1283	IMAGp998B072675	1.20	0.880	ILKAP	Integrin-linked kinase-associated serine/threonine phosphatase 2C
1284	IMAGp998J125468	1.20	0.837	OSBPL7	Oxysterol binding protein-like 7
1285	IMAGp998D195319	1.20	0.837	CTTNBP2NL	CTTNBP2 N-terminal like
1286	IMAGp998B0573	1.20	0.847	CCNYL1	Cyclin Y-like 1
1287	IMAGp998K125285	1.20	0.867	LOC152485	Hypothetical protein LOC152485
1288	IMAGp998C17370	1.20	0.874	N-PAC	Cytokine-like nuclear factor n-pac
1289	IMAGp998L19593	1.20	0.851	PAFAH1B2	Platelet-activating factor acetylhydrolase, isoform Ib, beta subunit 30kDa
1290	RZPDp201C0434D	1.20	0.898	BUD13	BUD13 homolog (S. cerevisiae)
1291	RZPDp202D043D	1.20	0.828	LOC646652	Integral membrane glycoprotein-like
1292	IMAGp998C06891	1.20	0.818	KIAA1345	KIAA1345 protein
1293	IMAGp998H0283	1.20	0.848	N/A	Transcribed locus
1294	RZPDp201H0526D	1.20	0.850	C12orf38	Chromosome 12 open reading frame 38
1295	IMAGp998M12921	1.20	0.869	N/A	Transcribed locus
1296	IMAGp998I22142	1.20	0.841	GABRB2	Gamma-aminobutyric acid (GABA) A receptor, beta 2
1297	IMAGp998O071016	1.20	0.845	N/A	Transcribed locus
1298	IMAGp998I14464	1.20	0.830	N/A	Transcribed locus
1299	IMAGp998I105989	1.20	0.872	ASXL1	Additional sex combs like 1 (Drosophila)
1300	IMAGp998E245464	1.20	0.879	C12orf41	Chromosome 12 open reading frame 41
1301	IMAGp998O211115	1.20	0.894	CRKL	V-crk sarcoma virus CT10 oncogene homolog (avian)-like
1302	IMAGp998C164248	1.20	0.901	N/A	CDNA FLJ25399 fis, clone TST02783
1303	IMAGp998F152002	1.20	0.897	VPS16	Vacuolar protein sorting 16 homolog (S. cerevisiae)
1304	IMAGp998A093970	1.20	0.825	N/A	Transcribed locus
1305	IMAGp998I075469	1.20	0.865	WDR16	WD repeat domain 16
1306	IMAGp998F01280	1.20	0.854	PDE4DIP	Phosphodiesterase 4D interacting protein (myomegalin)
1307	IMAGp998I026113	1.20	0.823	ZNF530	Zinc finger protein 530
1308	IMAGp998J243811	1.20	0.854	DHX29	DEAH (Asp-Glu-Ala-His) box polypeptide 29
1309	RZPDp201A0210D	1.20	0.884	N/A	Immunoglobulin lambda chain mRNA, VJ region, hybridoma LSF2
1310	IMAGp998N115084	1.20	0.892	N/A	Transcribed locus
1311	IMAGp998B155344	1.20	0.850	N/A	Transcribed locus
1312	IMAGp998M022008	1.20	0.847	HOXB13	Homeobox B13
1313	RZPDp201E118D	1.20	0.917	TTLL11	Tubulin tyrosine ligase-like family, member 11
1314	IMAGp998I012374	1.20	0.847	N/A	Transcribed locus
1315	RZPDp202D121D	1.20	0.838	C8orf51	Chromosome 8 open reading frame 51
1316	IMAGp998K14213	1.20	0.926	DKFZp761E198	DKFZp761E198 protein
1317	IMAGp998B215601	1.20	0.858	FUSIP1	FUS interacting protein (serine/arginine-rich) 1
1318	RZPDp202G052D	1.20	0.843	CHRNB4	Cholinergic receptor, nicotinic, beta 4
1319	IMAGp998H095405	1.20	0.865	PUS7	Pseudouridylate synthase 7 homolog (S. cerevisiae)
1320	IMAGp998A14738	1.20	0.889	TCF25	Transcription factor 25 (basic helix-loop-helix)
1321	IMAGp998B17236	1.20	0.840	CEP135	Centrosomal protein 135kDa
1322	IMAGp998F20158	1.20	0.906	N/A	CDNA FLJ42249 fis, clone TKIDN2007667
1323	RZPDp1096G066D	1.20	0.589	ZFPM1	Zinc finger protein, multitype 1
1324	RZPDp201B0931D	1.20	0.856	C5orf16	Chromosome 5 open reading frame 16
1325	IMAGp998H13616	1.20	0.860	DDX55	DEAD (Asp-Glu-Ala-Asp) box polypeptide 55
1326	IMAGp998N235627	1.20	0.859	N/A	Data not found
1327	RZPDp201A0316D	1.20	0.844	BBC3	BCL2 binding component 3
1328	IMAGp998K01391	1.20	0.850	N/A	Data not found
1329	IMAGp998H17281	1.20	0.912	GPR107	G protein-coupled receptor 107
1330	IMAGp998G114653	1.20	0.808	N/A	Transcribed locus
1331	RZPDp202B044D	1.20	0.843	TRIM46	Tripartite motif-containing 46
1332	IMAGp998B22161	1.20	0.856	EIF2C4	Eukaryotic translation initiation factor 2C, 4
1333	IMAGp998E071725	1.20	0.878	PTGS1	Prostaglandin-endoperoxide synthase 1 (prostaglandin G/H synthase and cyclooxygenase)
1334	IMAGp998B143460	1.20	0.860	N/A	Data not found
1335	IMAGp998N063577	1.20	0.843	N/A	Transcribed locus
1336	IMAGp998P18282	1.20	0.842	N/A	Data not found
1337	IMAGp998D235152	1.20	0.849	N/A	Transcribed locus
1338	IMAGp998F08885	1.20	0.869	CDKN1B	Cyclin-dependent kinase inhibitor 1B (p27, Kip1)
1339	IMAGp998E06386	1.20	0.908	UBE2V1	Ubiquitin-conjugating enzyme E2 variant 1
1340	IMAGp998D04269	1.20	0.858	BCL6	B-cell CLL/lymphoma 6 (zinc finger protein 51)
1341	RZPDp201C121D	1.20	0.855	RASGEF1A	RasGEF domain family, member 1A
1342	RZPDp202E105D	1.20	0.908	PRKAB1	Protein kinase, AMP-activated, beta 1 non-catalytic subunit
1343	IMAGp998I12278	1.20	0.868	MRPL28	Mitochondrial ribosomal protein L28
1344	IMAGp998N074209	1.20	0.869	N/A	Transcribed locus
1345	RZPDp202C057D	1.20	0.909	DDB1	Damage-specific DNA binding protein 1, 127kDa
1346	IMAGp998I201944	1.20	0.933	GAS8	Growth arrest-specific 8
1347	RZPDp202E121D	1.20	0.933	LMAN2L	Lectin, mannose-binding 2-like
1348	IMAGp998G01532	1.20	0.850	N/A	Data not found
1349	IMAGp998E084943	1.20	0.903	N/A	Transcribed locus
1350	IMAGp998B156111	1.89	0.894	N/A	Transcribed locus

1351	IMAGp998H236073	1.89	0.842	*N/A*	CDNA FLJ13625 fis, clone PLACE1011032
1352	IMAGp998B181743	1.89	0.834	*SLC11A1*	Solute carrier family 11 (proton-coupled divalent metal ion transporters), member 1
1353	IMAGp998K12270	1.89	0.839	*ARSF*	Arylsulfatase F
1354	IMAGp998D07267	1.89	0.875	*N/A*	CDNA: FLJ22073 fis, clone HEP11868
1355	RZPDp1096D124D	1.89	0.915	*GSTT2*	Glutathione S-transferase theta 2
1356	IMAGp998C171009	1.89	0.849	*MGC24039*	Hypothetical protein MGC24039
1357	IMAGp998E082758	1.89	0.844	*N/A*	MRNA; cDNA DKFZp686E22185 (from clone DKFZp686E22185)
1358	IMAGp998A18269	1.89	0.862	*TOM1*	Target of myb1 (chicken)
1359	IMAGp998K134160	1.89	0.840	*N/A*	Transcribed locus
1360	IMAGp998D122578	1.89	0.847	*BMP8B*	Bone morphogenetic protein 8b (osteogenic protein 2)
1361	IMAGp998A19231	1.89	0.855	*KPNA3*	Karyopherin alpha 3 (importin alpha 4)
1362	IMAGp998H065377	1.89	0.834	*PICK1*	Protein interacting with PRKCA 1
1363	IMAGp998N02684	1.89	0.845	*MGC14425*	Hypothetical protein MGC14425
1364	IMAGp998C095417	1.89	0.855	*N/A*	Transcribed locus
1365	IMAGp998D021964	1.89	0.858	*C11orf55*	Chromosome 11 open reading frame 55
1366	IMAGp998L204900	1.89	0.857	*N/A*	Data not found
1367	IMAGp998D154963	1.89	0.910	*RUNDC2A*	RUN domain containing 2A
1368	IMAGp998K245284	1.89	0.869	*SYNE2*	Spectrin repeat containing, nuclear envelope 2
1369	RZPDp202F117D	1.89	0.863	*PIH1D1*	PIH1 domain containing 1
1370	IMAGp998H16659	1.89	0.857	*N/A*	Transcribed locus
1371	IMAGp998B244351	1.89	0.845	*N/A*	Data not found
1372	IMAGp998M0769	1.89	0.906	*N/A*	Transcribed locus
1373	IMAGp998A211006	1.89	0.919	*N/A*	In multiple clusters
1374	RZPDp201H0313D	1.89	0.678	*FGF1*	Fibroblast growth factor 1 (acidic)
1375	IMAGp998P155327	1.89	0.872	*ST6GAL1*	ST6 beta-galactosamide alpha-2,6-sialyltranferase 1
1376	IMAGp998B221999	1.89	0.849	*CRTAP*	Cartilage associated protein
1377	IMAGp998E075635	1.89	0.859	*MGC33657*	Similar to hypothetical protein
1378	IMAGp998J224451	1.89	0.863	*HIST1H2AA*	Histone cluster 1, H2aa
1379	IMAGp998N072012	1.89	0.878	*SPIB*	Spi-B transcription factor (Spi-1/PU.1 related)
1380	IMAGp998G19404	1.89	0.860	*PKNOX1*	PBX/knotted 1 homeobox 1
1381	IMAGp998M02124	1.89	0.845	*ABCD4*	ATP-binding cassette, sub-family D (ALD), member 4
1382	IMAGp998G064329	1.89	0.843	*N/A*	Transcribed locus
1383	IMAGp998O08204	1.89	0.845	*DTWD1*	DTW domain containing 1
1384	IMAGp998D04657	1.89	0.855	*EIF4E2*	Eukaryotic translation initiation factor 4E family member 2
1385	IMAGp998G163712	1.89	0.878	*N/A*	Transcribed locus, strongly similar to XP_530773.1 hypothetical protein XP_530773 [Pan troglodytes]
1386	IMAGp998E16276	1.89	0.835	*JAKMIP2*	Janus kinase and microtubule interacting protein 2
1387	IMAGp998P01124	1.89	0.811	*HFE*	Hemochromatosis
1388	RZPDp201B081D	1.89	0.878	*SQRDL*	Sulfide quinone reductase-like (yeast)
1389	RZPDp1096G072D	1.89	0.804	*CDH1*	Cadherin 1, type 1, E-cadherin (epithelial)
1390	IMAGp998N135623	1.89	0.847	*N/A*	Data not found
1391	IMAGp998K24160	1.89	0.919	*RBM23*	RNA binding motif protein 23
1392	RZPDp202D046D	1.89	0.832	*KCNH4*	Potassium voltage-gated channel, subfamily H (eag-related), member 4
1393	IMAGp998P095851	1.89	0.918	*N/A*	Transcribed locus, strongly similar to XP_001161908.1 hypothetical protein [Pan troglodytes]
1394	RZPDp1096A1017D	1.89	0.886	*RNF44*	Ring finger protein 44
1395	RZPDp202E062D	1.89	0.888	*FUT10*	Fucosyltransferase 10 (alpha (1,3) fucosyltransferase)
1396	IMAGp998B16620	1.89	0.854	*N/A*	Transcribed locus
1397	IMAGp998F1792	1.89	0.905	*N/A*	Data not found
1398	IMAGp998K022011	1.89	0.836	*N/A*	Full-length cDNA clone CS0DL005YA15 of B cells (Ramos cell line) Cot 25-normalized of Homo sapiens (human)
1399	IMAGp998K08360	1.89	0.827	*N/A*	Transcribed locus
1400	IMAGp998N075488	1.89	0.861	*N/A*	Transcribed locus
1401	IMAGp998G013057	1.89	0.873	*N/A*	Transcribed locus
1402	IMAGp998M11976	1.89	0.886	*KIAA0317*	KIAA0317
1403	IMAGp998J01134	1.89	0.868	*DLG5*	Discs, large homolog 5 (Drosophila)
1404	IMAGp998H105380	1.89	0.860	*N/A*	CDNA clone IMAGE:5263963
1405	IMAGp998P03348	1.89	0.905	*SUPT5H*	Suppressor of Ty 5 homolog (S. cerevisiae)
1406	RZPDp202C122D	1.89	0.847	*ERN2*	Endoplasmic reticulum to nucleus signalling 2
1407	IMAGp998A144493	1.89	0.877	*N/A*	Transcribed locus, strongly similar to XP_529727.1 hypothetical protein XP_529727 [Pan troglodytes]
1408	IMAGp998G1592	1.89	0.914	*N/A*	Data not found
1409	IMAGp998O111795	1.89	0.868	*N/A*	Transcribed locus
1410	IMAGp998I114963	1.89	0.923	*LOC286382*	Hypothetical protein LOC286382
1411	IMAGp998I03599	1.89	0.904	*N/A*	Transcribed locus
1412	IMAGp998N171964	1.89	0.870	*LGR5*	Leucine-rich repeat-containing G protein-coupled receptor 5
1413	RZPDp202A057D	1.89	0.853	*CDT1*	Chromatin licensing and DNA replication factor 1
1414	IMAGp998A18679	1.89	0.873	*FAM98A*	Family with sequence similarity 98, member A
1415	RZPDp1096E053D	1.89	0.912	*CDC2L5*	Cell division cycle 2-like 5 (cholinesterase-related cell division controller)
1416	IMAGp998P214526	1.89	0.872	*N/A*	Transcribed locus
1417	IMAGp998M203722	1.89	0.816	*N/A*	Transcribed locus
1418	IMAGp998N221833	1.89	0.835	*PTENP1*	Phosphatase and tensin homolog (mutated in multiple advanced cancers 1), pseudogene 1
1419	IMAGp998A105872	1.89	0.873	*SP3*	Sp3 transcription factor
1420	IMAGp998P04279	1.89	0.854	*N/A*	CDNA FLJ42268 fis, clone TKIDN2015161
1421	IMAGp998L125470	1.89	0.838	*NY-SAR-48*	Sarcoma antigen NY-SAR-48
1422	IMAGp998D154408	1.89	0.858	*CD19*	CD19 molecule
1423	IMAGp998C145322	1.89	0.853	*PPP4R1L*	Protein phosphatase 4, regulatory subunit 1-like
1424	IMAGp998L185569	1.89	0.927	*N/A*	CDNA FLJ31291 fis, clone KIDNE2007356
1425	IMAGp998N195407	1.89	0.847	*RHPN1*	Rhophilin, Rho GTPase binding protein 1
1426	IMAGp998N18280	1.89	0.884	*ARL4D*	ADP-ribosylation factor-like 4D
1427	IMAGp998D035589	1.89	0.849	*N/A*	Transcribed locus, strongly similar to XP_001132656.1 hypothetical protein [Homo sapiens]
1428	IMAGp998O04141	1.89	0.811	*N/A*	Data not found
1429	RZPDp202B053D	1.89	0.906	*C14orf131*	Chromosome 14 open reading frame 131
1430	IMAGp998H20278	1.89	0.851	*N/A*	In multiple clusters
1431	IMAGp998E241852	1.89	0.896	*PSMB10*	Proteasome (prosome, macropain) subunit, beta type, 10
1432	IMAGp998N104413	1.89	0.913	*TTBK2*	Tau tubulin kinase 2
1433	RZPDp202G067D	1.89	0.882	*BCL3*	B-cell CLL/lymphoma 3
1434	IMAGp998D01274	1.89	0.845	*TRIM2*	Tripartite motif-containing 2
1435	IMAGp998N085589	1.89	0.884	*N/A*	Transcribed locus
1436	RZPDp202C129D	1.89	0.900	*AHR*	Aryl hydrocarbon receptor
1437	IMAGp998I194887	1.89	0.870	*N/A*	Data not found
1438	IMAGp998K065776	1.89	0.913	*LOC401098*	Hypothetical LOC401098
1439	IMAGp998F05615	1.89	0.907	*RND2*	Rho family GTPase 2
1440	IMAGp998F244451	1.89	0.865	*DSE*	Dermatan sulfate epimerase
1441	IMAGp998M07406	1.89	0.814	*N/A*	CDNA FLJ42951 fis, clone BRSTN2007765
1442	IMAGp998M231787	1.89	0.941	*C17orf74*	Chromosome 17 open reading frame 74
1443	IMAGp998C18440	1.89	0.909	*N/A*	Transcribed locus
1444	IMAGp998H21469	1.89	0.864	*MT1M*	Metallothionein 1M
1445	IMAGp998L03524	1.89	0.865	*N/A*	Data not found
1446	IMAGp998C04608	1.89	0.854	*N/A*	Data not found
1447	IMAGp998N111854	1.89	0.884	*PTGES*	Prostaglandin E synthase
1448	IMAGp998H24268	1.89	0.870	*N/A*	CDNA FLJ45742 fis, clone KIDNE2016327
1449	IMAGp998H04930	1.89	0.898	*EIF4G2*	Eukaryotic translation initiation factor 4 gamma, 2
1450	RZPDp202D064D	1.89	0.829	*TMC6*	Transmembrane channel-like 6
1451	IMAGp998L183958	1.89	0.871	*N/A*	Transcribed locus
1452	IMAGp998G1681	1.89	0.819	*N/A*	Transcribed locus
1453	IMAGp998M221962	1.89	0.920	*N/A*	Transcribed locus, moderately similar to XP_934196.2 similar to hect domain and RLD 2 [Homo sapiens]
1454	IMAGp998K21912	1.89	0.892	*N/A*	Transcribed locus
1455	IMAGp998L21872	1.89	0.837	*ADORA2B*	Adenosine A2b receptor
1456	IMAGp998C13872	1.89	0.860	*FOXP1*	Forkhead box P1
1457	IMAGp998L19154	1.89	0.819	*ARFRP1*	ADP-ribosylation factor related protein 1
1458	RZPDp201A041D	1.89	0.893	*EMR3*	Egf-like module containing, mucin-like, hormone receptor-like 3
1459	IMAGp998E111784	1.89	0.839	*FLJ23834*	Hypothetical protein FLJ23834
1460	RZPDp202C012D	1.89	0.842	*RABL2A*	RAB, member of RAS oncogene family-like 2A
1461	RZPDp202G092D	1.89	0.829	*SLC2A6*	Solute carrier family 2 (facilitated glucose transporter), member 6
1462	IMAGp998I154380	1.89	0.853	*COBLL1*	COBL-like 1
1463	IMAGp998A14649	1.89	0.824	*LOC90110*	Hypothetical protein LOC90110
1464	IMAGp998H18127	1.89	0.865	*N/A*	Data not found
1465	IMAGp998A075396	1.89	0.870	*N/A*	Transcribed locus
1466	RZPDp202F024D	1.89	0.856	*AICDA*	Activation-induced cytidine deaminase
1467	IMAGp998O11274	1.89	0.862	*CAMTA1*	Calmodulin binding transcription activator 1
1468	IMAGp998I24524	1.89	0.841	*WAC*	WW domain containing adaptor with coiled-coil
1469	RZPDp201D0615D	1.89	0.854	*FUT3*	Fucosyltransferase 3 (galactoside 3(4)-L-fucosyltransferase, Lewis blood group)
1470	IMAGp998C15114	1.89	0.865	*N/A*	Data not found
1471	IMAGp998E2090	1.89	0.826	*SLC4A1*	Solute carrier family 4, anion exchanger, member 1 (erythrocyte membrane protein band 3, Diego blood group)
1472	IMAGp998M204148	1.89	0.921	*N/A*	CDNA clone IMAGE:4826240
1473	IMAGp998C224297	1.89	0.898	*N/A*	Data not found
1474	RZPDp1096B106D	1.89	0.901	*KIF6*	Kinesin family member 6
1475	IMAGp998J093953	1.89	0.846	*N/A*	In multiple clusters
1476	IMAGp998M014900	1.89	0.869	*C11orf16*	Chromosome 11 open reading frame 16
1477	IMAGp998B216115	1.89	0.832	*N/A*	Transcribed locus
1478	IMAGp998C225499	1.89	0.881	*TRIM22*	Tripartite motif-containing 22
1479	IMAGp998M09396	1.89	0.838	*N/A*	Data not found
1480	RZPDp201G081D	1.89	0.866	*C20orf30*	Chromosome 20 open reading frame 30
1481	IMAGp998I155487	1.89	0.863	*N/A*	Transcribed locus
1482	IMAGp998G104452	1.89	0.861	*N/A*	Transcribed locus
1483	IMAGp998C01271	1.89	0.856	*FLJ37798*	Hypothetical gene supported by AK095117
1484	IMAGp998E02377	1.89	0.878	*N/A*	Data not found
1485	IMAGp998M08280	1.89	0.902	*CA10*	Carbonic anhydrase X

1486	IMAGp998D21724	1.89	0.893	*EBF3*	Early B-cell factor 3
1487	IMAGp998F10416	1.89	0.859	*ZFYVE27*	Zinc finger, FYVE domain containing 27
1488	IMAGp998O14726	1.89	0.887	*N/A*	Transcribed locus
1489	IMAGp998B09224	1.89	0.836	*ALF*	TFIIA-alpha/beta-like factor
1490	IMAGp998O17578	1.89	0.838	*H3F3B*	H3 histone, family 3B (H3.3B)
1491	RZPDp201B1032D	1.89	0.901	*TPM3*	Tropomyosin 3
1492	IMAGp998B226106	1.89	0.845	*N/A*	CDNA FLJ11927 fis, clone HEMBB1000402
1493	IMAGp998A245381	1.89	0.851	*POLH*	Polymerase (DNA directed), eta
1494	IMAGp998I23629	1.89	0.848	*N/A*	Data not found
1495	IMAGp998C09153	1.89	0.862	*N/A*	Transcribed locus
1496	IMAGp998D206134	1.89	0.914	*N/A*	Transcribed locus
1497	IMAGp998I23979	1.89	0.901	*TMLHE*	Trimethyllysine hydroxylase, epsilon
1498	RZPDp202E097D	1.89	0.904	*TAP2*	Transporter 2, ATP-binding cassette, sub-family B (MDR/TAP)
1499	IMAGp998M154203	1.89	0.846	*N/A*	Data not found
1500	IMAGp998C075408	1.89	0.869	*FLJ43663*	Hypothetical protein FLJ43663
1501	IMAGp998N105213	1.89	0.851	*ZNF404*	Zinc finger protein 404
1502	RZPDp1096G0416D	1.89	0.896	*POL3S*	Polyserase 3
1503	IMAGp998M196090	1.89	0.863	*PSCD3*	Pleckstrin homology, Sec7 and coiled-coil domains 3
1504	IMAGp998C21217	1.89	0.933	*GALNT10*	UDP-N-acetyl-alpha-D-galactosamine:polypeptide N-acetylgalactosaminyltransferase 10 (GalNAc-T10)
1505	IMAGp998M21367	1.89	0.843	*N/A*	Data not found
1506	IMAGp998M10601	1.89	0.865	*ADAMTSL5*	ADAMTS-like 5
1507	IMAGp998L22978	1.89	0.858	*DLD*	Dihydrolipoamide dehydrogenase
1508	RZPDp202C106D	1.89	0.846	*C10orf65*	Chromosome 10 open reading frame 65
1509	RZPDp202E019D	1.89	0.848	*ST8SIA6*	ST8 alpha-N-acetyl-neuraminide alpha-2,8-sialyltransferase 6
1510	IMAGp998K03526	1.89	0.857	*APH1A*	Anterior pharynx defective 1 homolog A (C. elegans)
1511	IMAGp998O24274	1.89	0.912	*CDH5*	Cadherin 5, type 2, VE-cadherin (vascular epithelium)
1512	IMAGp998E14365	1.89	0.839	*RUTBC2*	RUN and TBC1 domain containing 2
1513	IMAGp998D02844	1.89	0.933	*APOL3*	Apolipoprotein L, 3
1514	IMAGp998G232638	1.89	0.865	*ANGPTL1*	Angiopoietin-like 1
1515	IMAGp998O215411	1.89	0.853	*N/A*	Transcribed locus
1516	IMAGp998H19171	1.89	0.872	*GTPBP9*	GTP-binding protein 9 (putative)
1517	RZPDp202A094D	1.89	0.897	*CD160*	CD160 molecule
1518	IMAGp998O031852	1.89	0.856	*GNAL*	Guanine nucleotide binding protein (G protein), alpha activating activity polypeptide, olfactory type
1519	RZPDp201F094D	1.89	0.888	*HAPLN1*	Hyaluronan and proteoglycan link protein 1
1520	IMAGp998L134251	1.89	0.857	*ONECUT1*	One cut domain, family member 1
1521	IMAGp998L0892	1.89	0.847	*N/A*	Transcribed locus
1522	IMAGp998F16525	1.89	0.840	*PKHD1L1*	Polycystic kidney and hepatic disease 1 (autosomal recessive)-like 1
1523	IMAGp998P05167	1.89	0.920	*N/A*	Transcribed locus, strongly similar to XP_001149790.1 cysteine-rich with EGF-like domains 1 isoform 1 [Pan troglodytes]
1524	IMAGp998M246079	1.89	0.885	*N/A*	Transcribed locus
1525	IMAGp998D065670	1.89	0.862	*WDR86*	WD repeat domain 86
1526	IMAGp998C21827	1.89	0.832	*MED9*	Mediator of RNA polymerase II transcription, subunit 9 homolog (S. cerevisiae)
1527	RZPDp202F128D	1.89	0.843	*RARS2*	Arginyl-tRNA synthetase 2, mitochondrial (putative)
1528	RZPDp202B026D	1.89	0.840	*SLC12A8*	Solute carrier family 12 (potassium/chloride transporters), member 8
1529	IMAGp998P146081	1.89	0.866	*GPR172A*	G protein-coupled receptor 172A
1530	IMAGp998K213677	1.89	0.836	*N/A*	Transcribed locus
1531	IMAGp998B021168	1.89	0.869	*FBXL17*	F-box and leucine-rich repeat protein 17
1532	IMAGp998C02382	1.89	0.839	*SLC35A3*	Solute carrier family 35 (UDP-N-acetylglucosamine (UDP-GlcNAc) transporter), member A3
1533	IMAGp998P165227	1.89	0.896	*CYP4F8*	Cytochrome P450, family 4, subfamily F, polypeptide 8
1534	IMAGp998F084507	1.89	0.934	*N/A*	Transcribed locus
1535	RZPDp201H0934D	1.89	0.899	*FAM129B*	Family with sequence similarity 129, member B
1536	IMAGp998J08529	1.89	0.837	*N/A*	Transcribed locus
1537	IMAGp998L14657	1.89	0.850	*N/A*	Transcribed locus
1538	RZPDp1096B0218D	1.89	0.850	*IL6R*	Interleukin 6 receptor
1539	IMAGp998M155471	1.89	0.856	*N/A*	MRNA; cDNA DKFZp686B0610 (from clone DKFZp686B0610)
1540	IMAGp998B21150	1.89	0.894	*N/A*	Transcribed locus
1541	IMAGp998C1384	1.89	0.828	*RBM18*	RNA binding motif protein 18
1542	IMAGp998M244913	1.89	0.855	*N/A*	Transcribed locus
1543	IMAGp998M131943	1.89	0.868	*CRKL*	V-crk sarcoma virus CT10 oncogene homolog (avian)-like
1544	IMAGp998N05444	1.89	0.853	*RBM39*	RNA binding motif protein 39
1545	IMAGp998D19280	1.89	0.928	*MAMDC1*	MAM domain containing 1
1546	IMAGp998D11246	1.89	0.914	*MVD*	Mevalonate (diphospho) decarboxylase
1547	IMAGp998I0384	1.89	0.836	*N/A*	Transcribed locus
1548	IMAGp998D233814	1.89	0.855	*N/A*	Transcribed locus
1549	IMAGp998C1185	1.89	0.851	*N/A*	Data not found
1550	IMAGp998C243954	1.89	0.866	*N/A*	Transcribed locus
1551	IMAGp998E02525	1.89	0.890	*N/A*	Transcribed locus, weakly similar to XP_001163466.1 hypothetical protein isoform 8 [Pan troglodytes]
1552	IMAGp998E17571	1.89	0.845	*CYGB*	Cytoglobin
1553	RZPDp201D0527D	1.89	0.880	*CDC42EP3*	CDC42 effector protein (Rho GTPase binding) 3
1554	IMAGp998F01524	1.89	0.859	*N/A*	Data not found
1555	RZPDp201D0634D	1.89	0.860	*CDK5RAP3*	CDK5 regulatory subunit associated protein 3
1556	IMAGp998C01412	1.89	0.816	*N/A*	Data not found
1557	IMAGp998L012582	1.89	0.860	*ANK3*	Ankyrin 3, node of Ranvier (ankyrin G)
1558	IMAGp998N211897	1.89	0.905	*LTBR*	Lymphotoxin beta receptor (TNFR superfamily, member 3)
1559	IMAGp998J055634	1.89	0.857	*CEP70*	Centrosomal protein 70kDa
1560	IMAGp998P221999	1.89	0.861	*ANXA11*	Annexin A11
1561	RZPDp201G128D	1.89	0.937	*CAMP*	Cathelicidin antimicrobial peptide
1562	IMAGp998B09203	1.89	0.851	*GPR1*	G protein-coupled receptor 1
1563	IMAGp998N144409	1.89	0.872	*N/A*	Transcribed locus
1564	IMAGp998N141781	1.89	0.930	*ATG4B*	ATG4 autophagy related 4 homolog B (S. cerevisiae)
1565	IMAGp998K095575	1.89	0.838	*N/A*	CDNA FLJ12174 fis, clone MAMMA1000707
1566	IMAGp998M16110	1.89	0.828	*N/A*	CDNA FLJ37090 fis, clone BRACE2017587
1567	IMAGp998L2479	1.89	0.841	*N/A*	Data not found
1568	IMAGp998P02129	1.89	0.821	*N/A*	Data not found
1569	RZPDp201C0816D	1.89	0.872	*JAK1*	Janus kinase 1 (a protein tyrosine kinase)
1570	IMAGp998D101784	1.89	0.924	*N/A*	Transcribed locus
1571	IMAGp998F022236	1.89	0.862	*N/A*	Data not found
1572	IMAGp998M244500	1.89	0.879	*LOC645431*	Hypothetical protein LOC645431
1573	IMAGp998F1990	1.89	0.870	*N/A*	MRNA; cDNA DKFZp686E0389 (from clone DKFZp686E0389)
1574	IMAGp998O23136	1.89	0.847	*PHOSPHO1*	Phosphatase, orphan 1
1575	IMAGp998I121795	1.89	0.902	*NPAL2*	NIPA-like domain containing 2
1576	RZPDp202C074D	1.89	0.853	*FLJ12949*	Hypothetical protein FLJ12949
1577	IMAGp998G221825	1.89	0.849	*N/A*	Transcribed locus, moderately similar to XP_001173095.1 general transcription factor IIA, 2, 12kDa isoform 2 [Pan troglodytes]
1578	IMAGp998A022676	1.89	0.867	*N/A*	Transcribed locus
1579	RZPDp202E125D	1.89	0.878	*USP5*	Ubiquitin specific peptidase 5 (isopeptidase T)
1580	IMAGp998N21167	1.89	0.893	*PTN*	Pleiotrophin (heparin binding growth factor 8, neurite growth-promoting factor 1)
1581	IMAGp998F245319	1.89	0.835	*N/A*	Transcribed locus
1582	IMAGp998A22517	1.89	0.903	*PERLD1*	Per1-like domain containing 1
1583	IMAGp998C221070	1.89	0.859	*N/A*	Data not found
1584	IMAGp998H14136	1.89	0.832	*N/A*	Transcribed locus
1585	IMAGp998B07149	1.89	0.891	*M6PR*	Mannose-6-phosphate receptor (cation dependent)
1586	RZPDp201B096D	1.89	0.874	*GSTM5*	Glutathione S-transferase M5
1587	RZPDp202H078D	1.89	0.861	*CCNF*	Cyclin F
1588	IMAGp998B20732	1.89	0.861	*DOT1L*	DOT1-like, histone H3 methyltransferase (S. cerevisia
1589	IMAGp998H08840	1.89	0.909	*C4orf31*	Chromosome 4 open reading frame 31
1590	IMAGp998E18872	1.89	0.857	*N/A*	Transcribed locus, strongly similar to XP_001149562.1 hypothetical protein [Pan troglodytes]
1591	IMAGp998J225506	1.89	0.837	*N/A*	CDNA clone IMAGE:3827723
1592	RZPDp201E0512D	1.89	0.881	*NDUFS3*	NADH dehydrogenase (ubiquinone) Fe-S protein 3, 30kDa (NADH-coenzyme Q reductase)
1593	IMAGp998O221782	1.89	0.925	*MPN2*	Marapsin 2
1594	IMAGp998D105705	1.89	0.926	*N/A*	Data not found
1595	IMAGp998D08129	1.89	0.877	*THRB*	Thyroid hormone receptor, beta (erythroblastic leukemia viral (v-erb-a) oncogene homolog 2, avian)
1596	IMAGp998A182750	1.89	0.869	*N/A*	Data not found
1597	IMAGp998O1997	1.89	0.850	*N/A*	Transcribed locus
1598	IMAGp998L093843	1.89	0.872	*LOC619208*	Hypothetical protein LOC619208
1599	RZPDp1096B013D	1.89	0.869	*VAMP8*	Vesicle-associated membrane protein 8 (endobrevin)
1600	IMAGp998I112011	1.89	0.932	*PSCD1*	Pleckstrin homology, Sec7 and coiled-coil domains 1(cytohesin 1)
1601	IMAGp998M125155	1.89	0.922	*N/A*	Transcribed locus
1602	IMAGp998E223867	1.89	0.863	*N/A*	Data not found
1603	IMAGp998E194881	1.89	0.833	*ZNF614*	Zinc finger protein 614
1604	RZPDp202F093D	1.89	0.910	*MYH14*	Myosin, heavy chain 14
1605	IMAGp998J20137	1.89	0.848	*CCM2*	Cerebral cavernous malformation 2
1606	IMAGp998I051016	1.89	0.853	*N/A*	Transcribed locus
1607	IMAGp998N151747	1.89	0.875	*RHOF*	Ras homolog gene family, member F (in filopodia)
1608	RZPDp201D1028D	1.89	0.825	*KIAA0319L*	KIAA0319-like
1609	RZPDp202E051D	1.89	0.840	*RAB11FIP1*	RAB11 family interacting protein 1 (class I)
1610	IMAGp998F131008	1.89	0.830	*NARG1*	NMDA receptor regulated 1
1611	IMAGp998N17923	1.89	0.879	*N/A*	Transcribed locus
1612	IMAGp998C151780	1.89	0.898	*PLEKHA2*	Pleckstrin homology domain containing, family A (phosphoinositide binding specific) member 2
1613	IMAGp998D08281	1.89	0.861	*WBSCR17*	Williams-Beuren syndrome chromosome region 17
1614	IMAGp998J1792	1.89	0.854	*DONSON*	Downstream neighbor of SON
1615	RZPDp202H062D	1.89	0.837	*ZNF343*	Zinc finger protein 343
1616	RZPDp202A079D	1.89	0.847	*RHOF*	Ras homolog gene family, member F (in filopodia)
1617	IMAGp998E174460	1.89	0.898	*CPN1*	Carboxypeptidase N, polypeptide 1
1618	IMAGp998E025599	1.89	0.874	*N/A*	Transcribed locus
1619	IMAGp998M122038	1.89	0.893	*ZNF519*	Zinc finger protein 519
1620	RZPDp202F011D	1.89	0.846	*TNFRSF10A*	Tumor necrosis factor receptor superfamily, member 10a

1621	RZPDp201C0829D	1.89	0.854	FZD3	Frizzled homolog 3 (Drosophila)
1622	IMAGp998A182035	1.89	0.921	CCDC12	Coiled-coil domain containing 12
1623	IMAGp998C11653	1.89	0.853	SNX5	Sorting nexin 5
1624	IMAGp998L145599	1.89	0.859	N/A	Data not found
1625	IMAGp998D20558	1.89	0.877	ANGEL2	Angel homolog 2 (Drosophila)
1626	IMAGp998J19170	1.89	0.851	N/A	Transcribed locus
1627	IMAGp998I032514	1.89	0.858	N/A	Transcribed locus
1628	IMAGp998I235462	1.89	0.884	PDE7B	Phosphodiesterase 7B
1629	IMAGp998M085524	1.89	0.834	N/A	Transcribed locus
1630	RZPDp201D0629D	1.89	0.842	TMEM67	Transmembrane protein 67
1631	RZPDp1096D0520D	1.89	0.788	SERBP1	SERPINE1 mRNA binding protein 1
1632	IMAGp998H23783	1.89	0.855	KRT1	Keratin 1 (epidermolytic hyperkeratosis)
1633	IMAGp998B092976	1.89	0.850	N/A	Transcribed locus
1634	RZPDp201A0726D	1.89	0.858	GMPPB	GDP-mannose pyrophosphorylase B
1635	RZPDp201G0830D	1.89	0.889	MED25	Mediator of RNA polymerase II transcription, subunit 25 homolog (S. cerevisiae)
1636	IMAGp998C02247	1.89	0.849	HERC2P7	Hect domain and RLD 2 pseudogene 7
1637	IMAGp998M093856	1.89	0.868	N/A	Transcribed locus
1638	RZPDp201A1120D	1.89	0.833	ZNF815	Zinc finger protein 815
1639	IMAGp998O08597	1.89	0.837	N/A	Data not found
1640	IMAGp998E0882	1.89	0.873	SURF4	Surfeit 4
1641	RZPDp201E0515D	1.89	0.857	CPB1	Carboxypeptidase B1 (tissue)
1642	IMAGp998H19147	1.89	0.911	TNFAIP1	Tumor necrosis factor, alpha-induced protein 1 (endothelial)
1643	IMAGp998K16794	1.89	0.855	ST14	Suppression of tumorigenicity 14 (colon carcinoma)
1644	IMAGp998E08142	1.89	0.843	NCDN	Neurochondrin
1645	IMAGp998I22395	1.89	0.887	IL16	Interleukin 16 (lymphocyte chemoattractant factor)
1646	IMAGp998G22678	1.89	0.887	N/A	Transcribed locus
1647	IMAGp998D224914	1.89	0.863	N/A	CDNA clone IMAGE:5266242
1648	IMAGp998P125315	1.89	0.896	N/A	Transcribed locus
1649	IMAGp998E0379	1.89	0.855	N/A	Full length insert cDNA clone YP01H07
1650	IMAGp998I124162	1.89	0.863	N/A	Data not found
1651	IMAGp998O0189	1.89	0.840	C20orf74	Chromosome 20 open reading frame 74
1652	IMAGp998K195908	1.89	0.884	N/A	CDNA FLJ45490 fis, clone BRTHA2005831
1653	IMAGp998I055525	1.89	0.854	N/A	Full length insert cDNA clone ZE05E03
1654	IMAGp998A24920	1.89	0.887	N/A	Transcribed locus
1655	IMAGp998D19152	1.89	0.905	MYST3	MYST histone acetyltransferase (monocytic leukemia) 3
1656	IMAGp998F07649	1.89	0.858	SERINC1	Serine incorporator 1
1657	RZPDp202E094D	1.89	0.920	N/A	Immunoglobulin heavy chain variable region (clone Tmu83)
1658	RZPDp1096A036D	1.89	0.888	FLJ32784	Hypothetical protein FLJ32784
1659	IMAGp998A20579	1.89	0.828	N/A	Transcribed locus
1660	IMAGp998L17314	1.89	0.864	RABEPK	Rab9 effector protein with kelch motifs
1661	RZPDp202H115D	1.89	0.857	C10orf90	Chromosome 10 open reading frame 90
1662	IMAGp998A20412	1.89	0.859	N/A	Transcribed locus
1663	IMAGp998F166077	1.89	0.869	PLCB3	Phospholipase C, beta 3 (phosphatidylinositol-specific)
1664	IMAGp998O16405	1.89	0.882	N/A	Data not found
1665	IMAGp998G20165	1.89	0.868	CEP57	Centrosomal protein 57kDa
1666	RZPDp202B075D	1.89	0.844	RUNDC2B	RUN domain containing 2B
1667	RZPDp1096G0413D	1.89	0.856	SIGLEC11	Sialic acid binding Ig-like lectin 11
1668	RZPDp202G096D	1.89	0.924	N/A	Data not found
1669	IMAGp998L2384	1.89	0.894	N/A	Transcribed locus
1670	IMAGp998A032792	1.89	0.919	C1orf183	Chromosome 1 open reading frame 183
1671	IMAGp998M041825	1.89	0.887	TNRC6B	Trinucleotide repeat containing 6B
1672	RZPDp1096F097D	1.89	0.864	SYT1	Synaptotagmin I
1673	IMAGp998K21260	1.89	0.883	SLC22A18	Solute carrier family 22 (organic cation transporter), member 18
1674	RZPDp202E084D	1.89	0.848	DCLRE1C	DNA cross-link repair 1C (PSO2 homolog, S. cerevisiae)
1675	IMAGp998J065468	1.89	0.872	SPATA18	Spermatogenesis associated 18 homolog (rat)
1676	IMAGp998M085479	1.89	0.867	N/A	Transcribed locus
1677	IMAGp998E17133	1.89	0.869	N/A	CDNA: FLJ21850 fis, clone HEP01929
1678	IMAGp998E125112	1.89	0.861	N/A	Data not found
1679	IMAGp998O18144	1.89	0.925	XAB2	XPA binding protein 2
1680	IMAGp998K016117	1.89	0.863	HNRPU	Heterogeneous nuclear ribonucleoprotein U (scaffold attachment factor A)
1681	IMAGp998L06525	1.89	0.842	N/A	Data not found
1682	IMAGp998B114922	1.89	0.873	N/A	Transcribed locus
1683	IMAGp998L171010	1.89	0.845	N/A	Data not found
1684	IMAGp998M054931	1.89	0.859	N/A	Transcribed locus
1685	IMAGp998G081786	1.89	0.882	EFCBP1	EF-hand calcium binding protein 1
1686	IMAGp998F1188	1.89	0.849	KLF11	Kruppel-like factor 11
1687	IMAGp998F04273	1.89	0.847	N/A	MRNA full length insert cDNA clone EUROIMAGE 46506
1688	IMAGp998B08778	1.89	0.877	PPARA	Peroxisome proliferator-activated receptor alpha
1689	RZPDp201G0315D	1.89	0.856	CEACAM5	Carcinoembryonic antigen-related cell adhesion molecule 5
1690	IMAGp998E11286	1.89	0.919	RBBP8	Retinoblastoma binding protein 8
1691	RZPDp201B124D	1.89	0.936	DEFB1	Defensin, beta 1
1692	IMAGp998H231999	1.89	0.936	ANKRD52	Ankyrin repeat domain 52
1693	IMAGp998D22209	1.89	0.922	TMEM80	Transmembrane protein 80
1694	IMAGp998L13385	1.89	0.846	N/A	Transcribed locus
1695	IMAGp998P01238	1.89	0.857	FLJ23834	Hypothetical protein FLJ23834
1696	IMAGp998K18520	1.89	0.867	N/A	CDNA FLJ32200 fis, clone PLACE6002871
1697	IMAGp998B02331	1.89	0.885	PDE1B	Phosphodiesterase 1B, calmodulin-dependent
1698	IMAGp998O13663	1.89	0.869	COL18A1	Collagen, type XVIII, alpha 1
1699	IMAGp998F03282	1.89	0.876	MECR	Mitochondrial trans-2-enoyl-CoA reductase
1700	IMAGp998L045455	1.89	0.854	N/A	Transcribed locus
1701	IMAGp998D20381	1.89	0.865	TRIM39	Tripartite motif-containing 39
1702	IMAGp998E1671	1.89	0.857	N/A	Transcribed locus
1703	IMAGp998B055155	1.89	0.916	N/A	Data not found
1704	IMAGp998G18523	1.89	0.849	FANCC	Fanconi anemia, complementation group C
1705	IMAGp998L244500	1.89	0.927	C1orf102	Chromosome 1 open reading frame 102
1706	IMAGp998C023999	1.89	0.854	N/A	Data not found
1707	RZPDp1096G0214D	1.89	0.856	C6orf157	Chromosome 6 open reading frame 157
1708	IMAGp998M084779	1.89	0.943	N/A	Data not found
1709	IMAGp998G02114	1.89	0.861	N/A	Data not found
1710	IMAGp998B024914	1.89	0.853	ZNF532	Zinc finger protein 532
1711	IMAGp998K011928	1.89	0.890	MAP3K2	Mitogen-activated protein kinase kinase kinase 2
1712	IMAGp998I124614	1.89	0.861	N/A	Transcribed locus
1713	IMAGp998A151858	1.89	0.852	N/A	Transcribed locus
1714	IMAGp998E05583	1.89	0.894	KIAA1033	KIAA1033
1715	IMAGp998O045512	1.89	0.871	CARD11	Caspase recruitment domain family, member 11
1716	IMAGp998N185376	1.89	0.864	LIN9	Lin-9 homolog (C. elegans)
1717	IMAGp998A061905	1.89	0.883	UGT2B10	UDP glucuronosyltransferase 2 family, polypeptide B10
1718	IMAGp998F23544	1.89	0.864	KCNAB2	Potassium voltage-gated channel, shaker-related subfamily, beta member 2
1719	IMAGp998M035453	1.89	0.844	N/A	Transcribed locus
1720	RZPDp202D036D	1.89	0.884	MARCH4	Membrane-associated ring finger (C3HC4) 4
1721	IMAGp998P11882	1.89	0.841	N/A	Transcribed locus
1722	IMAGp998F23529	1.89	0.848	N/A	CDNA clone IMAGE:4794941
1723	IMAGp998J185497	1.89	0.874	ZNF43	Zinc finger protein 43
1724	IMAGp998O12872	1.89	0.846	N/A	Data not found
1725	RZPDp202D115D	1.89	0.874	MARCH3	Membrane-associated ring finger (C3HC4) 9
1726	IMAGp998B06472	1.89	0.878	FVT1	Follicular lymphoma variant translocation 1
1727	IMAGp998D105862	1.89	0.862	N/A	Transcribed locus
1728	IMAGp998J13267	1.89	0.857	N/A	Transcribed locus
1729	IMAGp998J134890	1.89	0.891	N/A	Transcribed locus
1730	IMAGp998A244174	1.89	0.935	N/A	Transcribed locus
1731	IMAGp998C045590	1.89	0.865	N/A	Data not found
1732	IMAGp998L21268	1.89	0.848	N/A	CDNA FLJ38867 fis, clone MESAN2013183
1733	IMAGp998F243582	1.89	0.882	N/A	Transcribed locus
1734	IMAGp998H1877	1.89	0.866	RAB27B	RAB27B, member RAS oncogene family
1735	IMAGp998A02618	1.89	0.915	C14orf22	Chromosome 14 open reading frame 22
1736	IMAGp998D113104	1.89	0.864	N/A	Transcribed locus
1737	IMAGp998P10837	1.89	0.860	N/A	Data not found
1738	IMAGp998B042332	1.89	0.900	LOC553103	Hypothetical LOC553103
1739	IMAGp998N10121	1.89	0.861	N/A	Transcribed locus
1740	IMAGp998I0682	1.89	0.830	N/A	CDNA FLJ42015 fis, clone SPLEN2032813
1741	IMAGp998M064359	1.89	0.860	N/A	CDNA clone IMAGE:5000386, containing frame-shift errors
1742	IMAGp998L15267	1.89	0.902	STOML1	Stomatin (EPB72)-like 1
1743	IMAGp998C24528	1.89	0.873	ZRANB1	Zinc finger, RAN-binding domain containing 1
1744	IMAGp998K03127	1.89	0.850	MT1M	Metallothionein 1M
1745	IMAGp998C03119	1.89	0.910	RAB3GAP1	RAB3 GTPase activating protein subunit 1 (catalytic)
1746	IMAGp998P24892	1.89	0.859	N/A	CDNA: FLJ22133 fis, clone HEP20529
1747	IMAGp998L01869	1.89	0.862	XPNPEP2	X-prolyl aminopeptidase (aminopeptidase P) 2, membrane-bound
1748	IMAGp998B15639	1.89	0.863	N/A	Transcribed locus, strongly similar to XP_001148422.1 similar to putative homeobox protein [Pan troglodytes]
1749	RZPDp1096B0219D	1.89	0.853	FAM3A	Family with sequence similarity 3, member A
1750	IMAGp998H035496	1.89	0.867	GCNT3	Glucosaminyl (N-acetyl) transferase 3, mucin type
1751	IMAGp998L093852	1.89	0.934	TMEM63C	Transmembrane protein 63C
1752	RZPDp201A068D	1.89	0.863	ZDHHC15	Zinc finger, DHHC-type containing 15
1753	IMAGp998M035470	1.89	0.871	N/A	Data not found
1754	IMAGp998F025614	1.89	0.855	N/A	Transcribed locus
1755	IMAGp998L215379	1.89	0.864	BAT1	HLA-B associated transcript 1

1756	RZPDp202C103D	1.89	0.847	*LOC730005*	Similar to SEC14p-like protein TAP3
1757	IMAGp998G15286	1.89	0.850	*ACTL6B*	Actin-like 6B
1758	RZPDp202B019D	1.89	0.842	*FCHO2*	FCH domain only 2
1759	IMAGp998A185586	1.89	0.875	*TRIO*	Triple functional domain (PTPRF interacting)
1760	IMAGp998D215786	1.89	0.875	*AK7*	Adenylate kinase 7
1761	IMAGp998J234893	1.89	0.854	*CXorf41*	Chromosome X open reading frame 41
1762	IMAGp998P154521	1.89	0.851	*ZNF425*	Zinc finger protein 425
1763	RZPDp202B032D	1.89	0.844	*RPS15*	Ribosomal protein S15
1764	IMAGp998A164332	1.89	0.863	*HSPA12B*	Heat shock 70kD protein 12B
1765	IMAGp998O22588	1.89	0.853	*BMPR1A*	Bone morphogenetic protein receptor, type IA
1766	IMAGp998A095942	1.89	0.922	*NPEPL1*	Aminopeptidase-like 1
1767	IMAGp998G086101	1.89	0.862	*STXBP6*	Syntaxin binding protein 6 (amisyn)
1768	IMAGp998P19177	1.89	0.897	*NTRK3*	Neurotrophic tyrosine kinase, receptor, type 3
1769	IMAGp998J183954	1.89	0.938	*N/A*	Transcribed locus
1770	IMAGp998B095857	1.89	0.930	*N/A*	MRNA; cDNA DKFZp761O1910 (from clone DKFZp761O1910)
1771	IMAGp998K065602	1.89	0.882	*ZDHHC1*	Zinc finger, DHHC-type containing 1
1772	RZPDp201C1115D	1.89	0.858	*FLJ13236*	Hypothetical protein FLJ13236
1773	IMAGp998F055668	1.89	0.918	*ANP32E*	Acidic (leucine-rich) nuclear phosphoprotein 32 family, member E
1774	IMAGp998D14441	1.89	0.839	*DIP2C*	DIP2 disco-interacting protein 2 homolog C (Drosophila)
1775	IMAGp998O1473	1.89	0.900	*N/A*	Transcribed locus
1776	IMAGp998J10836	1.89	0.874	*N/A*	Data not found
1777	RZPDp202E065D	1.89	0.883	*DOLK*	Dolichol kinase
1778	IMAGp998P041933	1.89	0.917	*WDR22*	WD repeat domain 22
1779	IMAGp998A12270	1.89	0.898	*FAT4*	FAT tumor suppressor homolog 4 (Drosophila)
1780	IMAGp998O04592	1.89	0.898	*L1CAM*	L1 cell adhesion molecule
1781	IMAGp998E18526	1.89	0.858	*N/A*	Data not found
1782	IMAGp998G08119	1.89	0.921	*MMS19L*	MMS19-like (MET18 homolog, S. cerevisiae)
1783	IMAGp998A225468	1.89	0.835	*PKP4*	Plakophilin 4
1784	IMAGp998E12654	1.89	0.827	*SORL1*	Sortilin-related receptor, L(DLR class) A repeats-containing
1785	RZPDp201G075D	1.89	0.868	*P53AIP1*	P53-regulated apoptosis-inducing protein 1
1786	IMAGp998J20561	1.89	0.909	*N/A*	Transcribed locus
1787	IMAGp998D06137	1.89	0.881	*CUGBP1*	CUG triplet repeat, RNA binding protein 1
1788	IMAGp998K075264	1.89	0.871	*N/A*	Transcribed locus
1789	IMAGp998E101866	1.89	0.947	*HOXD11*	Homeobox D11
1790	RZPDp202H077D	1.89	0.870	*PFN1*	Profilin 1
1791	IMAGp998E055648	1.89	0.860	*N/A*	Transcribed locus
1792	IMAGp998O17386	1.89	0.845	*USP34*	Ubiquitin specific peptidase 34
1793	IMAGp998K06192	1.89	0.839	*EEA1*	Early endosome antigen 1
1794	IMAGp998G18258	2.75	0.850	*TEGT*	Testis enhanced gene transcript (BAX inhibitor 1)
1795	IMAGp998P011825	2.75	0.870	*N/A*	Transcribed locus
1796	IMAGp998C16174	2.75	0.877	*NDRG4*	NDRG family member 4
1797	IMAGp998G07537	2.75	0.857	*N/A*	Data not found
1798	IMAGp998L031863	2.75	0.869	*N/A*	Data not found
1799	IMAGp998C16677	2.75	0.935	*N/A*	Transcribed locus
1800	IMAGp998N144185	2.75	0.918	*N/A*	CDNA FLJ11504 fis, clone HEMBA1002119
1801	IMAGp998N12884	2.75	0.859	*N/A*	Data not found
1802	IMAGp998J082266	2.75	0.834	*N/A*	Transcribed locus
1803	IMAGp998E13175	2.75	0.839	*N/A*	Data not found
1804	RZPDp202H076D	2.75	0.871	*NMUR1*	Neuromedin U receptor 1
1805	IMAGp998L1769	2.75	0.858	*N/A*	Transcribed locus
1806	IMAGp998O0877	2.75	0.860	*N/A*	Transcribed locus
1807	IMAGp998E132584	2.75	0.879	*N/A*	CDNA FLJ32438 fis, clone SKMUS2001402
1808	RZPDp202E033D	2.75	0.908	*ECHDC2*	Enoyl Coenzyme A hydratase domain containing 2
1809	RZPDp201A0536D	2.75	0.872	*SMCR7L*	Smith-Magenis syndrome chromosome region, candidate 7-like
1810	IMAGp998H11248	2.75	0.880	*N/A*	Transcribed locus
1811	IMAGp998H203891	2.75	0.863	*CIITA*	Class II, major histocompatibility complex, transactivator
1812	IMAGp998A135467	2.75	0.869	*N/A*	Transcribed locus
1813	RZPDp202A124D	2.75	0.866	*SLC26A6*	Solute carrier family 26, member 6
1814	IMAGp998H21732	2.75	0.897	*C20orf30*	Chromosome 20 open reading frame 30
1815	IMAGp998A105936	2.75	0.845	*LOC648556*	Uncharacterized gastric protein ZA43P
1816	IMAGp998D225779	2.75	0.921	*CMYA1*	Cardiomyopathy associated 1
1817	RZPDp1096E072D	2.75	0.897	*MYO1D*	Myosin ID
1818	IMAGp998I0492	2.75	0.870	*IDH1*	Isocitrate dehydrogenase 1 (NADP+), soluble
1819	IMAGp998P165515	2.75	0.865	*PFAS*	Phosphoribosylformylglycinamidine synthase (FGAR amidotransferase)
1820	IMAGp998O14155	2.75	0.865	*ZNF365*	Zinc finger protein 365
1821	IMAGp998E035633	2.75	0.855	*IDH1*	Isocitrate dehydrogenase 1 (NADP+), soluble
1822	IMAGp998K01349	2.75	0.902	*TBCD*	Tubulin folding cofactor D
1823	IMAGp998M14198	2.75	0.847	*N/A*	Full length insert cDNA YH96F07
1824	IMAGp998A034171	2.75	0.919	*hCG_2042202*	Similar to zinc finger and SCAN domain containing 5
1825	IMAGp998O05143	2.75	0.866	*PLCE1*	Phospholipase C, epsilon 1
1826	IMAGp998I155558	2.75	0.946	*CTF8*	Chromosome transmission fidelity factor 8 homolog (S. cerevisiae)
1827	Ara-LTP4-2	2.75	0.938	*N/A*	Data not found
1828	RZPDp201E034D	2.75	0.867	*MMACHC*	Methylmalonic aciduria (cobalamin deficiency) cblC type, with homocystinuria
1829	IMAGp998F184757	2.75	0.859	*MCART1*	Mitochondrial carrier triple repeat 1
1830	IMAGp998I024920	2.75	0.907	*ZNF182*	Zinc finger protein 182
1831	IMAGp998D09783	2.75	0.896	*KCNRG*	Potassium channel regulator
1832	RZPDp1096A1117D	2.75	0.900	*HTATSF1*	HIV-1 Tat specific factor 1
1833	IMAGp998F154914	2.75	0.880	*N/A*	Transcribed locus
1834	IMAGp998F09658	2.75	0.898	*C17orf32*	Chromosome 17 open reading frame 32
1835	IMAGp998M23279	2.75	0.905	*FLOT2*	Flotillin 2
1836	IMAGp998E19273	2.75	0.908	*N/A*	MRNA; cDNA DKFZp761P2314 (from clone DKFZp761P2314)
1837	IMAGp998P035265	2.75	0.859	*N/A*	Full-length cDNA clone CS0CAP004YK07 of Thymus of Homo sapiens (human)
1838	IMAGp998M071897	2.75	0.927	*VEGFB*	Vascular endothelial growth factor B
1839	IMAGp998I23276	2.75	0.874	*LRRN2*	Leucine rich repeat neuronal 2
1840	IMAGp998E03436	2.75	0.866	*N/A*	Data not found
1841	IMAGp998H0818	2.75	0.865	*N/A*	Data not found
1842	IMAGp998M14208	2.75	0.845	*N/A*	Transcribed locus
1843	RZPDp201D0915D	2.75	0.867	*N/A*	Data not found
1844	RZPDp1096D0718D	2.75	0.863	*SMC1A*	Structural maintenance of chromosomes 1A
1845	IMAGp998O214141	2.75	0.856	*N/A*	CDNA clone IMAGE:4823793
1846	IMAGp998F02174	2.75	0.930	*GPR162*	G protein-coupled receptor 162
1847	IMAGp998K201818	2.75	0.864	*GTSE1*	G-2 and S-phase expressed 1
1848	IMAGp998I163968	2.75	0.945	*TTC12*	Tetratricopeptide repeat domain 12
1849	IMAGp998F111004	2.75	0.836	*CFLAR*	CASP8 and FADD-like apoptosis regulator
1850	IMAGp998M093964	2.75	0.906	*N/A*	Transcribed locus
1851	IMAGp998H034356	2.75	0.855	*PQLC2*	PQ loop repeat containing 2
1852	IMAGp998E246083	2.75	0.877	*N/A*	CDNA FLJ30967 fis, clone HEART2000309, weakly similar to PTB-ASSOCIATED SPLICING FACTOR
1853	IMAGp998C01736	2.75	0.914	*C14orf179*	Chromosome 14 open reading frame 179
1854	RZPDp1096B0919D	2.75	0.843	*FANCA*	Fanconi anemia, complementation group A
1855	IMAGp998F19514	2.75	0.877	*ZFP3*	Zinc finger protein 3 homolog (mouse)
1856	IMAGp998E071162	2.75	0.893	*TLE2*	Transducin-like enhancer of split 2 (E(sp1) homolog, Drosophila)
1857	IMAGp998L14728	2.75	0.898	*SMPDL3B*	Sphingomyelin phosphodiesterase, acid-like 3B
1858	IMAGp998C05268	2.75	0.847	*ANKRD37*	Ankyrin repeat domain 37
1859	IMAGp998N16153	2.75	0.876	*N/A*	Data not found
1860	IMAGp998B24514	2.75	0.888	*MYO1D*	Myosin ID
1861	IMAGp998I24241	2.75	0.882	*FBXO6*	F-box protein 6
1862	IMAGp998I20530	2.75	0.924	*MEST*	Mesoderm specific transcript homolog (mouse)
1863	IMAGp998M22268	2.75	0.868	*N/A*	Data not found
1864	RZPDp202A036D	2.75	0.892	*CSTF2*	Cleavage stimulation factor, 3' pre-RNA, subunit 2, 64kDa
1865	RZPDp201F0915D	2.75	0.923	*PHACS*	1-aminocyclopropane-1-carboxylate synthase
1866	IMAGp998L09693	2.75	0.888	*CGNL1*	Cingulin-like 1
1867	IMAGp998B18275	2.75	0.881	*N/A*	Clone 23911 mRNA sequence
1868	IMAGp998D051063	2.75	0.868	*PHKA2*	Phosphorylase kinase, alpha 2 (liver)
1869	IMAGp998M17435	2.75	0.880	*CCDC90A*	Coiled-coil domain containing 90A
1870	IMAGp998B16143	2.75	0.898	*DUSP7*	Dual specificity phosphatase 7
1871	IMAGp998I04828	2.75	0.877	*LOC647059*	Similar to tubulin, alpha 8 like
1872	IMAGp998F19868	2.75	0.891	*GPAM*	Glycerol-3-phosphate acyltransferase, mitochondrial
1873	RZPDp1096H066D	2.75	0.887	*PELO*	Pelota homolog (Drosophila)
1874	IMAGp998J106085	2.75	0.880	*N/A*	Data not found
1875	IMAGp998J15175	2.75	0.870	*N/A*	Data not found
1876	IMAGp998A241825	2.75	0.932	*KRTAP5-9*	Keratin associated protein 5-9
1877	IMAGp998G11688	2.75	0.870	*LOC149478*	Hypothetical protein LOC149478
1878	IMAGp998O22166	2.75	0.863	*N/A*	Transcribed locus
1879	IMAGp998K084922	2.75	0.888	*MAP2K5*	Mitogen-activated protein kinase kinase 5
1880	IMAGp998P01378	2.75	0.858	*N/A*	Transcribed locus
1881	IMAGp998D146082	2.75	0.856	*N/A*	CDNA: FLJ22522 fis, clone HRC12491
1882	IMAGp998K13587	2.75	0.883	*CRIM1*	Cysteine rich transmembrane BMP regulator 1 (chordin-like)
1883	IMAGp998P03536	2.75	0.871	*N/A*	Data not found
1884	IMAGp998B02214	2.75	0.881	*ITGB4BP*	Integrin beta 4 binding protein
1885	IMAGp998D213240	2.75	0.910	*N/A*	Transcribed locus
1886	RZPDp1096E123D	2.75	0.869	*RAB40A*	RAB40A, member RAS oncogene family
1887	IMAGp998L05281	2.75	0.926	*PLXNA1*	Plexin A1
1888	IMAGp998K215833	2.75	0.930	*LOC401176*	Hypothetical gene supported by BC043001
1889	IMAGp998F194878	2.75	0.860	*N/A*	Data not found
1890	IMAGp998D192676	2.75	0.856	*N/A*	CDNA FLJ42548 fis, clone BRACE3004996

1891	IMAGp998H22381	2.75	0.865	*CDCA2*	Cell division cycle associated 2
1892	IMAGp998F044550	2.75	0.888	*N/A*	Transcribed locus
1893	IMAGp998O16617	2.75	0.913	*ANKRD18A*	Ankyrin repeat domain 18A
1894	IMAGp998K1884	2.75	0.887	*N/A*	Data not found
1895	IMAGp998I181007	2.75	0.880	*PIGS*	Phosphatidylinositol glycan anchor biosynthesis, class S
1896	IMAGp998D215063	2.75	0.922	*N/A*	Data not found
1897	IMAGp998P244500	2.75	0.891	*N/A*	Transcribed locus
1898	IMAGp998H01176	2.75	0.898	*PPP1R13B*	Protein phosphatase 1, regulatory (inhibitor) subunit 13B
1899	IMAGp998N19678	2.75	0.872	*PRR6*	Proline rich 6
1900	IMAGp998E06180	2.75	0.865	*PECAM1*	Platelet/endothelial cell adhesion molecule (CD31 antigen)
1901	IMAGp998O18150	2.75	0.881	*N/A*	CDNA clone IMAGE:4819084
1902	IMAGp998G15244	2.75	0.879	*GPR137*	G protein-coupled receptor 137
1903	RZPDp201E1018D	2.75	0.937	*ZNF462*	Zinc finger protein 462
1904	IMAGp998L111748	2.75	0.854	*LOC90784*	Hypothetical protein LOC90784
1905	IMAGp998C091944	2.75	0.940	*CNOT3*	CCR4-NOT transcription complex, subunit 3
1906	RZPDp201B0527D	2.75	0.894	*STRN*	Striatin, calmodulin binding protein
1907	IMAGp998K12415	2.75	0.896	*IDH3B*	Isocitrate dehydrogenase 3 (NAD+) beta
1908	IMAGp998C21401	2.75	0.862	*SAP130*	Sin3A-associated protein, 130kDa
1909	IMAGp998O076083	2.75	0.869	*N/A*	CDNA FLJ41751 fis, clone HSYRA2008154
1910	RZPDp1096G0913D	2.75	0.912	*PLXNA1*	Plexin A1
1911	RZPDp202E102D	2.75	0.826	*NBPF1*	Neuroblastoma breakpoint family, member 1
1912	IMAGp998K23156	2.75	0.908	*TAF6*	TAF6 RNA polymerase II, TATA box binding protein (TBP)-associated factor, 80kDa
1913	IMAGp998I04362	2.75	0.870	*ACOT12*	Acyl-CoA thioesterase 12
1914	IMAGp998P02594	2.75	0.874	*N/A*	Data not found
1915	IMAGp998D02274	2.75	0.868	*N/A*	Transcribed locus
1916	IMAGp998I056087	2.75	0.867	*CSPP1*	Centrosome and spindle pole associated protein 1
1917	IMAGp998D145397	2.75	0.864	*SLC1A4*	Solute carrier family 1 (glutamate/neutral amino acid transporter), member 4
1918	IMAGp998J02369	2.75	0.847	*N/A*	Data not found
1919	IMAGp998K24269	2.75	0.854	*FREQ*	Frequenin homolog (Drosophila)
1920	IMAGp998G03172	2.75	0.856	*N/A*	Transcribed locus
1921	IMAGp998G091791	2.75	0.882	*KCNJ10*	Potassium inwardly-rectifying channel, subfamily J, member 10
1922	IMAGp998G094962	2.75	0.924	*N/A*	Data not found
1923	IMAGp998C09166	2.75	0.863	*N/A*	Transcribed locus, strongly similar to XP_001148628.1 carnitine palmitoyltransferase II [Pan troglodytes]
1924	RZPDp1096G044D	2.75	0.869	*PNRC2*	Proline-rich nuclear receptor coactivator 2
1925	IMAGp998E234462	2.75	0.892	*N/A*	Transcribed locus
1926	RZPDp202C011D	2.75	0.916	*LOC389833*	Similar to hypothetical protein MGC27019
1927	IMAGp998A03781	2.75	0.891	*MAN1B1*	Mannosidase, alpha, class 1B, member 1
1928	IMAGp998H186070	2.75	0.877	*PLA2G4A*	Phospholipase A2, group IVA (cytosolic, calcium-dependent)
1929	RZPDp201D041D	2.75	0.910	*LOC400924*	Hypothetical gene supported by AK056895
1930	IMAGp998P025495	2.75	0.918	*L3MBTL2*	L(3)mbt-like 2 (Drosophila)
1931	IMAGp998I015616	2.75	0.861	*N/A*	Transcribed locus
1932	IMAGp998O17210	2.75	0.857	*N/A*	Transcribed locus
1933	IMAGp998P233150	2.75	0.867	*N/A*	Transcribed locus
1934	IMAGp998M05462	2.75	0.916	*HOXA11*	Homeobox A11
1935	IMAGp998A101934	2.75	0.930	*TRIM55*	Tripartite motif-containing 55
1936	IMAGp998I13246	2.75	0.941	*KLHL25*	Kelch-like 25 (Drosophila)
1937	IMAGp998K151963	2.75	0.858	*PALM2-AKAP2*	PALM2-AKAP2 protein
1938	IMAGp998G214819	2.75	0.863	*MGC42367*	Similar to 2010300C02Rik protein
1939	IMAGp998M193500	2.75	0.895	*LOC729799*	Similar to SEC14-like protein 1
1940	IMAGp998P05139	2.75	0.851	*N/A*	CDNA FLJ40127 fis, clone TESTI2011294
1941	IMAGp998K1469	2.75	0.853	*N/A*	Data not found
1942	IMAGp998E21254	2.75	0.921	*ING4*	Inhibitor of growth family, member 4
1943	IMAGp998N085685	2.75	0.923	*N/A*	Transcribed locus
1944	IMAGp998I061933	2.75	0.918	*MMP15*	Matrix metallopeptidase 15 (membrane-inserted)
1945	RZPDp202C121D	2.75	0.871	*PIF1*	PIF1 5'-to-3' DNA helicase homolog (S. cerevisiae)
1946	RZPDp201E058D	2.75	0.931	*CLDND2*	Claudin domain containing 2
1947	RZPDp1096A017D	2.75	0.870	*CUGBP1*	CUG triplet repeat, RNA binding protein 1
1948	IMAGp998K1878	2.75	0.933	*C17orf81*	Chromosome 17 open reading frame 81
1949	RZPDp202C026D	2.75	0.898	*NBLA00301*	Putative protein product of Nbla00301
1950	IMAGp998I04261	2.75	0.885	*COQ10B*	Coenzyme Q10 homolog B (S. cerevisiae)
1951	IMAGp998G2189	2.75	0.893	*NOM1*	Nucleolar protein with MIF4G domain 1
1952	RZPDp201G126D	2.75	0.906	*GRHL3*	Grainyhead-like 3 (Drosophila)
1953	IMAGp998A144210	2.75	0.871	*N/A*	CDNA FLJ41780 fis, clone IMR322017049
1954	RZPDp201D0335D	2.75	0.867	*MRPS16*	Mitochondrial ribosomal protein S16
1955	IMAGp998P20470	2.75	0.875	*N/A*	Transcribed locus
1956	IMAGp998M095465	2.75	0.859	*N/A*	Transcribed locus
1957	IMAGp998H081935	2.75	0.874	*RC3H2*	Ring finger and CCCH-type zinc finger domains 2
1958	IMAGp998L175762	2.75	0.856	*N/A*	Transcribed locus, strongly similar to XP_001136682.1 hypothetical protein [Pan troglodytes]
1959	RZPDp1096B1117D	2.75	0.905	*FAM43A*	Family with sequence similarity 43, member A
1960	IMAGp998N09214	2.75	0.865	*VGLL3*	Vestigial like 3 (Drosophila)
1961	IMAGp998F24442	2.75	0.850	*NTNG2*	Netrin G2
1962	IMAGp998D084893	2.75	0.856	*N/A*	Transcribed locus
1963	IMAGp998C151162	2.75	0.898	*TRIOBP*	TRIO and F-actin binding protein
1964	IMAGp998F014736	2.75	0.883	*SOLH*	Small optic lobes homolog (Drosophila)
1965	RZPDp1096H035D	2.75	0.863	*ZNF530*	Zinc finger protein 530
1966	IMAGp998O164203	2.75	0.899	*ZNF70*	Zinc finger protein 70
1967	RZPDp202H114D	2.75	0.924	*NPEPL1*	Aminopeptidase-like 1
1968	IMAGp998G19655	2.75	0.917	*TBC1D14*	TBC1 domain family, member 14
1969	IMAGp998K225633	2.75	0.861	*PARVG*	Parvin, gamma
1970	IMAGp998H225379	2.75	0.872	*N/A*	CDNA clone IMAGE:5263455
1971	IMAGp998K035617	2.75	0.863	*N/A*	Transcribed locus
1972	IMAGp998P19275	2.75	0.922	*N/A*	CDNA FLJ38434 fis, clone FEBRA2014939
1973	IMAGp998N03921	2.75	0.926	*LOC646778*	Hypothetical LOC646778
1974	IMAGp998E182574	2.75	0.882	*SPAG17*	Sperm associated antigen 17
1975	IMAGp998A235666	2.75	0.867	*N/A*	Homo sapiens, clone IMAGE:4295366, mRNA
1976	IMAGp998G215958	2.75	0.916	*LOC729710*	Hypothetical protein LOC729710
1977	IMAGp998M165875	2.75	0.860	*N/A*	Transcribed locus
1978	IMAGp998K01313	2.75	0.859	*N/A*	Data not found
1979	IMAGp998N24870	2.75	0.945	*YIPF3*	Yip1 domain family, member 3
1980	RZPDp201E0133D	2.75	0.867	*CASP2*	Caspase 2, apoptosis-related cysteine peptidase (neural precursor cell expressed, developmentally down-regulated 2)
1981	IMAGp998G242001	2.75	0.926	*MFSD7*	Major facilitator superfamily domain containing 7
1982	IMAGp998H135079	2.75	0.911	*ASCL2*	Achaete-scute complex homolog 2 (Drosophila)
1983	IMAGp998C055085	2.75	0.857	*N/A*	Transcribed locus
1984	IMAGp998P142228	2.75	0.873	*GIMAP6*	GTPase, IMAP family member 6
1985	IMAGp998H015712	2.75	0.883	*N/A*	CDNA FLJ33772 fis, clone BRSSN2000175
1986	IMAGp998N091943	2.75	0.936	*AMY2B*	Amylase, alpha 2B (pancreatic)
1987	RZPDp1096D1216D	2.75	0.890	*C11orf31*	Chromosome 11 open reading frame 31
1988	RZPDp202G112D	2.75	0.885	*SYT3*	Synaptotagmin III
1989	Ara-CAB-7	2.75	0.943	*N/A*	Data not found
1990	IMAGp998D06442	2.75	0.893	*ZNF295*	Zinc finger protein 295
1991	IMAGp998A21416	2.75	0.870	*CDC42SE2*	CDC42 small effector 2
1992	IMAGp998B04266	2.75	0.853	*LOC286189*	Hypothetical protein LOC286189
1993	IMAGp998A23408	2.75	0.891	*NAPE-PLD*	N-acyl-phosphatidylethanolamine-hydrolyzing phospholipase D
1994	IMAGp998F042616	2.75	0.848	*IRAK1BP1*	Interleukin-1 receptor-associated kinase 1 binding protein 1
1995	IMAGp998L104161	2.75	0.900	*N/A*	Transcribed locus
1996	RZPDp201G013D	2.75	0.866	*HMFN0672*	Hypothetical gene supported by AK129923
1997	RZPDp201A0233D	2.75	0.858	*NUP43*	Nucleoporin 43kDa
1998	IMAGp998J223812	2.75	0.944	*N/A*	Transcribed locus, moderately similar to XP_001138481.1 similar to variably charged X-C [Pan troglodytes]
1999	IMAGp998N12732	2.75	0.879	*EDG3*	Endothelial differentiation, sphingolipid G-protein-coupled receptor, 3
2000	IMAGp998O06135	2.75	0.866	*N/A*	Data not found
2001	IMAGp998A03630	2.75	0.872	*N/A*	Data not found
2002	IMAGp998M203863	2.75	0.897	*N/A*	Transcribed locus
2003	IMAGp998B07138	2.75	0.847	*N/A*	Data not found
2004	IMAGp998G22215	2.75	0.859	*N/A*	Data not found
2005	IMAGp998P075498	2.75	0.862	*N/A*	CDNA FLJ38345 fis, clone FCBBF3028671
2006	IMAGp998A085590	2.75	0.878	*KIF15*	Kinesin family member 15
2007	IMAGp998L11782	2.75	0.937	*FAM73B*	Family with sequence similarity 73, member B
2008	RZPDp201B0528D	2.75	0.855	*SLC39A11*	Solute carrier family 39 (metal ion transporter), member 11
2009	RZPDp202D013D	2.75	0.884	*PMS2CL*	PMS2-C terminal-like
2010	IMAGp998D02240	2.75	0.859	*N/A*	Transcribed locus, strongly similar to NP_037390.2 and CARD domain containing isoform a [Homo sapiens]
2011	RZPDp1096H086D	2.75	0.866	*LUZP1*	Leucine zipper protein 1
2012	IMAGp998O085470	2.75	0.879	*KHDRBS1*	KH domain containing, RNA binding, signal transduction associated 1
2013	IMAGp998E043495	2.75	0.866	*N/A*	Transcribed locus
2014	IMAGp998C155310	2.75	0.864	*N/A*	Data not found
2015	IMAGp998G03130	2.75	0.858	*N/A*	Transcribed locus
2016	IMAGp998L076080	2.75	0.895	*N/A*	CDNA: FLJ22706 fis, clone HSI13163
2017	RZPDp201G065D	2.75	0.903	*CACNA2D2*	Calcium channel, voltage-dependent, alpha 2/delta subunit 2
2018	IMAGp998M20676	2.75	0.939	*POMGNT1*	Protein O-linked mannose beta1,2-N-acetylglucosaminyltransferase
2019	IMAGp998G02844	2.75	0.864	*C14orf167*	Chromosome 14 open reading frame 167
2020	IMAGp998O21977	2.75	0.890	*CRLS1*	Cardiolipin synthase 1
2021	IMAGp998C023842	2.75	0.882	*ZNF530*	Zinc finger protein 530
2022	RZPDp201E0817D	2.75	0.902	*TTL*	Tubulin tyrosine ligase
2023	RZPDp201A116D	2.75	0.924	*ARGFX*	Arginine-fifty homeobox
2024	IMAGp998L13399	2.75	0.867	*EPB41*	Erythrocyte membrane protein band 4.1 (elliptocytosis 1, RH-linked)
2025	IMAGp998L06395	2.75	0.863	*N/A*	CDNA FLJ13434 fis, clone PLACE1002578

2026	IMAGp998G17271	2.75	0.894	*HTRA1*	HtrA serine peptidase 1
2027	IMAGp998C12887	2.75	0.880	*TRH*	Thyrotropin-releasing hormone
2028	IMAGp998I194413	2.75	0.888	*N/A*	Transcribed locus
2029	RZPDp201A1033D	2.75	0.918	*CACNA2D4*	Calcium channel, voltage-dependent, alpha 2/delta subunit 4
2030	IMAGp998N12248	2.75	0.890	*AFG3L2*	AFG3 ATPase family gene 3-like 2 (yeast)
2031	RZPDp201C119D	2.75	0.920	*CMA1*	Chymase 1, mast cell
2032	IMAGp998N10974	2.75	0.880	*LTF*	Lactotransferrin
2033	IMAGp998M185412	2.75	0.936	*N/A*	CDNA FLJ36875 fis, clone ASTRO2019039
2034	IMAGp998F02387	2.75	0.868	*N/A*	Data not found
2035	IMAGp998I051897	2.75	0.929	*ZNHIT1*	Zinc finger, HIT type 1
2036	IMAGp998I03413	2.75	0.866	*N/A*	Data not found
2037	IMAGp998E204273	2.75	0.940	*MBP*	Myelin basic protein
2038	IMAGp998G224493	2.75	0.942	*INSL3*	Insulin-like 3 (Leydig cell)
2039	IMAGp998E175467	2.75	0.863	*RNF141*	Ring finger protein 141
2040	IMAGp998F19639	2.75	0.942	*WDR35*	WD repeat domain 35
2041	IMAGp998B164585	2.75	0.914	*N/A*	Transcribed locus
2042	IMAGp998G011115	2.75	0.911	*DRG1*	Developmentally regulated GTP binding protein 1
2043	RZPDp202E037D	2.75	0.919	*CTSW*	Cathepsin W
2044	RZPDp1096D1214D	2.75	0.837	*TMEM14B*	Transmembrane protein 14B
2045	IMAGp998A191945	2.75	0.918	*N/A*	Primary neuroblastoma cDNA, clone:Nbla11051, full insert sequence
2046	RZPDp201A0531D	2.75	0.889	*CIB2*	Calcium and integrin binding family member 2
2047	IMAGp998I095495	2.75	0.880	*N/A*	Transcribed locus
2048	IMAGp998M05217	2.75	0.761	*RASEF*	RAS and EF-hand domain containing
2049	IMAGp998B23224	2.75	0.877	*SOS2*	Son of sevenless homolog 2 (Drosophila)
2050	IMAGp998E135290	2.75	0.881	*PCGF5*	Polycomb group ring finger 5
2051	RZPDp1096A0621D	2.75	0.924	*LOC283116*	Similar to Tripartite motif protein 49 (RING finger protein 18) (Testis-specific ring-finger protein)
2052	IMAGp998H234858	2.75	0.913	*N/A*	Transcribed locus
2053	IMAGp998P01248	2.75	0.867	*DKFZP564O0523*	Hypothetical protein DKFZp564O0523
2054	IMAGp998E233820	2.75	0.859	*N/A*	Transcribed locus
2055	IMAGp998K07371	2.75	0.909	*RUNX1T1*	Runt-related transcription factor 1; translocated to, 1 (cyclin D-related)
2056	IMAGp998K131785	2.75	0.939	*N/A*	Transcribed locus
2057	IMAGp998I044921	2.75	0.896	*HLA-DOB*	Major histocompatibility complex, class II, DO beta
2058	IMAGp998K15679	2.75	0.866	*LOC339803*	Hypothetical protein LOC339803
2059	IMAGp998N105462	2.75	0.880	*N/A*	Transcribed locus
2060	RZPDp201C0730D	2.75	0.897	*RASA3*	RAS p21 protein activator 3
2061	IMAGp998M025587	2.75	0.876	*PPIL2*	Peptidylprolyl isomerase (cyclophilin)-like 2
2062	IMAGp998J05150	2.75	0.867	*SNX15*	Sorting nexin 15
2063	IMAGp998J05208	2.75	0.863	*TRIM58*	Tripartite motif-containing 58
2064	IMAGp998H191864	2.75	0.907	*BSN*	Bassoon (presynaptic cytomatrix protein)
2065	IMAGp998O124335	2.75	0.895	*CHIT1*	Chitinase 1 (chitotriosidase)
2066	IMAGp998I07976	2.75	0.873	*N/A*	Data not found
2067	IMAGp998A015655	2.75	0.868	*LOC652968*	Hypothetical protein LOC652968
2068	IMAGp998N041743	2.75	0.860	*N/A*	Transcribed locus
2069	IMAGp998H205263	2.75	0.911	*GIMAP2*	GTPase, IMAP family member 2
2070	RZPDp201E0115D	2.75	0.871	*GTPBP3*	GTP binding protein 3 (mitochondrial)
2071	IMAGp998O1772	2.75	0.883	*ENTPD6*	Ectonucleoside triphosphate diphosphohydrolase 6 (putative function)
2072	IMAGp998A165383	2.75	0.931	*CTNNBL1*	Catenin, beta like 1
2073	IMAGp998O16420	2.75	0.909	*UBN1*	Ubinuclein 1
2074	IMAGp998H202012	2.75	0.867	*N/A*	Transcribed locus
2075	IMAGp998H17442	2.75	0.883	*FLJ25778*	Hypothetical protein FLJ25778
2076	RZPDp201H1115D	2.75	0.865	*C3orf34*	Chromosome 3 open reading frame 34
2077	IMAGp998B165319	2.75	0.854	*N/A*	Transcribed locus
2078	IMAGp998J11788	2.75	0.935	*VAMP5*	Vesicle-associated membrane protein 5 (myobrevin)
2079	IMAGp998D19532	2.75	0.926	*SLC22A7*	Solute carrier family 22 (organic anion transporter), member 7
2080	IMAGp998H216139	2.75	0.869	*N/A*	Transcribed locus
2081	RZPDp1096B112D	2.75	0.862	*PPFIA4*	Protein tyrosine phosphatase, receptor type, f polypeptide (PTPRF), interacting protein (liprin), alpha 4
2082	RZPDp201D0136D	2.75	0.886	*NASP*	Nuclear autoantigenic sperm protein (histone-binding)
2083	IMAGp998P19155	2.75	0.873	*NRG3*	Neuregulin 3
2084	IMAGp998A055391	2.75	0.875	*PIGG*	Phosphatidylinositol glycan anchor biosynthesis, class G
2085	IMAGp998M015615	2.75	0.876	*N/A*	Transcribed locus
2086	IMAGp998J06268	2.75	0.882	*THAP3*	THAP domain containing, apoptosis associated protein 3
2087	RZPDp201D034D	2.75	0.888	*TUBA3E*	Tubulin, alpha 3e
2088	IMAGp998M10277	2.75	0.876	*N/A*	CDNA clone IMAGE:4821984
2089	IMAGp998K123980	2.75	0.907	*N/A*	Data not found
2090	IMAGp998M185472	2.75	0.881	*TMTC3*	Transmembrane and tetratricopeptide repeat containing 3
2091	RZPDp201F1231D	2.75	0.918	*RHBDD2*	Rhomboid domain containing 2
2092	IMAGp998E12742	2.75	0.914	*MYL6B*	Myosin, light chain 6B, alkali, smooth muscle and non-muscle
2093	IMAGp998I241999	2.75	0.863	*HNRPU*	Heterogeneous nuclear ribonucleoprotein U (scaffold attachment factor A)
2094	IMAGp998A02697	2.75	0.909	*N/A*	Transcribed locus
2095	IMAGp998B21879	2.75	0.852	*N/A*	Transcribed locus
2096	IMAGp998N11650	2.75	0.929	*DAXX*	Death-associated protein 6
2097	IMAGp998A045212	2.75	0.872	*N/A*	Data not found
2098	RZPDp202G069D	2.75	0.921	*GCNT4*	Glucosaminyl (N-acetyl) transferase 4, core 2 (beta-1,6-N-acetylglucosaminyltransferase)
2099	IMAGp998F11274	2.75	0.876	*RFX1*	Regulatory factor X, 1 (influences HLA class II expression)
2100	IMAGp998B05287	2.75	0.906	*N/A*	Transcribed locus
2101	IMAGp998H105676	2.75	0.873	*N/A*	Transcribed locus
2102	RZPDp202H127D	2.75	0.917	*GLG1*	Golgi apparatus protein 1
2103	IMAGp998A06115	2.75	0.919	*ALDOB*	Aldolase B, fructose-bisphosphate
2104	IMAGp998A031937	2.75	0.902	*HOXA4*	Homeobox A4
2105	IMAGp998M055781	2.75	0.929	*C17orf46*	Chromosome 17 open reading frame 46
2106	IMAGp998J096100	2.75	0.870	*PFAAP5*	Phosphonoformate immuno-associated protein 5
2107	IMAGp998O10173	2.75	0.889	*PGM2L1*	Phosphoglucomutase 2-like 1
2108	IMAGp998O13601	2.75	0.877	*N/A*	Data not found
2109	RZPDp202E107D	2.75	0.931	*BATF*	Basic leucine zipper transcription factor, ATF-like
2110	IMAGp998L063863	2.75	0.931	*N/A*	Data not found
2111	IMAGp998K113901	2.75	0.863	*N/A*	Data not found
2112	IMAGp998B143729	2.75	0.895	*N/A*	Transcribed locus
2113	IMAGp998C09339	2.75	0.927	*PBX1*	Pre-B-cell leukemia homeobox 1
2114	IMAGp998F16386	2.75	0.885	*N/A*	Transcribed locus, weakly similar to NP_689672.2 protein LOC146556 [Homo sapiens]
2115	RZPDp202H069D	2.75	0.873	*GPR110*	G protein-coupled receptor 110
2116	IMAGp998J05373	2.75	0.862	*SNAP23*	Synaptosomal-associated protein, 23kDa
2117	IMAGp998D23141	2.75	0.867	*N/A*	Transcribed locus
2118	RZPDp202A084D	2.75	0.918	*TRAFD1*	TRAF-type zinc finger domain containing 1
2119	IMAGp998I215655	2.75	0.877	*N/A*	Transcribed locus
2120	IMAGp998J0617	2.75	0.868	*N/A*	Transcribed locus
2121	IMAGp998A055395	2.75	0.881	*SIRT5*	Sirtuin (silent mating type information regulation 2 homolog) 5 (S. cerevisiae)
2122	IMAGp998D09833	2.75	0.903	*N/A*	Transcribed locus
2123	IMAGp998G194974	2.75	0.915	*N/A*	Transcribed locus
2124	IMAGp998G134944	2.75	0.895	*N/A*	Transcribed locus
2125	IMAGp998N24234	2.75	0.906	*TCF21*	Transcription factor 21
2126	IMAGp998C184356	2.75	0.858	*N/A*	Data not found
2127	IMAGp998O03464	2.75	0.898	*LOC400451*	Hypothetical gene supported by AK075564; BC060873
2128	IMAGp998P135591	2.75	0.851	*RFX1*	Regulatory factor X, 1 (influences HLA class II expression)
2129	IMAGp998P205166	2.75	0.928	*HERC2*	Hect domain and RLD 2
2130	IMAGp998K243945	2.75	0.877	*N/A*	Transcribed locus
2131	RZPDp202G024D	2.75	0.888	*QPCTL*	Glutaminyl-peptide cyclotransferase-like
2132	IMAGp998O241822	2.75	0.925	*TTC16*	Tetratricopeptide repeat domain 16
2133	IMAGp998M05399	2.75	0.899	*CLU*	Clusterin
2134	IMAGp998A115595	2.75	0.919	*DUSP5P*	Dual specificity phosphatase 5 pseudogene
2135	IMAGp998N01358	2.75	0.866	*N/A*	Data not found
2136	IMAGp998M045391	2.75	0.924	*N/A*	Transcribed locus
2137	IMAGp998G13692	2.75	0.884	*NPR3*	Natriuretic peptide receptor C/guanylate cyclase C (atrionatriuretic peptide receptor C)
2138	IMAGp998M202307	2.75	0.894	*TRPV1*	Transient receptor potential cation channel, subfamily V, member 1
2139	IMAGp998A22374	2.75	0.870	*N/A*	Transcribed locus
2140	IMAGp998A04378	2.75	0.866	*N/A*	CDNA FLJ12206 fis, clone MAMMA1000941
2141	IMAGp998H085557	2.75	0.888	*N/A*	Data not found
2142	IMAGp998B025677	2.75	0.849	*MAGOH*	Mago-nashi homolog, proliferation-associated (Drosophila)
2143	RZPDp1096E042D	2.75	0.806	*ACSL1*	Acyl-CoA synthetase long-chain family member 1
2144	RZPDp201G0726D	2.75	0.879	*DKFZP434A0131*	DKFZp434A0131 protein
2145	IMAGp998L084887	2.75	0.899	*N/A*	Transcribed locus
2146	IMAGp998N215966	2.75	0.943	*WWP2*	WW domain containing E3 ubiquitin protein ligase 2
2147	IMAGp998M16387	2.75	0.898	*C11orf59*	Chromosome 11 open reading frame 59
2148	IMAGp998P10405	2.75	0.866	*N/A*	Transcribed locus
2149	IMAGp998N13411	2.75	0.878	*N/A*	Transcribed locus
2150	RZPDp202H068D	2.75	0.867	*JMJD5*	Jumonji domain containing 5
2151	IMAGp998A051817	2.75	0.868	*N/A*	Data not found
2152	IMAGp998F244736	2.75	0.861	*N/A*	Data not found
2153	IMAGp998N061743	2.75	0.901	*N/A*	Transcribed locus
2154	IMAGp998A035283	2.75	0.877	*N/A*	MRNA; cDNA DKFZp564G103 (from clone DKFZp564G103)
2155	IMAGp998M03196	2.75	0.876	*N/A*	Transcribed locus
2156	IMAGp998O052014	2.75	0.870	*WDR75*	WD repeat domain 75
2157	IMAGp998I16312	2.75	0.894	*TGOLN2*	Trans-golgi network protein 2
2158	IMAGp998I195399	2.75	0.889	*VPS16*	Vacuolar protein sorting 16 homolog (S. cerevisiae)
2159	IMAGp998E06373	2.75	0.854	*N/A*	Data not found
2160	IMAGp998B23793	2.75	0.937	*MUSTN1*	Musculoskeletal, embryonic nuclear protein 1

2161	IMAGp998J211999	2.75	0.897	*N/A*	Transcribed locus
2162	RZPDp202C059D	2.75	0.942	*PLEKHH2*	Pleckstrin homology domain containing, family H (with MyTH4 domain) member 2
2163	IMAGp998H235773	2.75	0.881	*N/A*	CDNA FLJ12727 fis, clone NT2RP2000027
2164	RZPDp201F0420D	2.75	0.945	*P2RXL1*	Purinergic receptor P2X-like 1, orphan receptor
2165	IMAGp998N09560	2.75	0.910	*TNRC6A*	Trinucleotide repeat containing 6A
2166	RZPDp201A1132D	2.75	0.904	*CUL1*	Cullin 1
2167	IMAGp998M051198	2.75	0.867	*N/A*	Transcribed locus
2168	RZPDp202G084D	2.75	0.861	*MYOM3*	Myomesin family, member 3
2169	IMAGp998F151858	2.75	0.925	*C21orf2*	Chromosome 21 open reading frame 2
2170	IMAGp998K225519	2.75	0.882	*RFT1*	RFT1 homolog (S. cerevisiae)
2171	IMAGp998H233944	2.75	0.911	*N/A*	CDNA FLJ44692 fis, clone BRACE3013986
2172	IMAGp998M065414	2.75	0.859	*N/A*	CDNA FLJ11750 fis, clone HEMBA1005568
2173	IMAGp998L03214	2.75	0.884	*N/A*	Transcribed locus
2174	IMAGp998J01174	2.75	0.881	*AP1GBP1*	AP1 gamma subunit binding protein 1
2175	IMAGp998L036083	2.75	0.896	*UTP6*	UTP6, small subunit (SSU) processome component, homolog (yeast)
2176	IMAGp998F21565	2.75	0.897	*SGCD*	Sarcoglycan, delta (35kDa dystrophin-associated glycoprotein)
2177	IMAGp998H01118	2.75	0.866	*N/A*	Data not found
2178	IMAGp998N111775	2.75	0.888	*FLJ39378*	Hypothetical protein FLJ39378
2179	IMAGp998O22144	2.75	0.861	*MAP3K5*	Mitogen-activated protein kinase kinase kinase 5
2180	RZPDp202H109D	2.75	0.868	*FLJ44379*	Similar to S-100 protein, alpha chain
2181	IMAGp998F242841	2.75	0.872	*N/A*	Transcribed locus
2182	IMAGp998I202380	2.75	0.870	*N/A*	Transcribed locus
2183	IMAGp998A015263	2.75	0.864	*N/A*	Transcribed locus
2184	IMAGp998H224500	2.75	0.944	*RNF190*	Ring finger protein 190
2185	IMAGp998O23147	2.75	0.887	*SPTAN1*	Spectrin, alpha, non-erythrocytic 1 (alpha-fodrin)
2186	IMAGp998N044513	2.75	0.920	*N/A*	Transcribed locus
2187	IMAGp998K015595	2.75	0.905	*N/A*	Transcribed locus
2188	IMAGp998B08175	2.75	0.772	*C3*	Complement component 3
2189	IMAGp998D09384	3.76	0.876	*N/A*	Transcribed locus
2190	IMAGp998M065772	3.76	0.917	*N/A*	Transcribed locus, weakly similar to NP_001032811.2 disintegrin and metalloprotease domain 15 (metargidin) isoform a [Mus musculus]
2191	IMAGp998E084899	3.76	0.912	*N/A*	Transcribed locus
2192	IMAGp998I215468	3.76	0.884	*N/A*	Transcribed locus
2193	IMAGp998A18791	3.76	0.875	*PUM1*	Pumilio homolog 1 (Drosophila)
2194	IMAGp998A03366	3.76	0.869	*KPNB1*	Karyopherin (importin) beta 1
2195	IMAGp998B15147	3.76	0.889	*TTK*	TTK protein kinase
2196	RZPDp202H073D	3.76	0.913	*RBM19*	RNA binding motif protein 19
2197	IMAGp998L1369	3.76	0.880	*N/A*	Clone 25153 mRNA sequence
2198	IMAGp998J226076	3.76	0.882	*N/A*	Transcribed locus
2199	IMAGp998C083938	3.76	0.781	*TXNIP*	Thioredoxin interacting protein
2200	RZPDp201G0225D	3.76	0.901	*LILRB3*	Leukocyte immunoglobulin-like receptor, subfamily B (with TM and ITIM domains), member 3
2201	IMAGp998N10167	3.76	0.907	*N/A*	MRNA; cDNA DKFZp686L15210 (from clone DKFZp686L15210)
2202	IMAGp998L095449	3.76	0.888	*FOXA2*	Forkhead box A2
2203	IMAGp998D135800	3.76	0.918	*LOC730184*	Hypothetical protein LOC730184
2204	RZPDp202G031D	3.76	0.866	*GTPBP5*	GTP binding protein 5 (putative)
2205	IMAGp998J18166	3.76	0.874	*OLFM2*	Olfactomedin 2
2206	RZPDp202A069D	3.76	0.921	*ZNF41*	Zinc finger protein 41
2207	IMAGp998I18117	3.76	0.871	*N/A*	Transcribed locus
2208	IMAGp998O035494	3.76	0.884	*N/A*	CDNA FLJ11764 fis, clone HEMBA1005685
2209	IMAGp998B234148	3.76	0.907	*N/A*	Clone HLS_IMAGE_1634998 mRNA sequence
2210	IMAGp998P18176	3.76	0.890	*SYNGR2*	Synaptogyrin 2
2211	RZPDp202D125D	3.76	0.907	*IFRD2*	Interferon-related developmental regulator 2
2212	IMAGp998I086077	3.76	0.887	*C20orf28*	Chromosome 20 open reading frame 28
2213	IMAGp998O13188	3.76	0.889	*N/A*	CDNA FLJ44142 fis, clone THYMU2016523
2214	IMAGp998B104174	3.76	0.917	*KIAA1086*	KIAA1086
2215	IMAGp998K074502	3.76	0.949	*N/A*	Transcribed locus
2216	IMAGp998A11266	3.76	0.882	*N/A*	Transcribed locus
2217	IMAGp998D084496	3.76	0.863	*EML4*	Echinoderm microtubule associated protein like 4
2218	RZPDp1096B022D	3.76	0.868	*KPNB1*	Karyopherin (importin) beta 1
2219	IMAGp998M12660	3.76	0.863	*SERINC3*	Serine incorporator 3
2220	IMAGp998E24687	3.76	0.935	*CDIPT*	CDP-diacylglycerol--inositol 3-phosphatidyltransferase (phosphatidylinositol synthase)
2221	RZPDp202E078D	3.76	0.921	*ARF3*	ADP-ribosylation factor 3
2222	IMAGp998A1471	3.76	0.901	*CD59*	CD59 molecule, complement regulatory protein
2223	RZPDp1096B0514D	3.76	0.911	*LOC441748*	Similar to NPIP gene
2224	IMAGp998N091854	3.76	0.878	*SPTY2D1*	SPT2, Suppressor of Ty, domain containing 1 (S. cerevisiae)
2225	IMAGp998I12240	3.76	0.908	*METT11D1*	Methyltransferase 11 domain containing 1
2226	RZPDp202H095D	3.76	0.881	*IGF2R*	Insulin-like growth factor 2 receptor
2227	IMAGp998L095594	3.76	0.895	*N/A*	CDNA FLJ43943 fis, clone TESTI4014306
2228	IMAGp998K052640	3.76	0.880	*N/A*	CDNA clone IMAGE:4825606
2229	IMAGp998E244069	3.76	0.896	*N/A*	Transcribed locus
2230	IMAGp998B226076	3.76	0.868	*N/A*	Transcribed locus
2231	IMAGp998I23413	3.76	0.903	*NFATC4*	Nuclear factor of activated T-cells, cytoplasmic, calcineurin-dependent 4
2232	RZPDp201G045D	3.76	0.933	*TMEM180*	Transmembrane protein 180
2233	IMAGp998C225317	3.76	0.876	*N/A*	Transcribed locus
2234	IMAGp998E105470	3.76	0.911	*PHF20L1*	PHD finger protein 20-like 1
2235	IMAGp998E235458	3.76	0.918	*N/A*	Data not found
2236	RZPDp1096C0815D	3.76	0.911	*OPN4*	Opsin 4 (melanopsin)
2237	IMAGp998N14836	3.76	0.927	*LMAN1*	Lectin, mannose-binding, 1
2238	IMAGp998K164574	3.76	0.915	*GEM*	GTP binding protein overexpressed in skeletal muscle
2239	IMAGp998P171011	3.76	0.876	*PRDM10*	PR domain containing 10
2240	IMAGp998D16203	3.76	0.869	*N/A*	Data not found
2241	IMAGp998M09612	3.76	0.910	*EGR3*	Early growth response 3
2242	IMAGp998F24252	3.76	0.932	*GALNS*	Galactosamine (N-acetyl)-6-sulfate sulfatase (Morquio syndrome, mucopolysaccharidosis type IVA)
2243	IMAGp998O08596	3.76	0.903	*N/A*	Data not found
2244	IMAGp998E11165	3.76	0.853	*GJA4*	Gap junction protein, alpha 4, 37kDa
2245	IMAGp998N211892	3.76	0.907	*N/A*	CDNA FLJ36867 fis, clone ASTRO2016491
2246	IMAGp998K194519	3.76	0.906	*N/A*	Transcribed locus
2247	IMAGp998I10385	3.76	0.842	*ALDH9A1*	Aldehyde dehydrogenase 9 family, member A1
2248	IMAGp998G15123	3.76	0.933	*FXYD2*	FXYD domain containing ion transport regulator 2
2249	IMAGp998N201889	3.76	0.936	*RMND5B*	Required for meiotic nuclear division 5 homolog B (S. cerevisiae)
2250	IMAGp998D041197	3.76	0.944	*LYPD3*	LY6/PLAUR domain containing 3
2251	IMAGp998L141821	3.76	0.897	*N/A*	Transcribed locus, weakly similar to NP_001039706.1 protein LOC518880 [Bos taurus]
2252	IMAGp998F18538	3.76	0.921	*ZNF532*	Zinc finger protein 532
2253	IMAGp998F03267	3.76	0.877	*UNQ9217*	AASA9217
2254	IMAGp998G245717	3.76	0.932	*INPP4A*	Inositol polyphosphate-4-phosphatase, type I, 107kDa
2255	IMAGp998J084408	3.76	0.882	*N/A*	Transcribed locus
2256	IMAGp998O20209	3.76	0.939	*MAGI1*	Membrane associated guanylate kinase, WW and PDZ domain containing 1
2257	RZPDp202E032D	3.76	0.899	*ZNF79*	Zinc finger protein 79
2258	IMAGp998H121817	3.76	0.866	*TSNAX*	Translin-associated factor X
2259	IMAGp998D06841	3.76	0.948	*N/A*	Homo sapiens, clone IMAGE:5168282, mRNA
2260	IMAGp998N035602	3.76	0.881	*HOXB4*	Homeobox B4
2261	RZPDp202F092D	3.76	0.924	*LOC55565*	Hypothetical protein LOC55565
2262	RZPDp202E034D	3.76	0.869	*NEK8*	NIMA (never in mitosis gene a)- related kinase 8
2263	IMAGp998I20670	3.76	0.854	*C1R*	Complement component 1, r subcomponent
2264	RZPDp202G105D	3.76	0.868	*TIRAP*	Toll-interleukin 1 receptor (TIR) domain containing adaptor protein
2265	RZPDp201H077D	3.76	0.884	*N/A*	Transcribed locus
2266	IMAGp998K145486	3.76	0.914	*N/A*	Transcribed locus
2267	RZPDp202H045D	3.76	0.867	*FAM3D*	Family with sequence similarity 3, member D
2268	IMAGp998P20281	3.76	0.883	*EPHX1*	Epoxide hydrolase 1, microsomal (xenobiotic)
2269	RZPDp202F122D	3.76	0.922	*CAMTA2*	Calmodulin binding transcription activator 2
2270	IMAGp998P08154	3.76	0.884	*NAT5*	N-acetyltransferase 5
2271	IMAGp998G095285	3.76	0.875	*N/A*	Transcribed locus
2272	RZPDp202E093D	3.76	0.873	*TNFRSF9*	Tumor necrosis factor receptor superfamily, member 9
2273	RZPDp201C0414D	3.76	0.933	*SPINK2*	Serine peptidase inhibitor, Kazal type 2 (acrosin-trypsin inhibitor)
2274	IMAGp998F025242	3.76	0.859	*N/A*	Data not found
2275	IMAGp998J084892	3.76	0.883	*EPB41L5*	Erythrocyte membrane protein band 4.1 like 5
2276	RZPDp202H054D	3.76	0.908	*BIRC7*	Baculoviral IAP repeat-containing 7 (livin)
2277	IMAGp998N12644	3.76	0.900	*N/A*	Transcribed locus
2278	IMAGp998C055391	3.76	0.913	*NTNG1*	Netrin G1
2279	IMAGp998O051942	3.76	0.887	*RP5-875H10.1*	SAM domain containing 1
2280	IMAGp998G014881	3.76	0.898	*NDOR1*	NADPH dependent diflavin oxidoreductase 1
2281	IMAGp998A09270	3.76	0.900	*ENG*	Endoglin (Osler-Rendu-Weber syndrome 1)
2282	RZPDp201A1215D	3.76	0.880	*TTC33*	Tetratricopeptide repeat domain 33
2283	IMAGp998J015636	3.76	0.923	*ABHD4*	Abhydrolase domain containing 4
2284	RZPDp201A0527D	3.76	0.873	*MFN1*	Mitofusin 1
2285	IMAGp998I04170	3.76	0.860	*N/A*	Transcribed locus
2286	IMAGp998H065383	3.76	0.933	*ATHL1*	ATH1, acid trehalase-like 1 (yeast)
2287	IMAGp998B113191	3.76	0.861	*N/A*	Transcribed locus
2288	RZPDp1096D0215D	3.76	0.938	*CBX8*	Chromobox homolog 8 (Pc class homolog, Drosophila)
2289	IMAGp998A155287	3.76	0.884	*RREB1*	Ras responsive element binding protein 1
2290	IMAGp998B135495	3.76	0.879	*N/A*	Transcribed locus
2291	IMAGp998D13110	3.76	0.887	*BECN1*	Beclin 1 (coiled-coil, myosin-like BCL2 interacting protein)
2292	IMAGp998P164172	3.76	0.894	*N/A*	Transcribed locus
2293	IMAGp998E044899	3.76	0.871	*PCDHB13*	Protocadherin beta 13
2294	IMAGp998O08234	3.76	0.902	*TH1L*	TH1-like (Drosophila)
2295	IMAGp998F062228	3.76	0.894	*N/A*	Transcribed locus

2296	IMAGp998P05174	3.76	0.772	*NCOA4*	Nuclear receptor coactivator 4
2297	RZPDp202C0410D	3.76	0.883	*N/A*	Data not found
2298	RZPDp202D129D	3.76	0.907	*TNPO2*	Transportin 2 (importin 3, karyopherin beta 2b)
2299	IMAGp998M06255	3.76	0.908	*ITGA11*	Integrin, alpha 11
2300	RZPDp1096B046D	3.76	0.890	*CD59*	CD59 molecule, complement regulatory protein
2301	IMAGp998F195610	3.76	0.871	*N/A*	Transcribed locus
2302	IMAGp998C225652	3.76	0.886	*BRSK1*	BR serine/threonine kinase 1
2303	RZPDp1096C0515D	3.76	0.947	*CLDN19*	Claudin 19
2304	RZPDp201E1118D	3.76	0.845	*LOC728320*	Similar to lactotransferrin
2305	IMAGp998H13626	3.76	0.874	*ACBD5*	Acyl-Coenzyme A binding domain containing 5
2306	IMAGp998N044258	3.76	0.910	*N/A*	Transcribed locus
2307	IMAGp998E13737	3.76	0.933	*FAM79A*	Family with sequence similarity 79, member A
2308	IMAGp998N16588	3.76	0.946	*MYO9B*	Myosin IXB
2309	RZPDp201D098D	3.76	0.933	*BTBD9*	BTB (POZ) domain containing 9
2310	IMAGp998P07417	3.76	0.874	*N/A*	Transcribed locus
2311	RZPDp1096B016D	3.76	0.868	*N/A*	Data not found
2312	IMAGp998J075672	3.76	0.870	*TMEM157*	Transmembrane protein 157
2313	IMAGp998O0274	3.76	0.885	*FTO*	Fatso
2314	RZPDp201F0630D	3.76	0.866	*MT2A*	Metallothionein 2A
2315	IMAGp998K205601	3.76	0.916	*BYSL*	Bystin-like
2316	IMAGp998N10270	3.76	0.914	*N/A*	In multiple clusters
2317	IMAGp998G16626	3.76	0.876	*N/A*	CDNA FLJ33715 fis, clone BRAWH2008577
2318	RZPDp201B0535D	3.76	0.869	*PRPSAP1*	Phosphoribosyl pyrophosphate synthetase-associated protein 1
2319	IMAGp998I063860	3.76	0.908	*HIST1H3E*	Histone cluster 1, H3e
2320	IMAGp998N211889	3.76	0.945	*HPS1*	Hermansky-Pudlak syndrome 1
2321	IMAGp998L093910	3.76	0.947	*N/A*	Transcribed locus
2322	IMAGp998O154178	3.76	0.895	*ASXL1*	Additional sex combs like 1 (Drosophila)
2323	IMAGp998E075595	3.76	0.916	*N/A*	Transcribed locus
2324	IMAGp998C215323	3.76	0.901	*DUSP2*	Dual specificity phosphatase 2
2325	IMAGp998G22317	3.76	0.915	*SEMA6D*	Sema domain, transmembrane domain (TM), and cytoplasmic domain, (semaphorin) 6D
2326	RZPDp201B0228D	3.76	0.879	*C12orf32*	Chromosome 12 open reading frame 32
2327	RZPDp201A0514D	3.76	0.885	*NDUFS3*	NADH dehydrogenase (ubiquinone) Fe-S protein 3, 30kDa (NADH-coenzyme Q reductase)
2328	IMAGp998L09132	3.76	0.856	*RUNDC2B*	RUN domain containing 2B
2329	IMAGp998D09542	3.76	0.880	*N/A*	Transcribed locus
2330	IMAGp998J18373	3.76	0.887	*CALM1*	Calmodulin 1 (phosphorylase kinase, delta)
2331	IMAGp998H07312	3.76	0.909	*PRDX5*	Peroxiredoxin 5
2332	IMAGp998D125629	3.76	0.883	*N/A*	Data not found
2333	RZPDp201C0629D	3.76	0.870	*MAGEA1*	Melanoma antigen family A, 1 (directs expression of antigen MZ2-E)
2334	RZPDp201A011D	3.76	0.874	*SPAG17*	Sperm associated antigen 17
2335	RZPDp1096C0314D	3.76	0.904	*TWSG1*	Twisted gastrulation homolog 1 (Drosophila)
2336	IMAGp998A094416	3.76	0.905	*N/A*	Transcribed locus, strongly similar to XP_001169671.1 hypothetical protein [Pan troglodytes]
2337	IMAGp998O13473	3.76	0.854	*N/A*	Data not found
2338	IMAGp998I056074	3.76	0.876	*HNRPR*	Heterogeneous nuclear ribonucleoprotein R
2339	IMAGp998E18440	3.76	0.876	*N/A*	Data not found
2340	RZPDp202A032D	3.76	0.874	*N/A*	Data not found
2341	IMAGp998J105780	3.76	0.925	*N/A*	Transcribed locus
2342	IMAGp998A10473	3.76	0.935	*SLC24A6*	Solute carrier family 24 (sodium/potassium/calcium exchanger), member 6
2343	IMAGp998L205371	3.76	0.866	*N/A*	Transcribed locus
2344	IMAGp998L246127	3.76	0.875	*GPR123*	G protein-coupled receptor 123
2345	IMAGp998C20729	3.76	0.914	*TTC8*	Tetratricopeptide repeat domain 8
2346	RZPDp202G028D	3.76	0.874	*RUNDC2B*	RUN domain containing 2B
2347	IMAGp998L11920	3.76	0.902	*PCBP3*	Poly(rC) binding protein 3
2348	RZPDp201C0831D	3.76	0.869	*KCNE3*	Potassium voltage-gated channel, Isk-related family, member 3
2349	IMAGp998E236101	3.76	0.882	*C11orf17*	Chromosome 11 open reading frame 17
2350	IMAGp998E115290	3.76	0.872	*CST1*	Cystatin SN
2351	IMAGp998J06146	3.76	0.905	*CACNB4*	Calcium channel, voltage-dependent, beta 4 subunit
2352	IMAGp998C08166	3.76	0.876	*MGC2752*	Hypothetical protein MGC2752
2353	IMAGp998N22159	3.76	0.870	*TMED10*	Transmembrane emp24-like trafficking protein 10 (yeast)
2354	RZPDp201A0828D	3.76	0.876	*POLR1C*	Polymerase (RNA) I polypeptide C, 30kDa
2355	IMAGp998L074319	3.76	0.881	*N/A*	In multiple clusters
2356	IMAGp998O21884	3.76	0.934	*INSM2*	Insulinoma-associated 2
2357	IMAGp998D134160	3.76	0.878	*N/A*	Transcribed locus
2358	IMAGp998P205580	3.76	0.866	*N/A*	Transcribed locus
2359	RZPDp1096B1118D	3.76	0.886	*ZNF588*	Zinc finger protein 588
2360	IMAGp998P014327	3.76	0.940	*N/A*	Transcribed locus
2361	IMAGp998C162270	3.76	0.879	*N/A*	Transcribed locus
2362	IMAGp998N055653	3.76	0.876	*ZC3H12D*	Zinc finger CCCH-type containing 12D
2363	IMAGp998P11699	3.76	0.897	*N/A*	Transcribed locus
2364	IMAGp998F21295	3.76	0.886	*VPS72*	Vacuolar protein sorting 72 homolog (S. cerevisiae)
2365	RZPDp1096C0213D	3.76	0.885	*N/A*	Data not found
2366	IMAGp998M224016	3.76	0.881	*N/A*	Transcribed locus
2367	RZPDp202G095D	3.76	0.927	*C19orf28*	Chromosome 19 open reading frame 28
2368	IMAGp998J225617	3.76	0.885	*N/A*	Transcribed locus
2369	IMAGp998A17213	3.76	0.930	*KLHL22*	Kelch-like 22 (Drosophila)
2370	IMAGp998A01654	3.76	0.902	*N/A*	Data not found
2371	IMAGp998K09246	3.76	0.873	*LOC731986*	Similar to cytochrome P450 monooxygenase CYP2T1
2372	IMAGp998P23282	3.76	0.881	*N/A*	Transcribed locus
2373	RZPDp202G018D	3.76	0.896	*HSD3B7*	Hydroxy-delta-5-steroid dehydrogenase, 3 beta- and steroid delta-isomerase 7
2374	RZPDp202C013D	3.76	0.875	*FLJ13236*	Hypothetical protein FLJ13236
2375	IMAGp998K096084	3.76	0.897	*N/A*	Transcribed locus
2376	RZPDp202C123D	3.76	0.891	*LOC146909*	Hypothetical protein LOC146909
2377	IMAGp998L035717	3.76	0.935	*N/A*	Transcribed locus
2378	IMAGp998I21730	3.76	0.896	*CXorf34*	Chromosome X open reading frame 34
2379	IMAGp998P15590	3.76	0.899	*N/A*	In multiple clusters
2380	IMAGp998B155635	3.76	0.893	*TBC1D2*	TBC1 domain family, member 2
2381	IMAGp998K17160	3.76	0.882	*SYT1*	Synaptotagmin I
2382	IMAGp998K175635	3.76	0.894	*SCNN1B*	Sodium channel, nonvoltage-gated 1, beta (Liddle syndrome)
2383	IMAGp998A044914	3.76	0.889	*N/A*	CDNA FLJ41019 fis, clone UTERU2019096
2384	IMAGp998L02114	3.76	0.880	*N/A*	Transcribed locus
2385	RZPDp202H094D	3.76	0.890	*TGIF2*	TGFB-induced factor homeobox 2
2386	IMAGp998H13780	3.76	0.887	*GMPPA*	GDP-mannose pyrophosphorylase A
2387	IMAGp998G205509	3.76	0.921	*GALK2*	Galactokinase 2
2388	IMAGp998C204954	3.76	0.941	*FLJ44896*	FLJ44896 protein
2389	RZPDp202G091D	3.76	0.880	*KCNK13*	Potassium channel, subfamily K, member 13
2390	RZPDp1096C0620D	3.76	0.902	*GJA5*	Gap junction protein, alpha 5, 40kDa
2391	IMAGp998N096072	3.76	0.881	*N/A*	Transcribed locus
2392	RZPDp1096C106D	3.76	0.900	*NMNAT2*	Nicotinamide nucleotide adenylyltransferase 2
2393	IMAGp998N193343	3.76	0.933	*CST3*	Cystatin C (amyloid angiopathy and cerebral hemorrhage)
2394	RZPDp201G086D	3.76	0.918	*FLAD1*	FAD1 flavin adenine dinucleotide synthetase homolog (S. cerevisiae)
2395	IMAGp998N125766	3.76	0.882	*N/A*	Transcribed locus
2396	RZPDp201A1231D	3.76	0.898	*PER3*	Period homolog 3 (Drosophila)
2397	IMAGp998H244004	3.76	0.950	*ESPNL*	Espin-like
2398	IMAGp998M22170	3.76	0.923	*N/A*	Transcribed locus, moderately similar to XP_508499.1 similar to hypothetical protein [Pan troglodytes]
2399	IMAGp998E21545	3.76	0.907	*GTDC1*	Glycosyltransferase-like domain containing 1
2400	IMAGp998P081964	3.76	0.887	*DNAJC14*	DnaJ (Hsp40) homolog, subfamily C, member 14
2401	IMAGp998K09975	3.76	0.869	*N/A*	Transcribed locus
2402	IMAGp998F134165	3.76	0.937	*C9orf79*	Chromosome 9 open reading frame 79
2403	IMAGp998B015598	3.76	0.888	*TTC21B*	Tetratricopeptide repeat domain 21B
2404	IMAGp998L184576	3.76	0.886	*GALM*	Galactose mutarotase (aldose 1-epimerase)
2405	IMAGp998G20469	3.76	0.945	*RTKN*	Rhotekin
2406	IMAGp998D23152	3.76	0.890	*LOC89944*	Hypothetical protein BC008326
2407	IMAGp998L205761	3.76	0.901	*DENND1C*	DENN/MADD domain containing 1C
2408	IMAGp998F155608	3.76	0.885	*N/A*	Transcribed locus, strongly similar to XP_001147093.1 hypothetical protein isoform 2 [Pan troglodytes]
2409	IMAGp998I10274	3.76	0.894	*N/A*	MRNA; cDNA DKFZp686G08203 (from clone DKFZp686G08203)
2410	IMAGp998O034640	3.76	0.908	*OXNAD1*	Oxidoreductase NAD-binding domain containing 1
2411	IMAGp998G195877	3.76	0.912	*ZNF224*	Zinc finger protein 224
2412	IMAGp998P20152	3.76	0.865	*C1orf95*	Chromosome 1 open reading frame 95
2413	IMAGp998J09869	3.76	0.875	*DLX6*	Distal-less homeobox 6
2414	RZPDp201D021D	3.76	0.887	*GPR21*	G protein-coupled receptor 21
2415	IMAGp998H13266	3.76	0.880	*WDR61*	WD repeat domain 61
2416	IMAGp998D13678	3.76	0.902	*MYLPF*	Fast skeletal myosin light chain 2
2417	IMAGp998I223967	3.76	0.922	*N/A*	Transcribed locus
2418	RZPDp1096A113D	3.76	0.927	*TRPM1*	Transient receptor potential cation channel, subfamily M, member 1
2419	IMAGp998K124150	3.76	0.892	*PGPEP1*	Pyroglutamyl-peptidase I
2420	RZPDp201C0418D	3.76	0.875	*NBPF1*	Neuroblastoma breakpoint family, member 1
2421	IMAGp998A241870	3.76	0.908	*N/A*	Transcribed locus
2422	IMAGp998D01580	3.76	0.910	*MUM1*	Melanoma associated antigen (mutated) 1
2423	IMAGp998D04133	3.76	0.879	*MRPS18B*	Mitochondrial ribosomal protein S18B
2424	IMAGp998O03618	3.76	0.899	*FLJ44894*	Similar to zinc finger protein 91
2425	IMAGp998J095284	3.76	0.897	*STYX*	Serine/threonine/tyrosine interacting protein
2426	RZPDp201G0619D	3.76	0.897	*NUBPL*	Nucleotide binding protein-like
2427	IMAGp998H16435	3.76	0.874	*N/A*	Transcribed locus
2428	IMAGp998J095378	3.76	0.918	*SOCS6*	Suppressor of cytokine signaling 6
2429	RZPDp202H098D	3.76	0.904	*CLSTN3*	Calsyntenin 3
2430	IMAGp998O06789	3.76	0.903	*GPSM3*	G-protein signalling modulator 3 (AGS3-like, C. elegans)

2431	RZPDp1096G107D	3.76	0.871	REEP5	Receptor accessory protein 5
2432	RZPDp201E1129D	3.76	0.935	C12orf52	Chromosome 12 open reading frame 52
2433	IMAGp998C14241	3.76	0.916	PRSS8	Protease, serine, 8
2434	RZPDp202A129D	3.76	0.930	PLEKHG4	Pleckstrin homology domain containing, family G (with RhoGef domain) member 4
2435	RZPDp201C097D	3.76	0.911	CORO6	Coronin 6
2436	IMAGp998N12404	3.76	0.907	MPP1	Membrane protein, palmitoylated 1, 55kDa
2437	IMAGp998O226081	3.76	0.870	N/A	Transcribed locus
2438	IMAGp998C113293	3.76	0.880	N/A	Transcribed locus
2439	IMAGp998H104209	3.76	0.926	N/A	Transcribed locus
2440	IMAGp998M073715	3.76	0.889	N/A	Data not found
2441	RZPDp201C1036D	3.76	0.925	ARPC1B	Actin related protein 2/3 complex, subunit 1B, 41kDa
2442	RZPDp201A124D	3.76	0.949	SLC22A14	Solute carrier family 22 (organic cation transporter), member 14
2443	IMAGp998M015310	3.76	0.882	N/A	Transcribed locus
2444	IMAGp998F06610	3.76	0.895	TXNL1	Thioredoxin-like 1
2445	IMAGp998C244308	3.76	0.884	LOC400713	Zinc finger-like
2446	IMAGp998O1470	3.76	0.883	SLC4A4	Solute carrier family 4, sodium bicarbonate cotransporter, member 4
2447	RZPDp201G048D	3.76	0.951	LGALS9	Lectin, galactoside-binding, soluble, 9 (galectin 9)
2448	IMAGp998O115175	3.76	0.936	N/A	Transcribed locus
2449	RZPDp1096A0313D	3.76	0.859	HSP90AA2	Heat shock protein 90kDa alpha (cytosolic), class A member 2
2450	IMAGp998I0689	3.76	0.860	N/A	Transcribed locus
2451	IMAGp998F242009	3.76	0.922	LOC92196	Death-associated protein-like 1
2452	IMAGp998A06365	3.76	0.901	WDR13	WD repeat domain 13
2453	RZPDp202G058D	3.76	0.902	DDB1	Damage-specific DNA binding protein 1, 127kDa
2454	IMAGp998D115389	3.76	0.892	N/A	CDNA FLJ41109 fis, clone BLADE2009452
2455	IMAGp998E05658	3.76	0.907	N/A	Data not found
2456	RZPDp201D1218D	3.76	0.927	PCYT1A	Phosphate cytidylyltransferase 1, choline, alpha
2457	IMAGp998C063949	3.76	0.885	N/A	Data not found
2458	RZPDp202D062D	3.76	0.916	KLHL21	Kelch-like 21 (Drosophila)
2459	IMAGp998C185465	3.76	0.894	N/A	Transcribed locus
2460	IMAGp998D18113	3.76	0.886	ORC1L	Origin recognition complex, subunit 1-like (yeast)
2461	IMAGp998L134872	3.76	0.887	N/A	Transcribed locus
2462	IMAGp998P24378	3.76	0.895	N/A	Transcribed locus, moderately similar to XP_529427.1 hypothetical protein XP_529427 [Pan troglodytes]
2463	RZPDp1096C0719D	3.76	0.879	LRRC57	Leucine rich repeat containing 57
2464	IMAGp998F011924	3.76	0.912	LOC400027	Hypothetical gene supported by BC047417
2465	IMAGp998E014359	3.76	0.871	N/A	Transcribed locus
2466	RZPDp201C094D	3.76	0.903	ZUBR1	Zinc finger, UBR1 type 1
2467	RZPDp201G0416D	3.76	0.922	MPZL1	Myelin protein zero-like 1
2468	IMAGp998G2018	3.76	0.904	N/A	CDNA FLJ32348 fis, clone PROST2007200
2469	IMAGp998I102226	3.76	0.882	N/A	Transcribed locus
2470	IMAGp998C245514	3.76	0.891	LOC729967	Hypothetical protein LOC729967
2471	RZPDp201C1235D	3.76	0.902	TAX1BP1	Tax1 (human T-cell leukemia virus type I) binding protein 1
2472	RZPDp201A061D	3.76	0.928	CCL16	Chemokine (C-C motif) ligand 16
2473	IMAGp998H10156	3.76	0.942	LRRC4	Leucine rich repeat containing 4
2474	RZPDp1096A0119D	3.76	0.882	FOSL1	FOS-like antigen 1
2475	IMAGp998O045469	3.76	0.879	PDPK1	3-phosphoinositide dependent protein kinase-1
2476	IMAGp998C025064	3.76	0.949	N/A	Data not found
2477	IMAGp998H01658	3.76	0.858	N/A	Transcribed locus
2478	IMAGp998M081935	3.76	0.926	GPR125	G protein-coupled receptor 125
2479	IMAGp998O03269	3.76	0.883	JARID1A	Jumonji, AT rich interactive domain 1A
2480	IMAGp998B115205	3.76	0.921	N/A	Transcribed locus
2481	IMAGp998F056098	3.76	0.884	WDR72	WD repeat domain 72
2482	IMAGp998N225777	4.84	0.920	KIAA1432	KIAA1432
2483	IMAGp998N19565	4.84	0.914	PKNOX1	PBX/knotted 1 homeobox 1
2484	IMAGp998G066074	4.84	0.900	N/A	Transcribed locus
2485	IMAGp998O10781	4.84	0.914	LPHN1	Latrophilin 1
2486	IMAGp998D17325	4.84	0.940	FAM19A5	Family with sequence similarity 19 (chemokine (C-C motif)-like), member A5
2487	IMAGp998J14591	4.84	0.930	ARMC9	Armadillo repeat containing 9
2488	RZPDp201A062D	4.84	0.887	TUBA4B	Tubulin, alpha 4b
2489	RZPDp201C118D	4.84	0.913	AREG	Amphiregulin (schwannoma-derived growth factor)
2490	IMAGp998B184115	4.84	0.886	STON2	Stonin 2
2491	IMAGp998E24269	4.84	0.893	MAP4	Microtubule-associated protein 4
2492	IMAGp998H04417	4.84	0.899	GLI2	GLI-Kruppel family member GLI2
2493	IMAGp998O173560	4.84	0.902	N/A	Data not found
2494	IMAGp998B134113	4.84	0.898	N/A	Full length insert cDNA clone ZD75H06
2495	IMAGp998E054894	4.84	0.900	N/A	CDNA FLJ43589 fis, clone SKNSH2010015
2496	IMAGp998K175766	4.84	0.910	UNC5C	Unc-5 homolog C (C. elegans)
2497	RZPDp1096H0515D	4.84	0.907	IRAK2	Interleukin-1 receptor-associated kinase 2
2498	IMAGp998M245946	4.84	0.911	N/A	Transcribed locus
2499	IMAGp998E134616	4.84	0.945	N/A	Transcribed locus
2500	IMAGp998C055261	4.84	0.920	PPM1A	Protein phosphatase 1A (formerly 2C), magnesium-dependent, alpha isoform
2501	IMAGp998O081775	4.84	0.950	MFN2	Mitofusin 2
2502	IMAGp998D051166	4.84	0.888	LUC7L2	LUC7-like 2 (S. cerevisiae)
2503	IMAGp998C11139	4.84	0.893	CLIC1	Chloride intracellular channel 1
2504	IMAGp998J21247	4.84	0.910	LRP2	Low density lipoprotein-related protein 2
2505	IMAGp998N215667	4.84	0.909	NTN1	Netrin 1
2506	IMAGp998M141945	4.84	0.945	GALT	Galactose-1-phosphate uridylyltransferase
2507	IMAGp998G214497	4.84	0.886	N/A	Transcribed locus
2508	IMAGp998M164246	4.84	0.877	N/A	Data not found
2509	IMAGp998N035615	4.84	0.876	TMEM30A	Transmembrane protein 30A
2510	IMAGp998M211962	4.84	0.951	N/A	Transcribed locus, strongly similar to XP_523598.1 similar to tousled-like kinase 2; serine/threonine kinase; tousled-like kinase [Pan troglodytes]
2511	IMAGp998D11523	4.84	0.805	ASS1	Argininosuccinate synthetase 1
2512	IMAGp998M052584	4.84	0.892	N/A	Transcribed locus
2513	IMAGp998K061869	4.84	0.883	N/A	Transcribed locus
2514	IMAGp998P114129	4.84	0.874	N/A	Data not found
2515	RZPDp201C1213D	4.84	0.902	MAGEB4	Melanoma antigen family B, 4
2516	IMAGp998A055756	4.84	0.923	N/A	Data not found
2517	RZPDp202C078D	4.84	0.887	CDT1	Chromatin licensing and DNA replication factor 1
2518	IMAGp998C211112	4.84	0.888	ST8SIA4	ST8 alpha-N-acetyl-neuraminide alpha-2,8-sialyltransferase 4
2519	IMAGp998P24268	4.84	0.870	ZNF576	Zinc finger protein 576
2520	IMAGp998O145145	4.84	0.951	N/A	Transcribed locus
2521	IMAGp998O184548	4.84	0.908	HELB	Helicase (DNA) B
2522	IMAGp998E084519	4.84	0.921	N/A	Transcribed locus
2523	IMAGp998M10237	4.84	0.931	ELN	Elastin (supravalvular aortic stenosis, Williams-Beuren syndrome)
2524	IMAGp998B11175	4.84	0.936	N/A	Transcribed locus
2525	RZPDp202G074D	4.84	0.925	LOC339483	Hypothetical LOC339483
2526	IMAGp998E19161	4.84	0.920	N/A	Transcribed locus
2527	IMAGp998D076106	4.84	0.876	N/A	Transcribed locus
2528	IMAGp998H19382	4.84	0.936	TRAF4	TNF receptor-associated factor 4
2529	IMAGp998D02437	4.84	0.892	N/A	Data not found
2530	IMAGp998A134513	4.84	0.953	N/A	Data not found
2531	IMAGp998M194870	4.84	0.884	N/A	Transcribed locus
2532	RZPDp202C015D	4.84	0.866	PMS2CL	PMS2-C terminal-like
2533	IMAGp998P15782	4.84	0.892	N/A	Transcribed locus
2534	IMAGp998N184411	4.84	0.913	LOC730085	Hypothetical protein LOC730085
2535	RZPDp201H1226D	4.84	0.896	CXXC5	CXXC finger 5
2536	IMAGp998D105729	4.84	0.886	RPL3L	Ribosomal protein L3-like
2537	RZPDp1096F0816D	4.84	0.890	TNFRSF21	Tumor necrosis factor receptor superfamily, member 21
2538	IMAGp998J17145	4.84	0.918	NOTCH1	Notch homolog 1, translocation-associated (Drosophila)
2539	RZPDp1096A0817D	4.84	0.939	ZFYVE27	Zinc finger, FYVE domain containing 27
2540	RZPDp201B1232D	4.84	0.932	C9orf156	Chromosome 9 open reading frame 156
2541	IMAGp998C11885	4.84	0.899	LOC54103	Hypothetical protein LOC54103
2542	IMAGp998O01202	4.84	0.903	DKFZp779O175	Hypothetical protein DKFZp779O175
2543	RZPDp1096G053D	4.84	0.919	IL32	Interleukin 32
2544	IMAGp998D104538	4.84	0.920	N/A	Transcribed locus, moderately similar to XP_510320.2 INO80 complex homolog 1 [Pan troglodytes]
2545	IMAGp998N241859	4.84	0.932	C11orf2	Chromosome 11 open reading frame2
2546	RZPDp202A117D	4.84	0.913	DVL1	Dishevelled, dsh homolog 1 (Drosophila)
2547	RZPDp201H0616D	4.84	0.924	MYBPH	Myosin binding protein H
2548	IMAGp998C086061	4.84	0.918	CXCL6	Chemokine (C-X-C motif) ligand 6 (granulocyte chemotactic protein 2)
2549	IMAGp998J111166	4.84	0.870	TRAK1	Trafficking protein, kinesin binding 1
2550	RZPDp1096E116D	4.84	0.894	N/A	In multiple clusters
2551	IMAGp998O041825	4.84	0.929	N/A	Data not found
2552	IMAGp998D075394	4.84	0.936	YTHDC1	YTH domain containing 1
2553	IMAGp998B11310	4.84	0.944	N/A	Transcribed locus, moderately similar to NP_060312.1 protein LOC55652 [Homo sapiens]
2554	IMAGp998C19155	4.84	0.928	DDO	D-aspartate oxidase
2555	IMAGp998I2117	4.84	0.897	GRIA4	Glutamate receptor, ionotrophic, AMPA 4
2556	IMAGp998C102840	4.84	0.914	N/A	Transcribed locus
2557	IMAGp998H134030	4.84	0.938	ITPR1	Inositol 1,4,5-triphosphate receptor, type 1
2558	IMAGp998M161858	4.84	0.928	C14orf161	Chromosome 14 open reading frame 161
2559	RZPDp201C1220D	4.84	0.884	TCTA	T-cell leukemia translocation altered gene
2560	RZPDp202A112D	4.84	0.832	ZNF599	Zinc finger protein 599
2561	IMAGp998D121778	4.84	0.936	RXRB	Retinoid X receptor, beta
2562	IMAGp998C213343	4.84	0.914	N/A	Transcribed locus
2563	IMAGp998L08257	4.84	0.937	APOL2	Apolipoprotein L, 2
2564	IMAGp998P22678	4.84	0.894	TMEM16E	Transmembrane protein 16E
2565	IMAGp998B1273	4.84	0.911	TACC2	Transforming, acidic coiled-coil containing protein 2

2566	IMAGp998C044415	4.84	0.906	*OSR2*	Odd-skipped related 2 (Drosophila)
2567	IMAGp998D11664	4.84	0.891	*E2F8*	E2F transcription factor 8
2568	IMAGp998P192228	4.84	0.895	*SLC12A8*	Solute carrier family 12 (potassium/chloride transporters), member 8
2569	IMAGp998N195511	4.84	0.879	*R3HCC1*	R3H domain and coiled-coil containing 1
2570	IMAGp998P245940	4.84	0.912	*N/A*	Transcribed locus
2571	IMAGp998L145525	4.84	0.898	*N/A*	Transcribed locus
2572	IMAGp998F14579	4.84	0.935	*POLR2A*	Polymerase (RNA) II (DNA directed) polypeptide A, 220kDa
2573	RZPDp202A091D	4.84	0.889	*MGC3771*	Hypothetical protein MGC3771
2574	IMAGp998F17140	4.84	0.897	*N/A*	Data not found
2575	RZPDp1096H057D	4.84	0.906	*ATF4*	Activating transcription factor 4 (tax-responsive enhancer element B67)
2576	IMAGp998G181831	4.84	0.914	*N/A*	CDNA clone IMAGE:5272469
2577	IMAGp998N115776	4.84	0.946	*N/A*	Transcribed locus
2578	IMAGp998C1118	4.84	0.882	*N/A*	Homo sapiens, clone IMAGE:5193340, mRNA
2579	RZPDp202G014D	4.84	0.892	*RAB15*	RAB15, member RAS onocogene family
2580	IMAGp998C16472	4.84	0.861	*N/A*	Transcribed locus, moderately similar to XP_001162695.1 transmembrane BAX inhibitor motif containing 4 isoform 4 [Pan troglodytes]
2581	RZPDp201E0619D	4.84	0.894	*FLJ00049*	FLJ00049 protein
2582	IMAGp998N11579	4.84	0.892	*SLC25A16*	Solute carrier family 25 (mitochondrial carrier; Graves disease autoantigen), member 16
2583	IMAGp998G05975	4.84	0.899	*N/A*	Data not found
2584	IMAGp998J125884	4.84	0.893	*N/A*	Transcribed locus
2585	IMAGp998P1481	4.84	0.895	*GNG2*	Guanine nucleotide binding protein (G protein), gamma 2
2586	IMAGp998J211206	4.84	0.917	*FXC1*	Fracture callus 1 homolog (rat)
2587	IMAGp998L055672	4.84	0.921	*PELP1*	Proline, glutamic acid and leucine rich protein 1
2588	IMAGp998M24338	4.84	0.924	*ASB3*	Ankyrin repeat and SOCS box-containing 3
2589	IMAGp998F08139	4.84	0.900	*SIN3A*	SIN3 homolog A, transcription regulator (yeast)
2590	IMAGp998F141749	4.84	0.928	*SEPW1*	Selenoprotein W, 1
2591	IMAGp998G013909	4.84	0.870	*N/A*	Transcribed locus
2592	RZPDp201F1116D	4.84	0.874	*DNAJC5*	DnaJ (Hsp40) homolog, subfamily C, member 5
2593	IMAGp998N092622	4.84	0.914	*N/A*	Transcribed locus, strongly similar to XP_001164180.1 hypothetical protein [Pan troglodytes]
2594	IMAGp998F10679	4.84	0.913	*C15orf17*	Chromosome 15 open reading frame 17
2595	RZPDp202G129D	4.84	0.889	*NUBPL*	Nucleotide binding protein-like
2596	IMAGp998C051019	4.84	0.886	*N/A*	Data not found
2597	RZPDp201E028D	4.84	0.916	*SCN4A*	Sodium channel, voltage-gated, type IV, alpha subunit
2598	IMAGp998H21778	4.84	0.887	*PRPF8*	PRP8 pre-mRNA processing factor 8 homolog (S. cerevisiae)
2599	IMAGp998F092010	4.84	0.888	*ARMCX3*	Armadillo repeat containing, X-linked 3
2600	IMAGp998N19137	4.84	0.923	*SLC11A2*	Solute carrier family 11 (proton-coupled divalent metal ion transporters), member 2
2601	IMAGp998F151861	4.84	0.954	*LCT*	Lactase
2602	IMAGp998M23578	4.84	0.919	*SLMAP*	Sarcolemma associated protein
2603	IMAGp998G10467	4.84	0.892	*FLJ16478*	FLJ16478 protein
2604	IMAGp998J155261	4.84	0.879	*PTPLAD2*	Protein tyrosine phosphatase-like A domain containing 2
2605	IMAGp998F14623	4.84	0.496	*POLE*	Polymerase (DNA directed), epsilon
2606	RZPDp201B033D	4.84	0.895	*BNC2*	Basonuclin 2
2607	IMAGp998F20177	4.84	0.930	*N/A*	MRNA,partial cDNA sequence from cDNA selection, DCR1-17.0,
2608	IMAGp998G131825	4.84	0.791	*TOB2*	Transducer of ERBB2, 2
2609	RZPDp1096C0519D	4.84	0.913	*TMEM164*	Transmembrane protein 164
2610	RZPDp201E0517D	4.84	0.913	*DKFZp434K1815*	Hypothetical protein DKFZp434K1815
2611	IMAGp998B115795	4.84	0.939	*N/A*	Transcribed locus
2612	RZPDp202H088D	4.84	0.926	*TSPAN17*	Tetraspanin 17
2613	RZPDp202E075D	4.84	0.933	*SAMD4B*	Sterile alpha motif domain containing 4B
2614	RZPDp201A074D	4.84	0.913	*CCDC83*	Coiled-coil domain containing 83
2615	RZPDp201G0517D	4.84	0.937	*PORCN*	Porcupine homolog (Drosophila)
2616	IMAGp998P041999	4.84	0.890	*N/A*	Transcribed locus
2617	IMAGp998F023725	4.84	0.922	*N/A*	Transcribed locus
2618	IMAGp998D094458	4.84	0.901	*N/A*	Transcribed locus
2619	RZPDp202C111D	4.84	0.941	*NUFIP1*	Nuclear fragile X mental retardation protein interacting protein 1
2620	RZPDp201B1019D	4.84	0.872	*CTSL1*	Cathepsin L1
2621	IMAGp998G215551	4.84	0.944	*N/A*	Transcribed locus
2622	RZPDp202D111D	4.84	0.916	*CUTL2*	Cut-like 2 (Drosophila)
2623	RZPDp201F0131D	4.84	0.901	*PSMD8*	Proteasome (prosome, macropain) 26S subunit, non-ATPase, 8
2624	IMAGp998G194012	4.84	0.923	*ARFGEF2*	ADP-ribosylation factor guanine nucleotide-exchange factor 2 (brefeldin A-inhibited)
2625	IMAGp998M222003	4.84	0.938	*FAM101A*	Family with sequence similarity 101, member A
2626	IMAGp998A11560	4.84	0.892	*N/A*	In multiple clusters
2627	RZPDp201D1217D	4.84	0.871	*ELOVL7*	ELOVL family member 7, elongation of long chain fatty acids (yeast)
2628	IMAGp998J171206	4.84	0.887	*NIT1*	Nitrilase 1
2629	IMAGp998F22334	4.84	0.918	*HECW1*	HECT, C2 and WW domain containing E3 ubiquitin protein ligase 1
2630	IMAGp998D211786	4.84	0.935	*N/A*	Transcribed locus
2631	RZPDp201A069D	4.84	0.912	*FLJ44968*	FLJ44968 protein
2632	RZPDp201D0932D	4.84	0.919	*NSF*	N-ethylmaleimide-sensitive factor
2633	RZPDp202C025D	4.84	0.937	*FLJ39609*	Hypothetical protein FLJ39609
