# Supplementary material for: Differential expression of apoptotic genes PDIA3 and MAP3K5 distinguishes between low- and high-risk prostate cancer
Source: Mol Cancer. 2009 Dec 27;8:130. doi: 10.1186/1476-4598-8-130 (PMC2807430; doi:10.1186/1476-4598-8-130)
Supplement: Additional file 3 — Differentially expressed genes between tumors of high versus low Gleason score across independent datasets. Intersection of differentially expressed genes between high- and low risk prostate cancer with microarray data of True et al. 2006 [6] and Lapointe et al. 2004 [7]. [file 1476-4598-8-130-S3.PDF]

Additional File 3: Differentially expressed genes between tumors of high versus low Gleason score across independent datasets.

Intersection with True et al. 2006 [6]

| RZPD ID | Gene Symbol Pressinotti et al. | Gene Symbol True et al. [6] | Tendency |
|---|---|---|---|
| RZPDp202E078D | *ARF3* | *ARF3* | up |
| RZPDp1096A051D | *ATP6V1F* | *ATP6V1F* | up |
| IMAGp998D22407 | *AZGP1* | *AZGP1* | down |
| RZPDp201B1229D | *CAPZA2* | *CAPZA2* | up |
| IMAGp998O0186 | *COX6C* | *COX6C* | up |
| IMAGp998P04272 | *COX7A2L* | *COX7A2L* | up |
| RZPDp201E123D | *DAD1* | *DAD1* | up |
| IMAGp998B1472 | *GNAS* | *C20orf45* | up |
| RZPDp201F0834D | *HMGB1* | *HMGB1* | up |
| RZPDp201H0327D | *HSD17B4* | *HSD17B4* | up |
| IMAGp998G02615 | *MYBPC1* | *MYBPC1* | down |
| IMAGp998L18825 | *NDUFB3* | *NDUFB3* | up |
| IMAGp998K18671 | *NME1* | *NME1* | up |
| RZPDp201B0633D | *PCCB* | *PCCB* | up |
| IMAGp998H07312 | *PRDX5* | *PRDX5* | up |
| IMAGp998M22367 | *RAB2A* | *RAB2* | up |
| IMAGp998M22367 | *RHOA* | *RHOA* | up |
| IMAGp998M20388 | *SLC22A3* | *SLC22A3* | down |
| IMAGp998C16178 | *SNX3* | *SNX3* | up |
| RZPDp201D0836D | *SPCS1* | *SPC12* | up |
| RZPDp202D1010D | *SUB1* | *PC4* | up |
| IMAGp998A01140 | *TCEA1* | *TCEA1* | up |
| IMAGp998K06152 | *YBX1* | *NSEP1* | up |
| IMAGp998M13132 | *YWHAZ* | *YWHAZ* | up |

Intersection with Lapointe et al. 2004 [7]

| RZPD ID | Gene Symbol Pressinotti et al. | Gene Symbol Lapointe et al. [7] | Tendency |
|---|---|---|---|
| IMAGp998K04139 | *SPARC* | *SPARC* | up |
| IMAGp998N21461 | *BGN* | *BGN* | up |
| RZPDp1096C062D | *VCAN* | *CSPG2* | up |
| IMAGp998E21690 | *COL3A1* | *COL3A1* | up |
| IMAGp998D09597 | *COL1A2* | *COL1A2* | up |
| IMAGp998N13665 | *PLA2G2A* | *PLA2G2A* | up |
