# Supplementary material for: Differential expression of apoptotic genes PDIA3 and MAP3K5 distinguishes between low- and high-risk prostate cancer
Source: Mol Cancer. 2009 Dec 27;8:130. doi: 10.1186/1476-4598-8-130 (PMC2807430; doi:10.1186/1476-4598-8-130)
Supplement: Additional file 4 — Selected genes linked to apoptosis and differentially expressed genes between tumors of high versus low Gleason score. Gene ontology analyses (FatiGO, Ingenuity, GOstat) were performed to link differentially expressed genes between high versus low Gleason score to the gene ontology apoptosis (GO:0006915, GO:0008219). Statistical data from the microarray experiment were included. [file 1476-4598-8-130-S4.PDF]

Additional File 4: Selected genes linked to apoptosis and differentially expressed genes between tumors of high versus low Gleason score

No.	Gene Symbol	RZPD ID	q-value	fold change	Gene Name	qRT-PCR Validation
1	*ANXA5*	IMAGp998G1518	0.00	1.47	Annexin A5	no
2	*EI24*	RZPDp1096B0120D	0.00	1.18	Etoposide induced 2.4 mRNA	no
3	*HMGB1*	RZPDp201F0834D	0.00	1.40	High-mobility group box 1	yes
4	*NGFRAP1*	IMAGp998O16794	0.00	1.32	Nerve growth factor receptor (TNFRSF16) associated protein 1	yes
5	*NPM1*	RZPDp202B129D	0.00	1.30	Nucleophosmin (nucleolar phosphoprotein B23, numatrin)	yes
6	*TEGT*	RZPDp1096F101D	0.00	1.32	Testis enhanced gene transcript (BAX inhibitor 1)	yes
7	*TRIAP1*	IMAGp998M191167	0.00	1.34	TP53 regulated inhibitor of apoptosis 1	no
8	*VDAC1*	IMAGp998D08136	0.00	1.51	Voltage-dependent anion channel 1	yes
9	*CYCS*	IMAGp998H09170	0.17	1.22	Cytochrome c, somatic	no
10	*MAP3K1*	IMAGp998N23220	0.17	1.18	Mitogen-activated protein kinase kinase kinase 1	yes
11	*MAP3K5*	IMAGp998O22144	0.17	1.35	Mitogen-activated protein kinase kinase kinase 5	yes
12	*PERP*	IMAGp998F10191	0.17	1.22	PERP, TP53 apoptosis effector	no
13	*ROCK1*	RZPDp201C1129D	0.17	1.17	Rho-associated, coiled-coil containing protein kinase 1	yes
14	*BIRC7*	RZPDp202H054D	0.30	1.13	Baculoviral IAP repeat-containing 7 (livin)	yes
15	*NGFR*	IMAGp998C08143	0.50	1.17	Nerve growth factor receptor (TNFR superfamily, member 16)	yes
16	*NME1*	IMAGp998K18671	0.50	1.16	Non-metastatic cells 1, protein (NM23A) expressed in	yes
17	*SGPP1*	RZPDp1096G056D	0.50	1.13	Sphingosine-1-phosphate phosphotase 2	no
18	*NAIP*	IMAGp998M184415	0.56	1.16	NLR family, apoptosis inhibitory protein	yes
19	*SERINC3*	IMAGp998M12660	0.56	1.28	Serine incorporator 3	no
20	*YWHAZ*	RZPDp202F036D	0.56	1.18	Tyrosine 3-monooxygenase/tryptophan 5-monooxygenase activation protein, zeta polypeptide	yes
21	*HIP1*	IMAGp998F041817	0.94	1.28	Huntingtin interacting protein 1	yes
22	*TNFRSF21*	RZPDp1096F0816D	0.94	1.16	Tumor necrosis factor receptor superfamily, member 21	no
23	*RRAGA*	IMAGp998M03235	1.13	1.14	Sphingosine-1-phosphate phosphatase 1	no
24	*PDIA3*	RZPDp1096G0216D	1.29	1.43	Protein disulfide isomerase family A, member 3	yes
25	*RTN4*	IMAGp998E05168	1.29	1.31	Reticulon 4	yes
26	*VCP*	IMAGp998O10119	1.29	1.18	Valosin-containing protein	yes
27	*DAD1*	RZPDp201E123D	1.52	1.08	Defender against cell death 1	yes
28	*AIFM3*	IMAGp998A042227	1.86	1.44	Apoptosis-inducing factor, mitochondrion-associated, 3	yes
29	*CD74*	IMAGp998M1678	1.86	1.32	CD74 molecule, major histocompatibility complex, class II invariant chain	no
30	*FASLG*	RZPDp202B124D	1.86	1.16	Fas ligand (TNF superfamily, member 6)	no
31	*TP53INP1*	IMAGp998F025462	1.86	1.19	Tumor protein p53 inducible nuclear protein 1	no
32	*NCKAP1*	IMAGp998F09192	2.10	1.11	NCK-associated protein 1	no
33	*PARP4*	IMAGp998B03399	2.10	1.06	Poly (ADP-ribose) polymerase family, member 4	no
34	*PML*	IMAGp998A19870	2.43	1.11	Promyelocytic leukemia	no
35	*EEF1E1*	IMAGp998J10689	3.78	1.60	Eukaryotic translation elongation factor 1 epsilon 1	no
36	*PROC*	IMAGp998K164731	3.78	1.08	Protein C (inactivator of coagulation factors Va and VIIIa)	no
37	*BCL2L2*	IMAGp998D06697	4.29	1.35	BCL2-like 2	yes
38	*MX1*	IMAGp998P231815	4.29	1.15	Myxovirus (influenza virus) resistance 1, interferon-inducible protein p78 (mouse)	no
39	*TRAF4*	RZPDp201H0728D	4.29	1.26	TNF receptor-associated factor 4	yes
40	*DOCK1*	IMAGp998O061827	4.71	0.90	Dedicator of cytokinesis 1	no
41	*MGMT*	IMAGp998D22127	4.71	0.90	O-6-methylguanine-DNA methyltransferase	no
42	*NR2E1*	IMAGp998J01156	4.71	1.09	Nuclear receptor subfamily 2, group E, member 1	yes
43	*P53AIP1*	RZPDp201G075D	4.71	0.79	P53-regulated apoptosis-inducing protein 1	no
44	*STAT1*	RZPDp202E047D	4.71	1.25	Signal transducer and activator of transcription 1, 91kDa	yes
45	*SPP1*	IMAGp998I20178	5.36	1.19	Secreted phosphoprotein 1 (osteopontin, bone sialoprotein I, early T-lymphocyte activation 1)	yes
46	*YARS*	RZPDp201F0228D	5.36	1.10	Tyrosyl-tRNA synthetase	no
