# Supplementary material for: Differential expression of apoptotic genes PDIA3 and MAP3K5 distinguishes between low- and high-risk prostate cancer
Source: Mol Cancer. 2009 Dec 27;8:130. doi: 10.1186/1476-4598-8-130 (PMC2807430; doi:10.1186/1476-4598-8-130)
Supplement: Additional file 5 — Genes analyzed by quantitative real-time PCR. In total, 68 genes were analyzed by qRT-PCR in order to validate expression differences between tumors of high (GS8-10) and low (GS6) Gleason scores. p-values from the statistical analysis are shown. In addition, fold change, mean Ct values of both tumor groups and the primer sequences of the gene-specific assays are indicated. [file 1476-4598-8-130-S5.PDF]

Additional File 5: Genes analyzed by quantitative real-time PCR

No.	RZPD ID	Gene Symbol	p-value (Wilcox	Fold Change	mean Ct value GS 6	mean Ct value GS 8-10	forward primer	reverse primer
1	RZPDp201C1129D	*ROCK1*	0.00	9.76	13.34	10.06	ccctcgaacgctttctaca	gggcactcagtcacatggtt
2	IMAGp998G1518	*ANXA5*	0.00	4.28	8.97	6.88	gctcaagttgaacaagatgctc	tcttcatctgtcccccattt
3	IMAGp998D08136	*VDAC1*	0.00	5.41	11.89	9.45	ttctcacctaacactgggaaaaa	ccagcaatgtcgaaatccat
4	IMAGp998O16794	*NGFRAP1*	0.01	3.95	10.20	8.22	tcggtgcagtcgtcactc	gacacttagcctcgcagacc
5	RZPDp1096F101D	*TEGT*	0.01	2.77	5.30	3.83	tgctggatttgcattcctta	ctggggttgacagcaataca
6	RZPDp202B129D	*NPM1*	0.03	3.07	6.61	4.99	cgccttctctcctacctaagtg	tcggcctttagttcacaacc
7	RZPDp1096G0216D	*PDIA3*	0.03	2.40	6.82	5.56	ctgctaaaggagagaagtttgtca	tctccagagccttcccatc
8	RZPDp201F0834D	*HMGB1*	0.03	4.68	15.28	13.06	gagtgaggaggctgcgtct	tgcccatgtttagttatttttcc
9	IMAGp998D184645	*TMEM16G*	0.04	0.35	16.83	18.33	gctctgtggtgatcgtggt	ggcacggtacaggatgataga
10	IMAGp998I22653	*TMEM69*	0.05	2.77	14.70	13.23	ttcaataggggccagtatgc	gaaagtatatctgttctgctggttctt
11	IMAGp998N13665	*PLA2G2A*	0.05	3.00	5.95	4.36	aaatttctgagctacaagtttagcaac	acactcacacagttgacttctgc
12	IMAGp998D09597	*COL1A2*	0.05	2.02	14.55	13.54	tctggagaggctggtactgc	gagcaccaagaagaccctga
13	IMAGp998L12154	*ECHS1*	0.06	1.89	16.30	15.39	tcaaccaggccctgaagat	caggttctgcatttccttgata
14	RZPDp1096A0718D	*NUB1*	0.06	1.62	11.42	10.73	aaagggatgggctactccac	ccacatctgaggattgctga
15	IMAGp998M03537	*APLP2*	0.06	2.83	13.31	11.81	tggctgtgtgtaaagcgatg	tcgaaatacacatcaacatcattg
16	RZPDp1096A0717D	*6-Mar*	0.07	0.64	10.22	10.88	ggaggaagatgacgctggt	gcattccaattcatgtcatcc
17	IMAGp998O22144	*MAP3K5*	0.07	2.98	10.36	8.78	ttaaaatcttcatggagcaggtc	atttggaacgaaggagagca
18	RZPDp1096C062D	*VCAN*	0.07	2.92	22.79	21.24	gcacctgtgtgccaggata	cagggattagagtgacattcatca
19	IMAGp998D20286	*HADHB*	0.08	0.66	6.00	6.59	ggcttagtggctgcgtgt	ataagcttccactatcatagcatgg
20	IMAGp998D20518	*SAT1*	0.08	0.64	6.44	7.07	gactgttcaagatcgacaagga	tgtcatctacagcagcactcc
21	IMAGp998O10119	*VCP*	0.08	2.75	13.17	11.71	gtgtacgcctaggggatgtc	tggatacgtttgccgtacttc
22	IMAGp998K13587	*CRIM1*	0.09	3.23	18.15	16.46	ccatcaacgtggaaggaag	ttggttggttctgggacatac
23	RZPDp201H0728D	*TRAF4*	0.09	2.59	15.39	14.02	ggaccagcttcctctggact	ggataggcaggcccaatact
24	IMAGp998C021833	*HNRPUL1*	0.11	2.85	19.74	18.23	agctacagccctccacagc	tgtagccctggctgtaacct
25	IMAGp998B06472	*FVT1*	0.13	1.70	11.44	10.67	gggatggctccagtaacttct	aagcaatagtgcggaaaagg
26	IMAGp998L081748	*IFITM3*	0.13	1.87	12.34	11.43	agatgctcaaggaggagcac	gatgtggatcacggtggac
27	IMAGp998M15530	*SRPK1*	0.16	0.62	17.47	18.16	cctcattcaggggaagagtaca	ctgccacaatgagcttgc
28	IMAGp998C20738	*SERPINE1*	0.17	0.72	2.49	2.96	gaggacgaggtggacgtg	ggagcagaagcacttgacttg
29	IMAGp998O05787	*F8A1*	0.18	0.70	4.22	4.72	aagccacgacatgaaaatga	aggctgcgttaggtggaag
30	RZPDp202B088D	*MFAP1*	0.21	2.68	13.56	12.14	caaccgcccattcagtctac	tttttccattgaaatctcacctt
31	IMAGp998E05168	*RTN4*	0.23	3.20	13.71	12.03	gagcctgtgatacgctcctc	ttgaccagccgaaatagtgtt
32	IMAGp998F061005	*CASP3*	0.24	2.26	21.46	20.28	ctggttttcggtgggtgt	cagtgttctccatggatacctttatt
33	RZPDp1096G107D	*REEP5*	0.27	0.85	10.08	10.31	acaggagcttcatcgctctt	gaggctccataaccgaacac
34	IMAGp998J125970	*PRSS23*	0.28	1.71	10.05	9.28	caatgacatcggcatggat	ctcttgtggggctttttgag
35	IMAGp998F16171	*ATRN*	0.37	0.60	15.40	16.14	tggacctggtacagttcttcg	ctttgtttgatcttccaaaccac
36	RZPDp201E123D	*DAD1*	0.37	1.46	6.63	6.09	tggattttcttcagatttgacact	agttgttctgacacacagtgaactc
37	IMAGp998A16170	*PABPC1*	0.38	0.74	14.15	14.57	cttgggctacgcgtatgtg	aattcatggtgtccaaagcac
38	RZPDp202D108D	*IK*	0.39	1.24	5.67	5.36	cgctgaagaagccagaagac	tatccacagccatgtcatcc
39	RZPDp201E0230D	*LRIG1*	0.46	0.66	15.86	16.45	atttcgggcacaatagagga	cacagacttgatcttgtttccaa
40	RZPDp202E047D	*STAT1*	0.53	2.00	20.28	19.28	ctgctcctttggttgaatcc	gctgaagttcgtaccactgaga
41	IMAGp998P10268	*SCYE1*	0.54	1.04	15.79	15.73	gtggctggacctacatgctt	ccaagagacggcagaaaatc
42	IMAGp998D03170	*TMEM123*	0.54	1.17	13.16	12.93	cacaatgaccgtaacccaca	cagaatgcatagttgttgtgattg
43	IMAGp998G09159	*ADAMTS1*	0.54	0.73	16.27	16.72	gctgctccgtcatagaagatg	gcatcatcatgtggcatgtta
44	RZPDp202H054D	*BIRC7*	0.57	0.67	14.97	15.55	gcgcaccttcctgtccta	ggcccagaacacagtcca
45	IMAGp998M221819	*INDO*	0.63	1.27	17.53	17.18	gtgtttcaccaaatccacga	ctgatagctgggggttgc
46	IMAGp998K04139	*SPARC*	0.64	1.17	12.37	12.14	tgcctgggtgttagcaaag	ctccctggactctccacct
47	IMAGp998E12372	*HDGF*	0.66	1.81	10.82	9.96	aggggacttgctggagga	ataggaaggggcctctcaac
48	IMAGp998O19735	*ADIPOR2*	0.73	1.46	16.19	15.64	gggcattgcagccattat	aggcccaaaaacactcctg
49	RZPDp202A056D	*PLAT*	0.83	0.87	14.67	14.87	cgggtggaatattgctggt	cttggctcgctgcaactt
50	IMAGp998D22407	*AZGP1*	0.87	0.80	10.80	11.13	cccaggagaaccaagatgg	cggggacgtcttcaacat
51	IMAGp998K011928	*MAP3K2*	0.87	1.86	18.75	17.85	ggctcaggaagttgtccatc	aggcattcgtgattttggat
52	IMAGp998F041817	*HIP1*	0.92	1.03	13.61	13.57	aagagctggaggattcgttg	cttccagctgttcttgagtcttc
53	IMAGp998B16371	*SMS*	0.94	1.21	16.03	15.75	tgaacaaagtagaggaaagaatgaaa	gtgggccagtatctgtcgat
54	IMAGp998F151749	*USP39*	0.98	1.14	18.30	18.11	aacctccacaccctcaagttt	aagtgggcttcaacacatacg
55	IMAGp998D06697	*BCL2L2*	1.00	0.95	13.04	13.11	tggatggtggcctacctg	cgtccccgtatagagctgtg
56	IMAGp998C15391	*DDX5*	1.00	1.15	15.22	15.02	gccatgtcgggttattcg	ggtttccaaacttctttccaga
57	RZPDp202F076D	*LOXL2*	NA	NA	NA	NA	actgccagctcctcctacg	tcgttgccagtacagtggag
58	IMAGp998N23220	*MAP3K1*	NA	NA	NA	NA	caccaccactgcatgtcaa	acaaaggggacatattaaaggttct
59	IMAGp998M184415	*NAIP*	NA	NA	NA	NA	gattccaaggtgcattcattg	aaatgctctgtccgtcctttag
60	IMAGp998C08143	*NGFR*	NA	NA	NA	NA	tcatccctgtctattgctcca	tgttctgcttgcagctgttc
61	IMAGp998K18671	*NME1*	NA	NA	NA	NA	cagccggagttcaaacctaa	caggctgacttagtcctgtgtaga
62	IMAGp998J01156	*NR2E1*	NA	NA	NA	NA	ggacctatgtctgcaaatctgg	tttctgtgcgtcttgtccac
63	IMAGp998C18473	*REV3L*	NA	NA	NA	NA	ccttgtgaaaaaccgtctcg	atgtcacggacgcaaacc
64	IMAGp998I20178	*SPP1*	NA	NA	NA	NA	gagggcttggttgtcagc	caattctcatggtagtgagttttcc
65	IMAGp998H06241	*SRM*	NA	NA	NA	NA	tgctgtgcagcaagaacc	ggtgcacgtcggagttgta
66	IMAGp998L20376	*WNK1*	NA	NA	NA	NA	caagtatcagctggaaaacagc	tgaggttcgccttggatagt
67	IMAGp998M13132	*YWHAZ*	NA	NA	NA	NA	cgttacttggctgaggttgc	tgcttgttgtgactgatcgac
68	IMAGp998C12887	*TRH*	NA	NA	NA	NA	gggcctggatgacttcct	gagagccagtcagattgaaagat
		B2M	housekeeping gene				ttctggcctggaggctatc	tcaggaaatttgactttccattc

NA: Ct values above detection level
