# Supplementary material for: Differential expression of apoptotic genes PDIA3 and MAP3K5 distinguishes between low- and high-risk prostate cancer
Source: Mol Cancer. 2009 Dec 27;8:130. doi: 10.1186/1476-4598-8-130 (PMC2807430; doi:10.1186/1476-4598-8-130)
Supplement: Additional file 6 — Sequences of siRNAs (Dharmacon smartPool On-Target plus) used for knockdown of PDIA3. [file 1476-4598-8-130-S6.PDF]

Additional File 6: Sequences of siRNAs (Dharmacon smartPool On-Target plus) used for knockdown of PDIA3

siRNA Sequences. ON-TARGETplus
PDIA3 **; LU-003674-00**
siRNA #1; J-003674-09
Target Sequence: GGAAUAGUCCCAUUAGCAA
siRNA #2; J-003674-10
Target Sequence: GGGCAAGGACUUACUUAUU
siRNA #3; J-003674-11
Target Sequence: AGACCCAAAUAUCGUCAUA
siRNA #4; J-003674-12
Target Sequence: GAGGAGUUCUCGCGUGAUG
